# Supplementary figures and images for: T1000: a reduced gene set prioritized for toxicogenomic studies (part 1 of 2)
Source: PeerJ. 2019 Oct 29;7:e7975. doi: 10.7717/peerj.7975 (PMC6824333; doi:10.7717/peerj.7975)

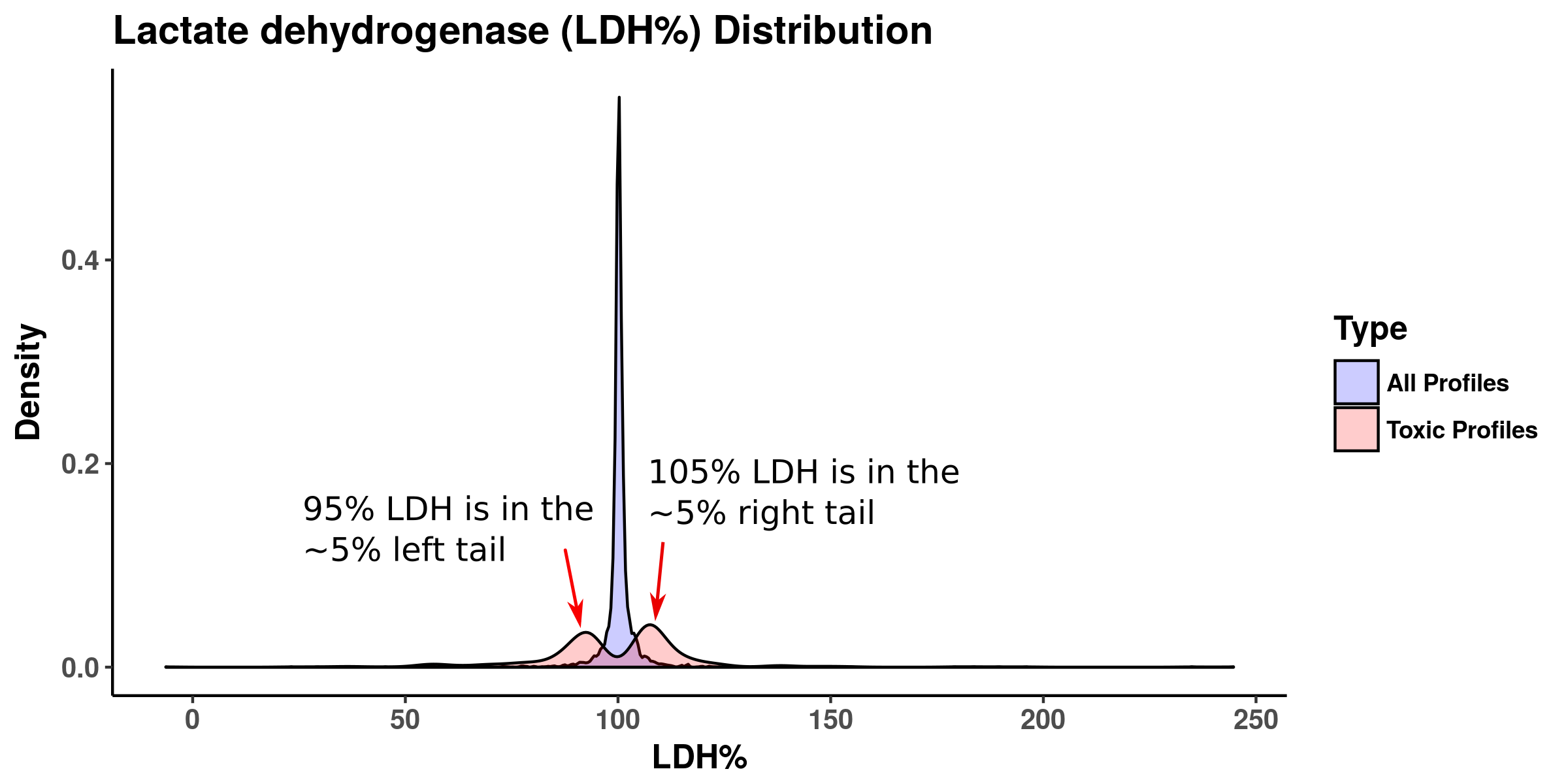

Supplement: Figure S1 — About 86% of experiments were indicated normal in the range of 95%-105% and the remaining 14% were cytotoxic cases. 95% and 105% are cut-offs that appear at 5% of left and right tails, respectively. [file peerj-07-7975-s001.png]

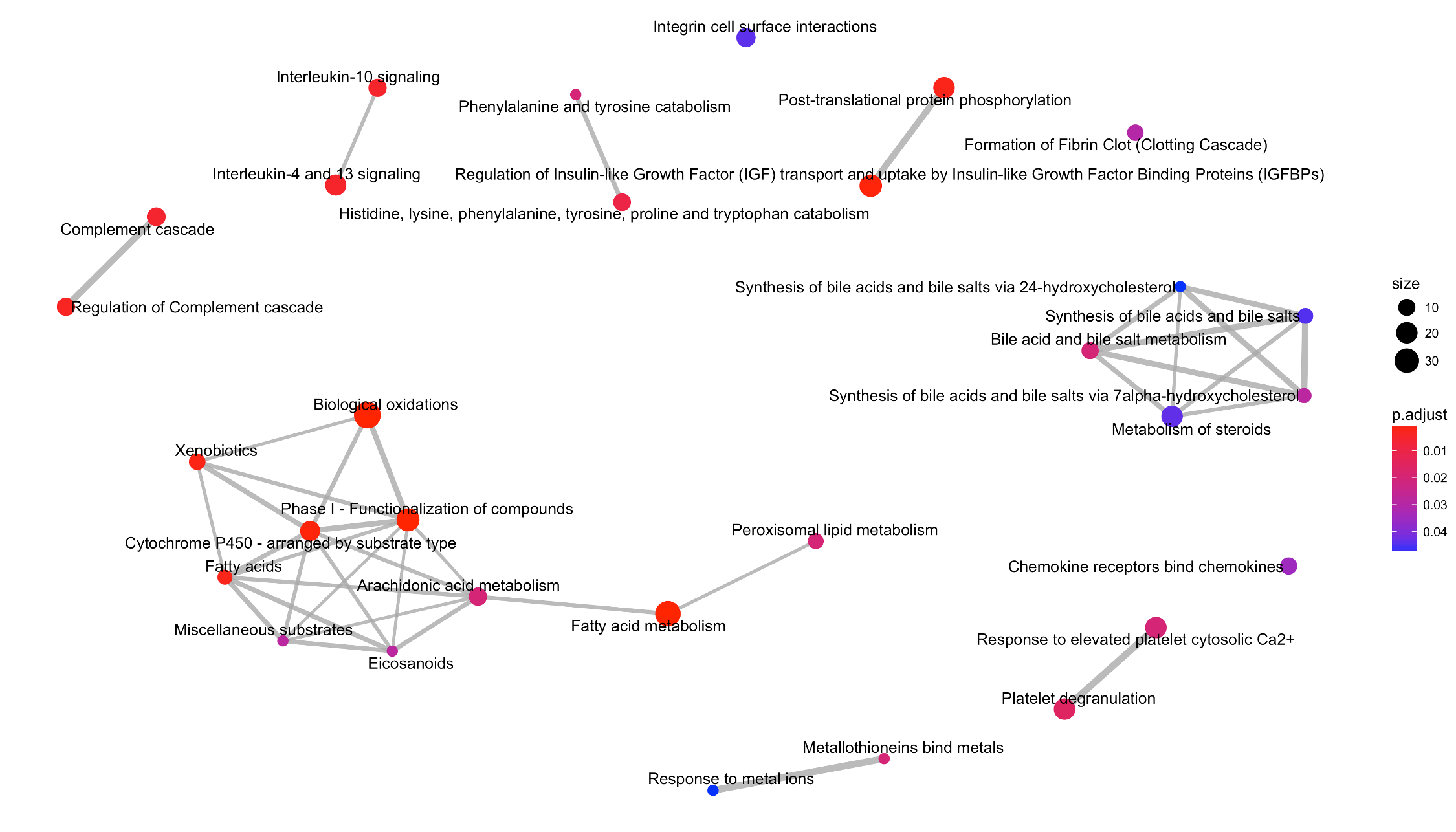

Supplement: Figure S2 — The gradient of colors represents p-adjusted of enrichment, where a high-intensity red color corresponds to more significance for the enriched term. The different sized circles reflect the number of matched genes between T1000 and the enriched reference gene set. The thickness of the edges indicates the ratio of common genes between the enriched gene sets on both sides of the edge. [file peerj-07-7975-s002.png]

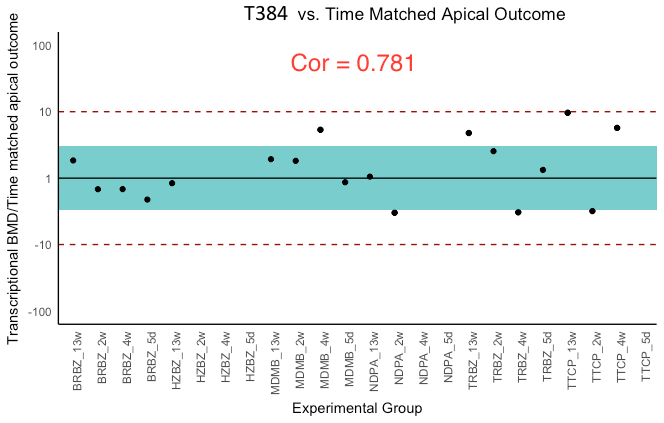

Supplement: Figure S3 [file peerj-07-7975-s003.png]

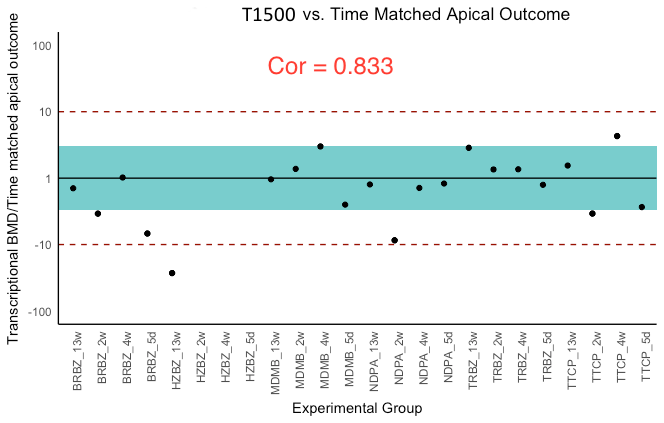

Supplement: Figure S4 [file peerj-07-7975-s004.png]

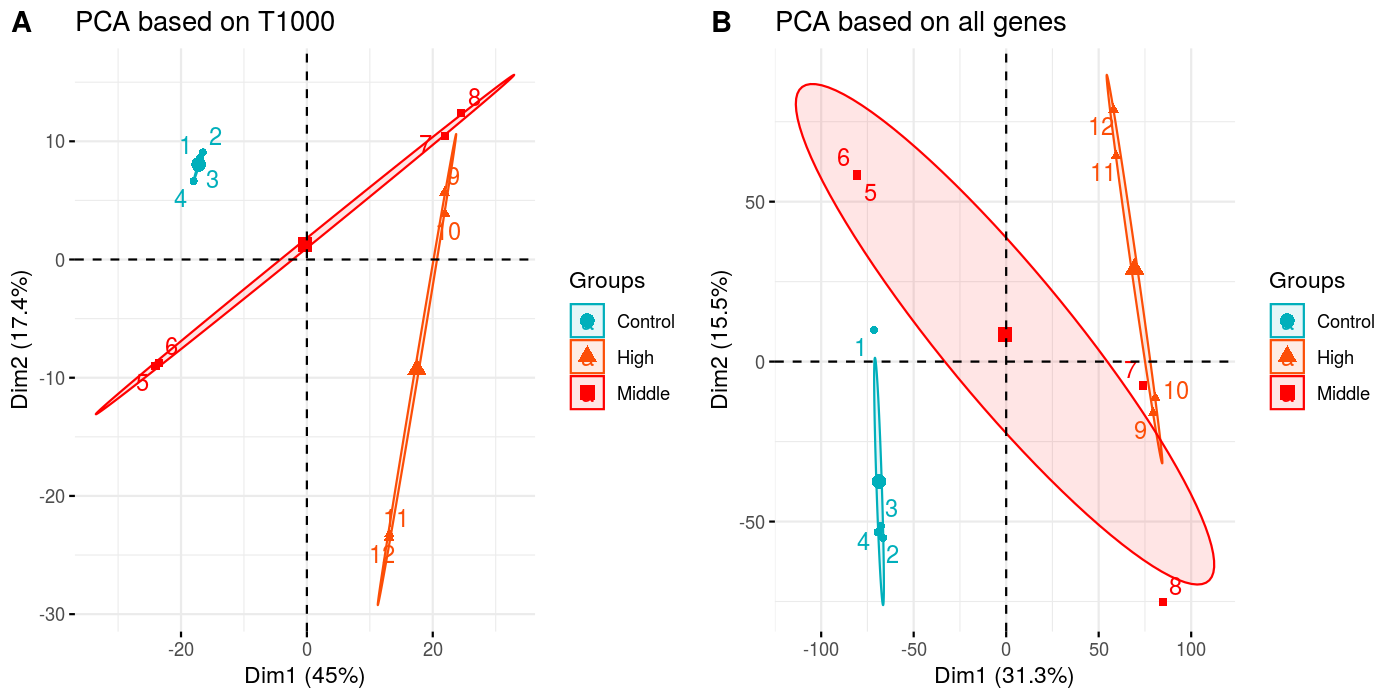

Supplement: Figure S5 [file peerj-07-7975-s005.zip › Supplementary_Figures_S5/iproniazid.Human.in_vitro.Liver.tiff]

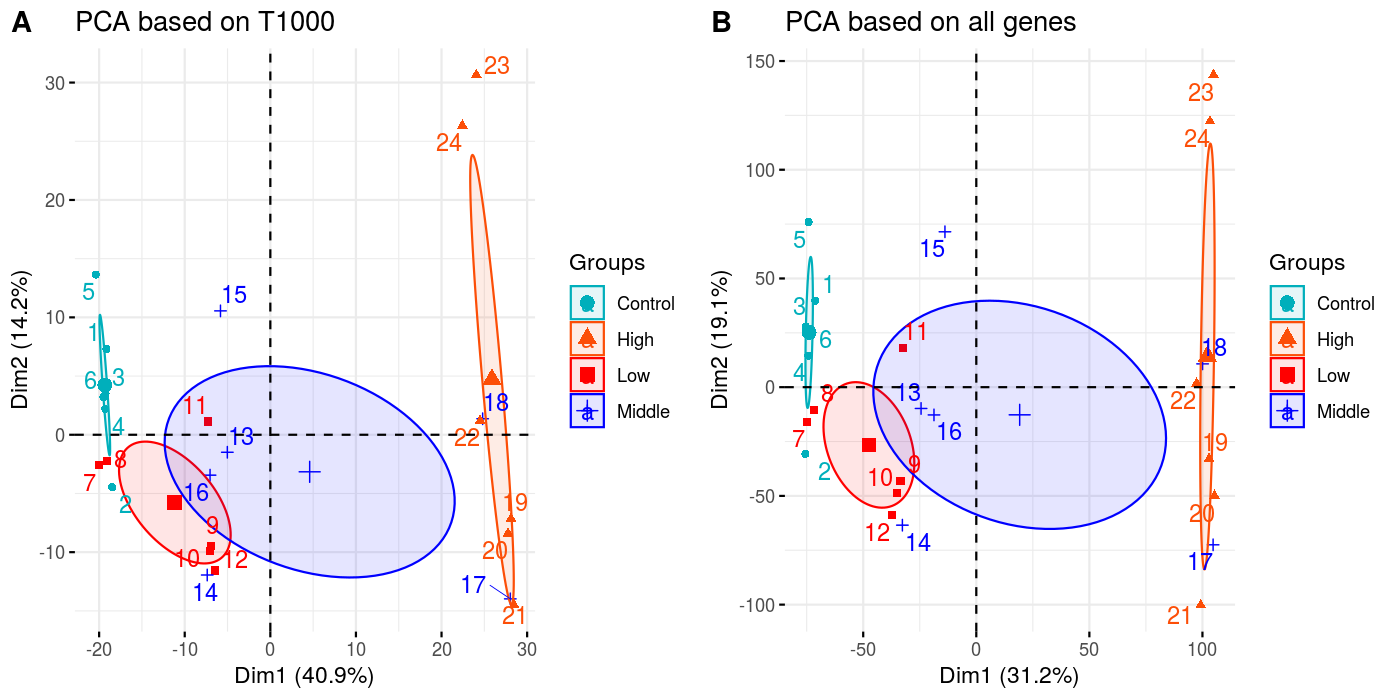

Supplement: Figure S5 [file peerj-07-7975-s005.zip › Supplementary_Figures_S5/thioridazine.Human.in_vitro.Liver.tiff]

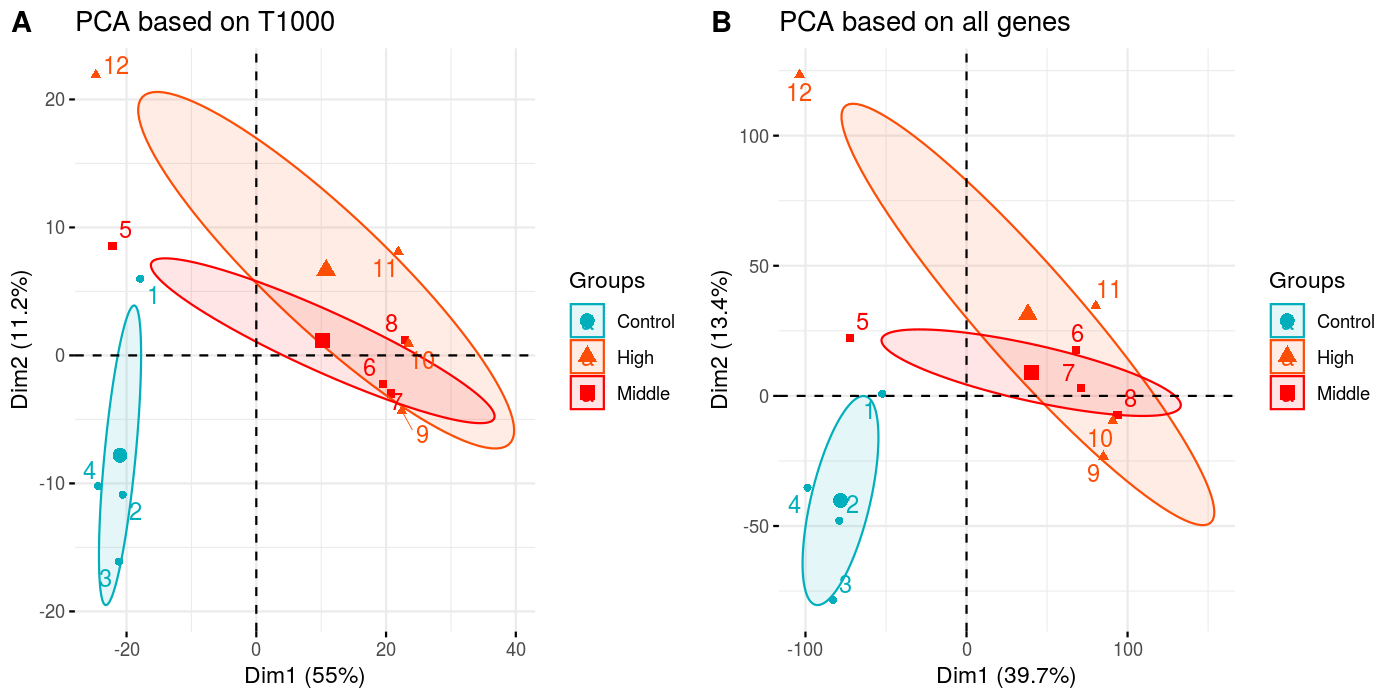

Supplement: Figure S5 [file peerj-07-7975-s005.zip › Supplementary_Figures_S5/erythromycin_ethylsuccinate.Human.in_vitro.Liver.tiff]

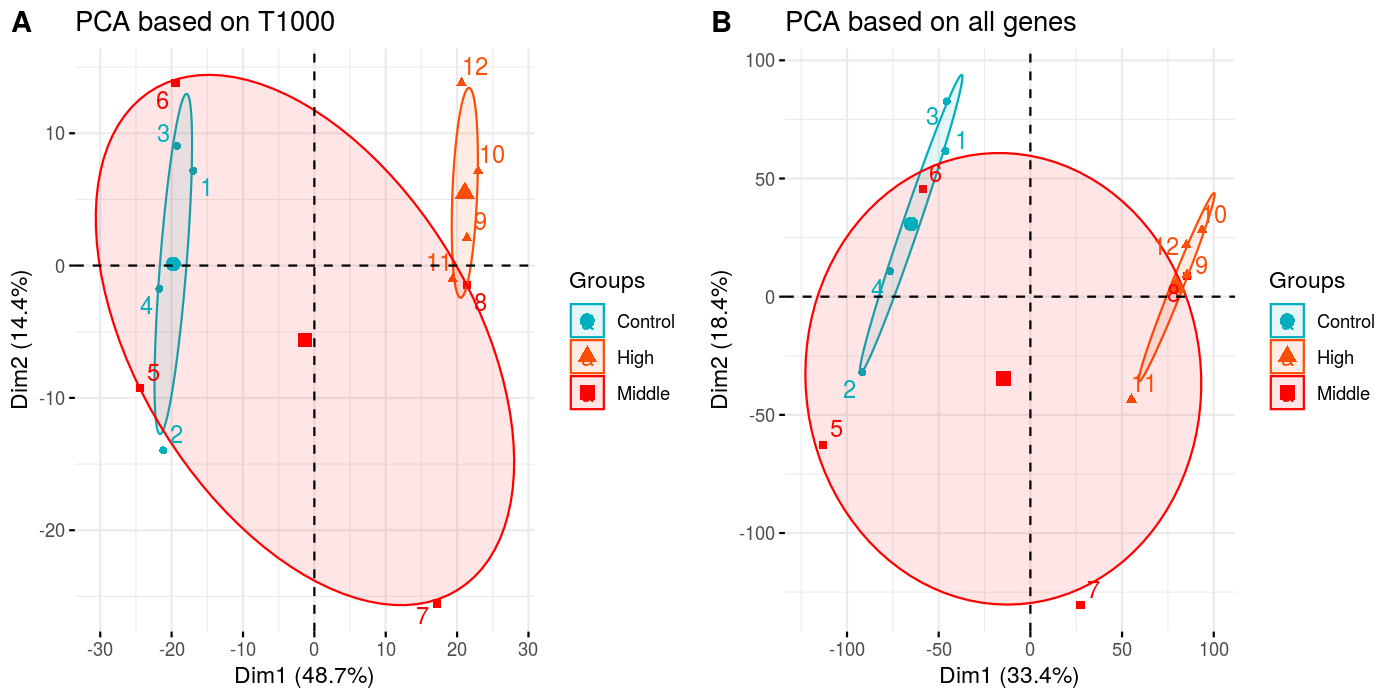

Supplement: Figure S5 [file peerj-07-7975-s005.zip › Supplementary_Figures_S5/chlorpropamide.Human.in_vitro.Liver.tiff]

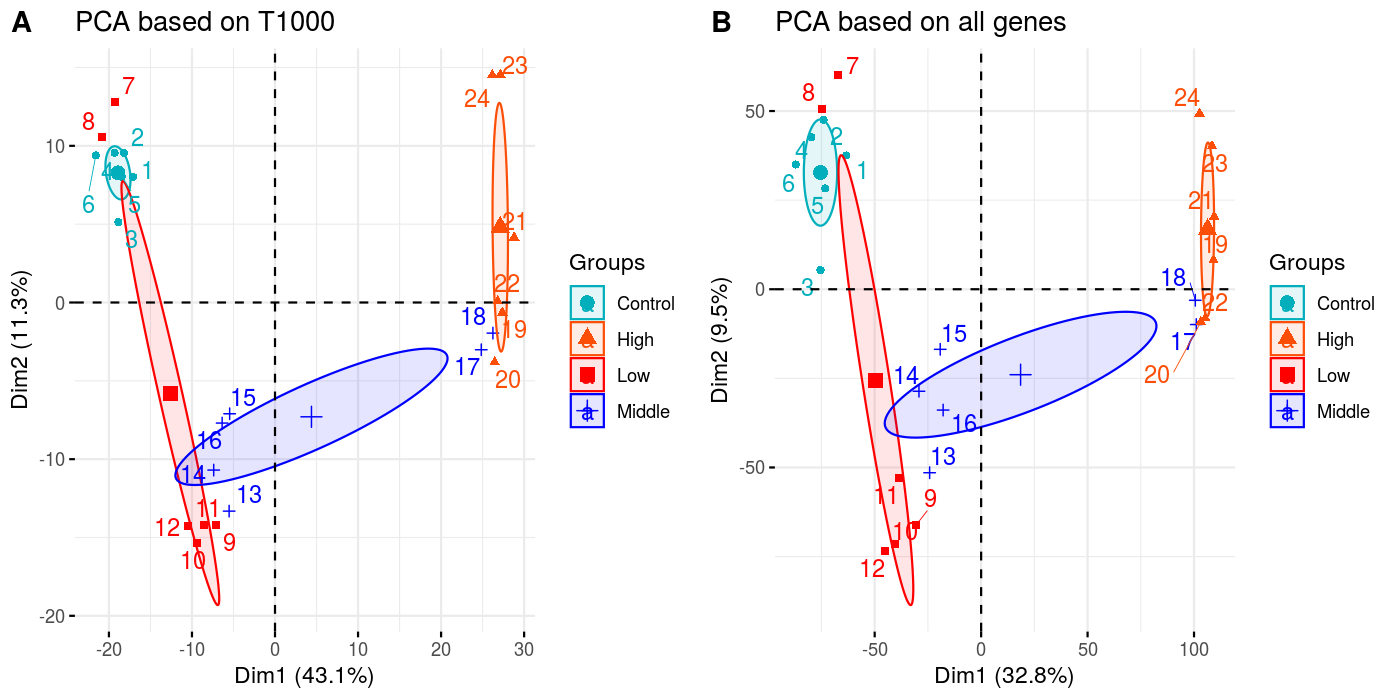

Supplement: Figure S5 [file peerj-07-7975-s005.zip › Supplementary_Figures_S5/fluphenazine.Human.in_vitro.Liver.tiff]

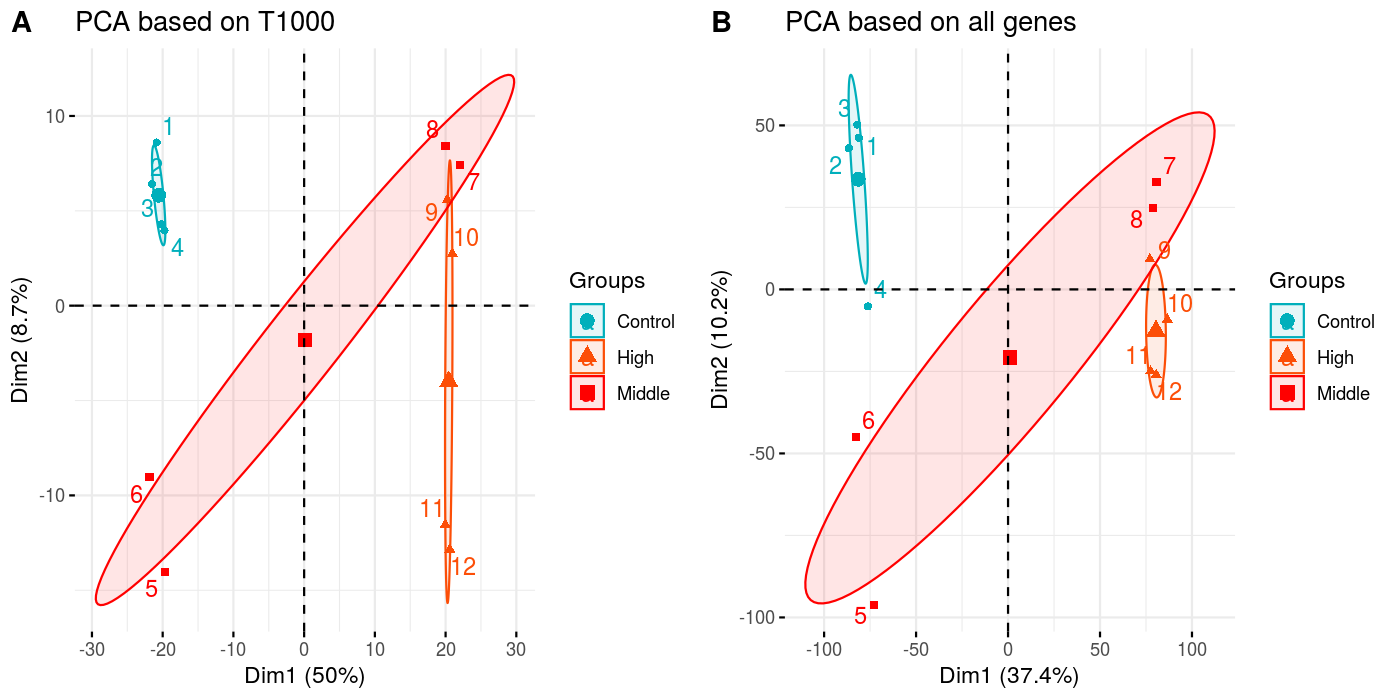

Supplement: Figure S5 [file peerj-07-7975-s005.zip › Supplementary_Figures_S5/phenacetin.Human.in_vitro.Liver.tiff]

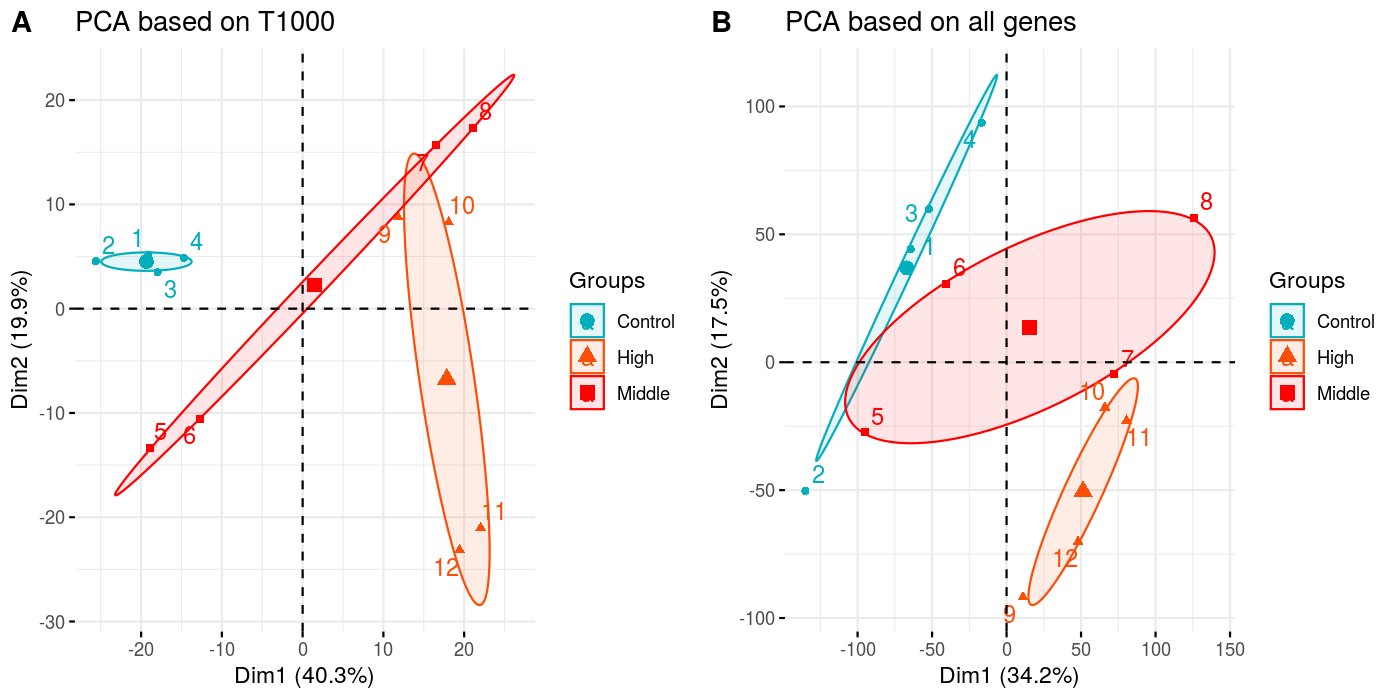

Supplement: Figure S5 [file peerj-07-7975-s005.zip › Supplementary_Figures_S5/ajmaline.Human.in_vitro.Liver.tiff]

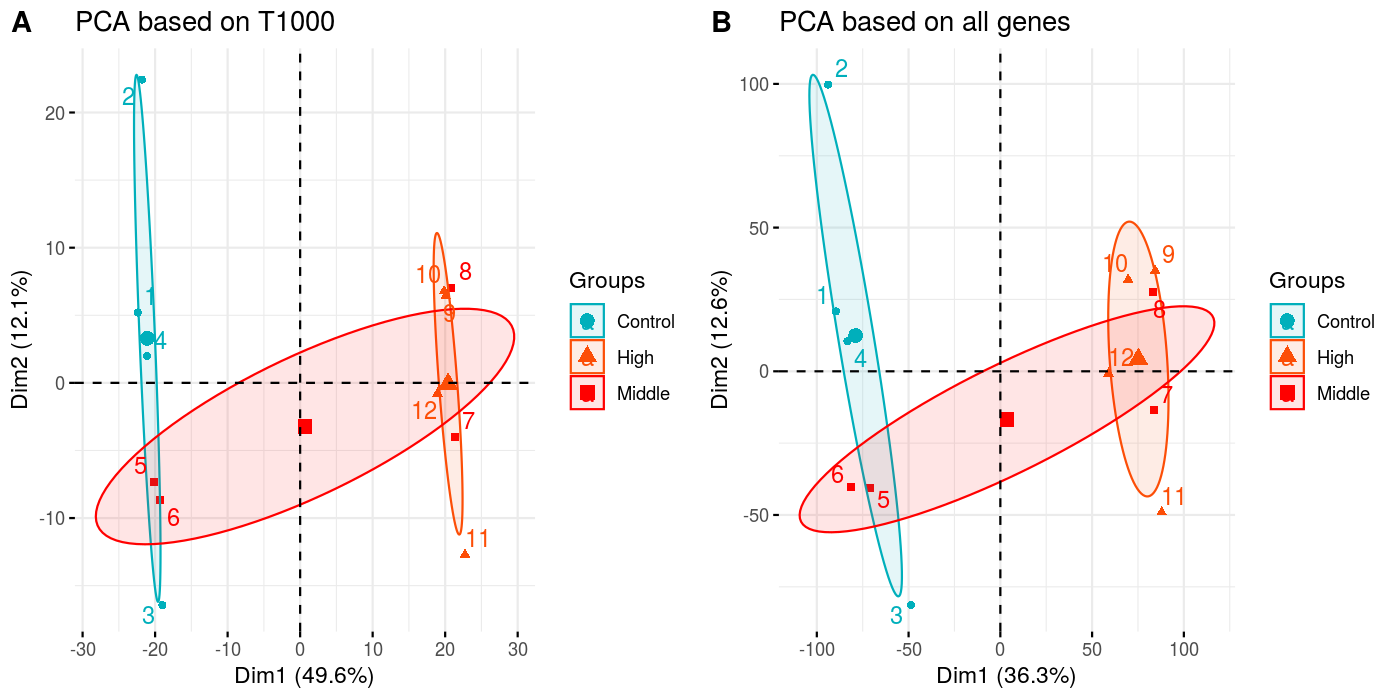

Supplement: Figure S5 [file peerj-07-7975-s005.zip › Supplementary_Figures_S5/pemoline.Human.in_vitro.Liver.tiff]

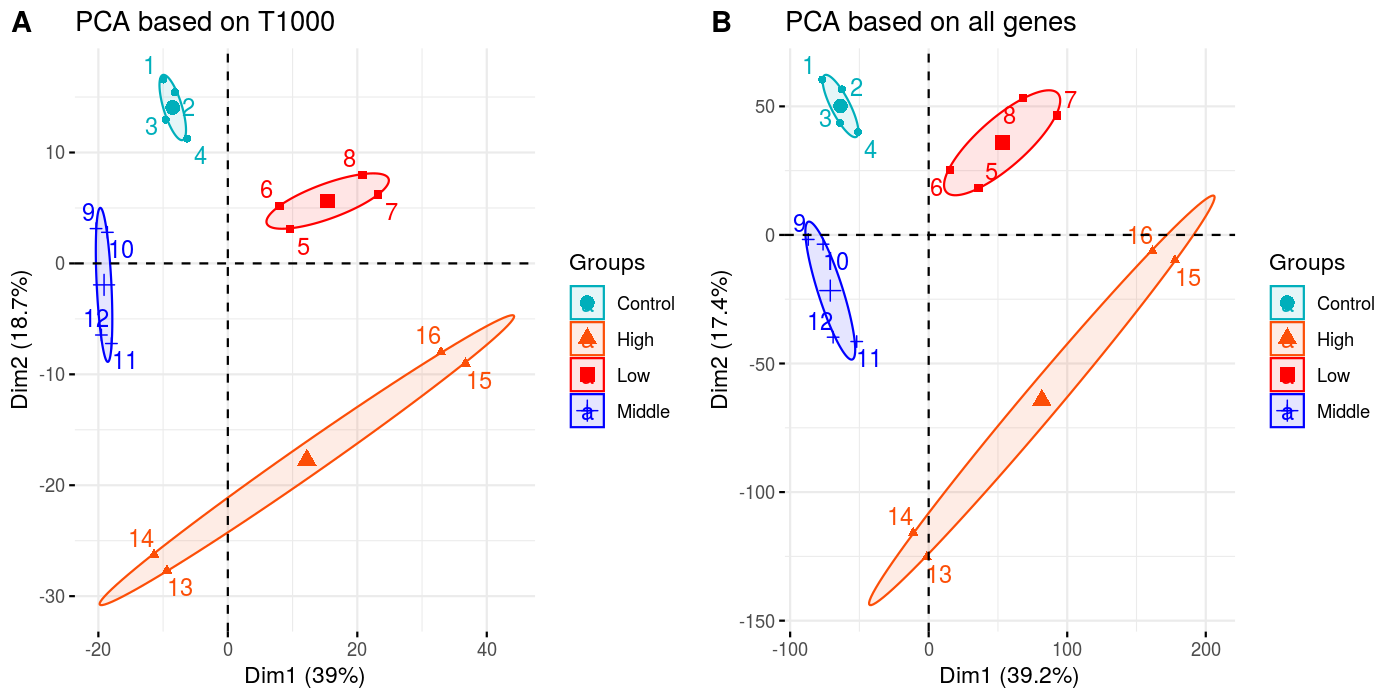

Supplement: Figure S5 [file peerj-07-7975-s005.zip › Supplementary_Figures_S5/doxorubicin.Human.in_vitro.Liver.tiff]

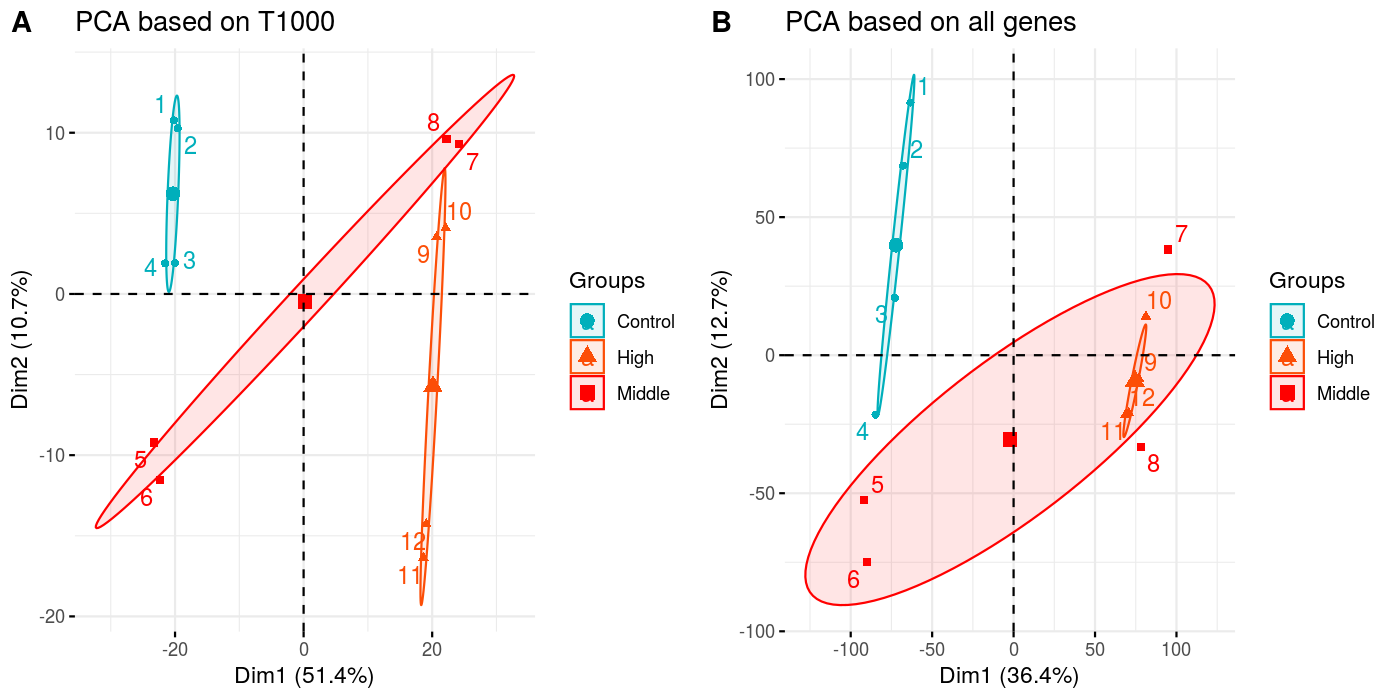

Supplement: Figure S5 [file peerj-07-7975-s005.zip › Supplementary_Figures_S5/chloramphenicol.Human.in_vitro.Liver.tiff]

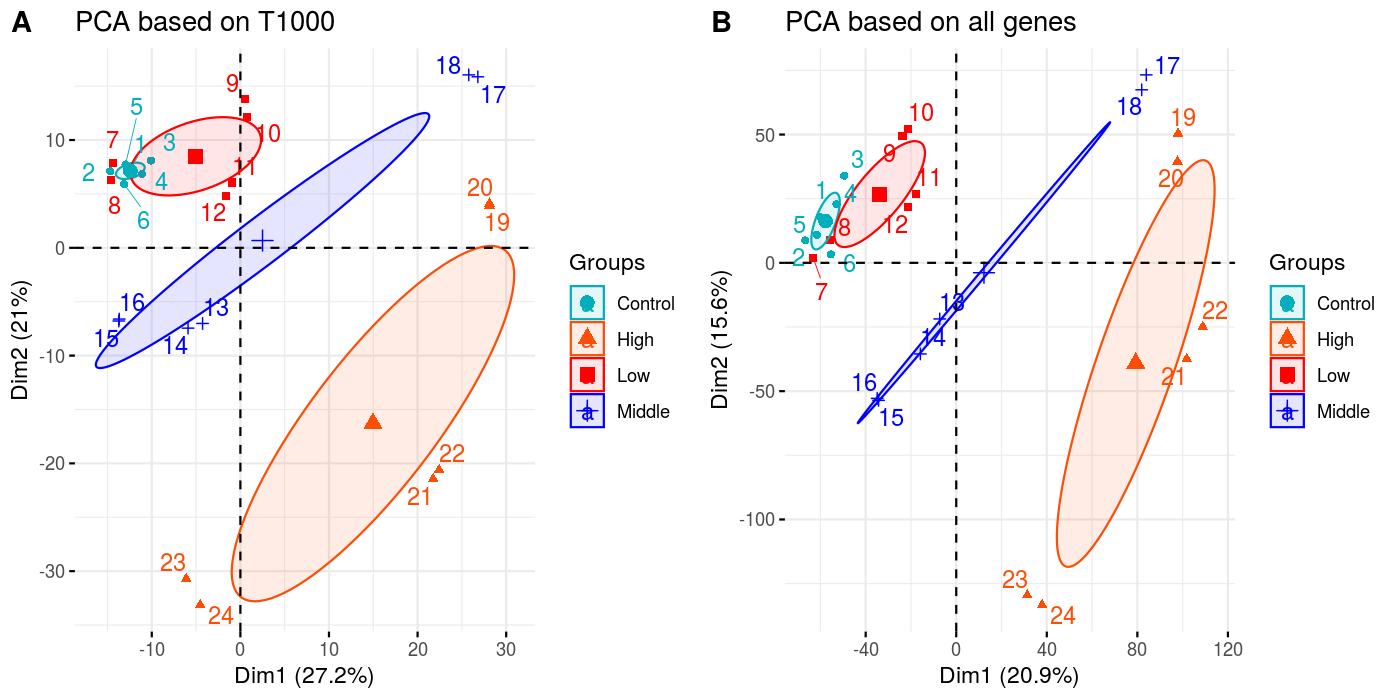

Supplement: Figure S5 [file peerj-07-7975-s005.zip › Supplementary_Figures_S5/propylthiouracil.Human.in_vitro.Liver.tiff]

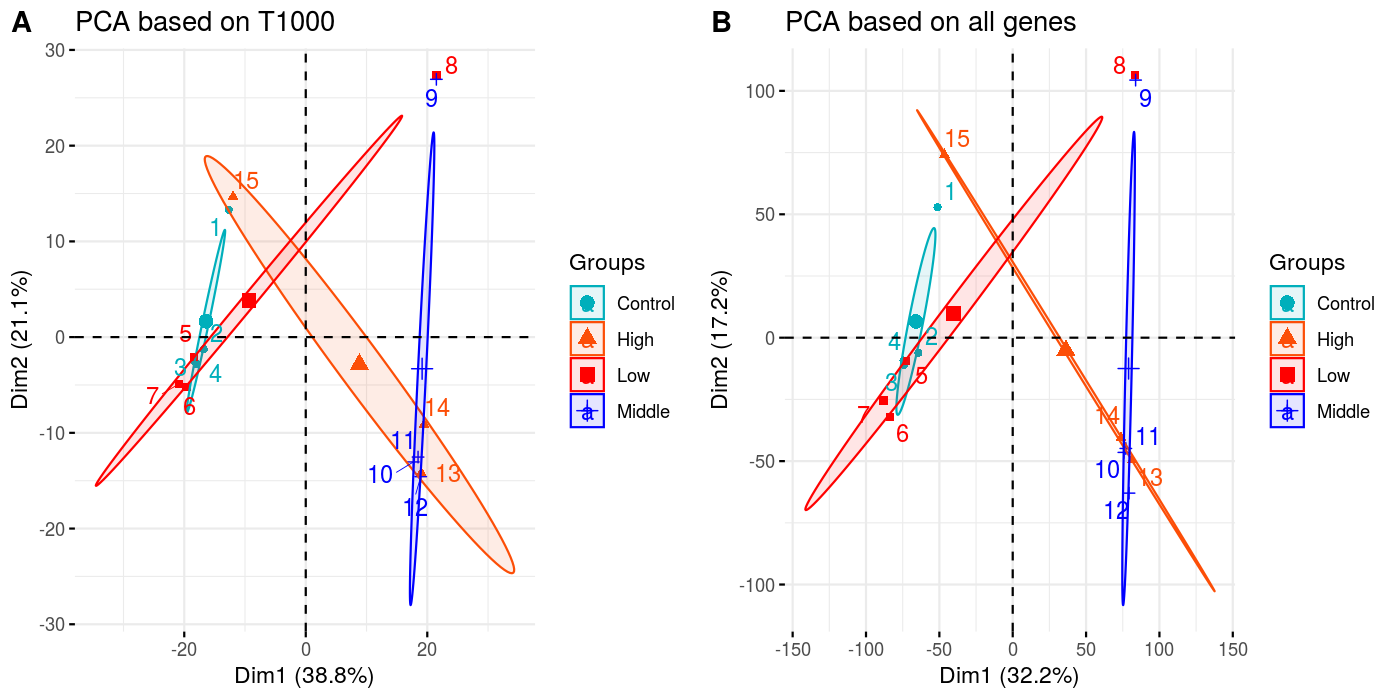

Supplement: Figure S5 [file peerj-07-7975-s005.zip › Supplementary_Figures_S5/LPS.Human.in_vitro.Liver.tiff]

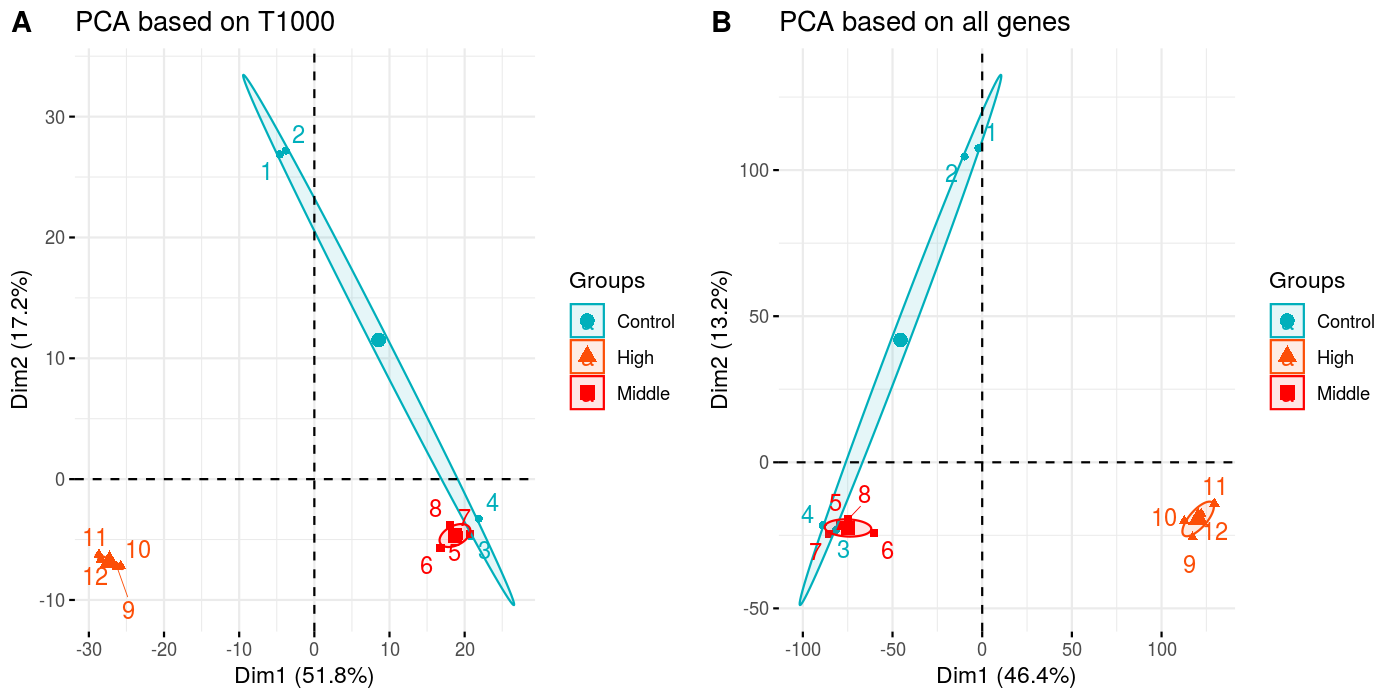

Supplement: Figure S5 [file peerj-07-7975-s005.zip › Supplementary_Figures_S5/dantrolene.Human.in_vitro.Liver.tiff]

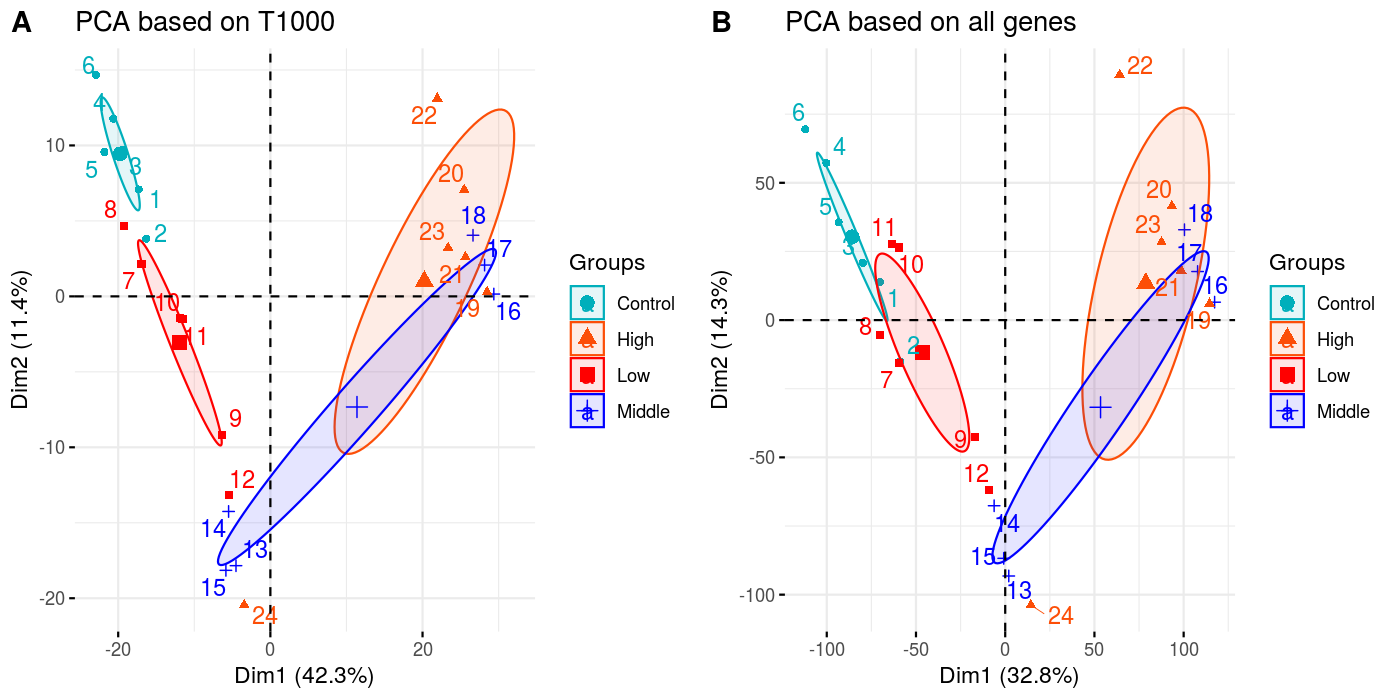

Supplement: Figure S5 [file peerj-07-7975-s005.zip › Supplementary_Figures_S5/griseofulvin.Human.in_vitro.Liver.tiff]

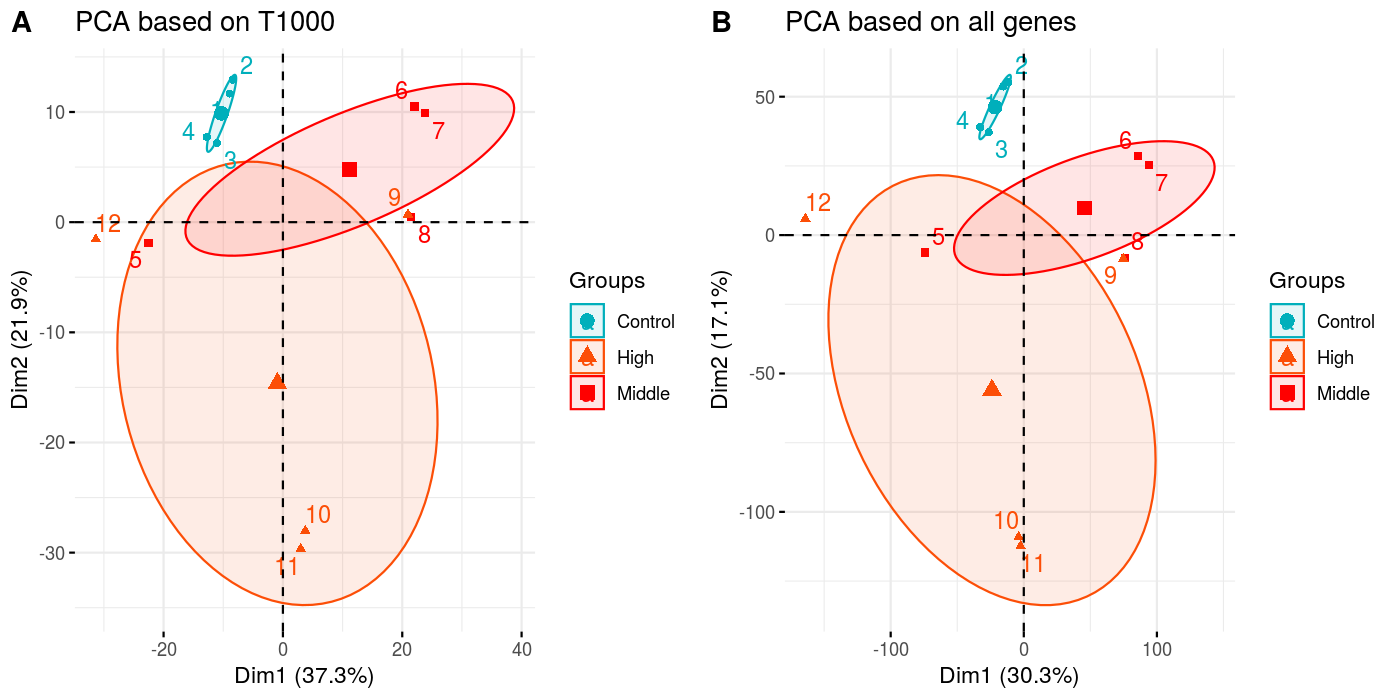

Supplement: Figure S5 [file peerj-07-7975-s005.zip › Supplementary_Figures_S5/chlormadinone.Human.in_vitro.Liver.tiff]

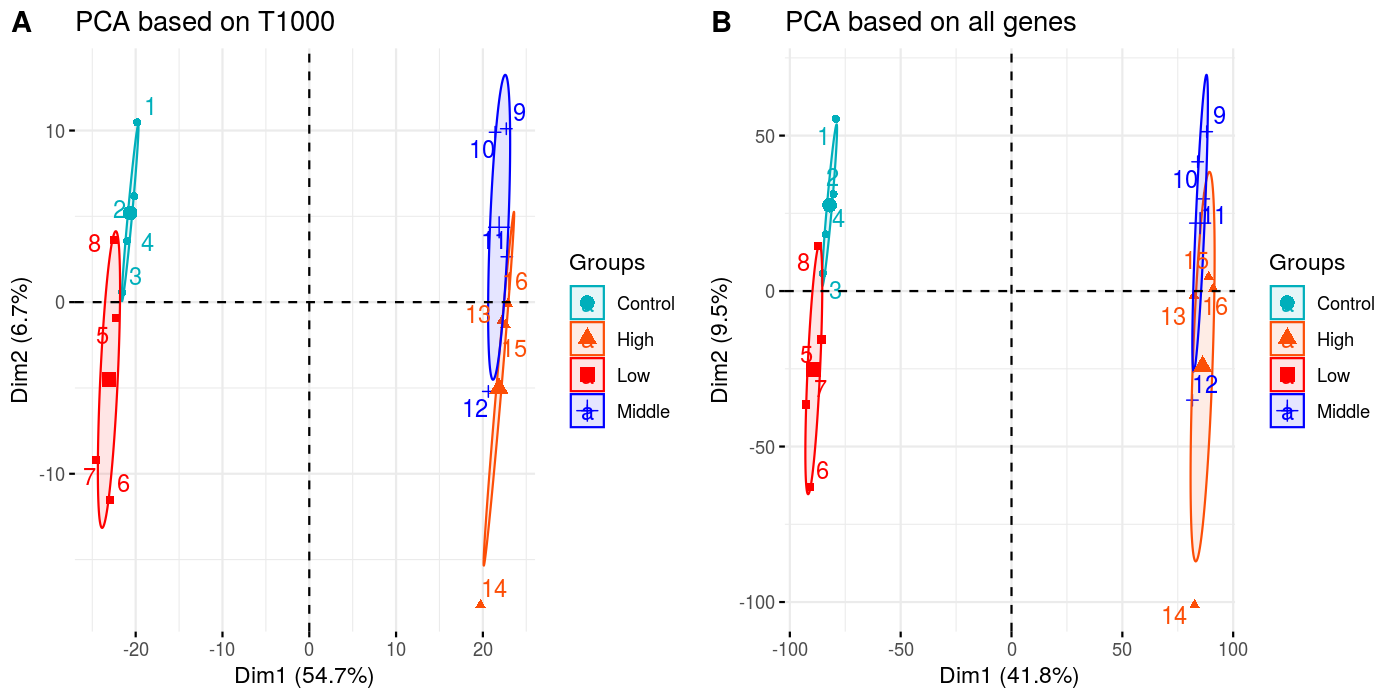

Supplement: Figure S5 [file peerj-07-7975-s005.zip › Supplementary_Figures_S5/acetamide.Human.in_vitro.Liver.tiff]

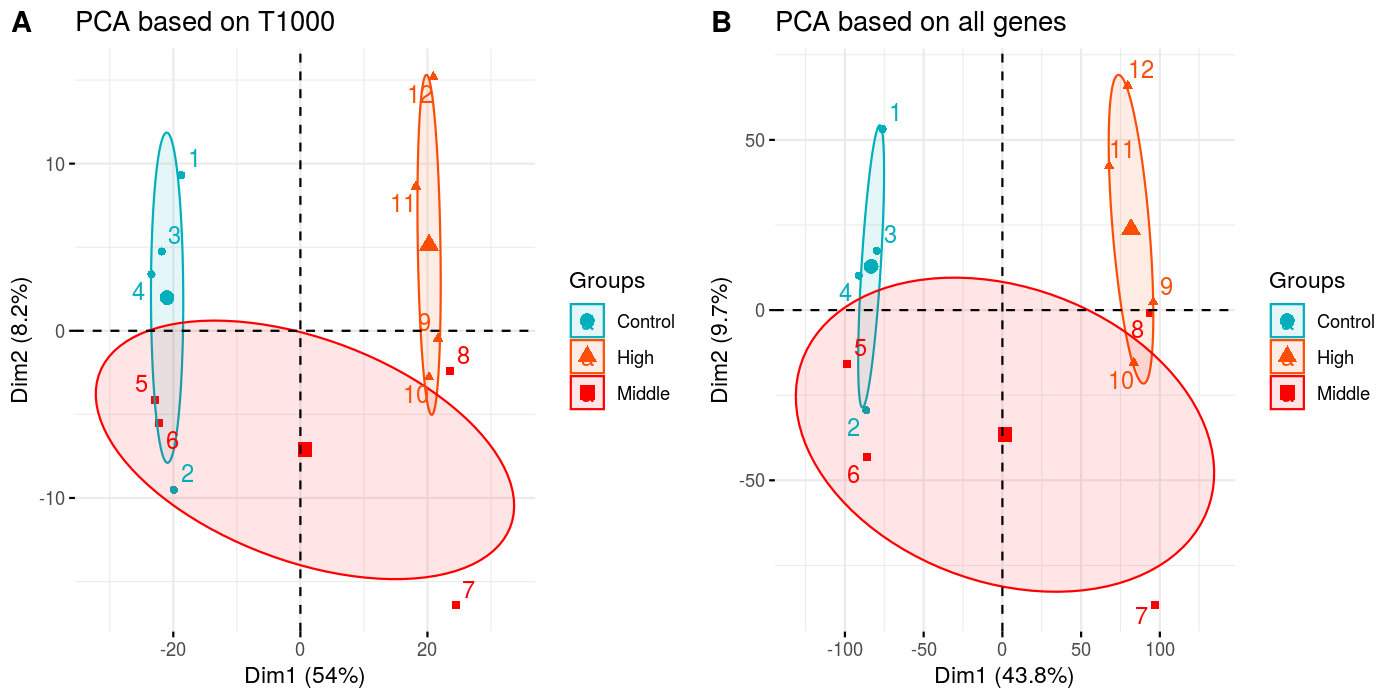

Supplement: Figure S5 [file peerj-07-7975-s005.zip › Supplementary_Figures_S5/clomipramine.Human.in_vitro.Liver.tiff]

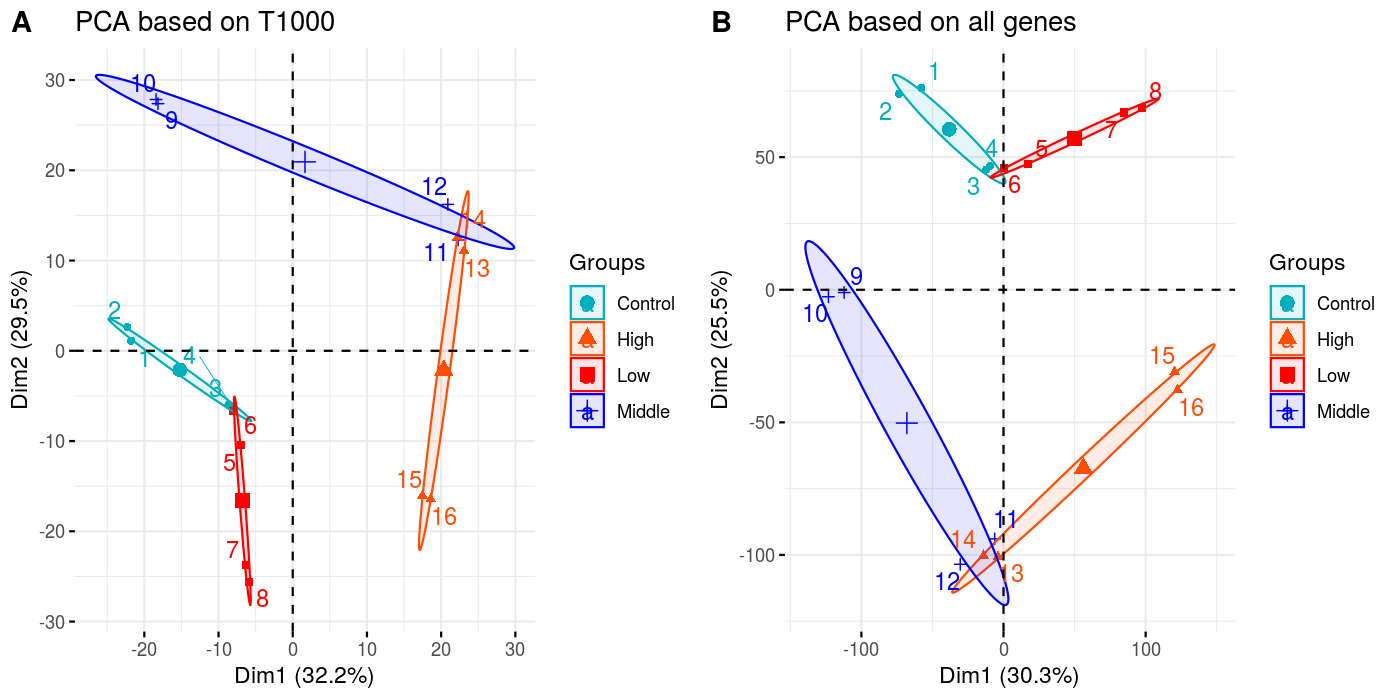

Supplement: Figure S5 [file peerj-07-7975-s005.zip › Supplementary_Figures_S5/tunicamycin.Human.in_vitro.Liver.tiff]

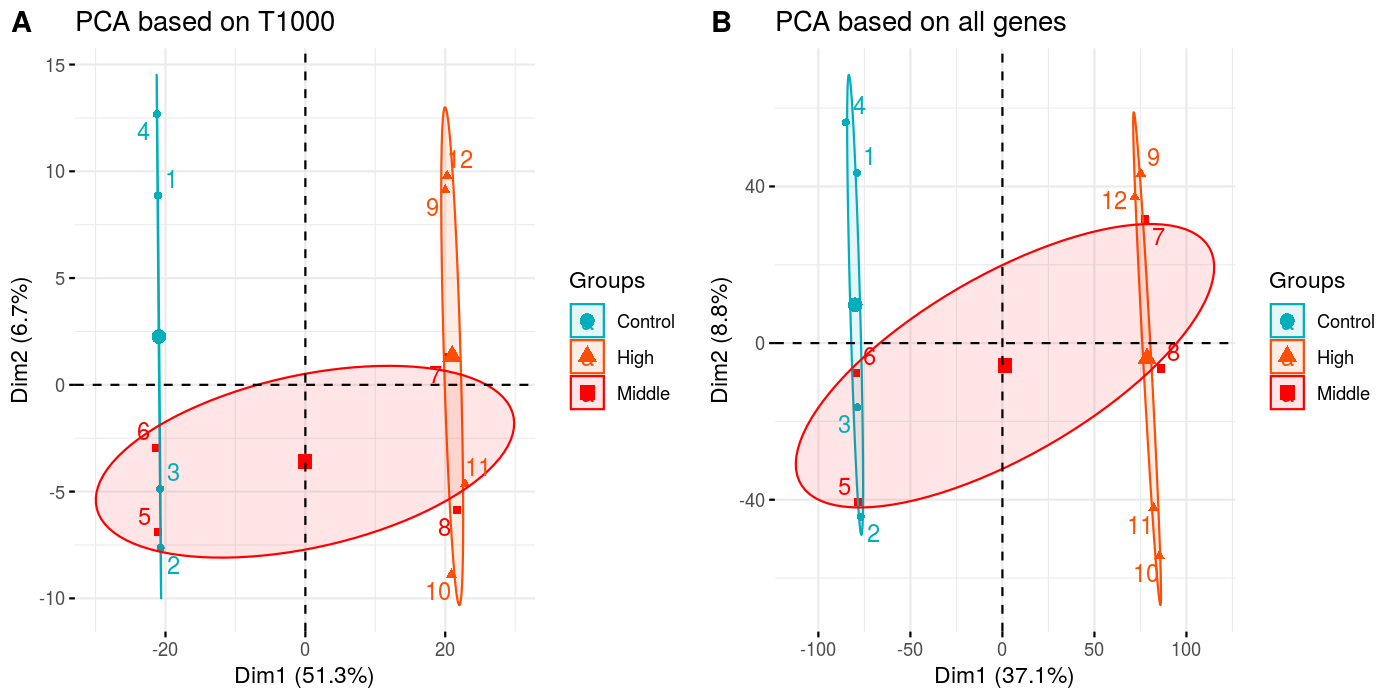

Supplement: Figure S5 [file peerj-07-7975-s005.zip › Supplementary_Figures_S5/acetazolamide.Human.in_vitro.Liver.tiff]

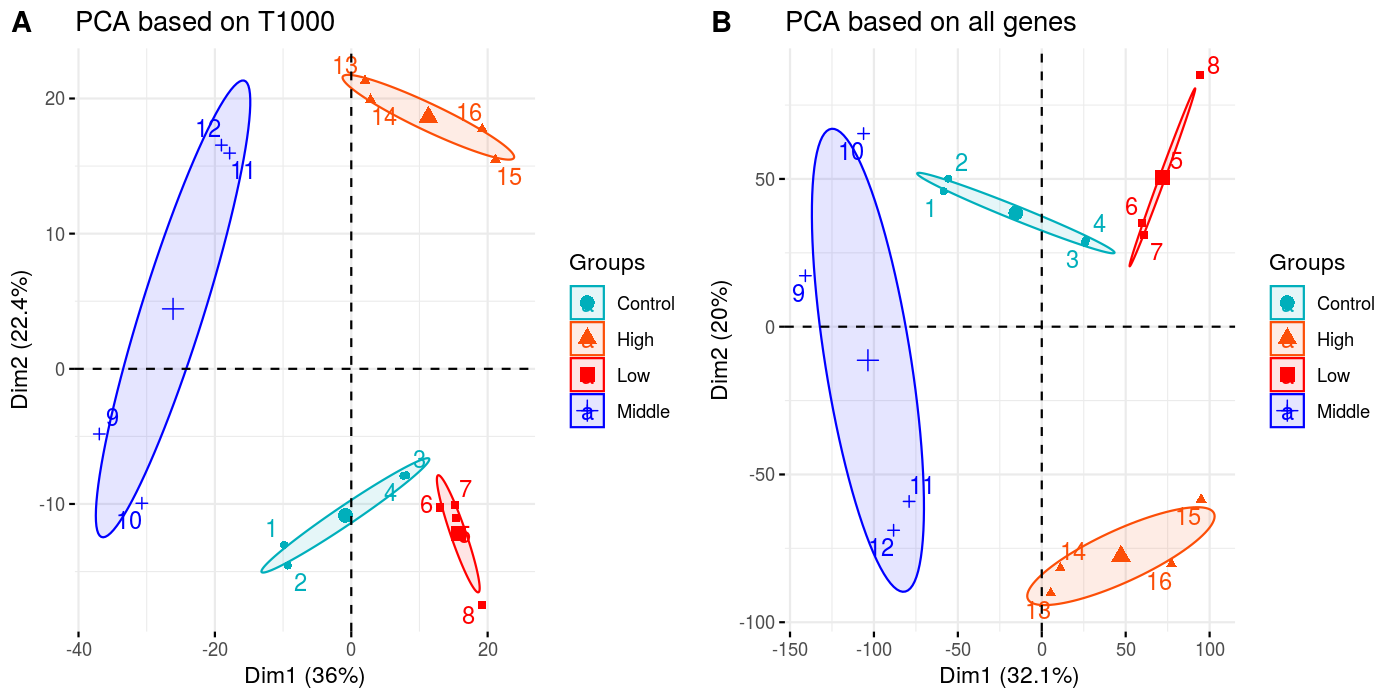

Supplement: Figure S5 [file peerj-07-7975-s005.zip › Supplementary_Figures_S5/cycloheximide.Human.in_vitro.Liver.tiff]

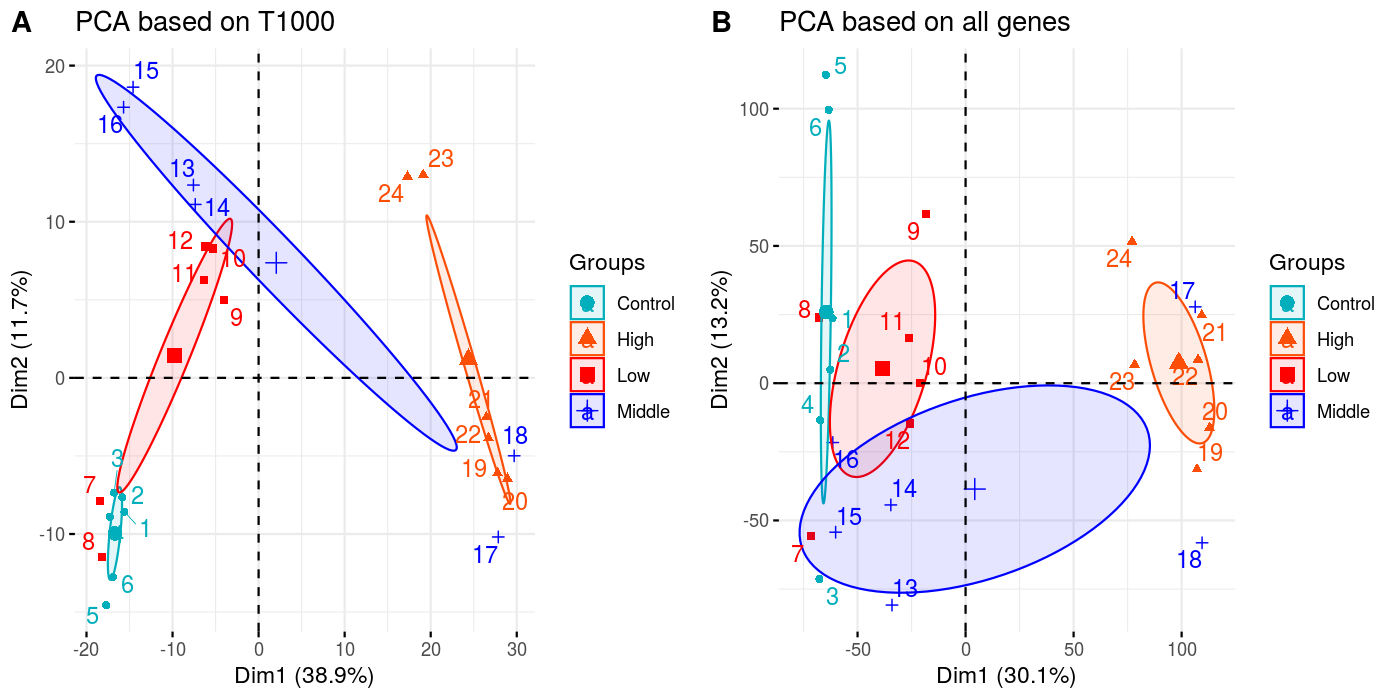

Supplement: Figure S5 [file peerj-07-7975-s005.zip › Supplementary_Figures_S5/lomustine.Human.in_vitro.Liver.tiff]

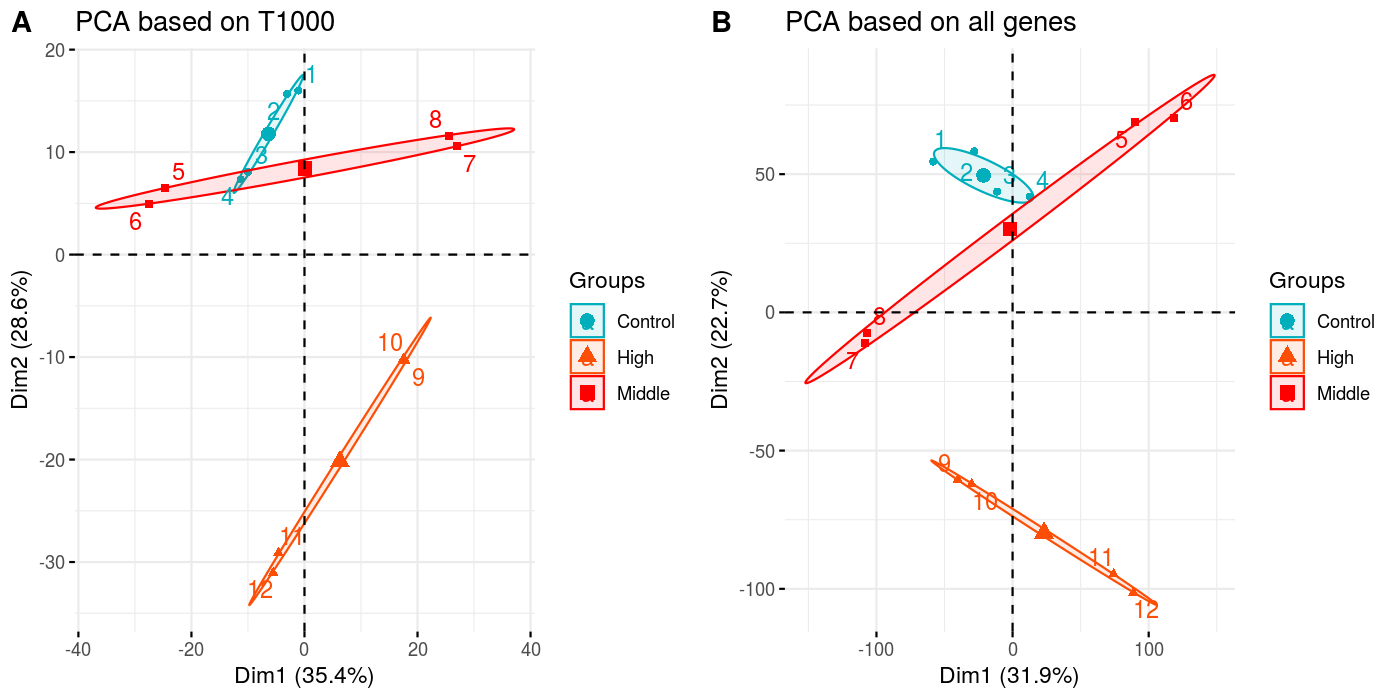

Supplement: Figure S5 [file peerj-07-7975-s005.zip › Supplementary_Figures_S5/theophylline.Human.in_vitro.Liver.tiff]

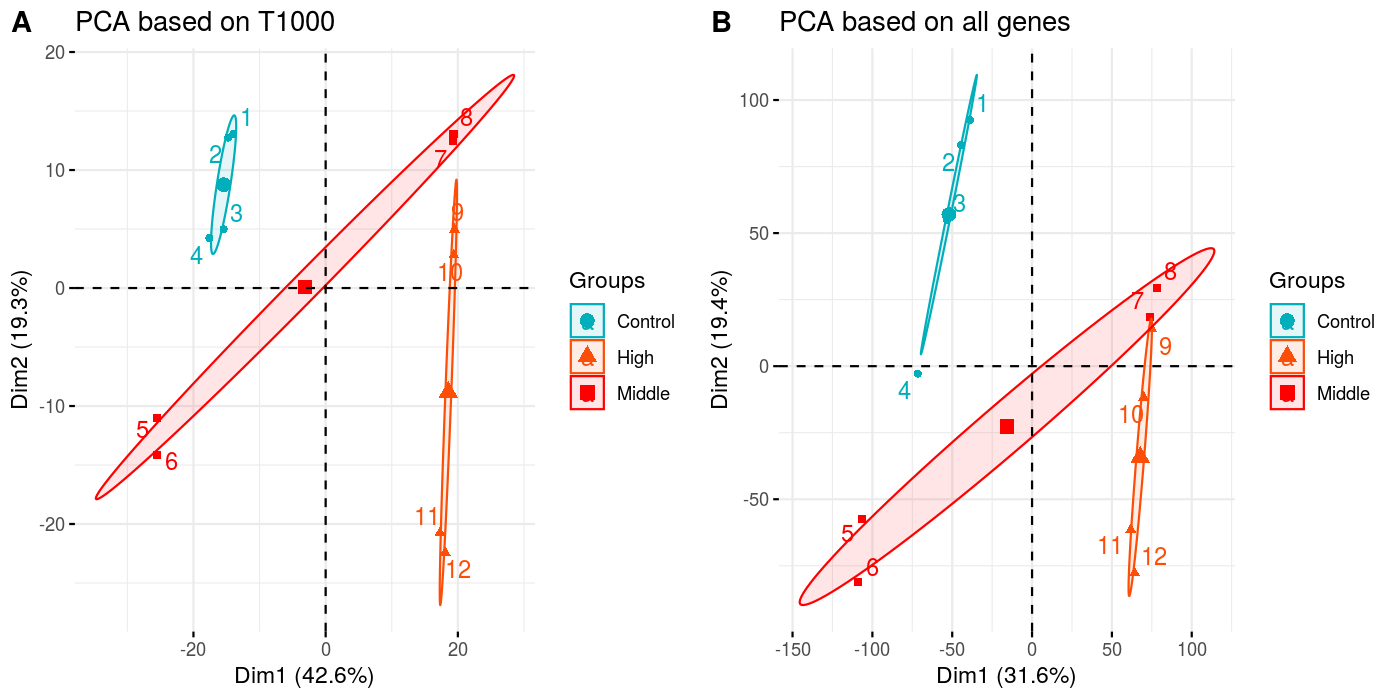

Supplement: Figure S5 [file peerj-07-7975-s005.zip › Supplementary_Figures_S5/methimazole.Human.in_vitro.Liver.tiff]

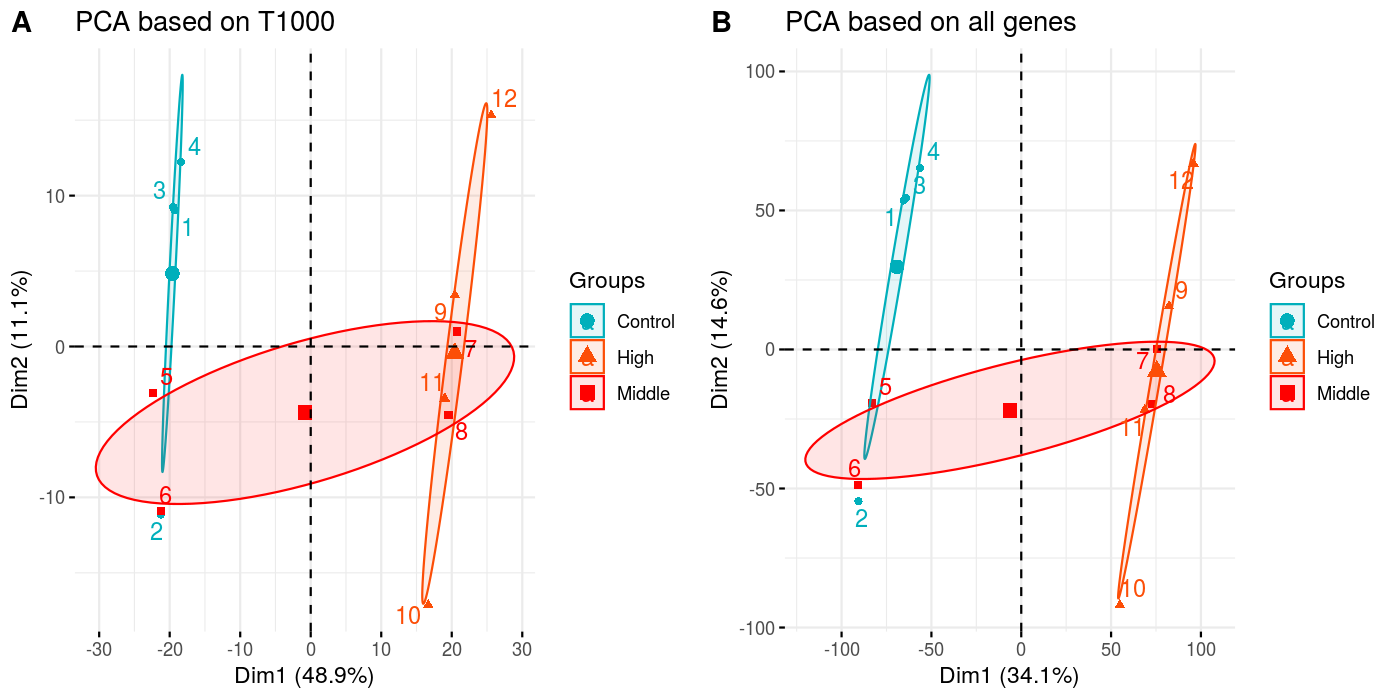

Supplement: Figure S5 [file peerj-07-7975-s005.zip › Supplementary_Figures_S5/monocrotaline.Human.in_vitro.Liver.tiff]

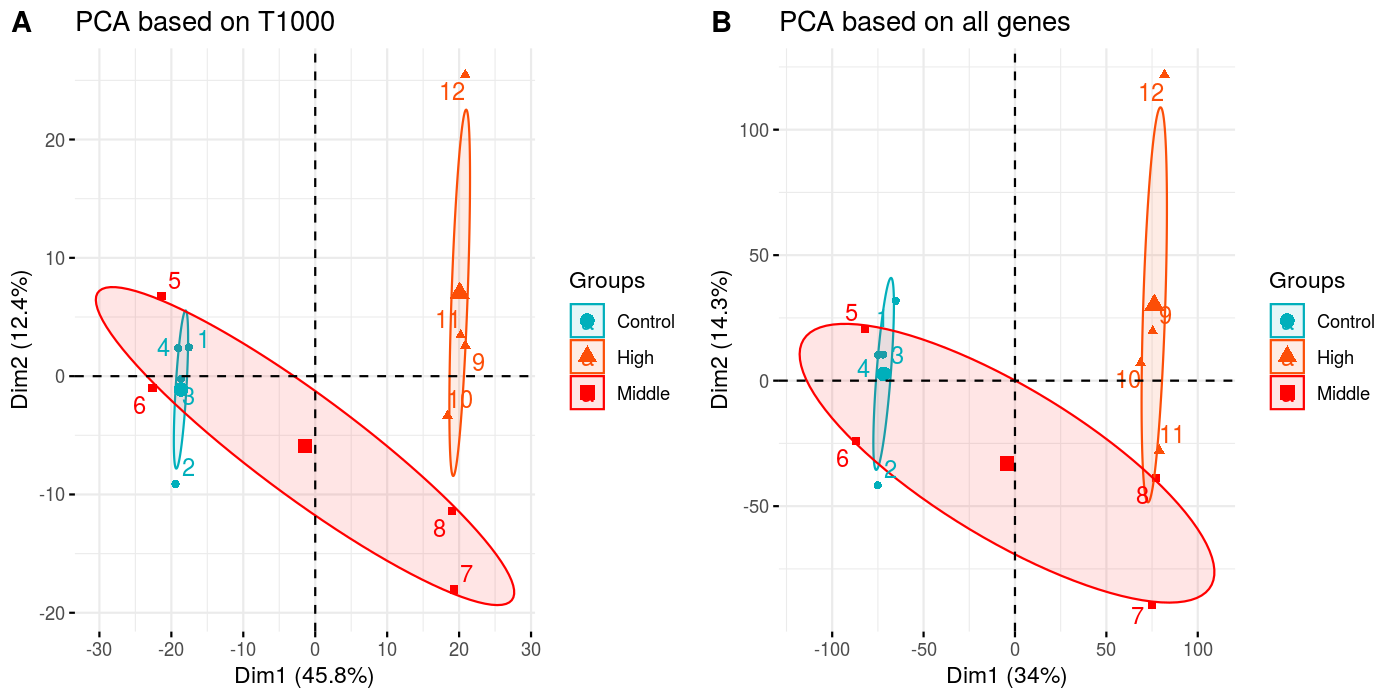

Supplement: Figure S5 [file peerj-07-7975-s005.zip › Supplementary_Figures_S5/nitrosodiethylamine.Human.in_vitro.Liver.tiff]

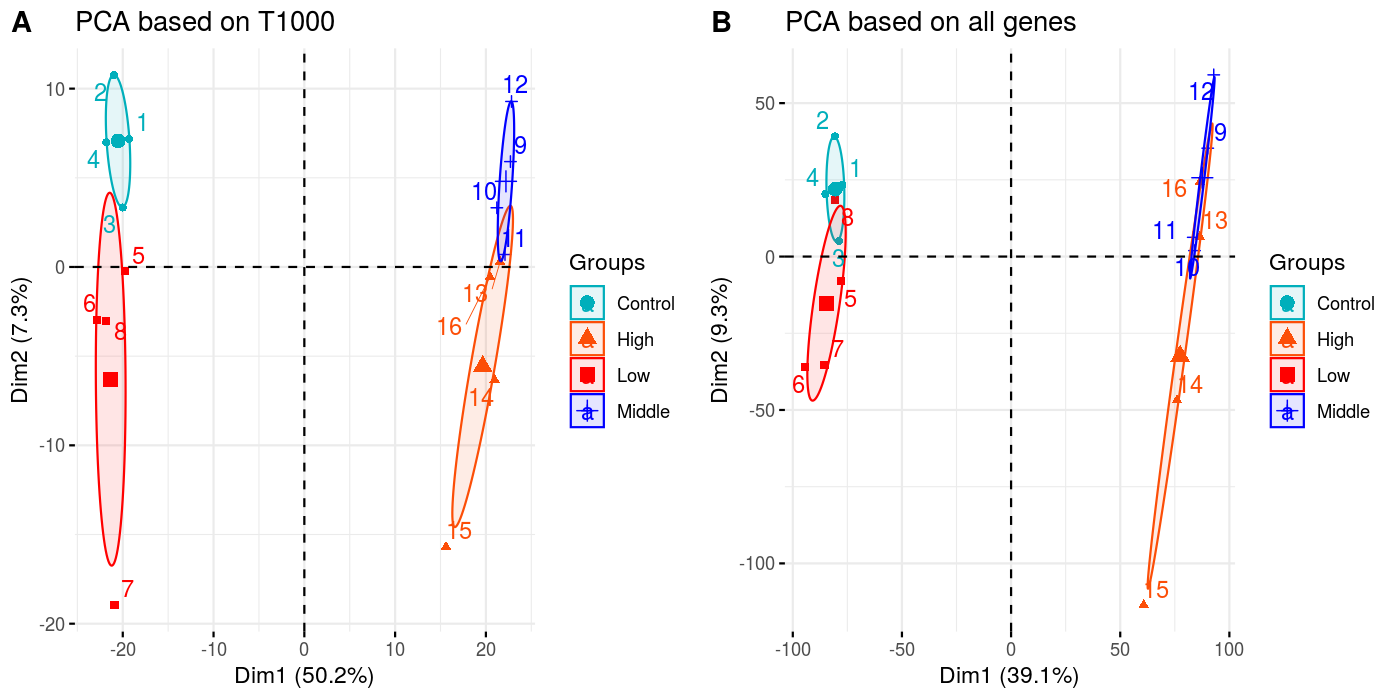

Supplement: Figure S5 [file peerj-07-7975-s005.zip › Supplementary_Figures_S5/rotenone.Human.in_vitro.Liver.tiff]

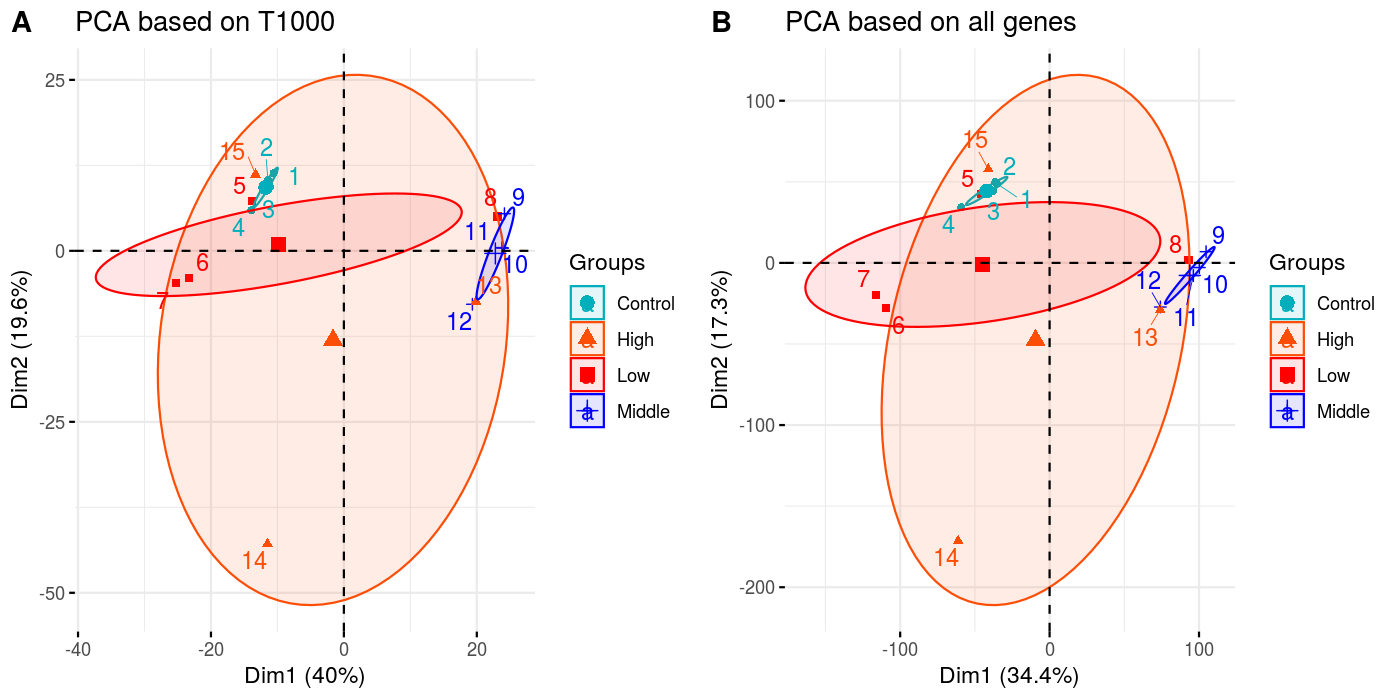

Supplement: Figure S5 [file peerj-07-7975-s005.zip › Supplementary_Figures_S5/bromoethylamine.Human.in_vitro.Liver.tiff]

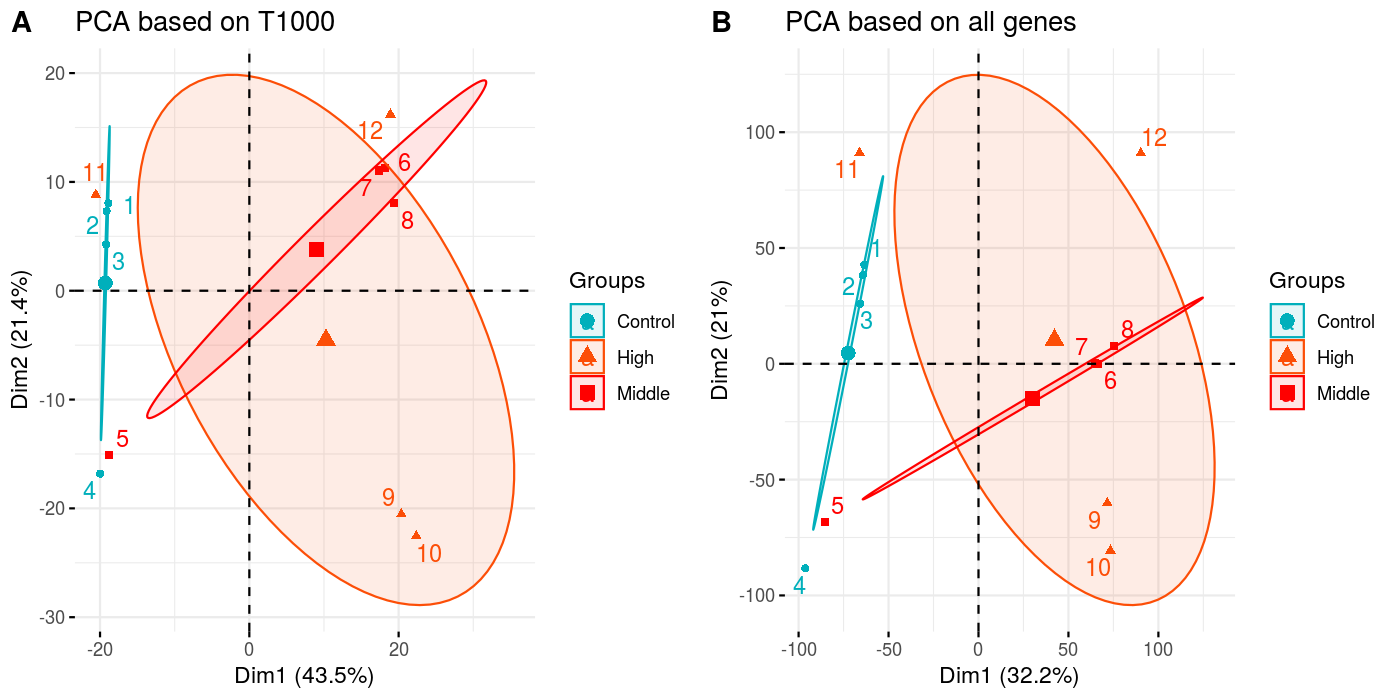

Supplement: Figure S5 [file peerj-07-7975-s005.zip › Supplementary_Figures_S5/ethambutol.Human.in_vitro.Liver.tiff]

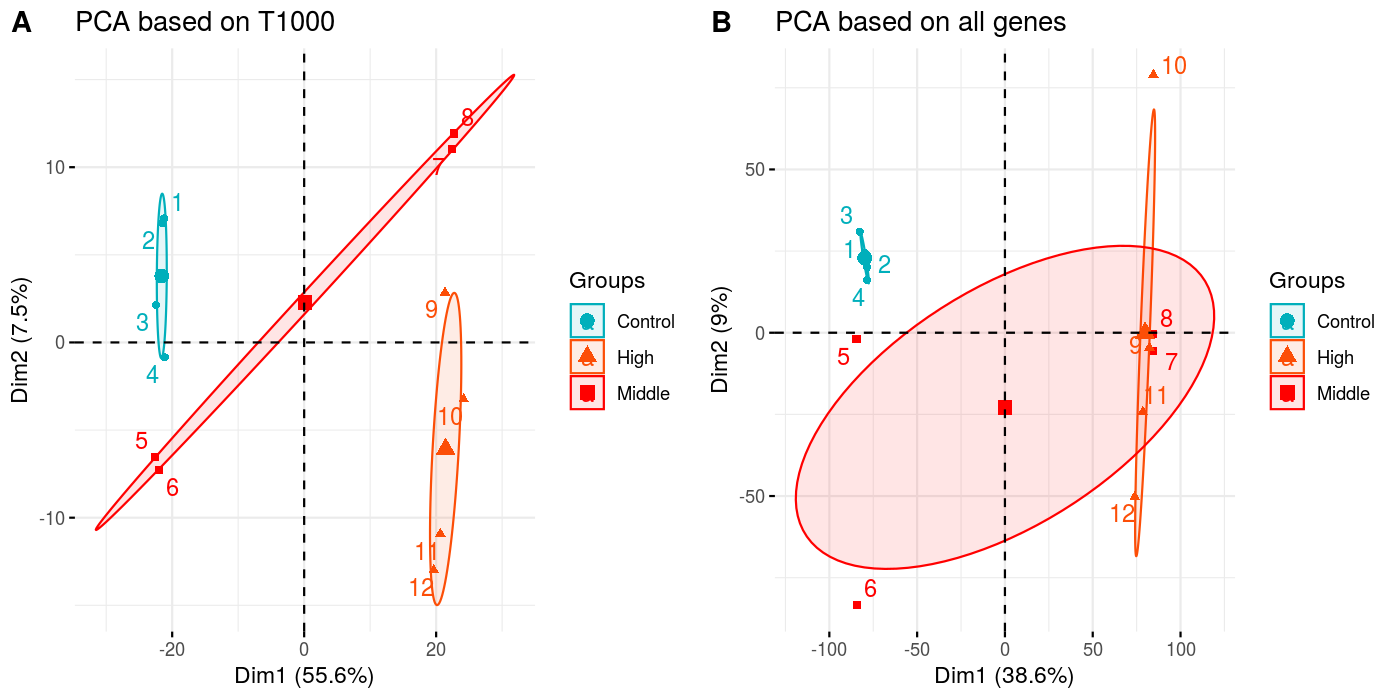

Supplement: Figure S5 [file peerj-07-7975-s005.zip › Supplementary_Figures_S5/famotidine.Human.in_vitro.Liver.tiff]

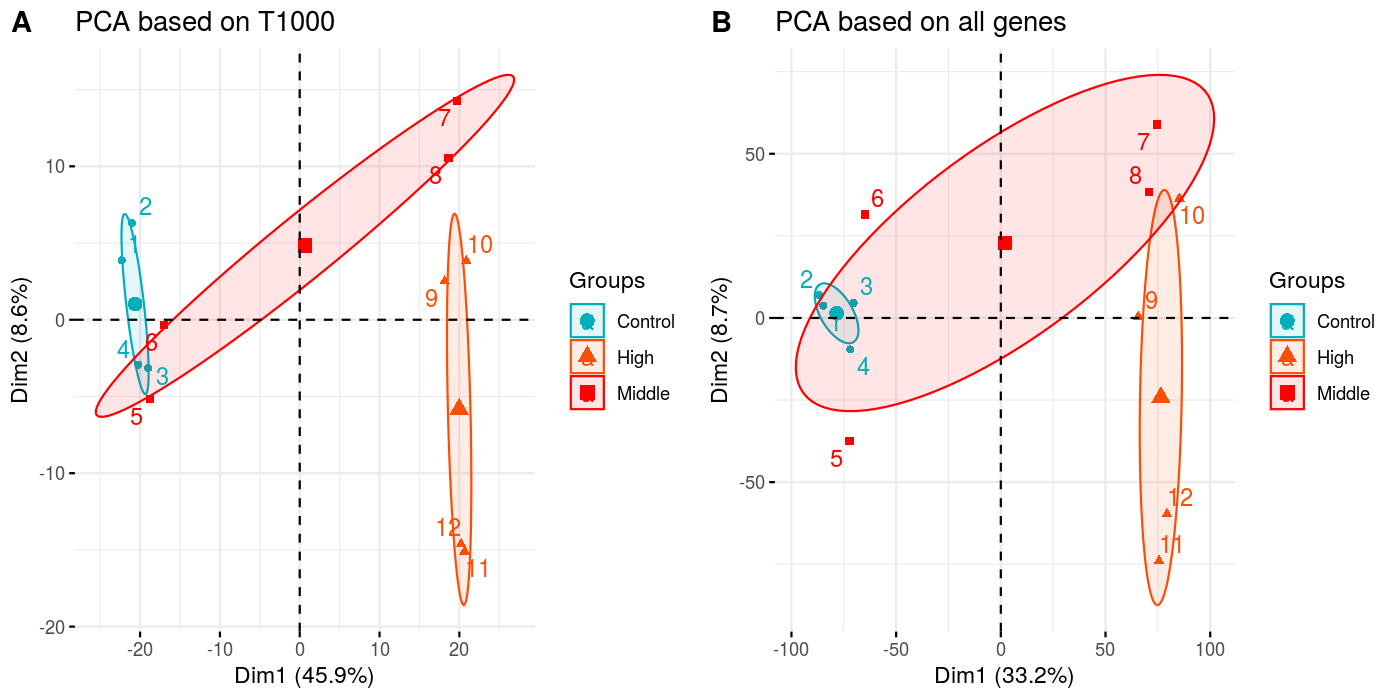

Supplement: Figure S5 [file peerj-07-7975-s005.zip › Supplementary_Figures_S5/ethanol.Human.in_vitro.Liver.tiff]

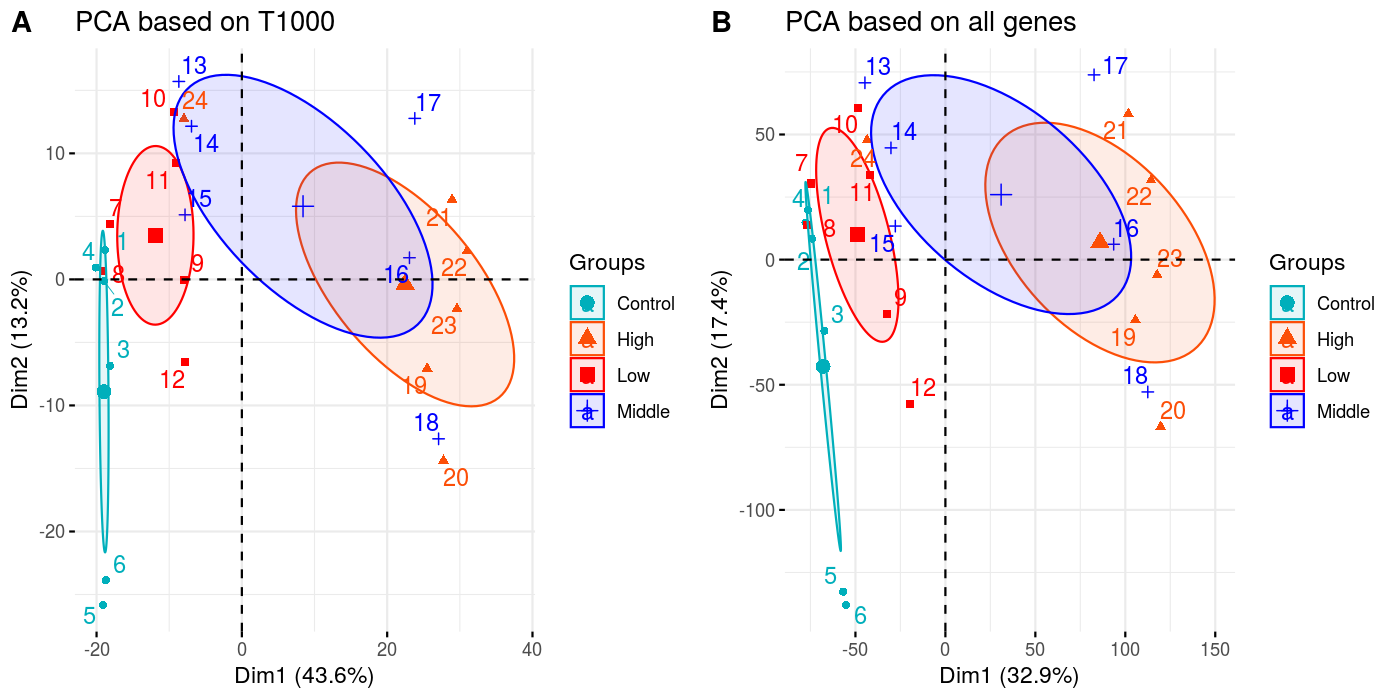

Supplement: Figure S5 [file peerj-07-7975-s005.zip › Supplementary_Figures_S5/thioacetamide.Human.in_vitro.Liver.tiff]

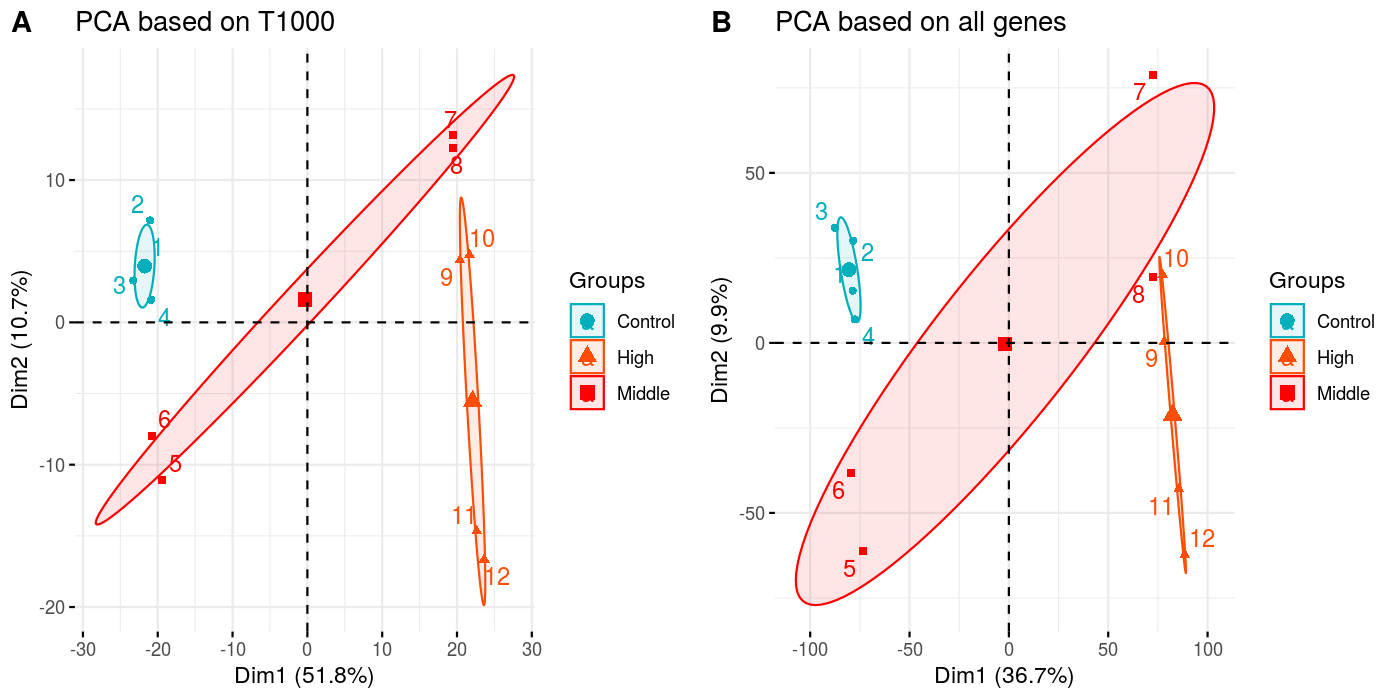

Supplement: Figure S5 [file peerj-07-7975-s005.zip › Supplementary_Figures_S5/chlorpheniramine.Human.in_vitro.Liver.tiff]

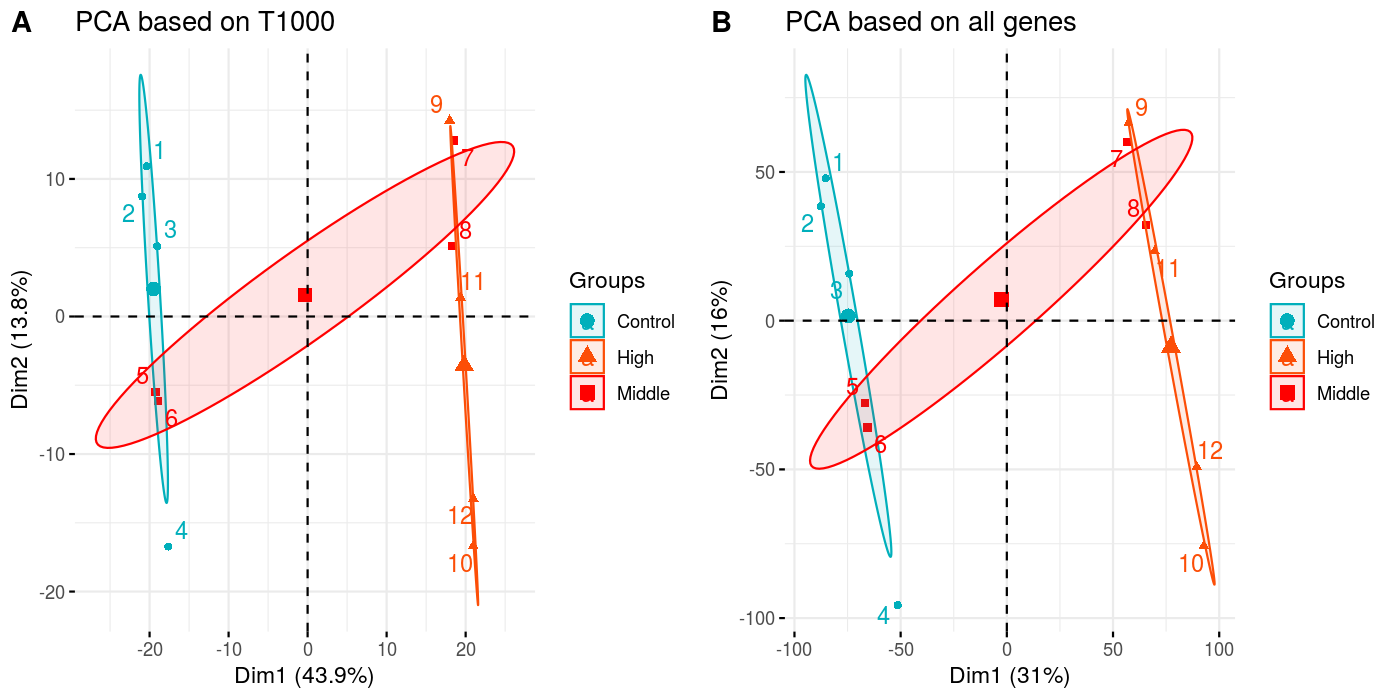

Supplement: Figure S5 [file peerj-07-7975-s005.zip › Supplementary_Figures_S5/bucetin.Human.in_vitro.Liver.tiff]

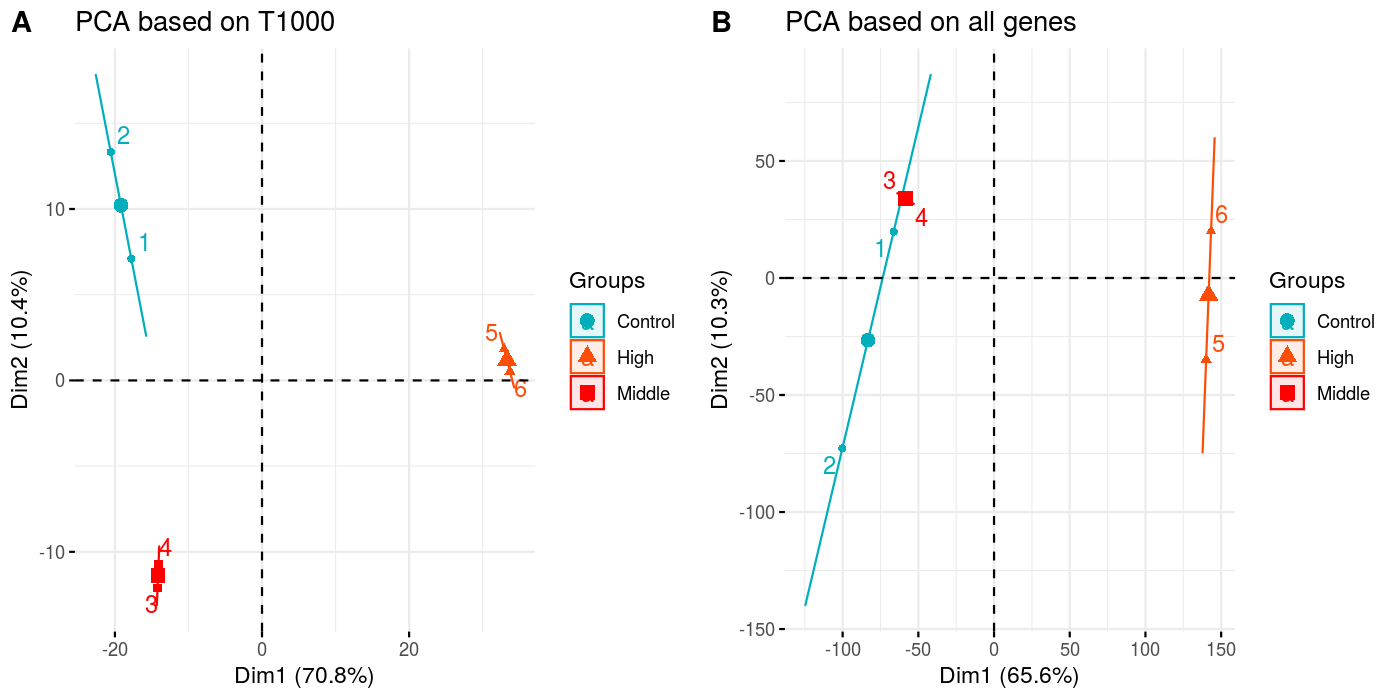

Supplement: Figure S5 [file peerj-07-7975-s005.zip › Supplementary_Figures_S5/nefazodone.Human.in_vitro.Liver.tiff]

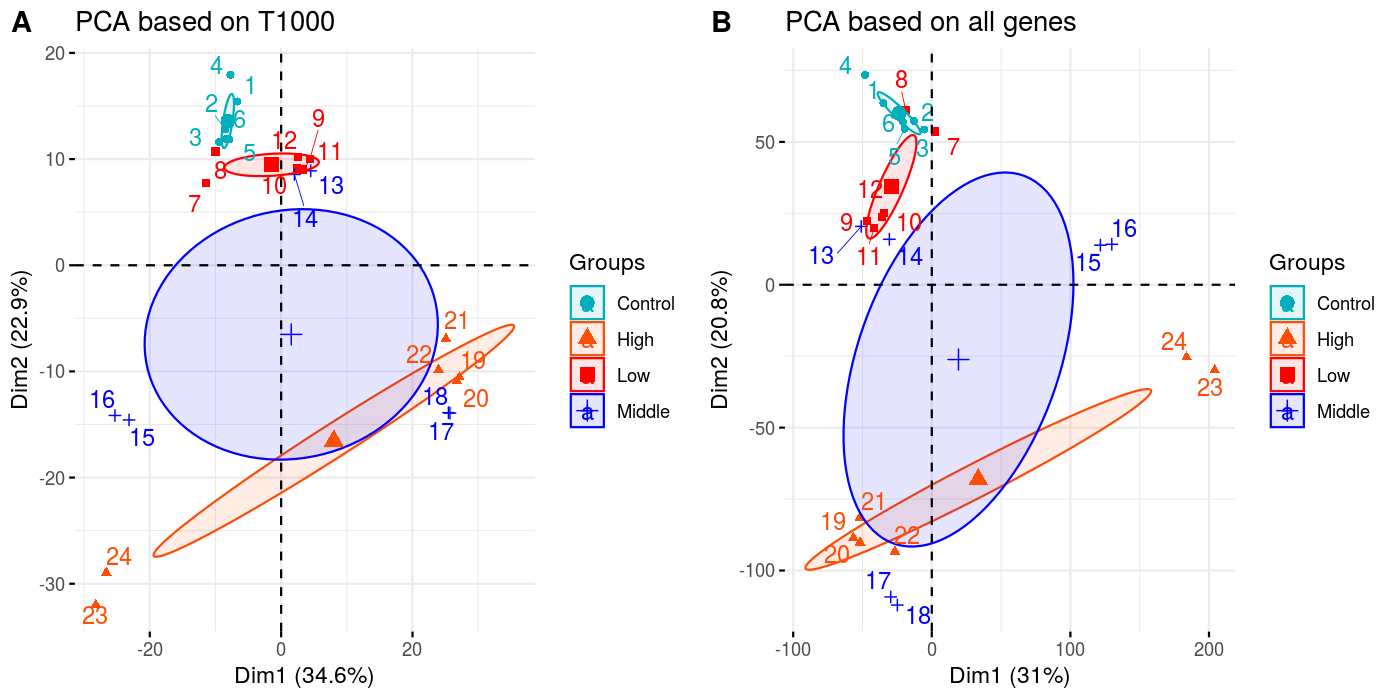

Supplement: Figure S5 [file peerj-07-7975-s005.zip › Supplementary_Figures_S5/phenobarbital.Human.in_vitro.Liver.tiff]

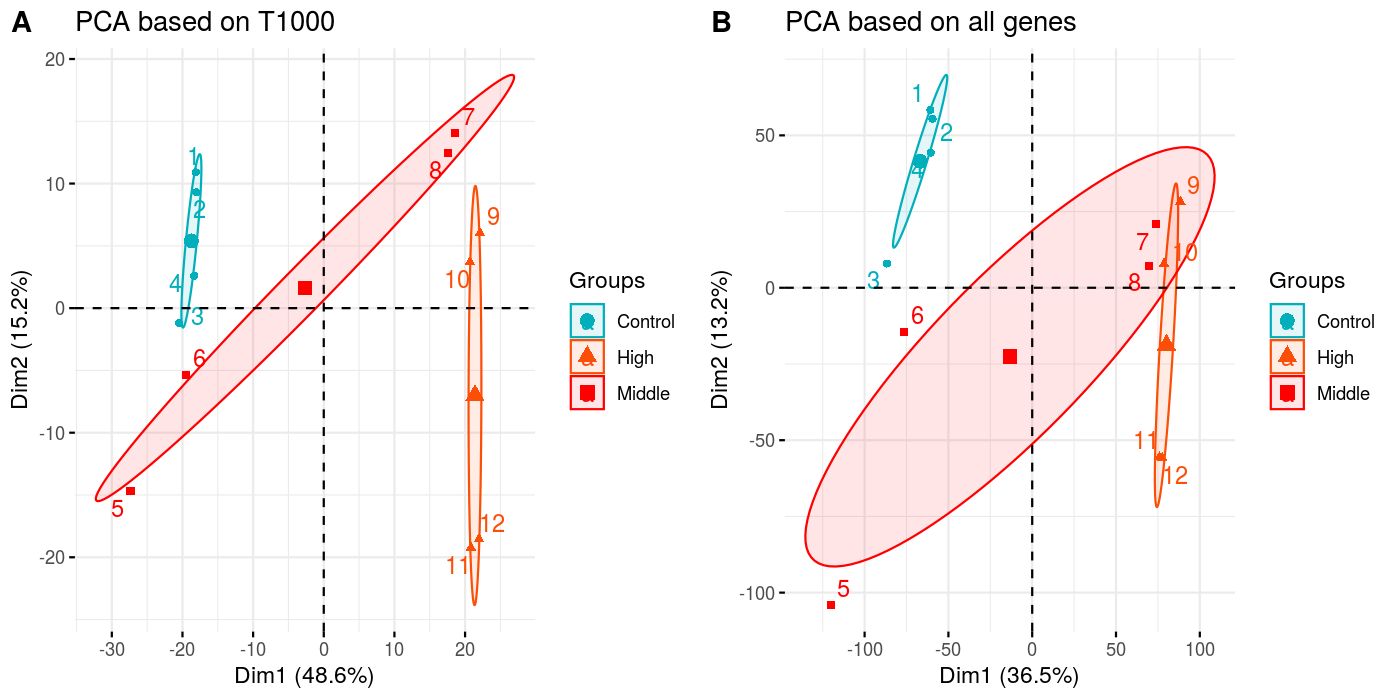

Supplement: Figure S5 [file peerj-07-7975-s005.zip › Supplementary_Figures_S5/naproxen.Human.in_vitro.Liver.tiff]

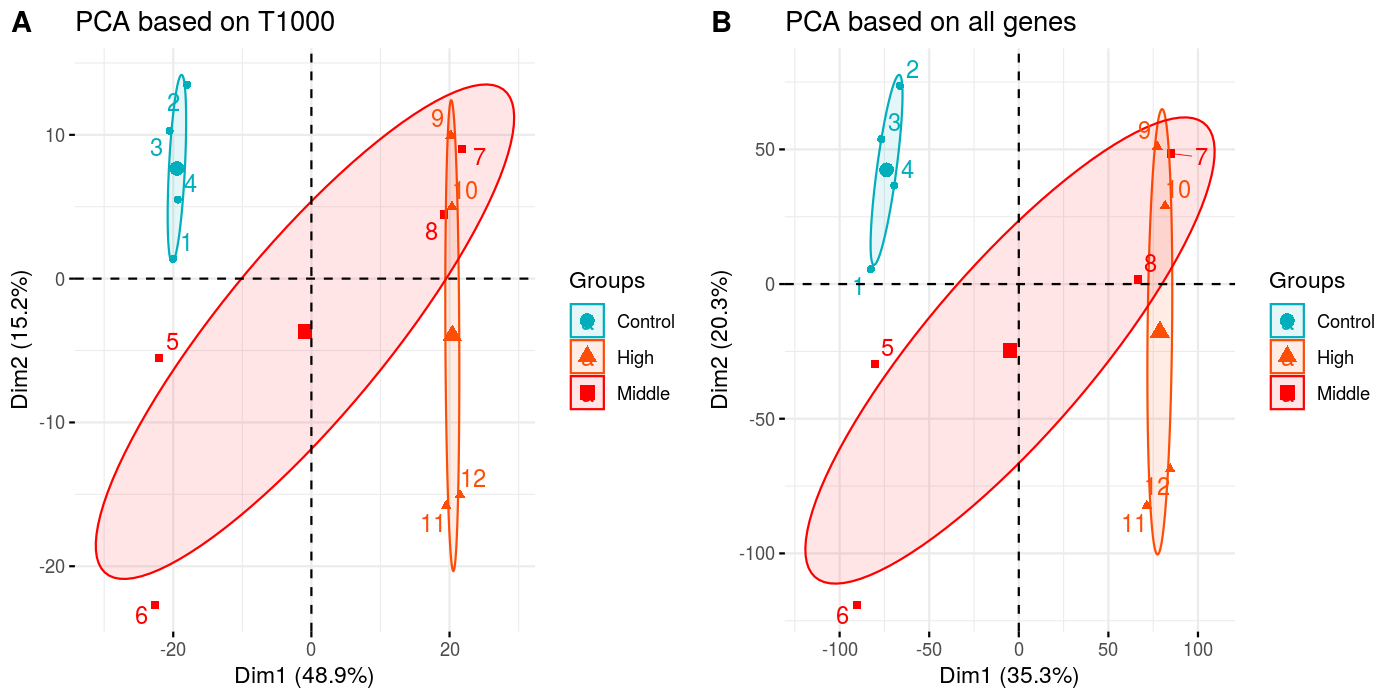

Supplement: Figure S5 [file peerj-07-7975-s005.zip › Supplementary_Figures_S5/terbinafine.Human.in_vitro.Liver.tiff]

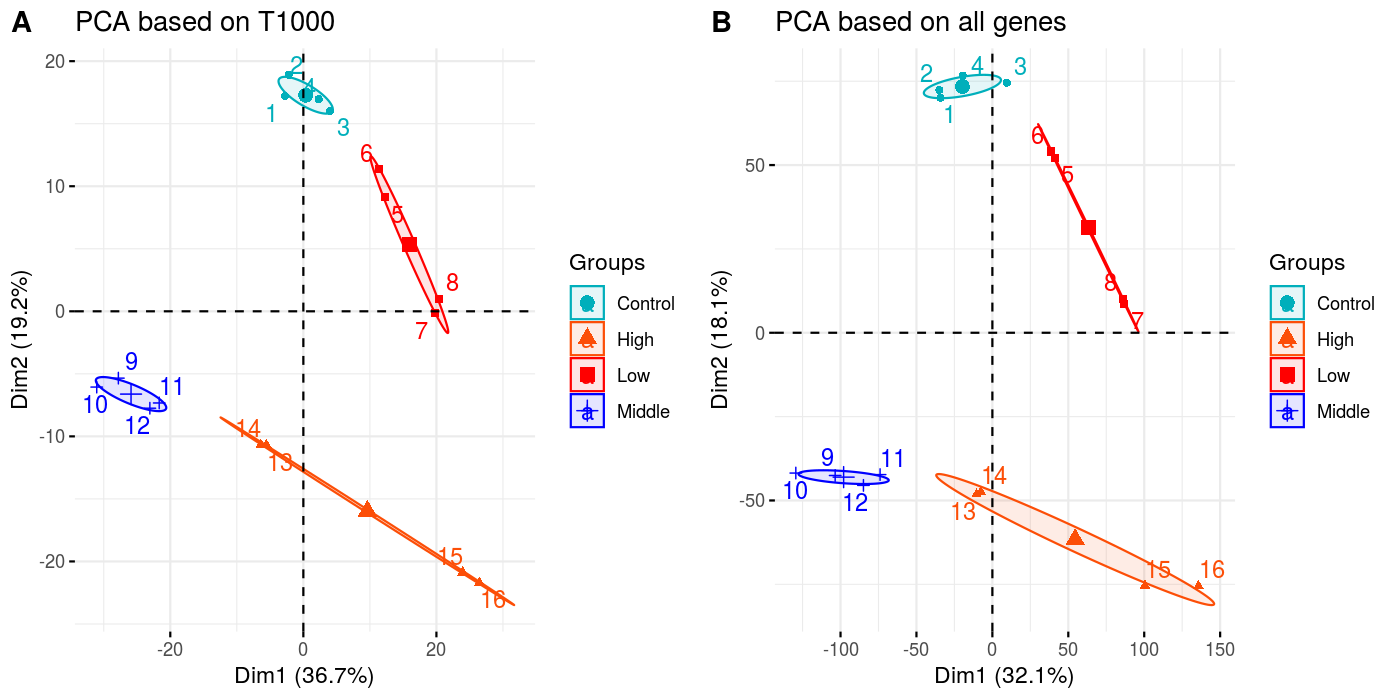

Supplement: Figure S5 [file peerj-07-7975-s005.zip › Supplementary_Figures_S5/aflatoxin_B1.Human.in_vitro.Liver.tiff]

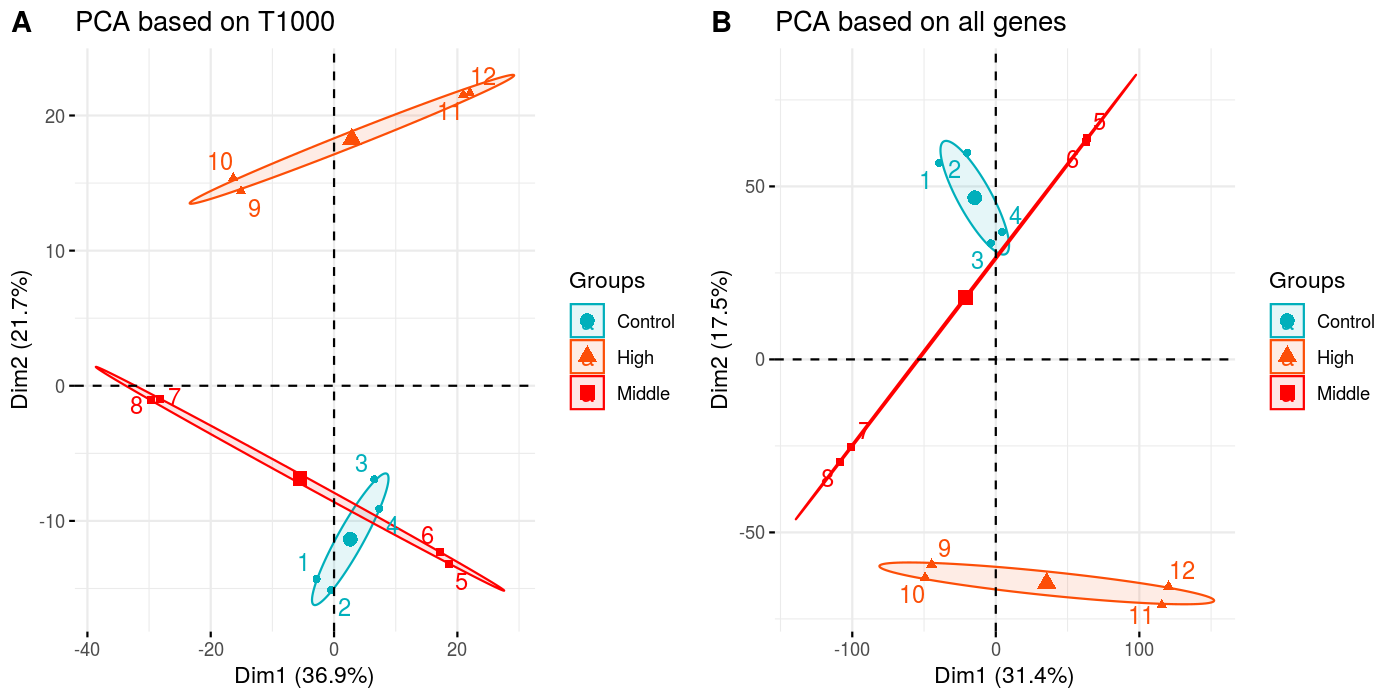

Supplement: Figure S5 [file peerj-07-7975-s005.zip › Supplementary_Figures_S5/sulindac.Human.in_vitro.Liver.tiff]

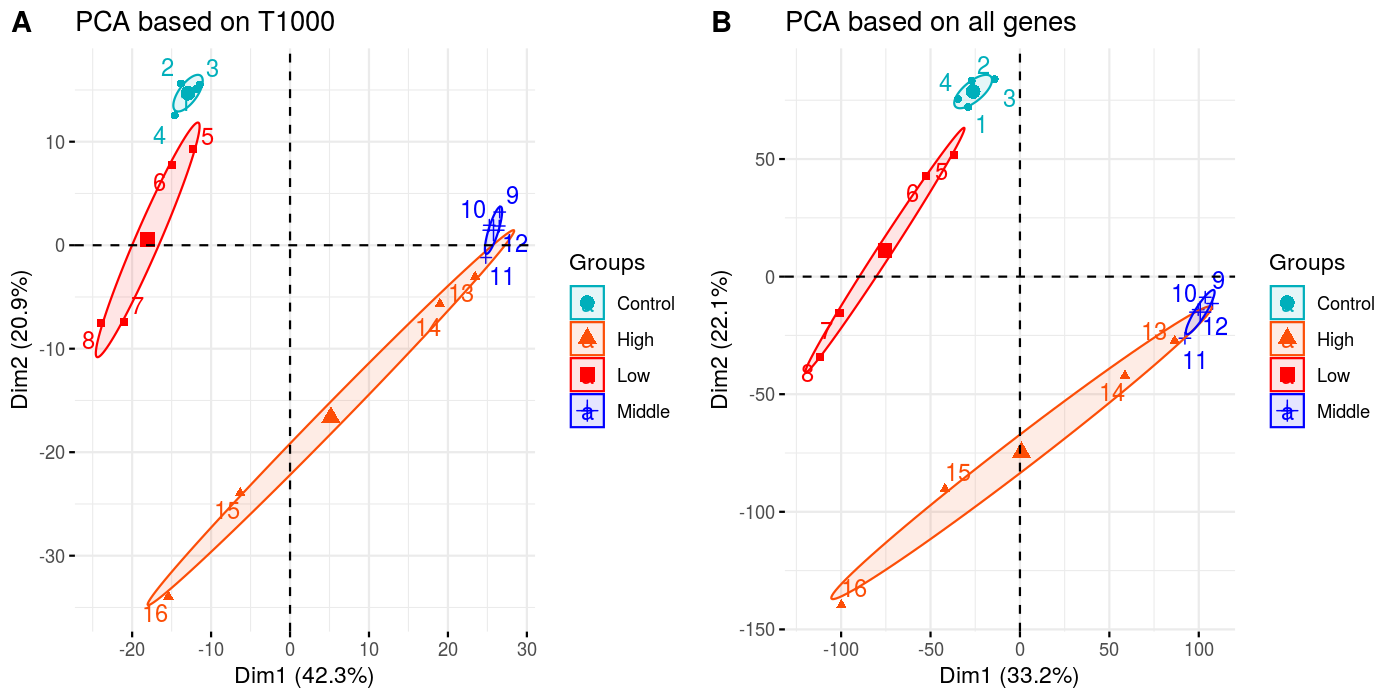

Supplement: Figure S5 [file peerj-07-7975-s005.zip › Supplementary_Figures_S5/galactosamine.Human.in_vitro.Liver.tiff]

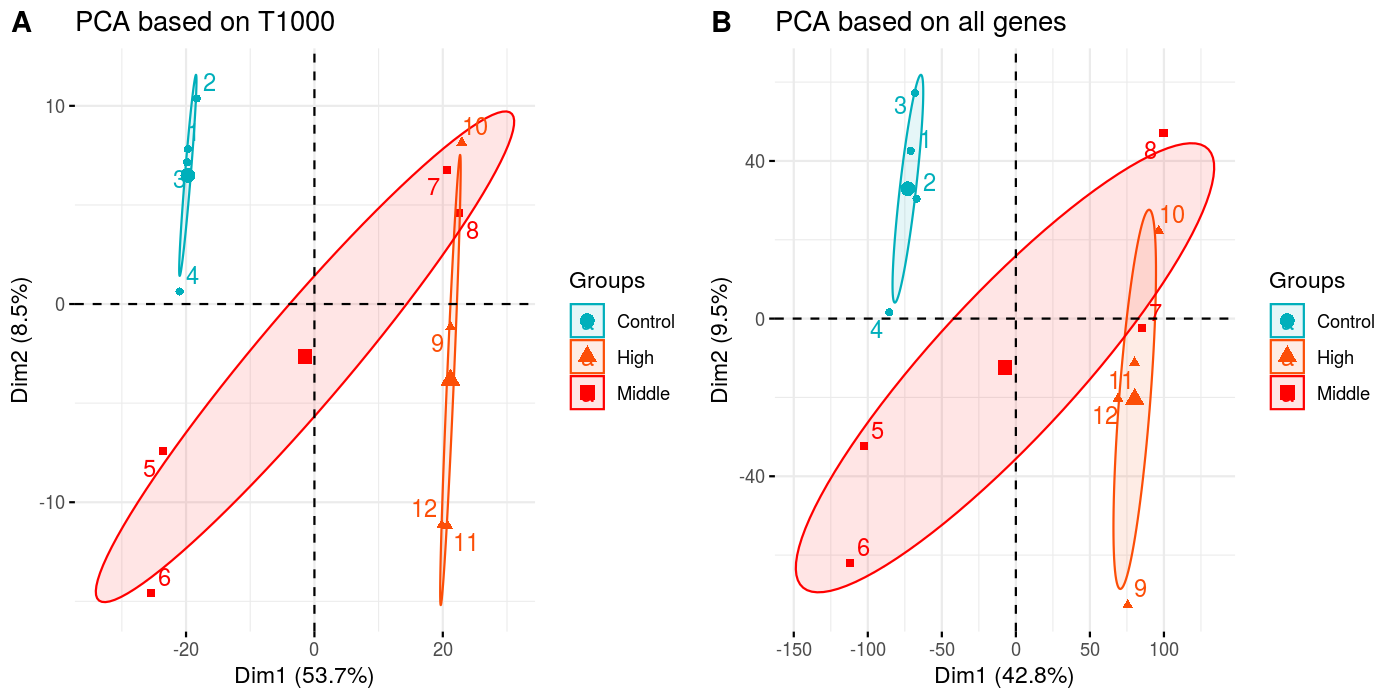

Supplement: Figure S5 [file peerj-07-7975-s005.zip › Supplementary_Figures_S5/ethinylestradiol.Human.in_vitro.Liver.tiff]

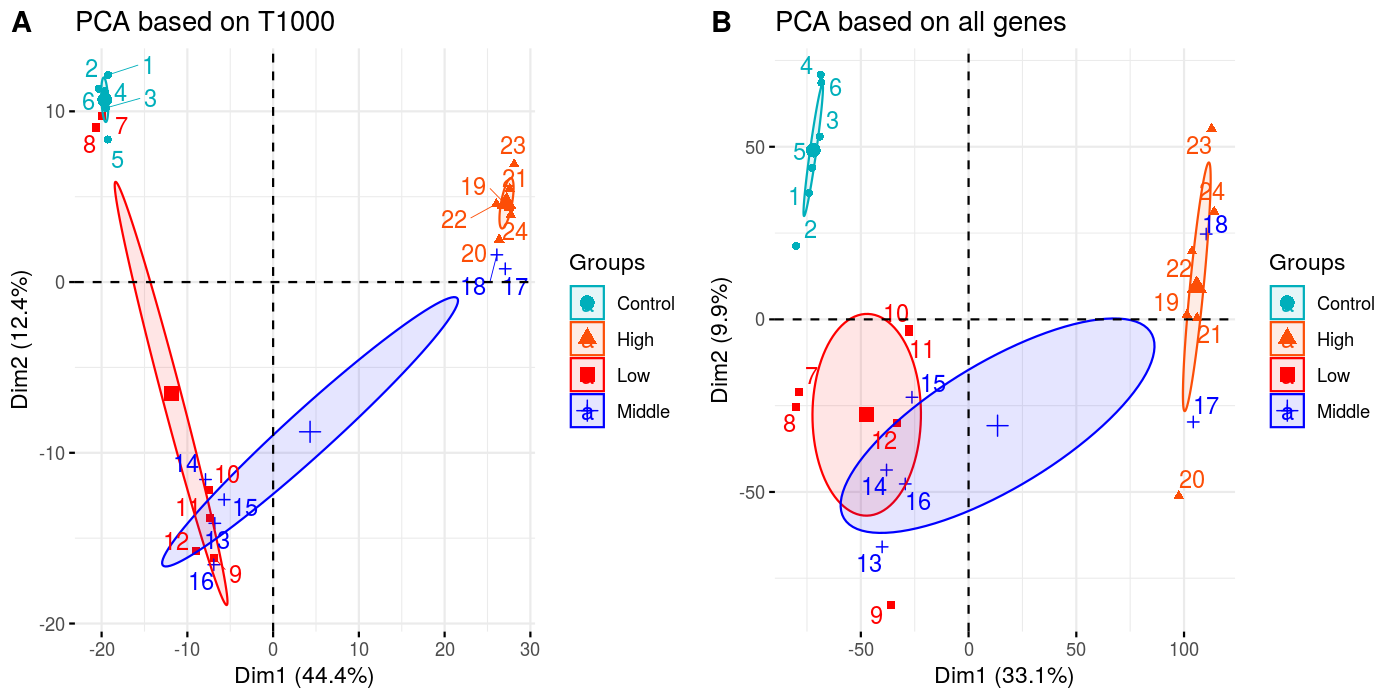

Supplement: Figure S5 [file peerj-07-7975-s005.zip › Supplementary_Figures_S5/clofibrate.Human.in_vitro.Liver.tiff]

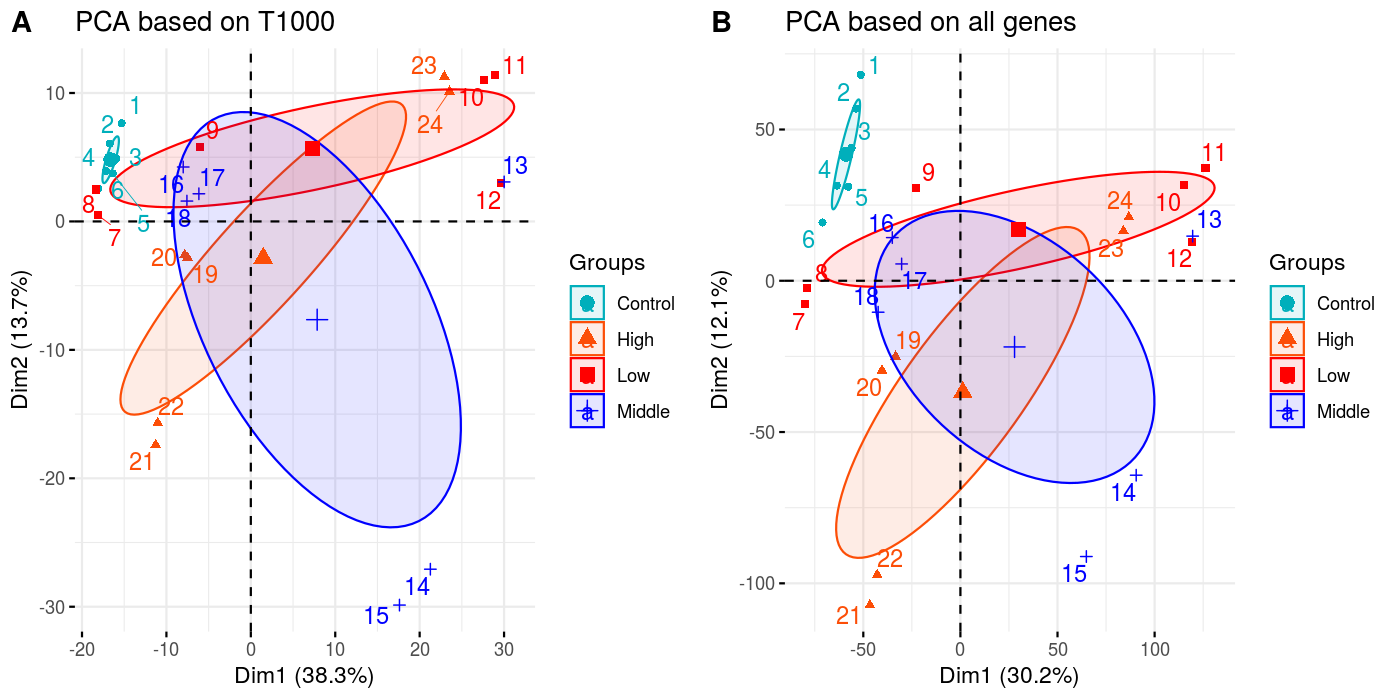

Supplement: Figure S5 [file peerj-07-7975-s005.zip › Supplementary_Figures_S5/isoniazid.Human.in_vitro.Liver.tiff]

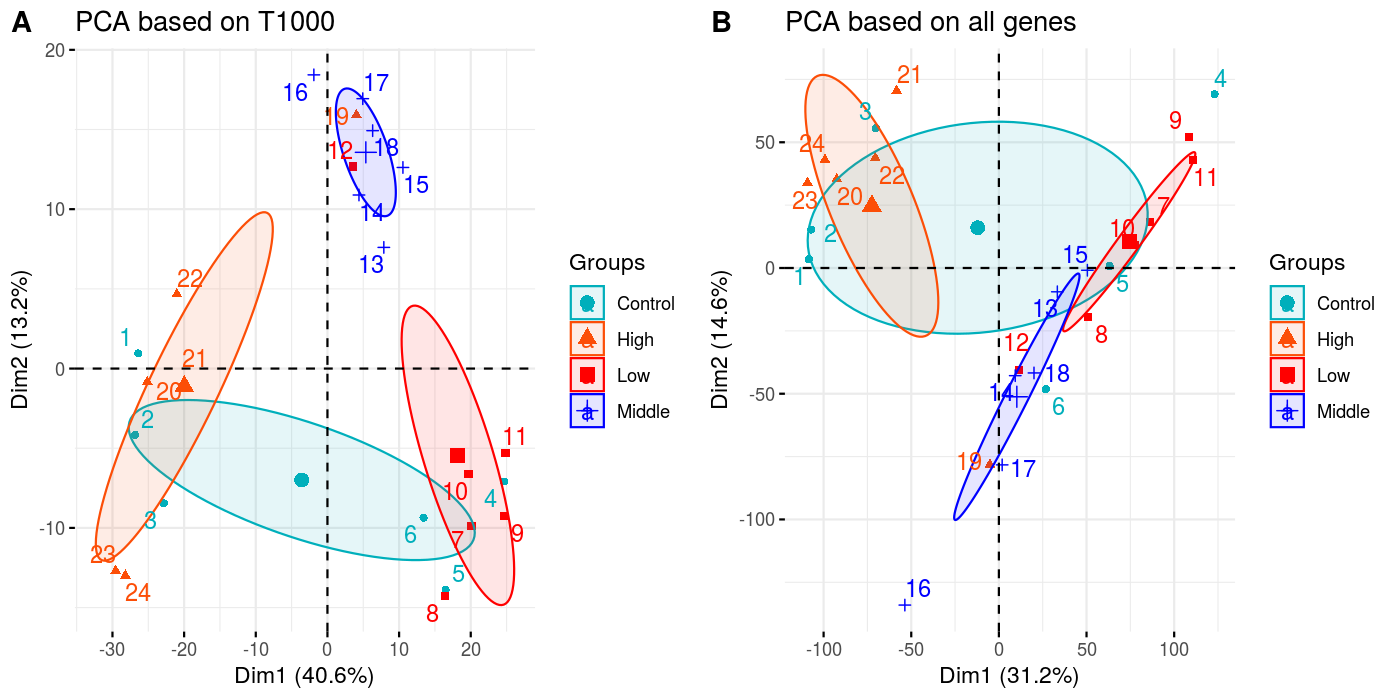

Supplement: Figure S5 [file peerj-07-7975-s005.zip › Supplementary_Figures_S5/tetracycline.Human.in_vitro.Liver.tiff]

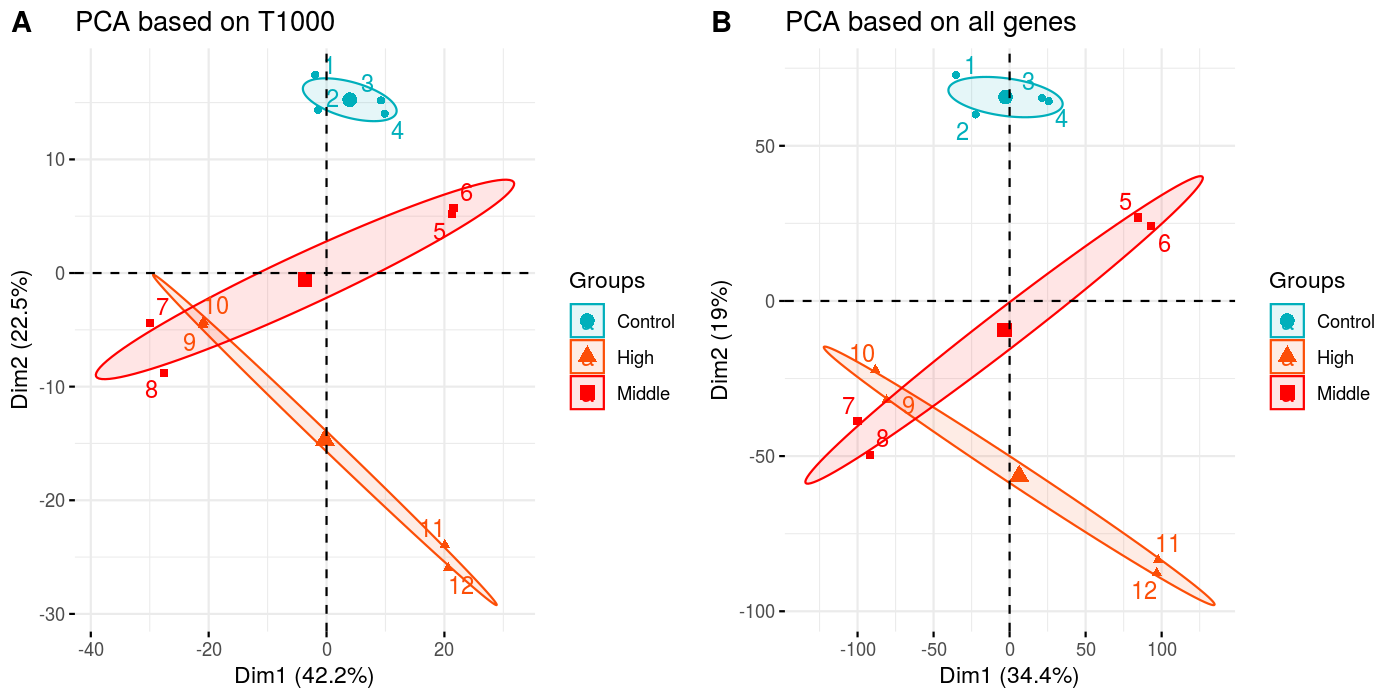

Supplement: Figure S5 [file peerj-07-7975-s005.zip › Supplementary_Figures_S5/papaverine.Human.in_vitro.Liver.tiff]

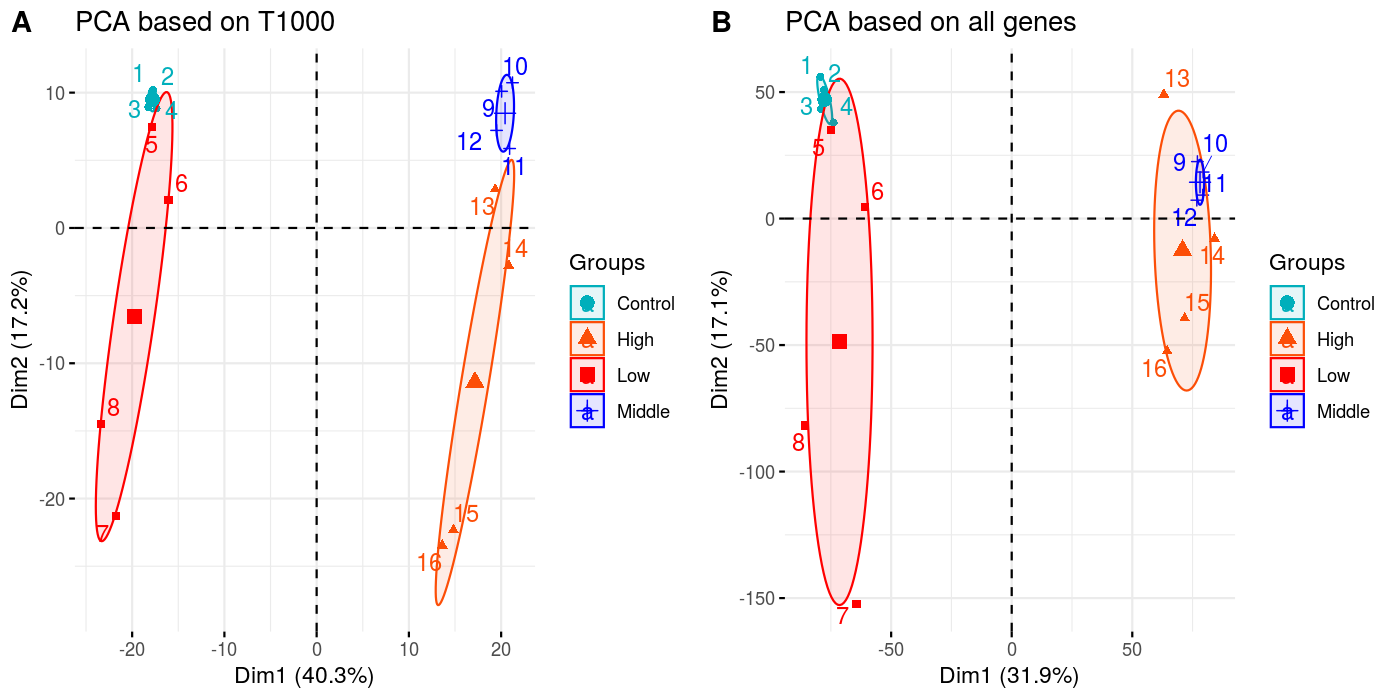

Supplement: Figure S5 [file peerj-07-7975-s005.zip › Supplementary_Figures_S5/amphotericin_B.Human.in_vitro.Liver.tiff]

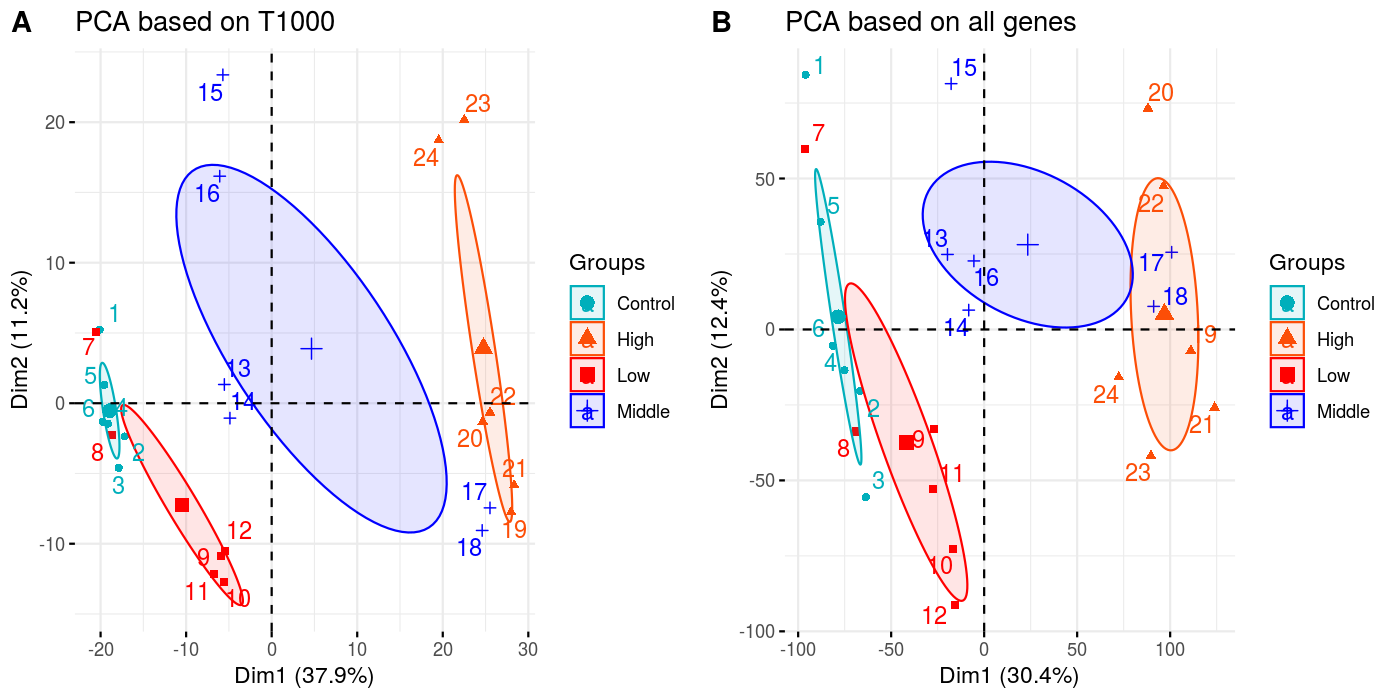

Supplement: Figure S5 [file peerj-07-7975-s005.zip › Supplementary_Figures_S5/perhexiline.Human.in_vitro.Liver.tiff]

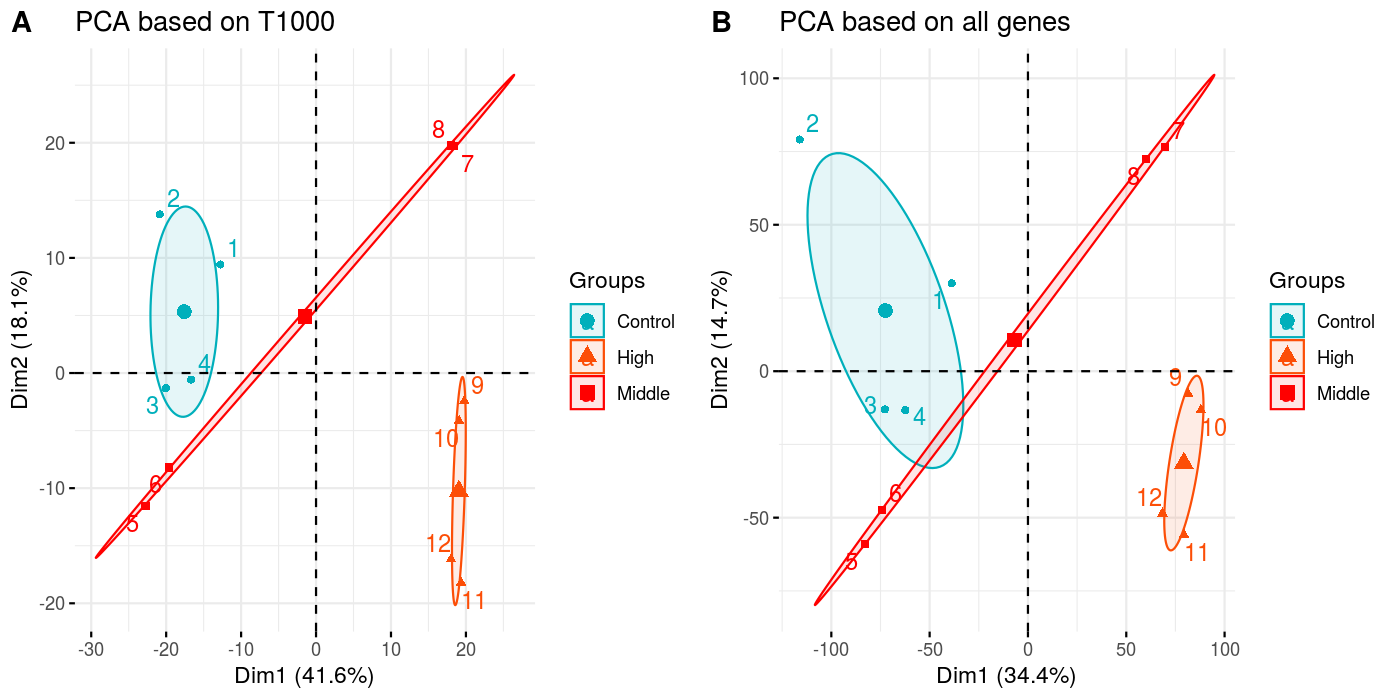

Supplement: Figure S5 [file peerj-07-7975-s005.zip › Supplementary_Figures_S5/etoposide.Human.in_vitro.Liver.tiff]

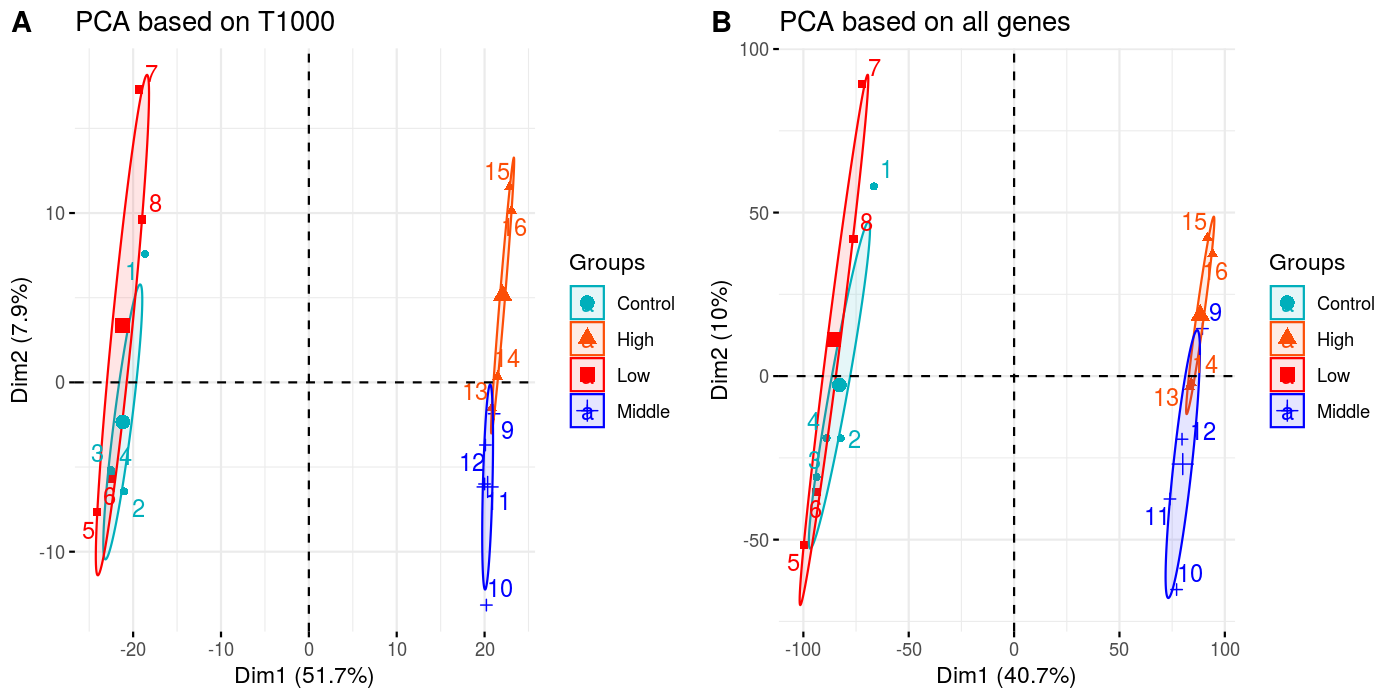

Supplement: Figure S5 [file peerj-07-7975-s005.zip › Supplementary_Figures_S5/N-nitrosomorpholine.Human.in_vitro.Liver.tiff]

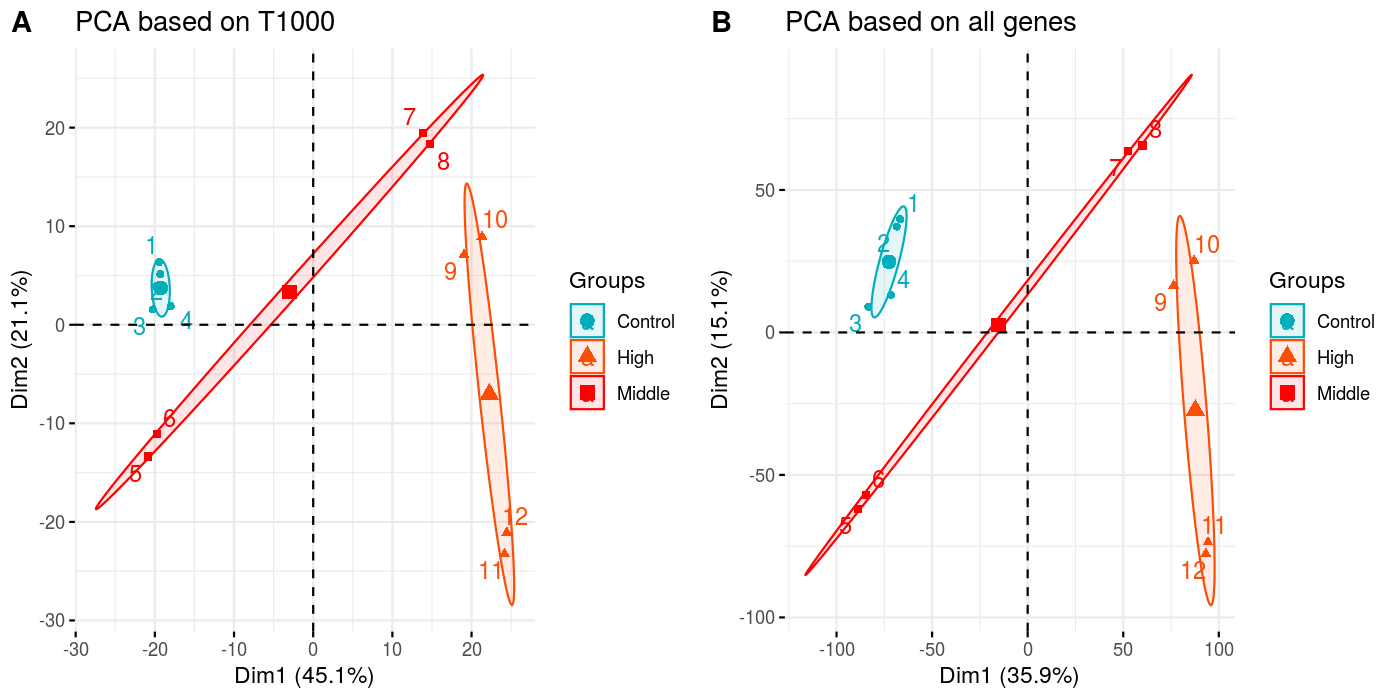

Supplement: Figure S5 [file peerj-07-7975-s005.zip › Supplementary_Figures_S5/captopril.Human.in_vitro.Liver.tiff]

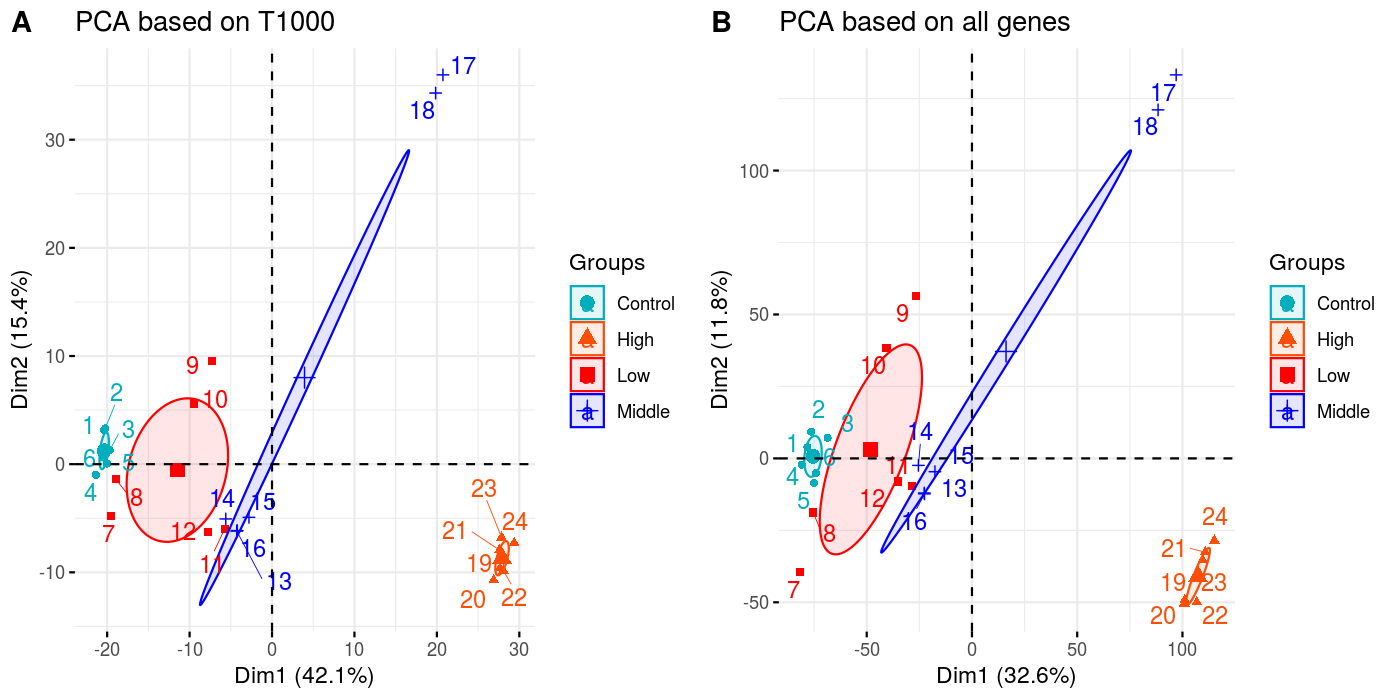

Supplement: Figure S5 [file peerj-07-7975-s005.zip › Supplementary_Figures_S5/transforming_growth_factor_beta_1.Human.in_vitro.Liver.tiff]

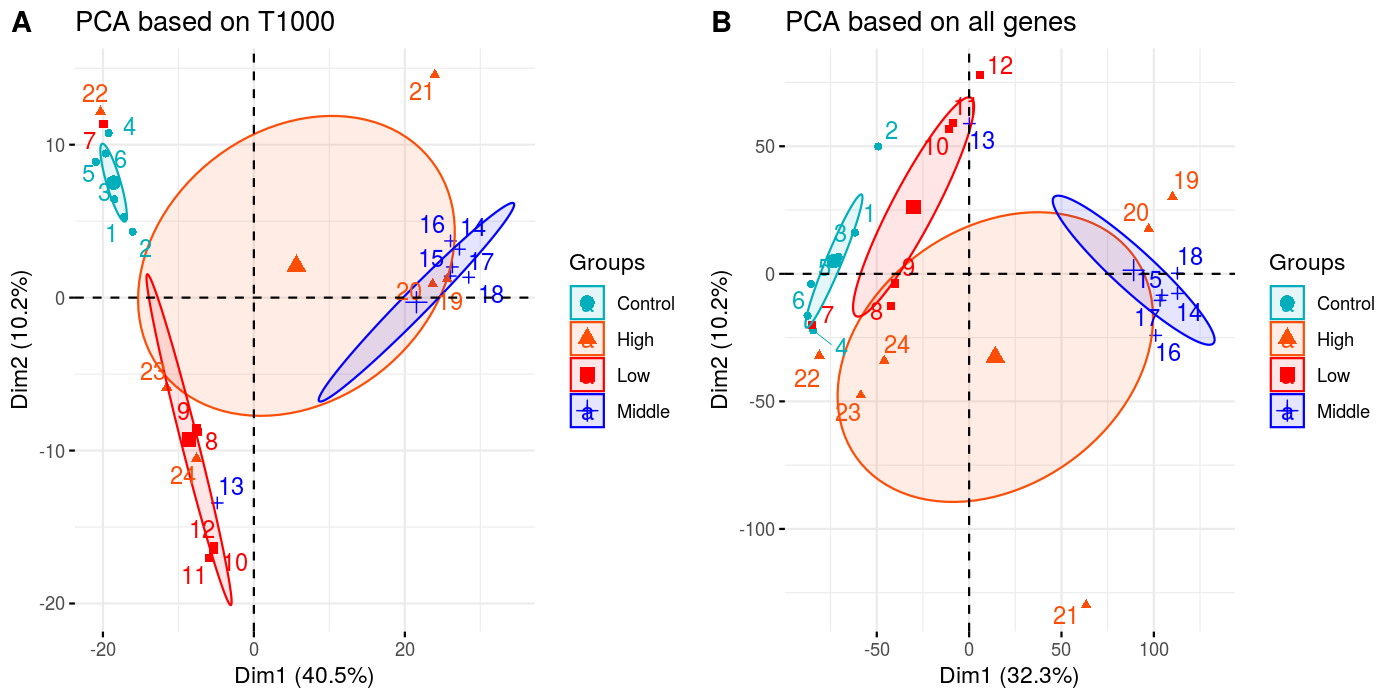

Supplement: Figure S5 [file peerj-07-7975-s005.zip › Supplementary_Figures_S5/chlorpromazine.Human.in_vitro.Liver.tiff]

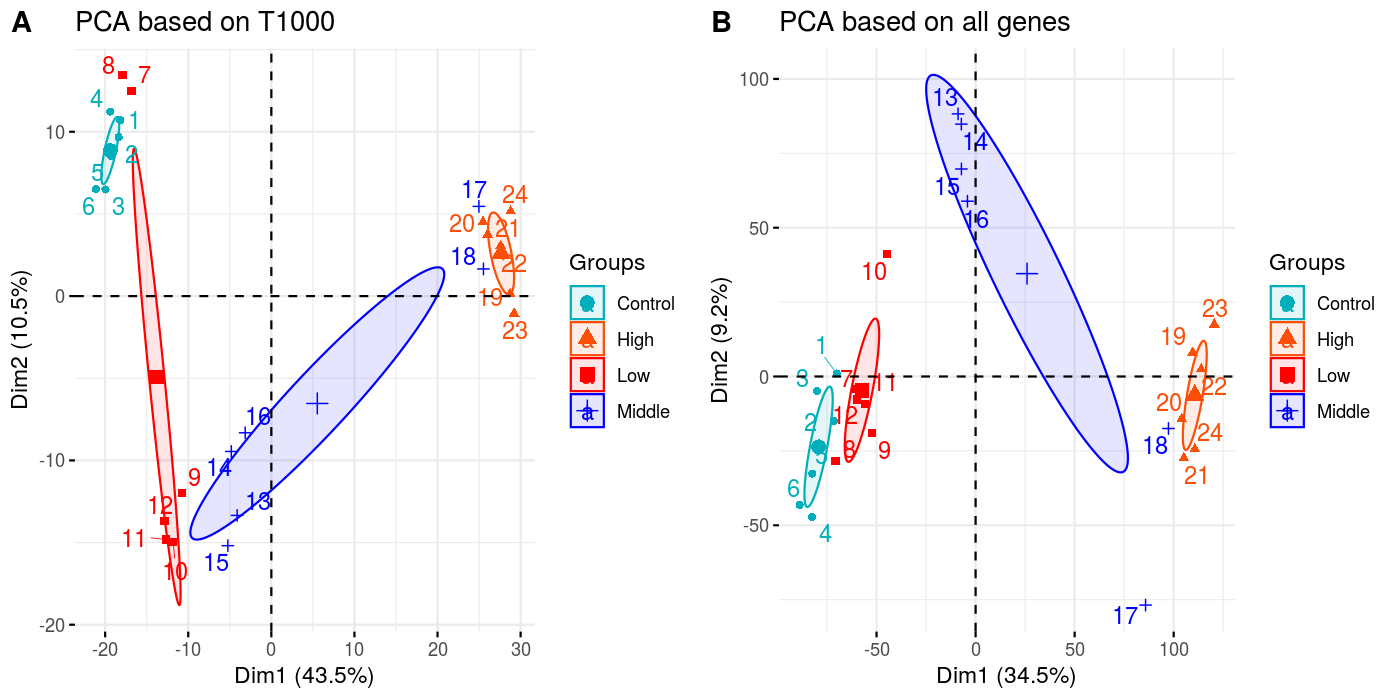

Supplement: Figure S5 [file peerj-07-7975-s005.zip › Supplementary_Figures_S5/aspirin.Human.in_vitro.Liver.tiff]

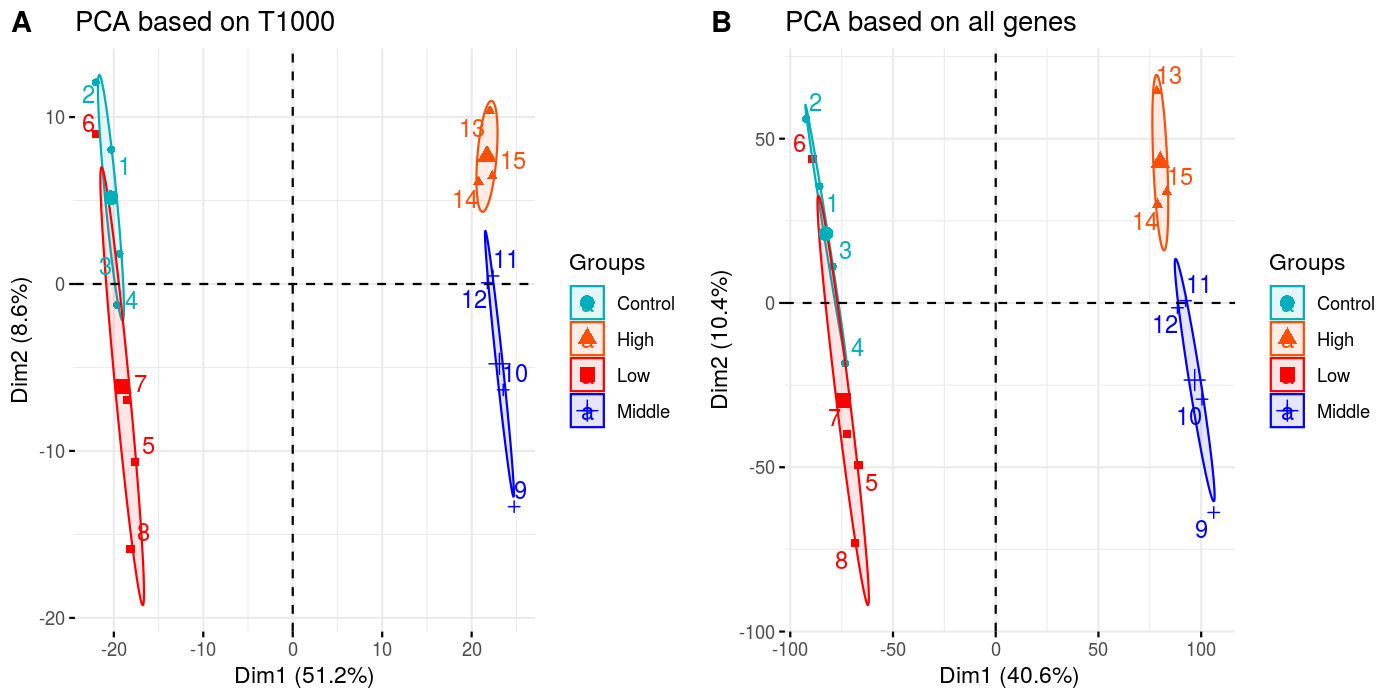

Supplement: Figure S5 [file peerj-07-7975-s005.zip › Supplementary_Figures_S5/trimethadione.Human.in_vitro.Liver.tiff]

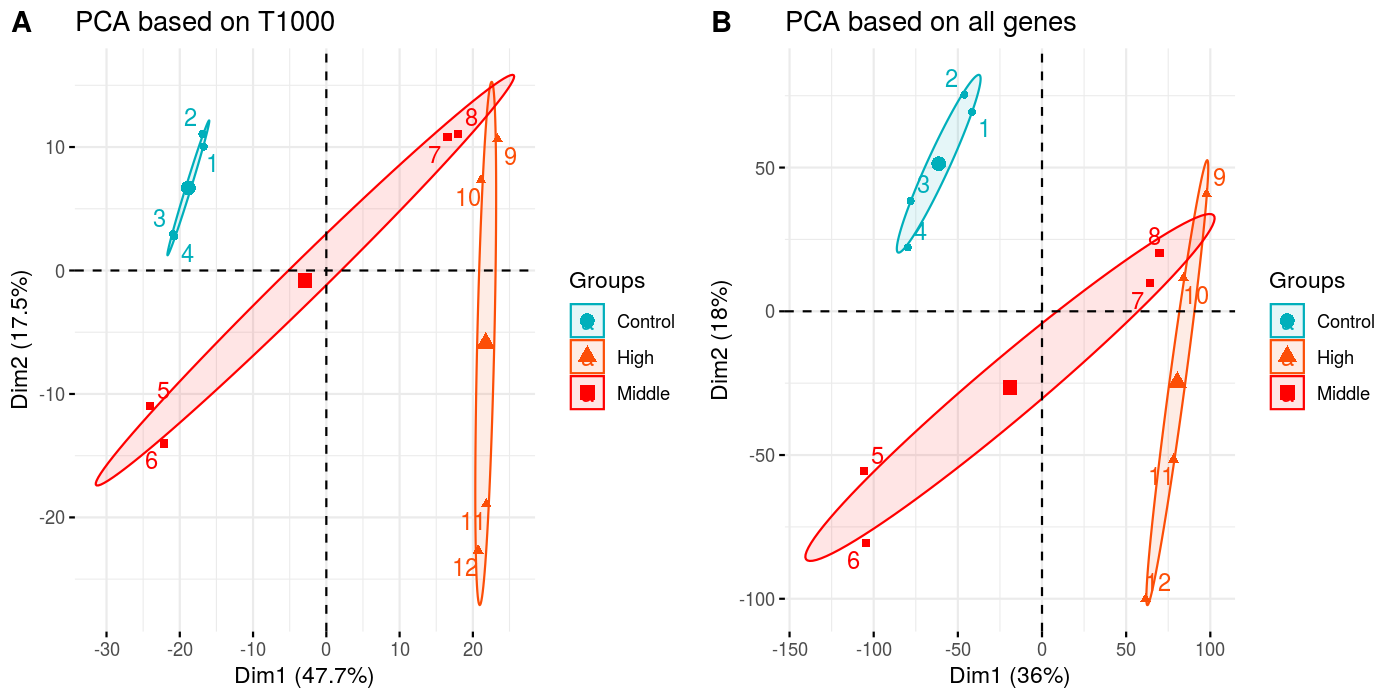

Supplement: Figure S5 [file peerj-07-7975-s005.zip › Supplementary_Figures_S5/penicillamine.Human.in_vitro.Liver.tiff]

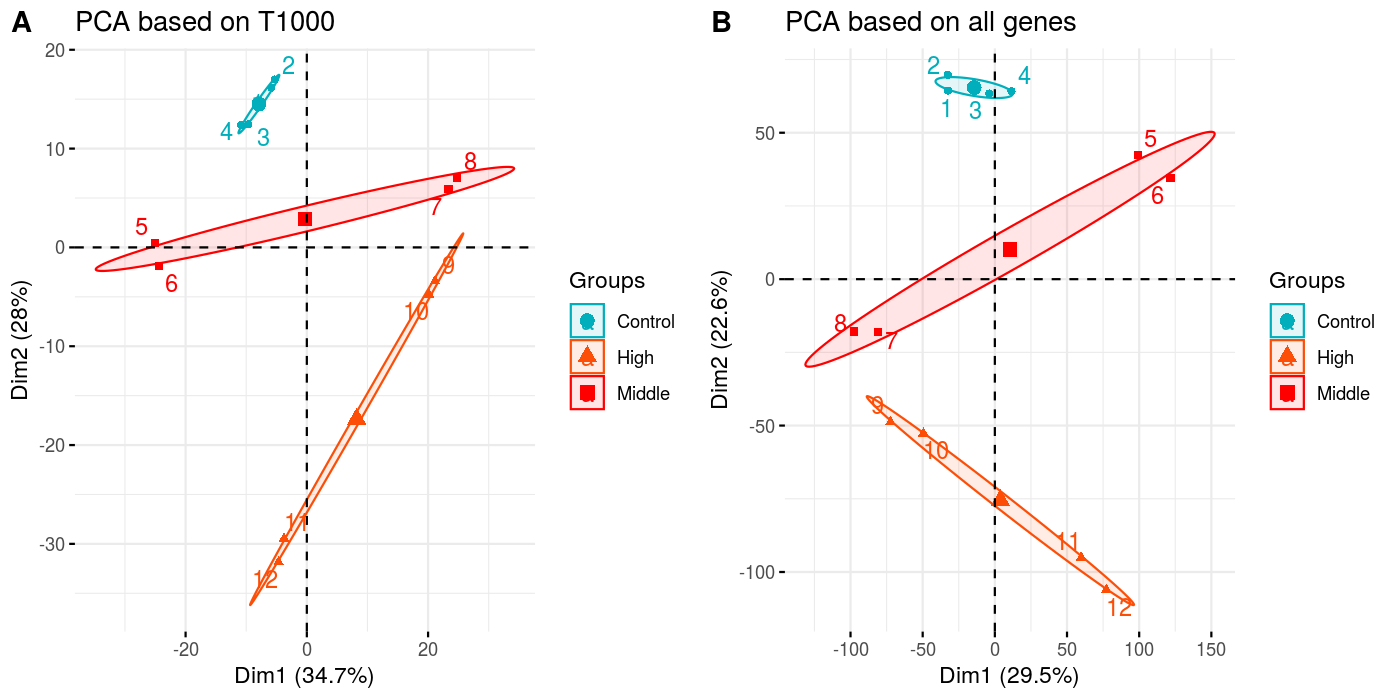

Supplement: Figure S5 [file peerj-07-7975-s005.zip › Supplementary_Figures_S5/disopyramide.Human.in_vitro.Liver.tiff]

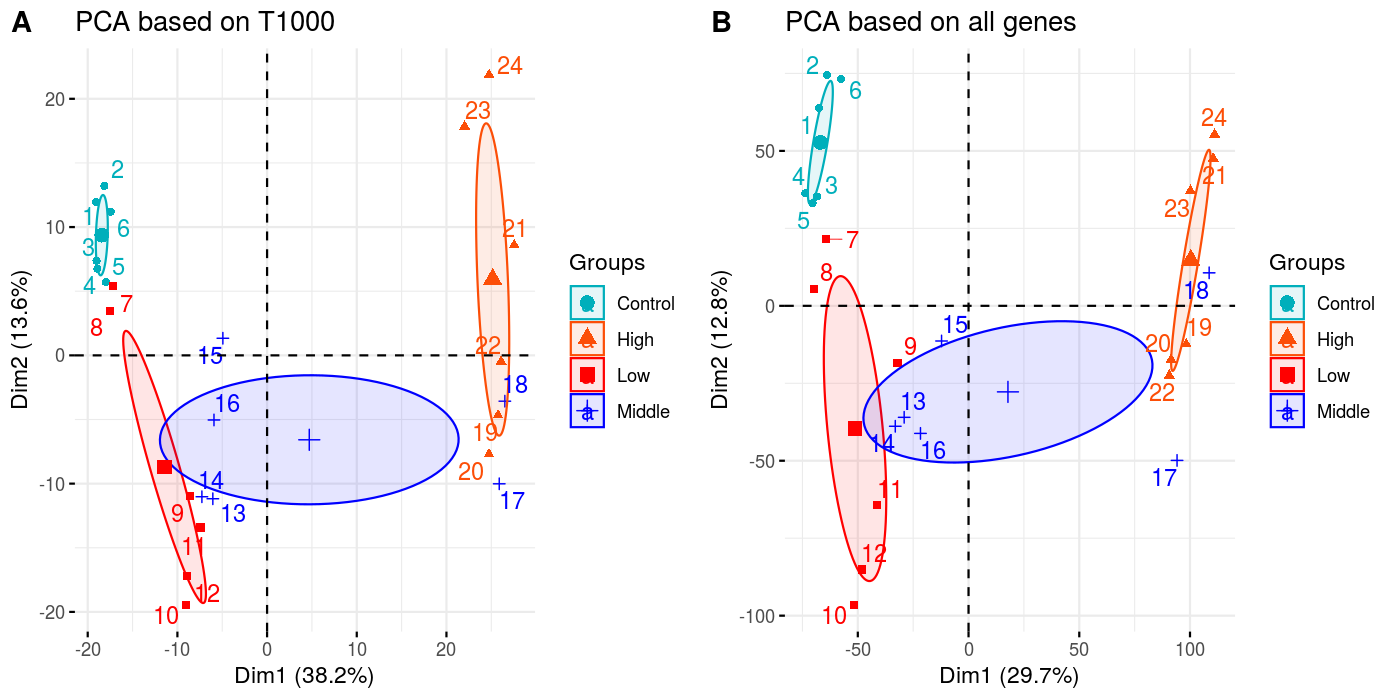

Supplement: Figure S5 [file peerj-07-7975-s005.zip › Supplementary_Figures_S5/phenylbutazone.Human.in_vitro.Liver.tiff]

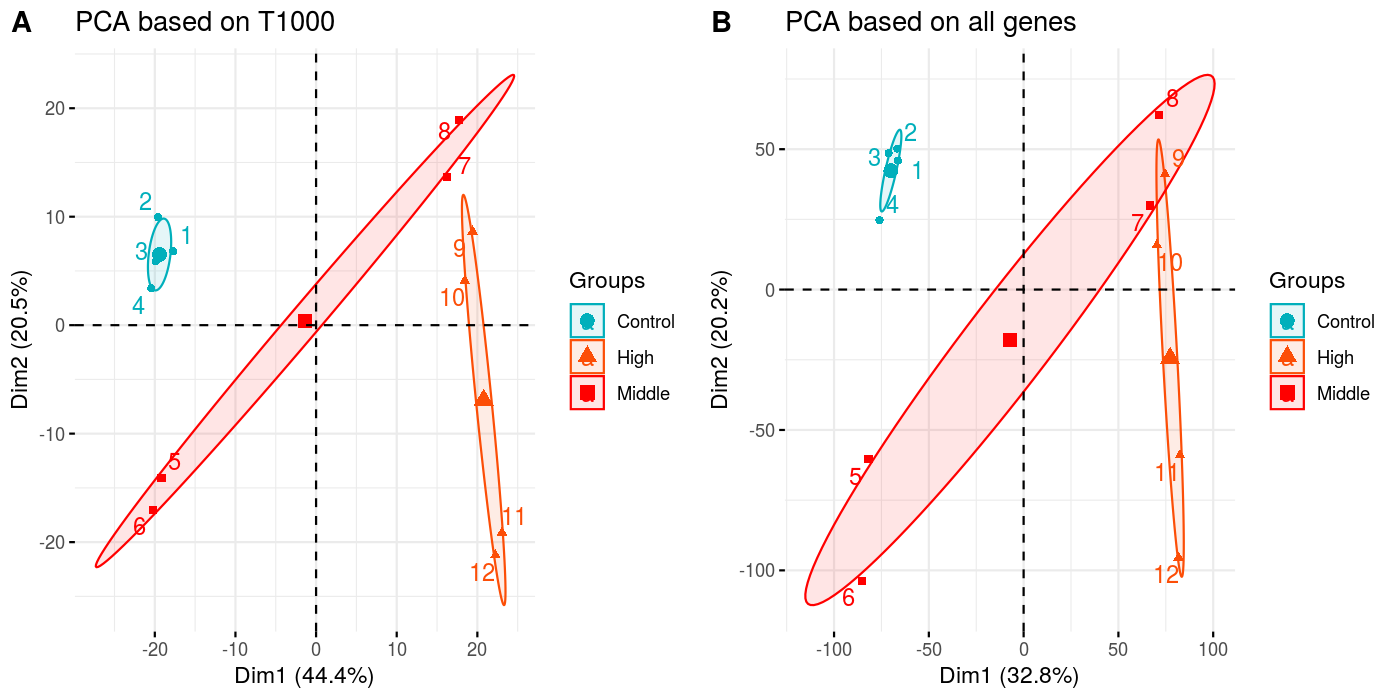

Supplement: Figure S5 [file peerj-07-7975-s005.zip › Supplementary_Figures_S5/diltiazem.Human.in_vitro.Liver.tiff]

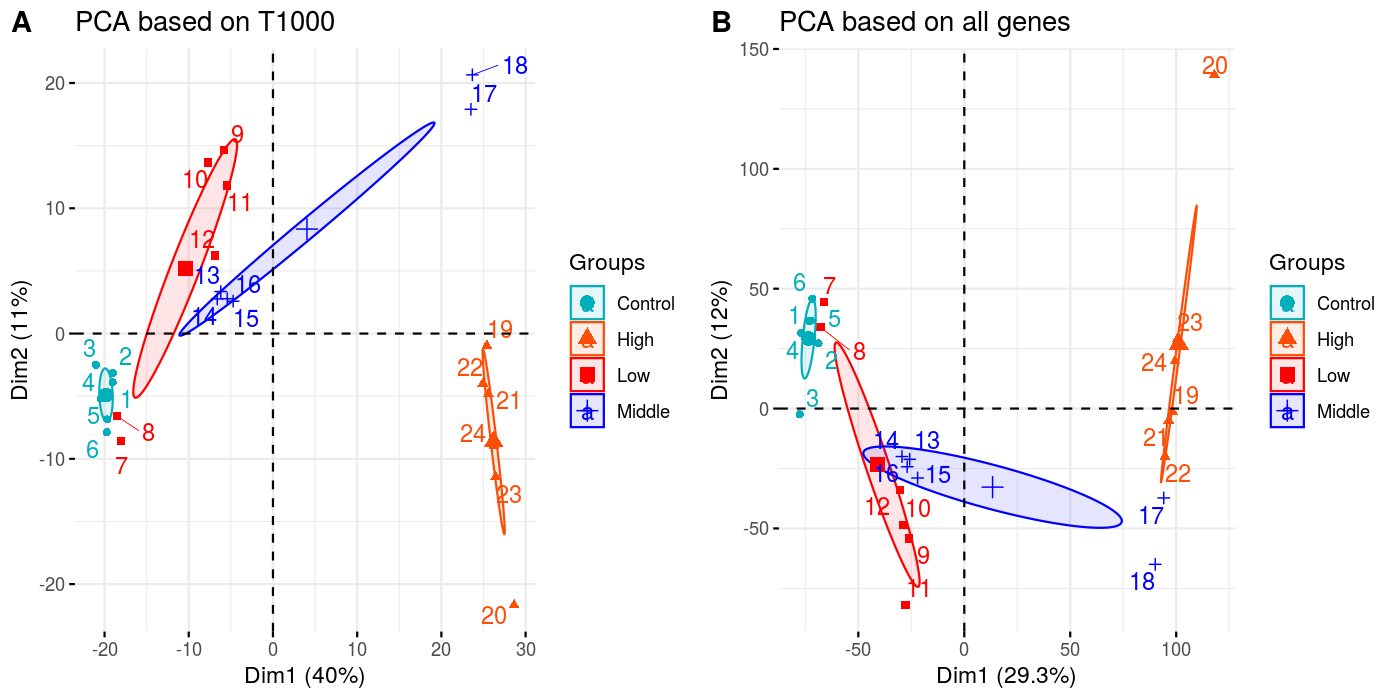

Supplement: Figure S5 [file peerj-07-7975-s005.zip › Supplementary_Figures_S5/interleukin_6,_human.Human.in_vitro.Liver.tiff]

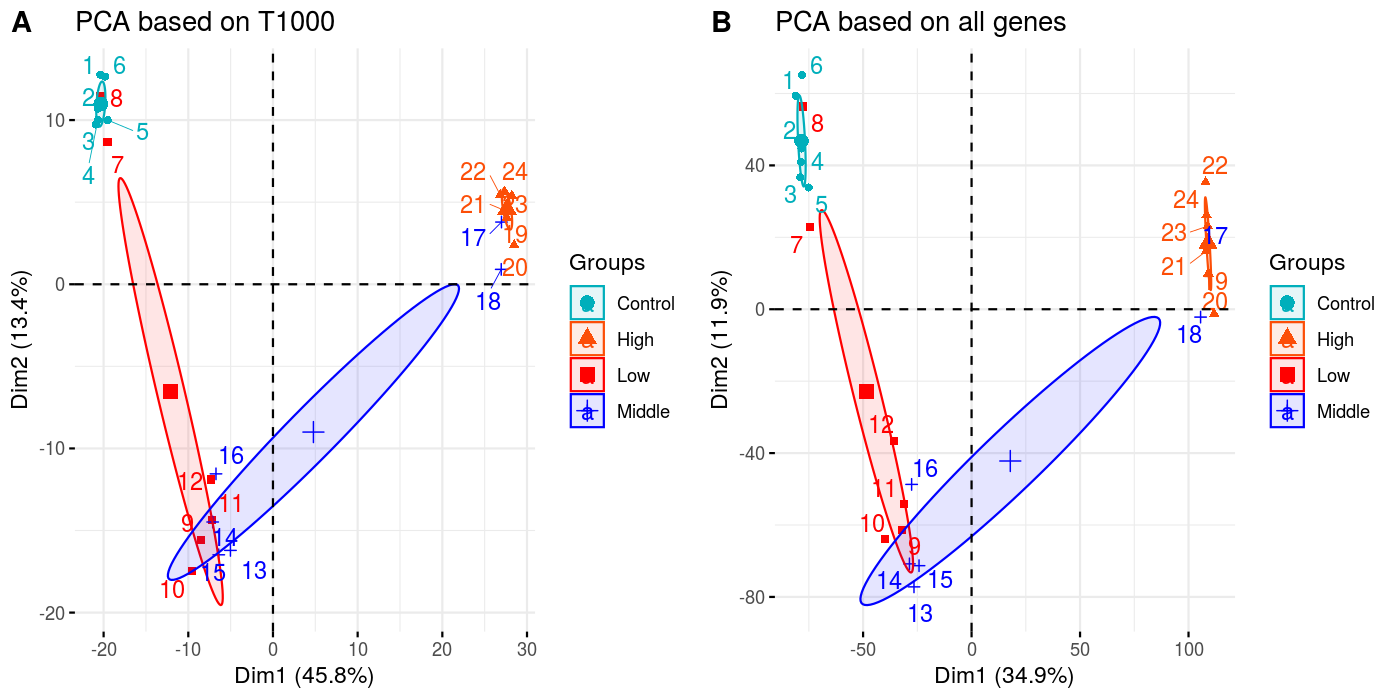

Supplement: Figure S5 [file peerj-07-7975-s005.zip › Supplementary_Figures_S5/amiodarone.Human.in_vitro.Liver.tiff]

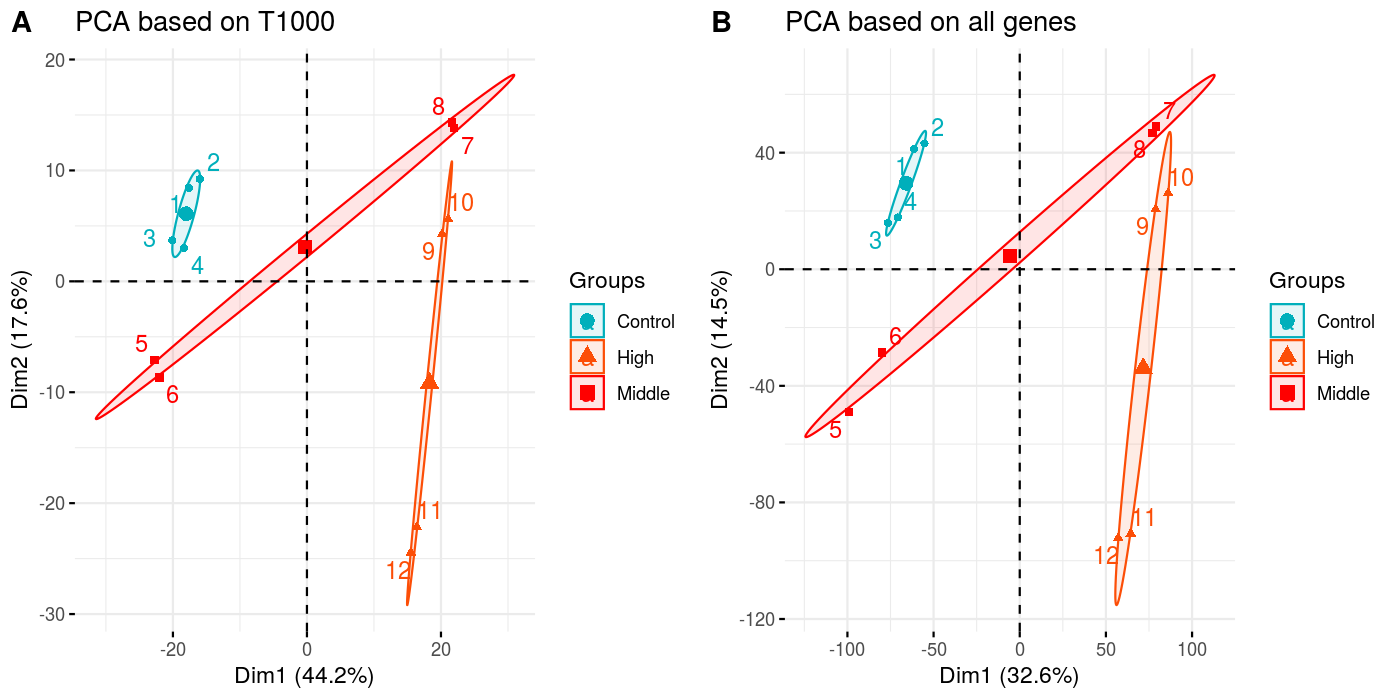

Supplement: Figure S5 [file peerj-07-7975-s005.zip › Supplementary_Figures_S5/tacrine.Human.in_vitro.Liver.tiff]

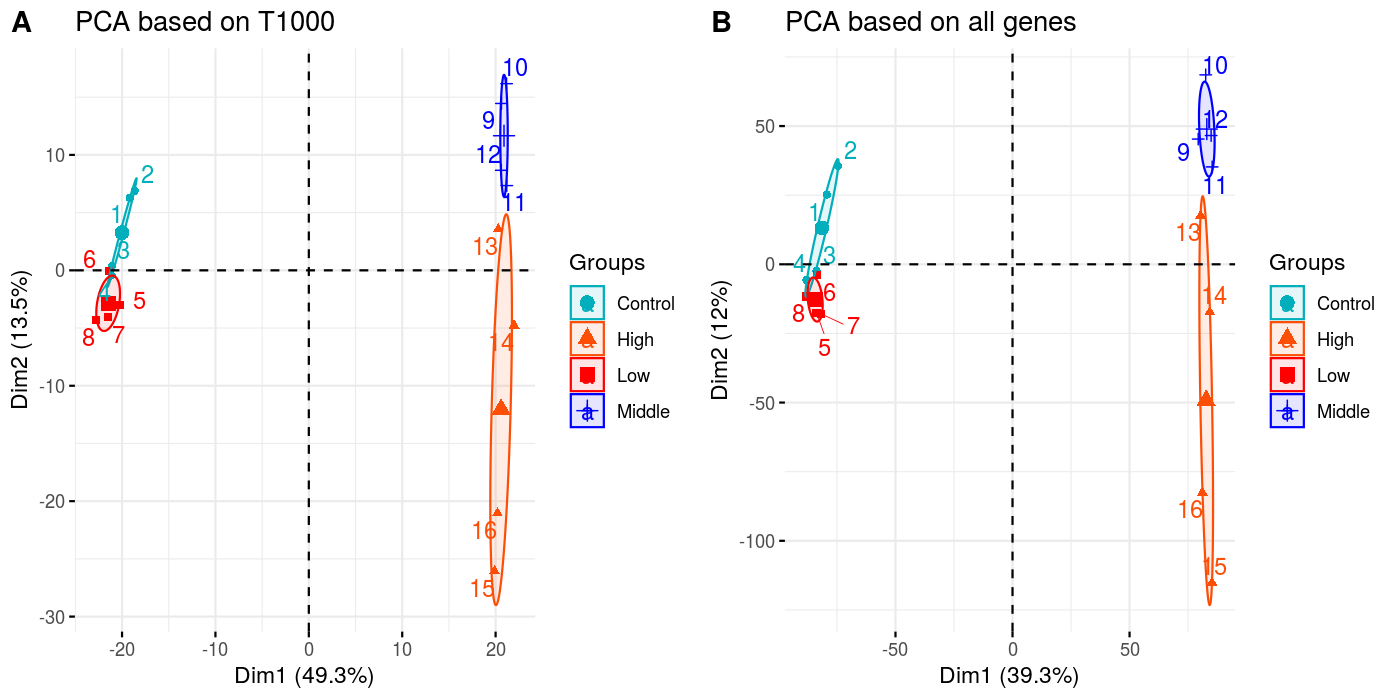

Supplement: Figure S5 [file peerj-07-7975-s005.zip › Supplementary_Figures_S5/dexamethasone.Human.in_vitro.Liver.tiff]

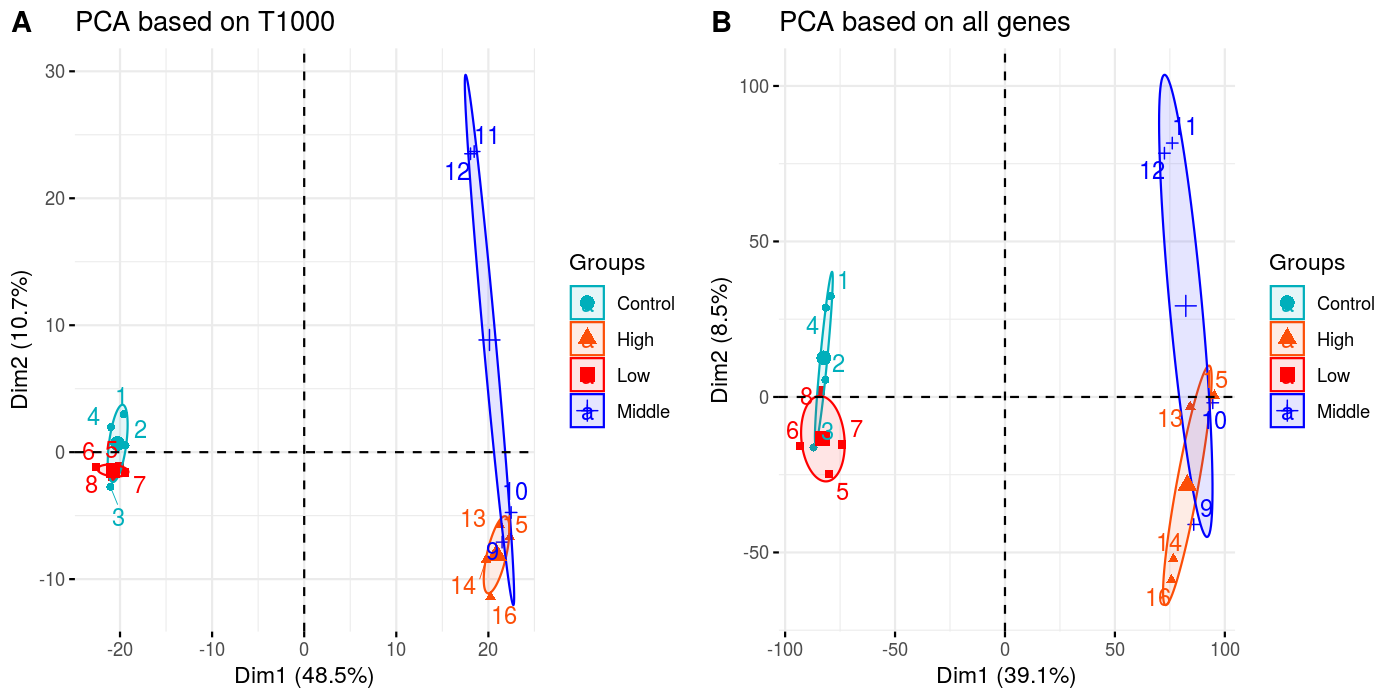

Supplement: Figure S5 [file peerj-07-7975-s005.zip › Supplementary_Figures_S5/triazolam.Human.in_vitro.Liver.tiff]

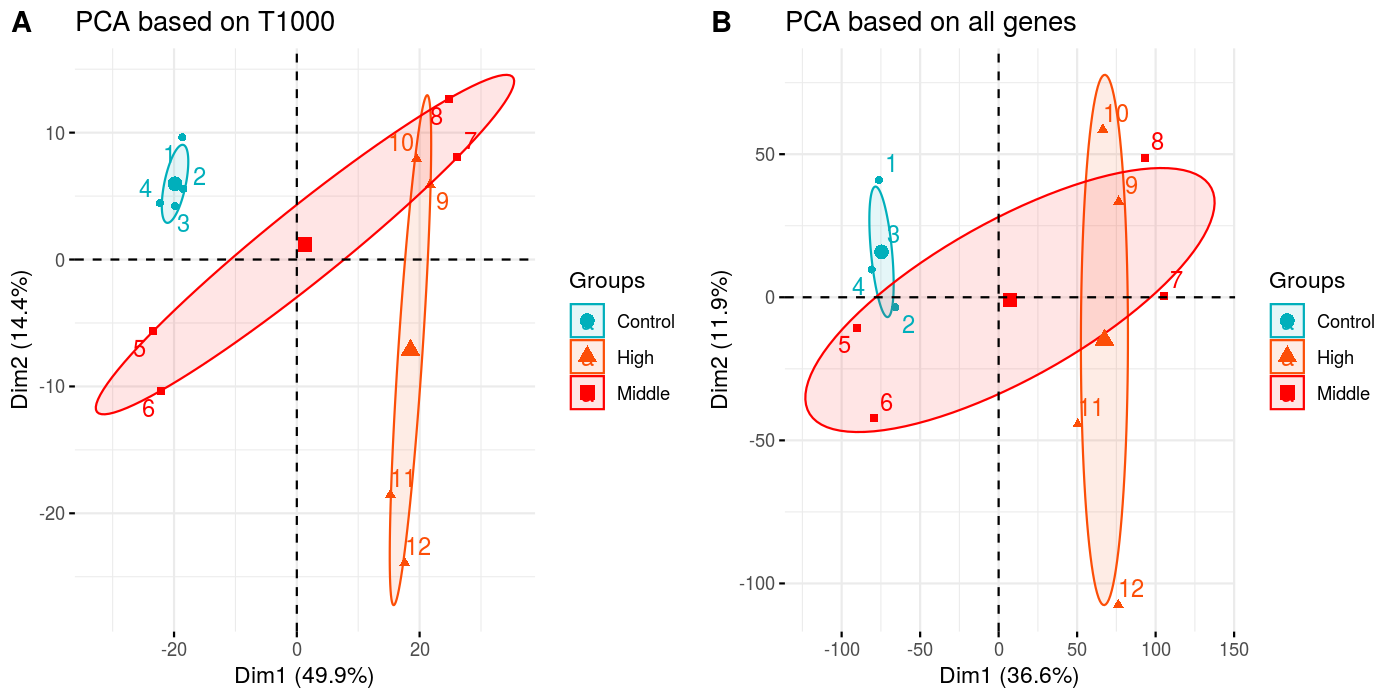

Supplement: Figure S5 [file peerj-07-7975-s005.zip › Supplementary_Figures_S5/nitrofurazone.Human.in_vitro.Liver.tiff]

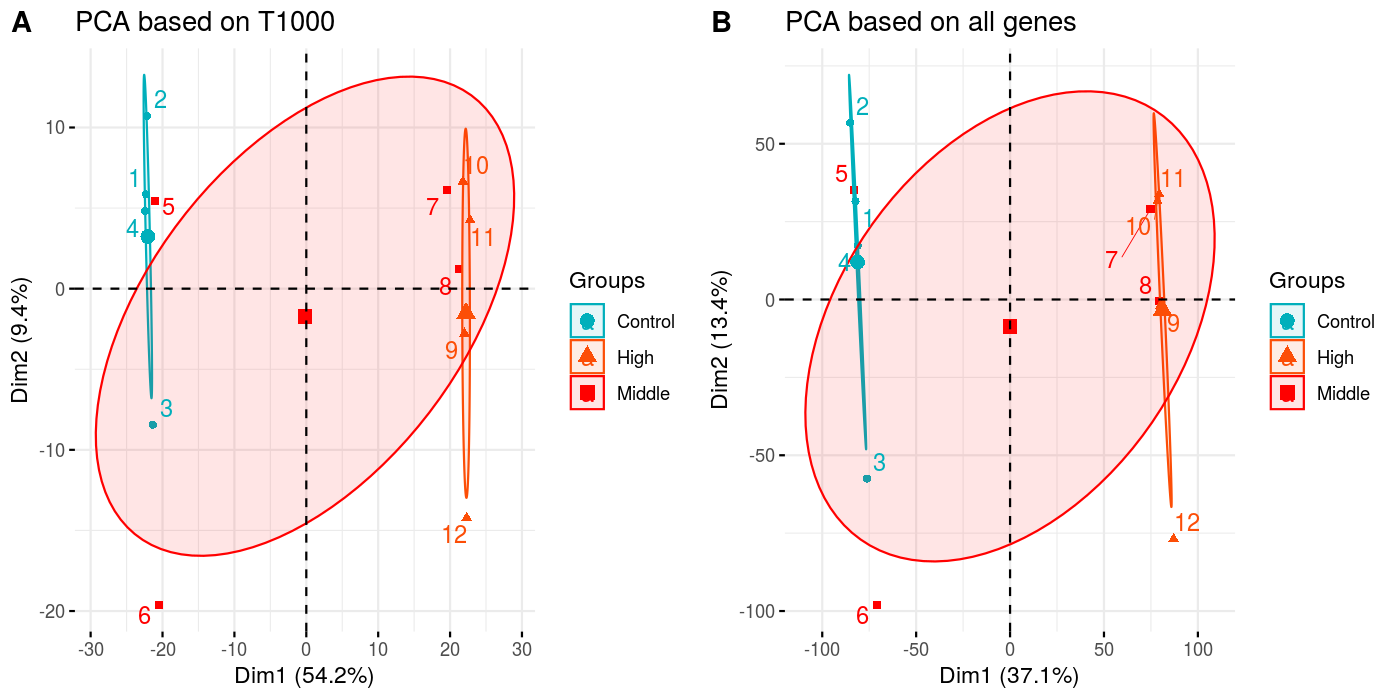

Supplement: Figure S5 [file peerj-07-7975-s005.zip › Supplementary_Figures_S5/fenofibrate.Human.in_vitro.Liver.tiff]

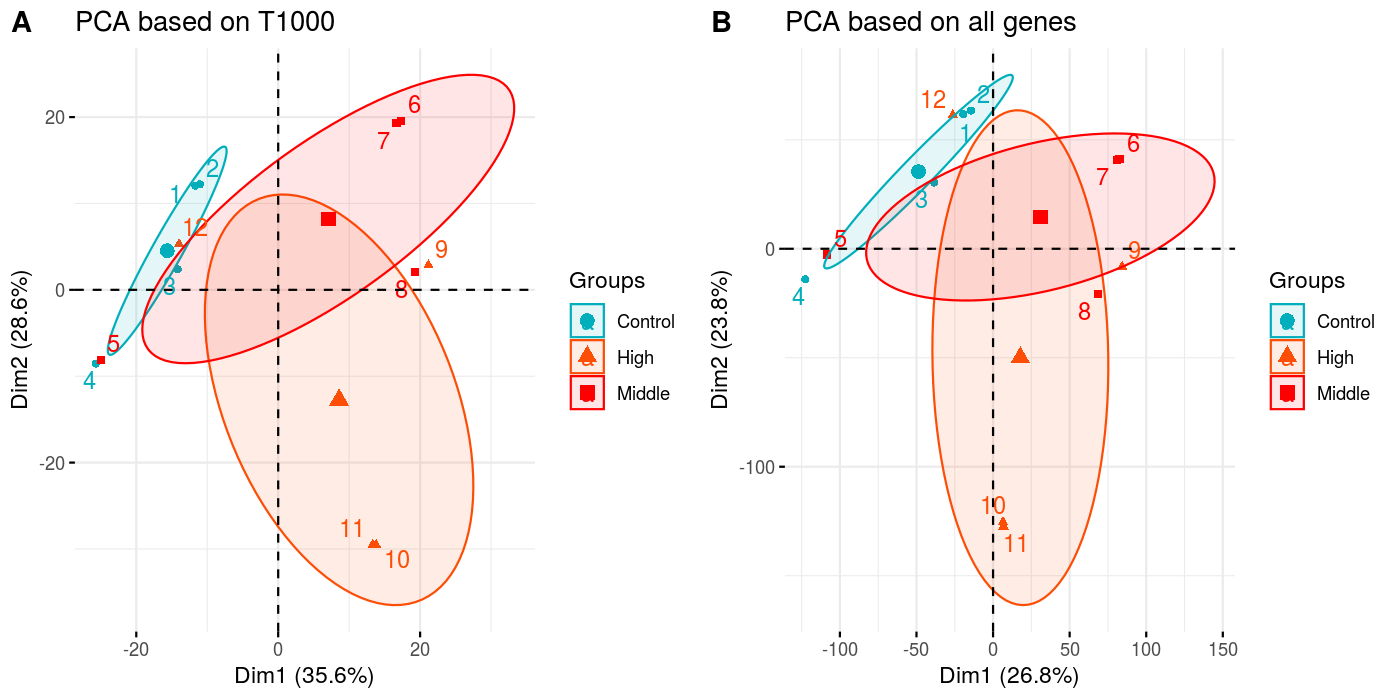

Supplement: Figure S5 [file peerj-07-7975-s005.zip › Supplementary_Figures_S5/caffeine.Human.in_vitro.Liver.tiff]

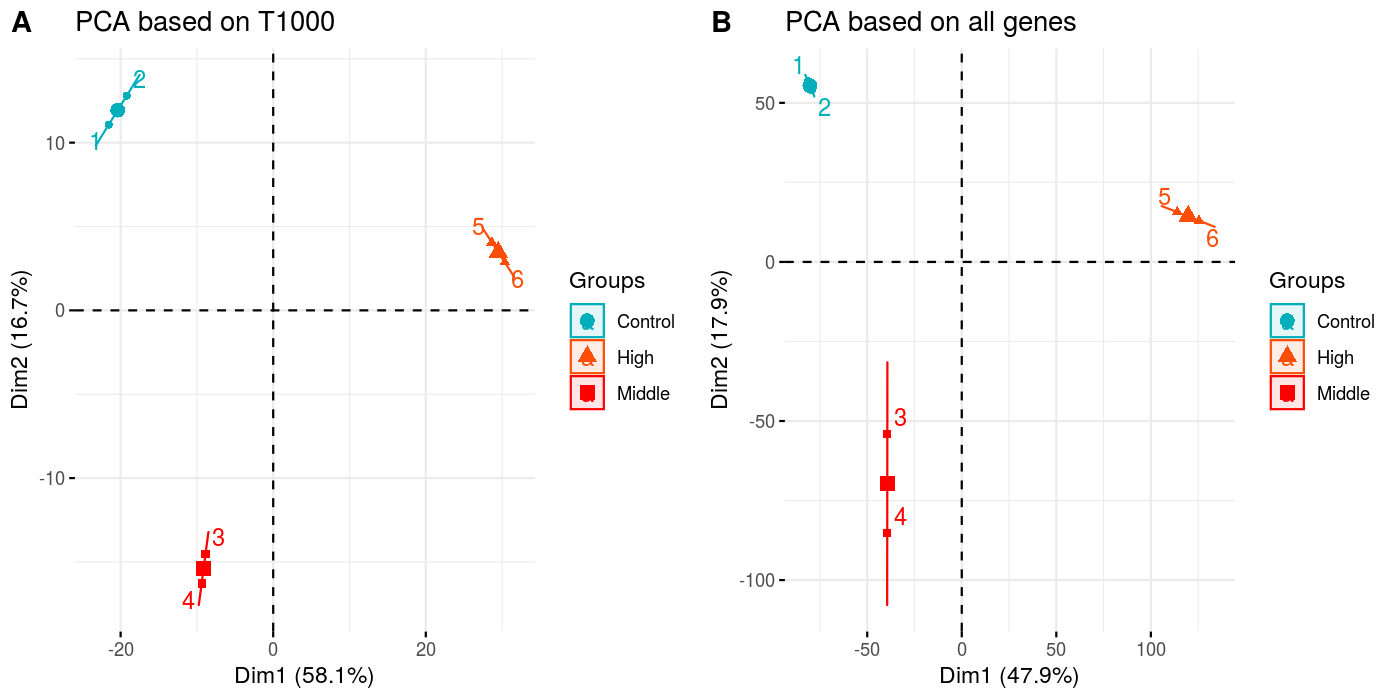

Supplement: Figure S5 [file peerj-07-7975-s005.zip › Supplementary_Figures_S5/venlafaxine.Human.in_vitro.Liver.tiff]

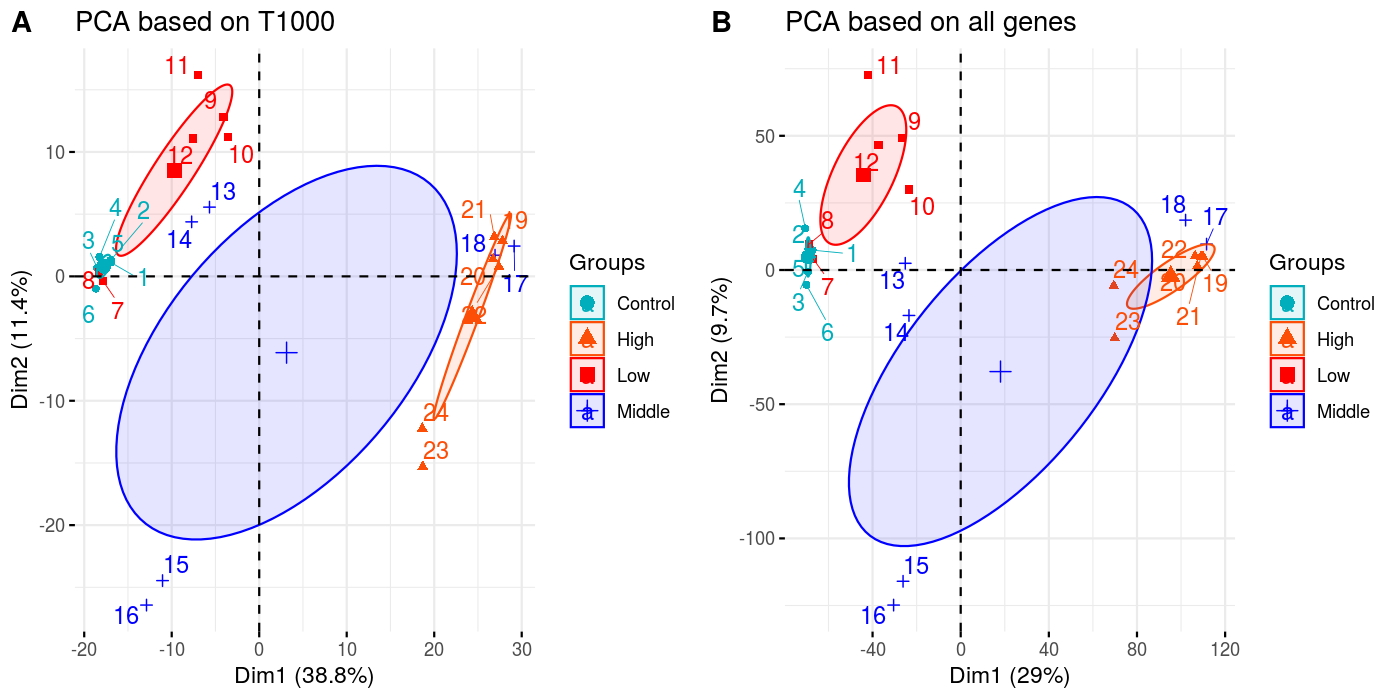

Supplement: Figure S5 [file peerj-07-7975-s005.zip › Supplementary_Figures_S5/labetalol.Human.in_vitro.Liver.tiff]

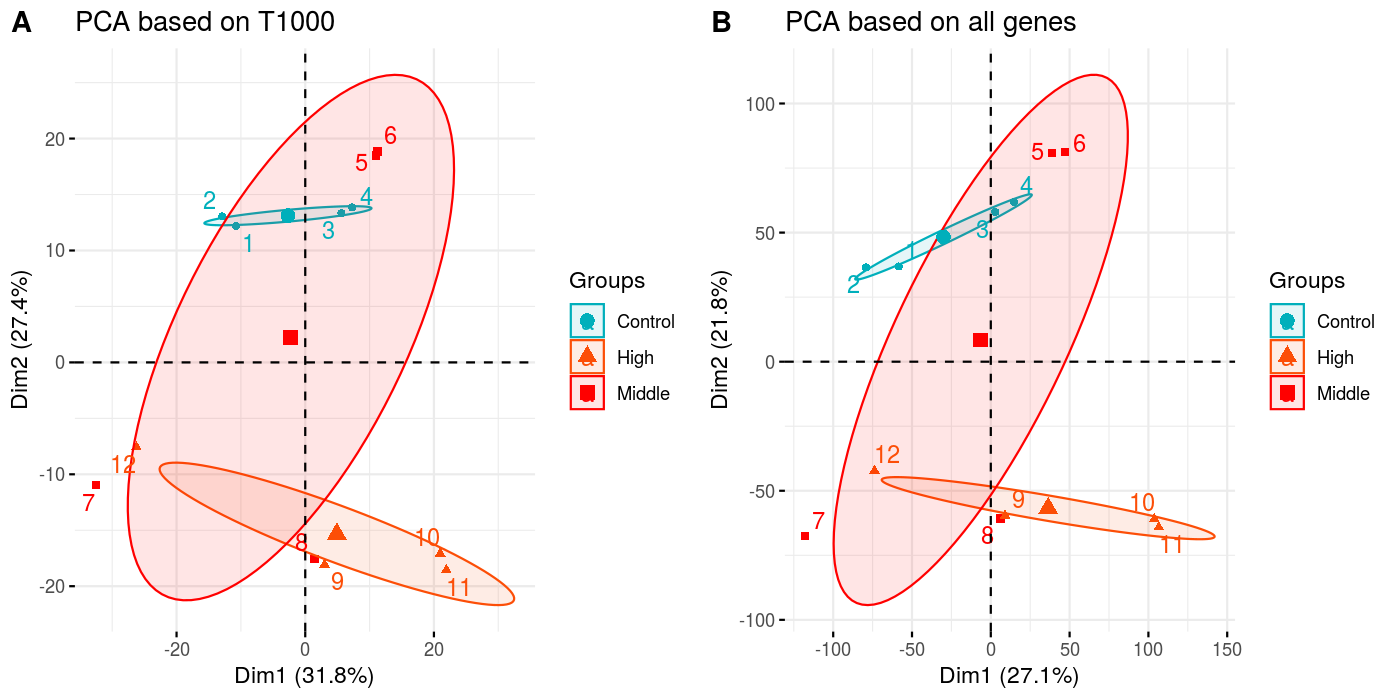

Supplement: Figure S5 [file peerj-07-7975-s005.zip › Supplementary_Figures_S5/colchicine.Human.in_vitro.Liver.tiff]

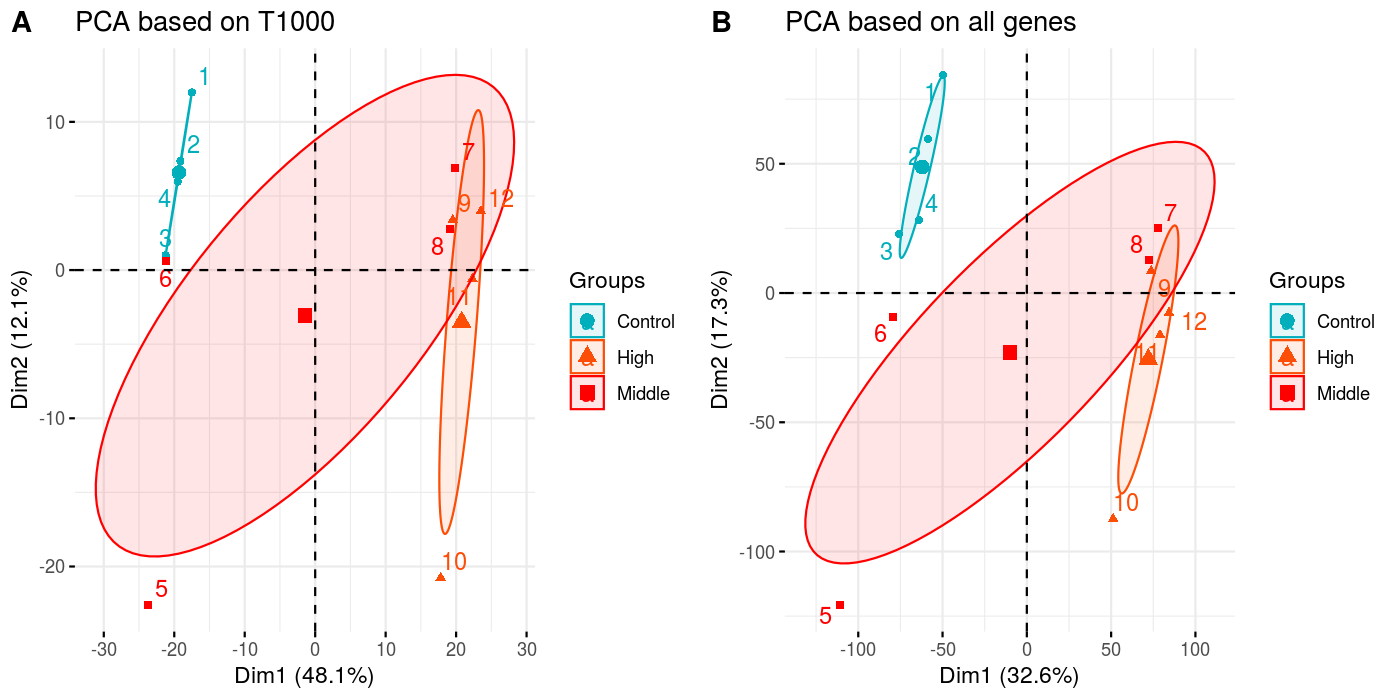

Supplement: Figure S5 [file peerj-07-7975-s005.zip › Supplementary_Figures_S5/nicotinic_acid.Human.in_vitro.Liver.tiff]

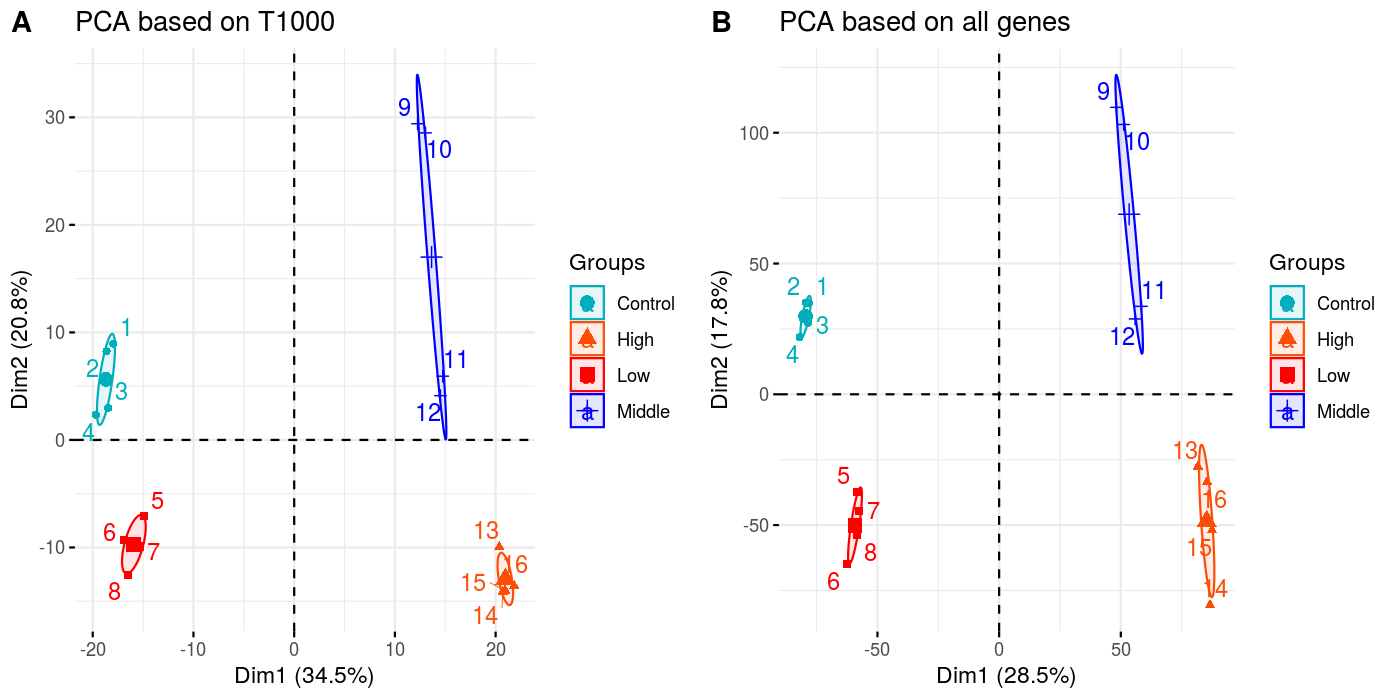

Supplement: Figure S5 [file peerj-07-7975-s005.zip › Supplementary_Figures_S5/phalloidin.Human.in_vitro.Liver.tiff]

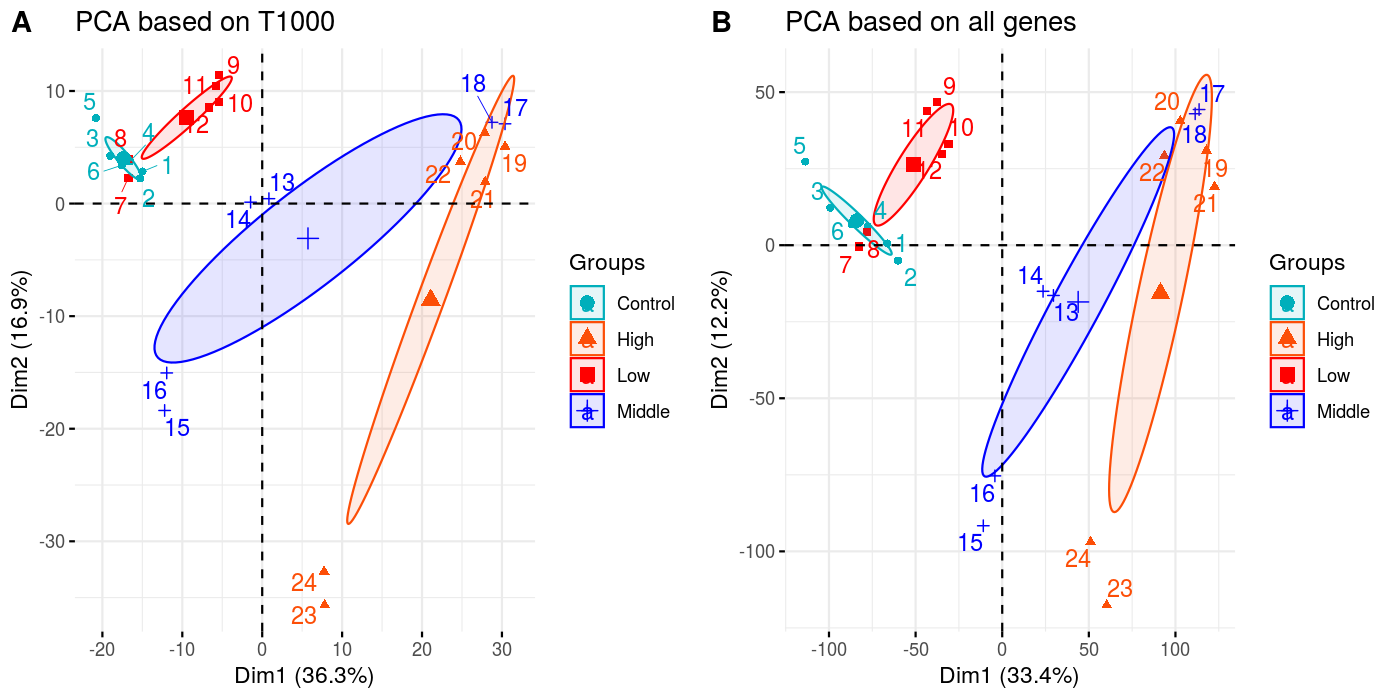

Supplement: Figure S5 [file peerj-07-7975-s005.zip › Supplementary_Figures_S5/naphthyl_isothiocyanate.Human.in_vitro.Liver.tiff]

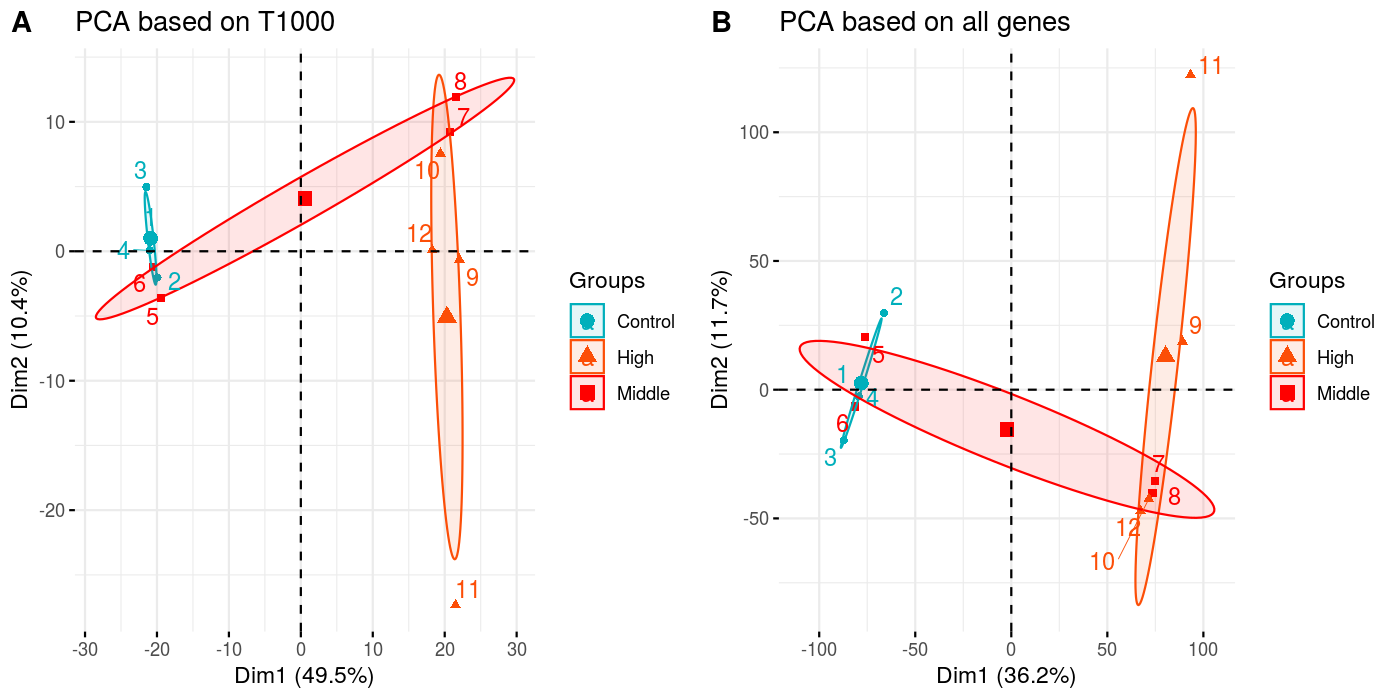

Supplement: Figure S5 [file peerj-07-7975-s005.zip › Supplementary_Figures_S5/chlormezanone.Human.in_vitro.Liver.tiff]

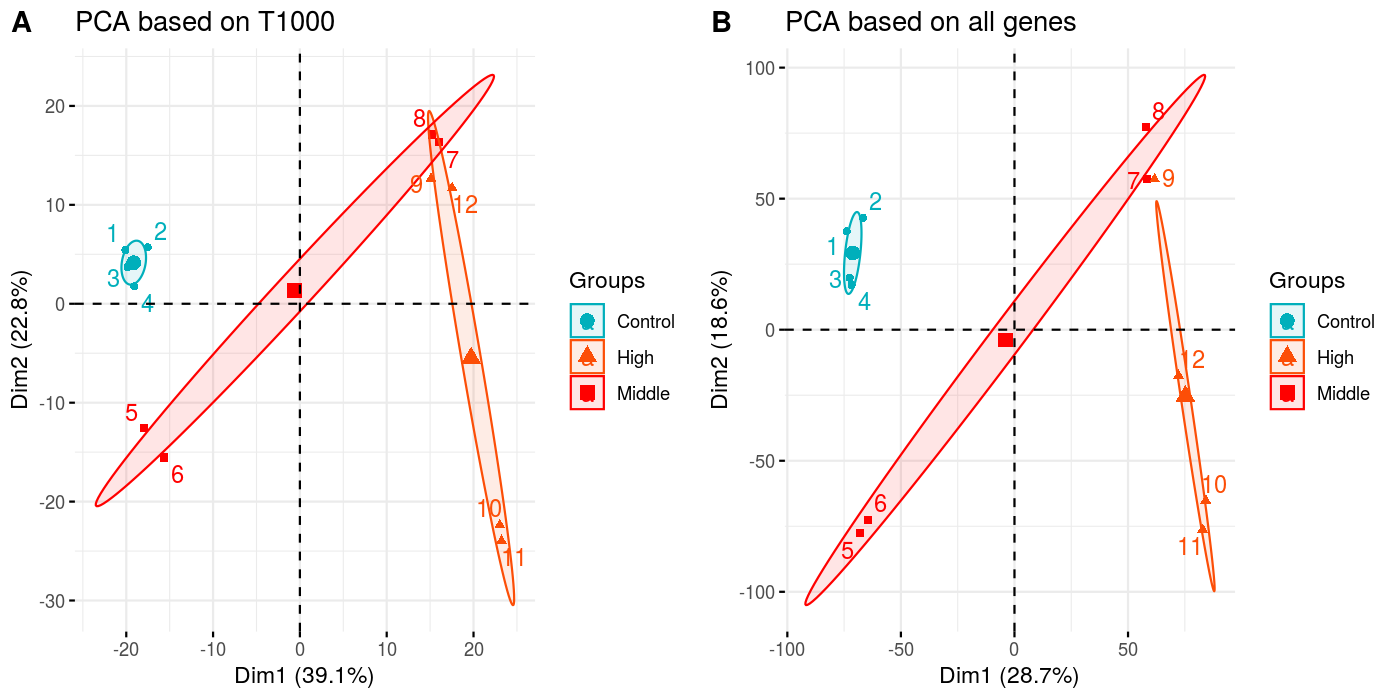

Supplement: Figure S5 [file peerj-07-7975-s005.zip › Supplementary_Figures_S5/promethazine.Human.in_vitro.Liver.tiff]

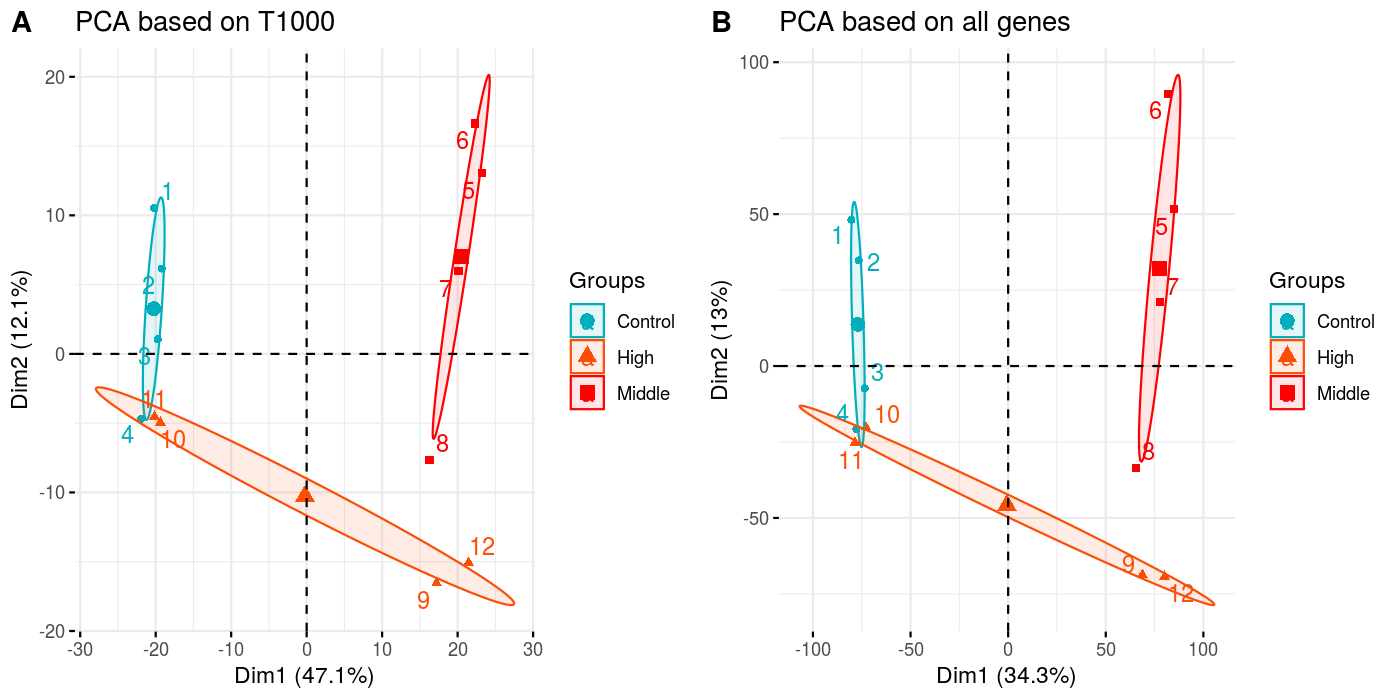

Supplement: Figure S5 [file peerj-07-7975-s005.zip › Supplementary_Figures_S5/acarbose.Human.in_vitro.Liver.tiff]

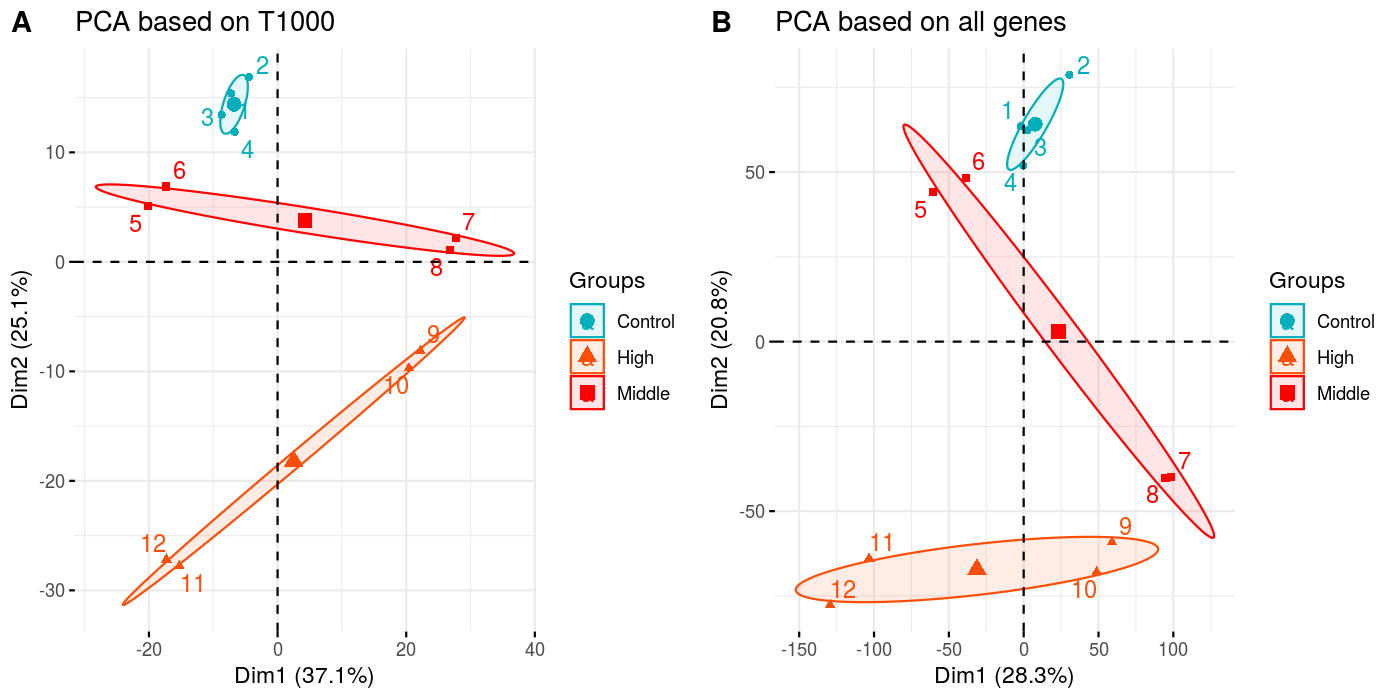

Supplement: Figure S5 [file peerj-07-7975-s005.zip › Supplementary_Figures_S5/metformin.Human.in_vitro.Liver.tiff]

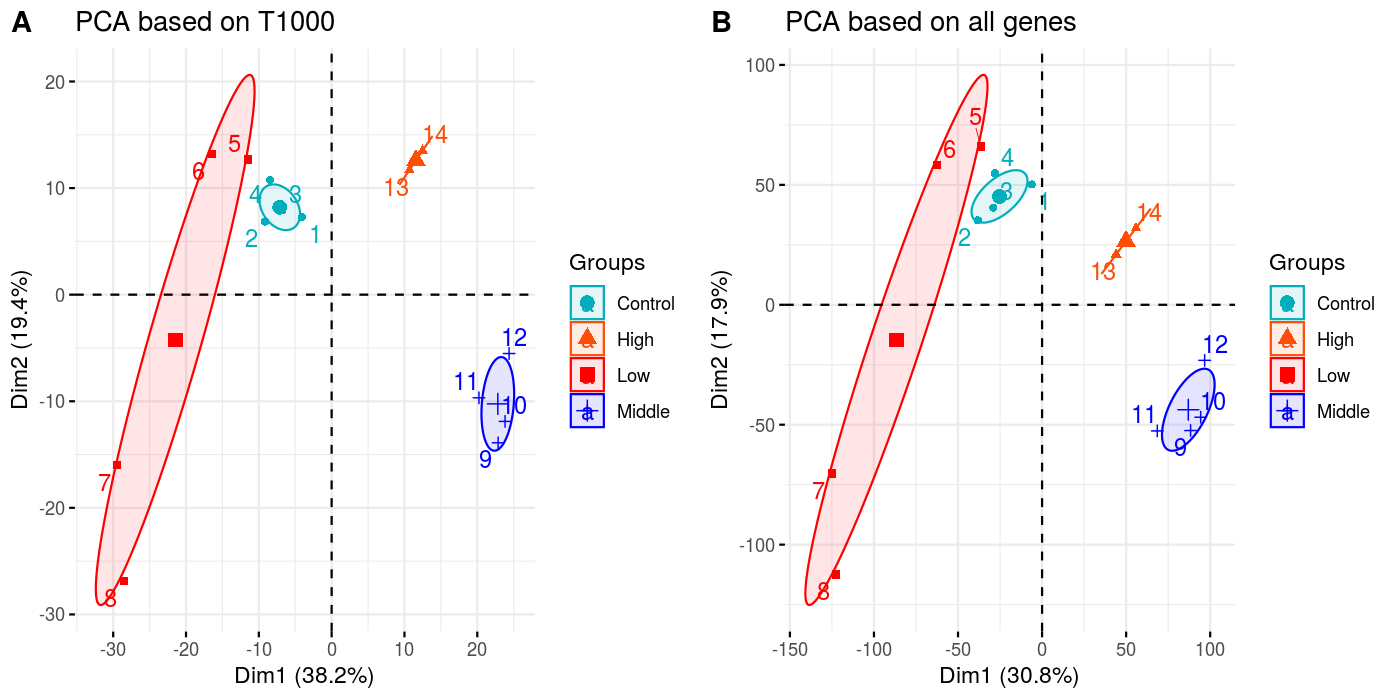

Supplement: Figure S5 [file peerj-07-7975-s005.zip › Supplementary_Figures_S5/phorone.Human.in_vitro.Liver.tiff]

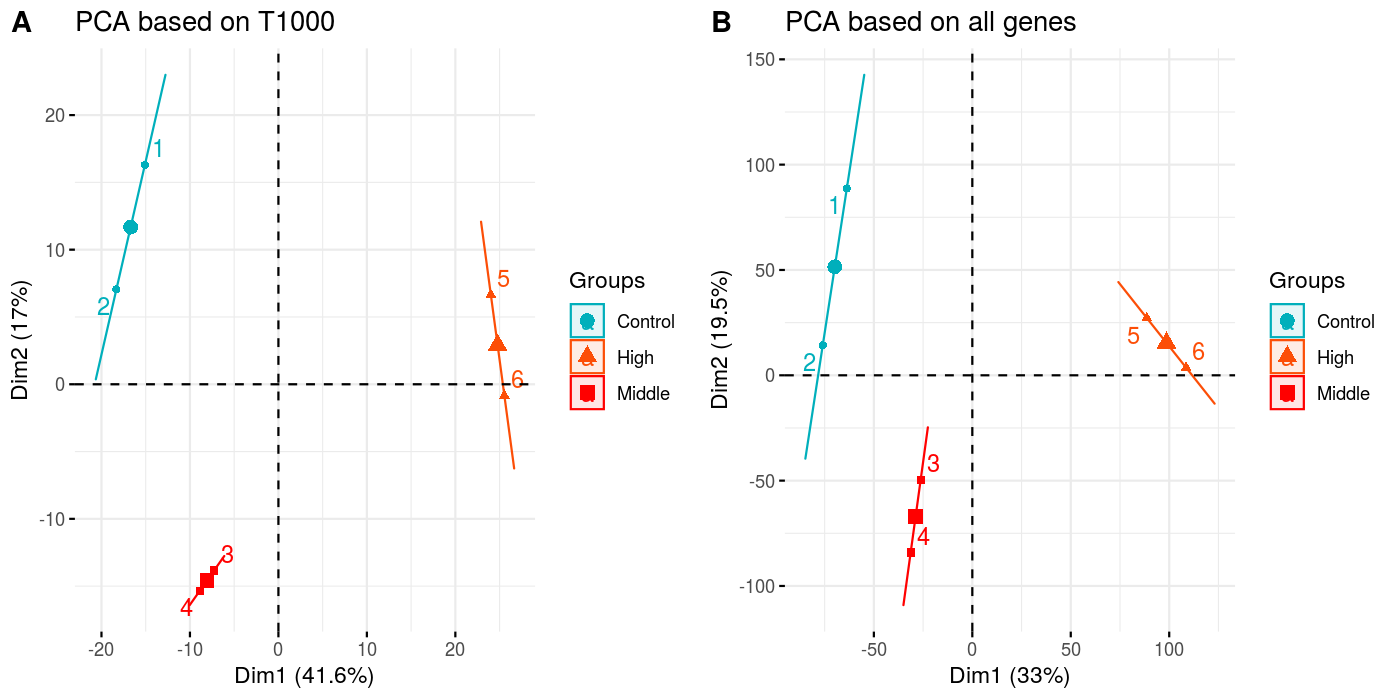

Supplement: Figure S5 [file peerj-07-7975-s005.zip › Supplementary_Figures_S5/alpidem.Human.in_vitro.Liver.tiff]

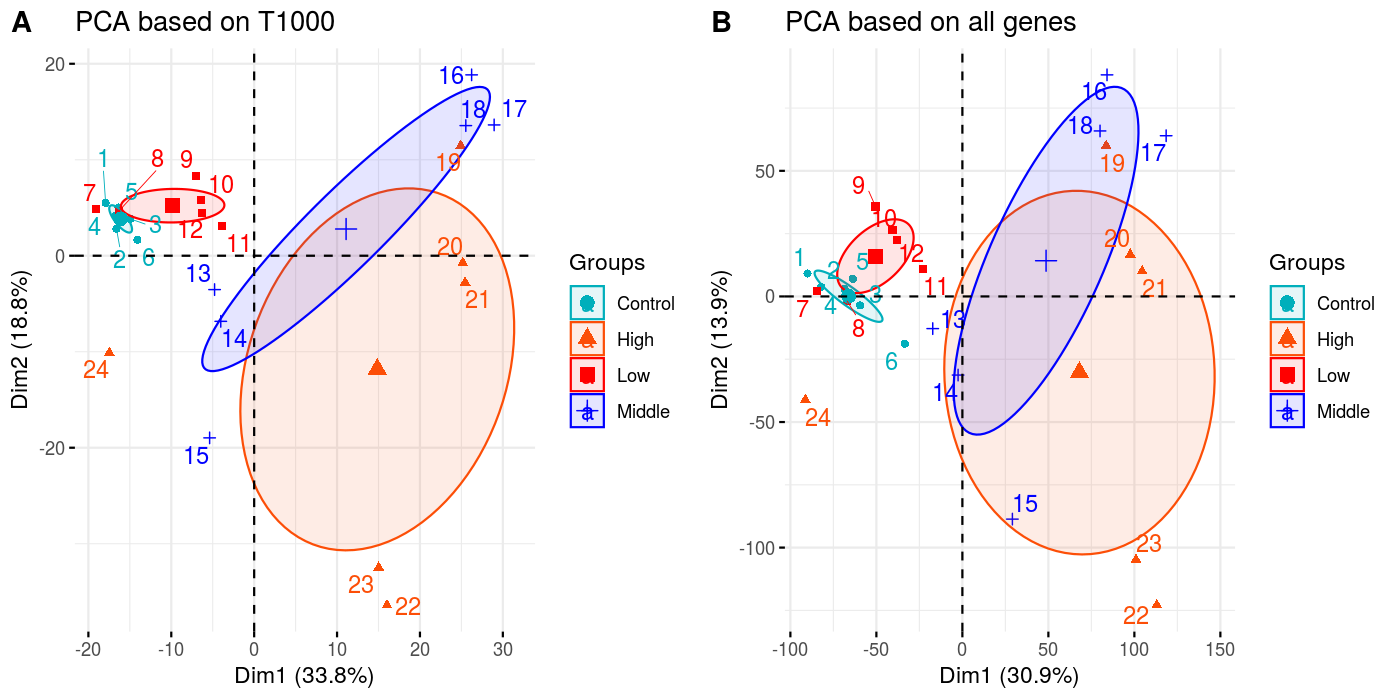

Supplement: Figure S5 [file peerj-07-7975-s005.zip › Supplementary_Figures_S5/diazepam.Human.in_vitro.Liver.tiff]

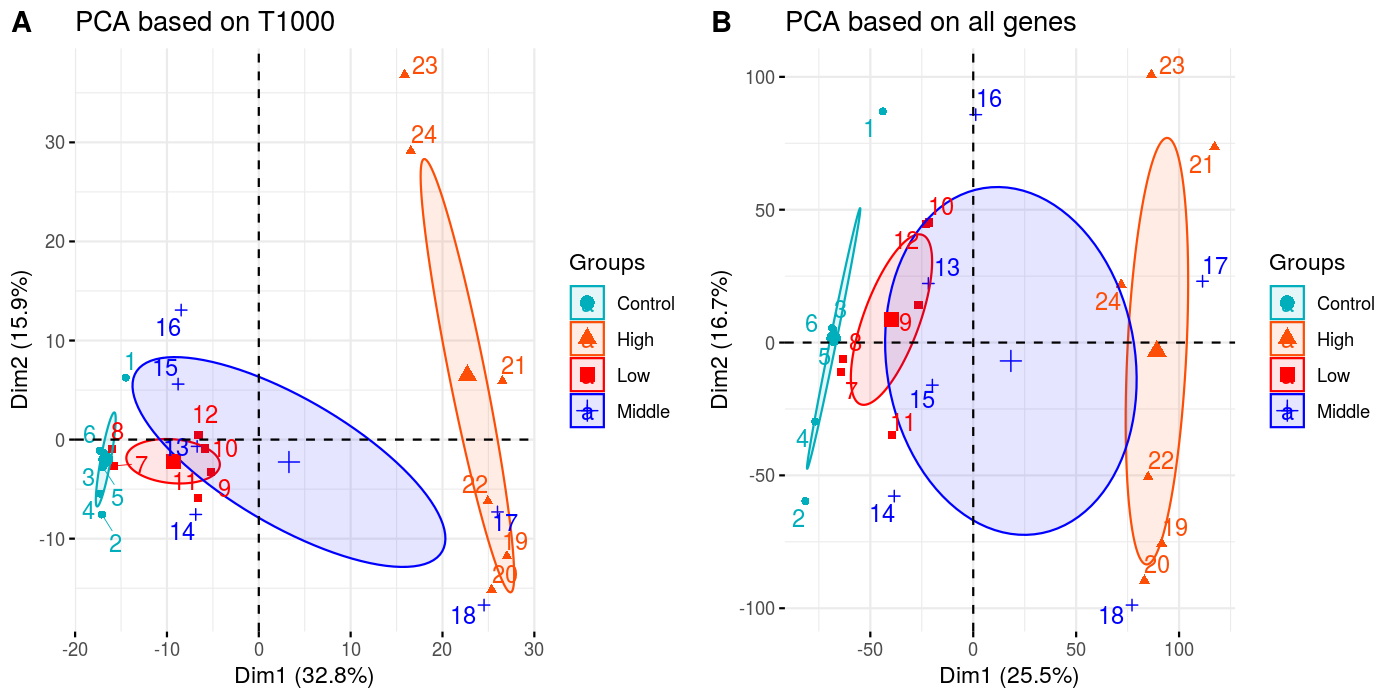

Supplement: Figure S5 [file peerj-07-7975-s005.zip › Supplementary_Figures_S5/ketoconazole.Human.in_vitro.Liver.tiff]

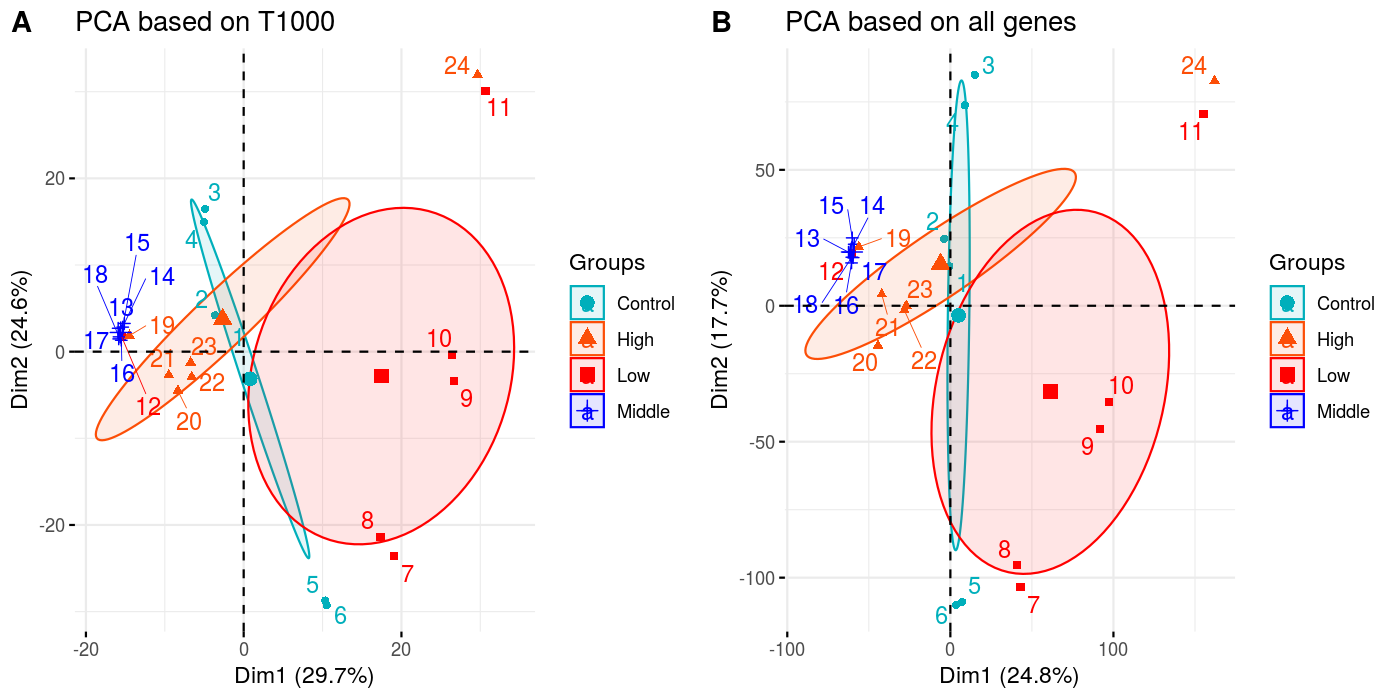

Supplement: Figure S5 [file peerj-07-7975-s005.zip › Supplementary_Figures_S5/ethionine.Human.in_vitro.Liver.tiff]

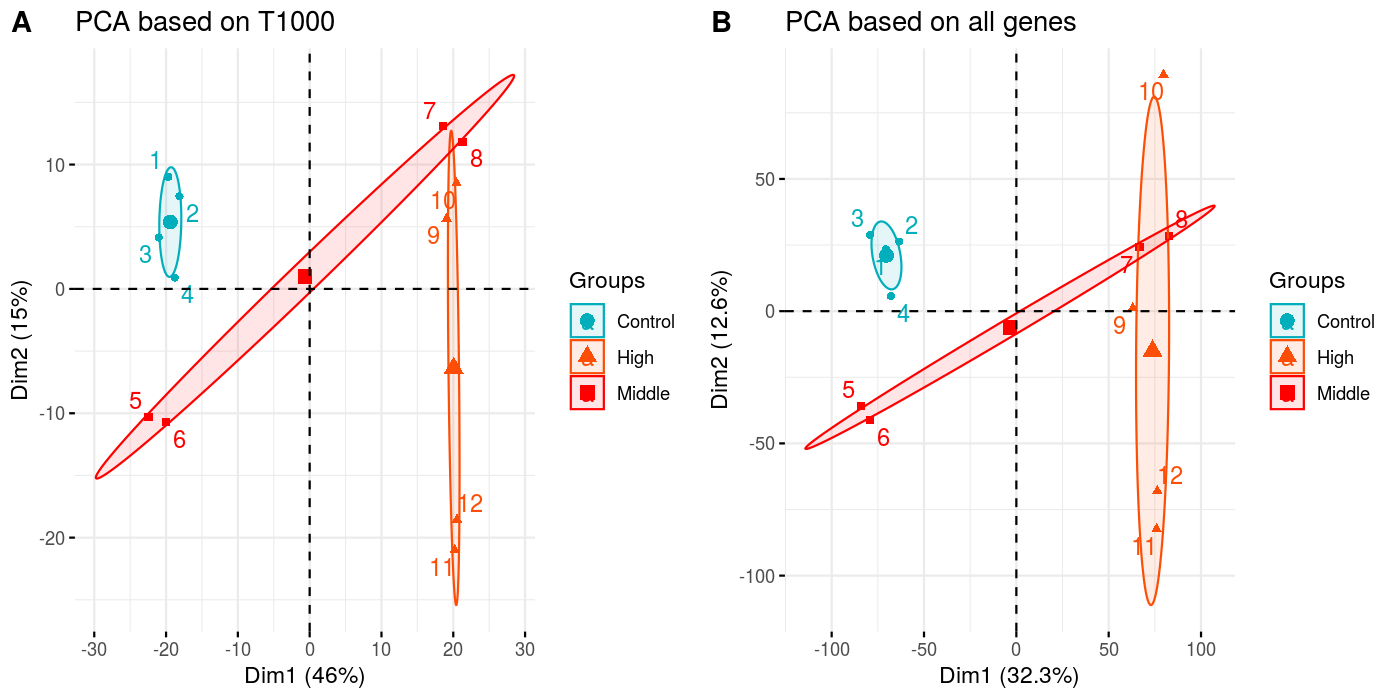

Supplement: Figure S5 [file peerj-07-7975-s005.zip › Supplementary_Figures_S5/quinidine.Human.in_vitro.Liver.tiff]

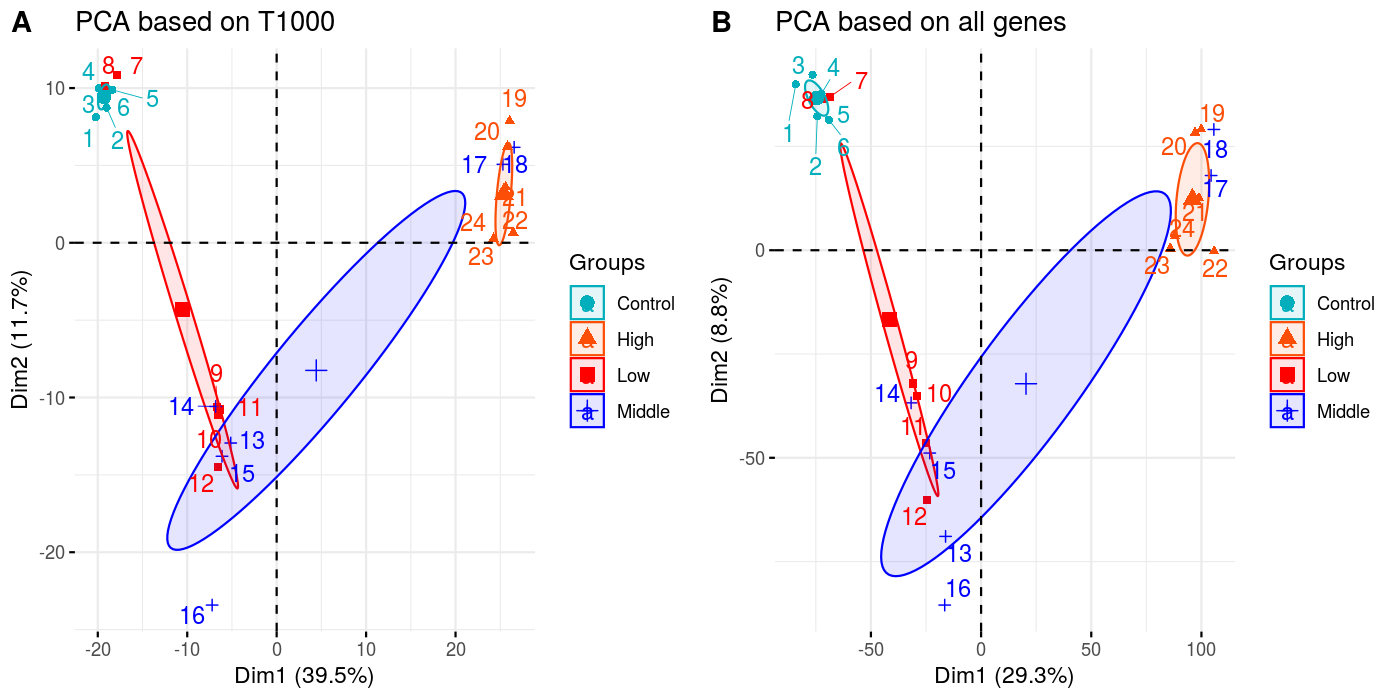

Supplement: Figure S5 [file peerj-07-7975-s005.zip › Supplementary_Figures_S5/cyclophosphamide.Human.in_vitro.Liver.tiff]

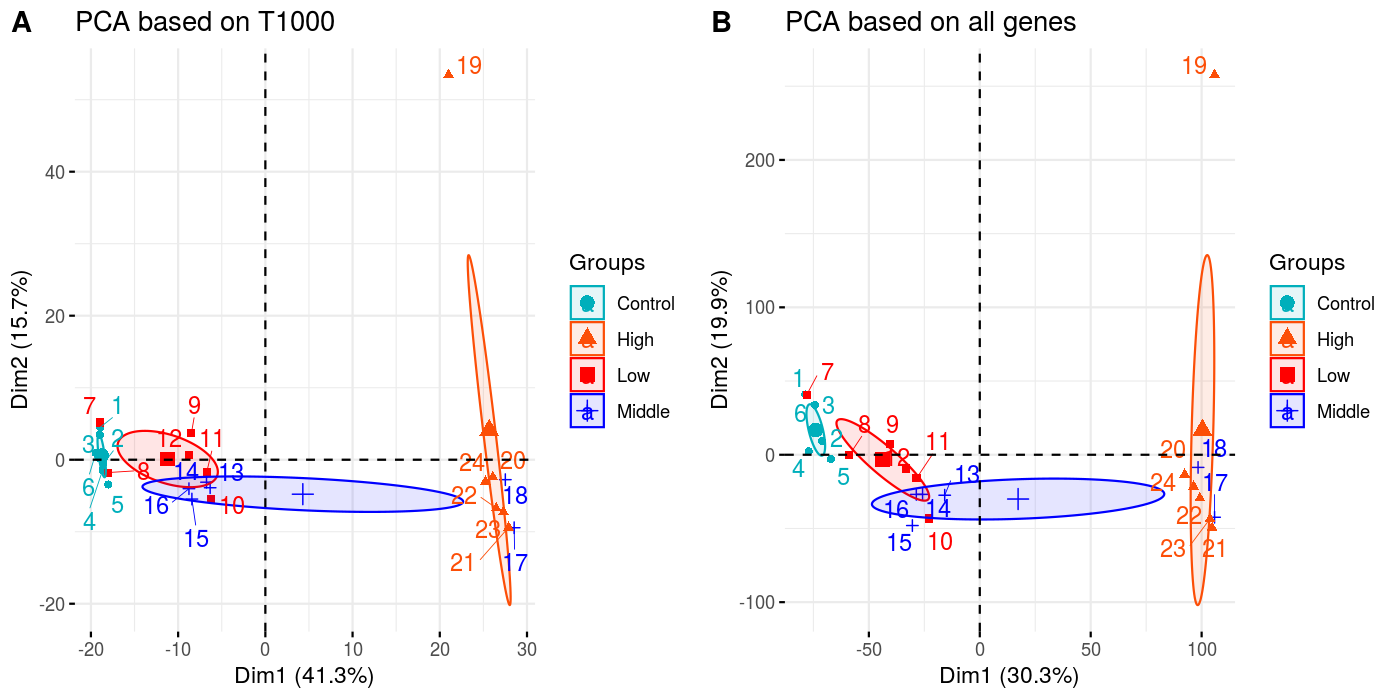

Supplement: Figure S5 [file peerj-07-7975-s005.zip › Supplementary_Figures_S5/carbon_tetrachloride.Human.in_vitro.Liver.tiff]

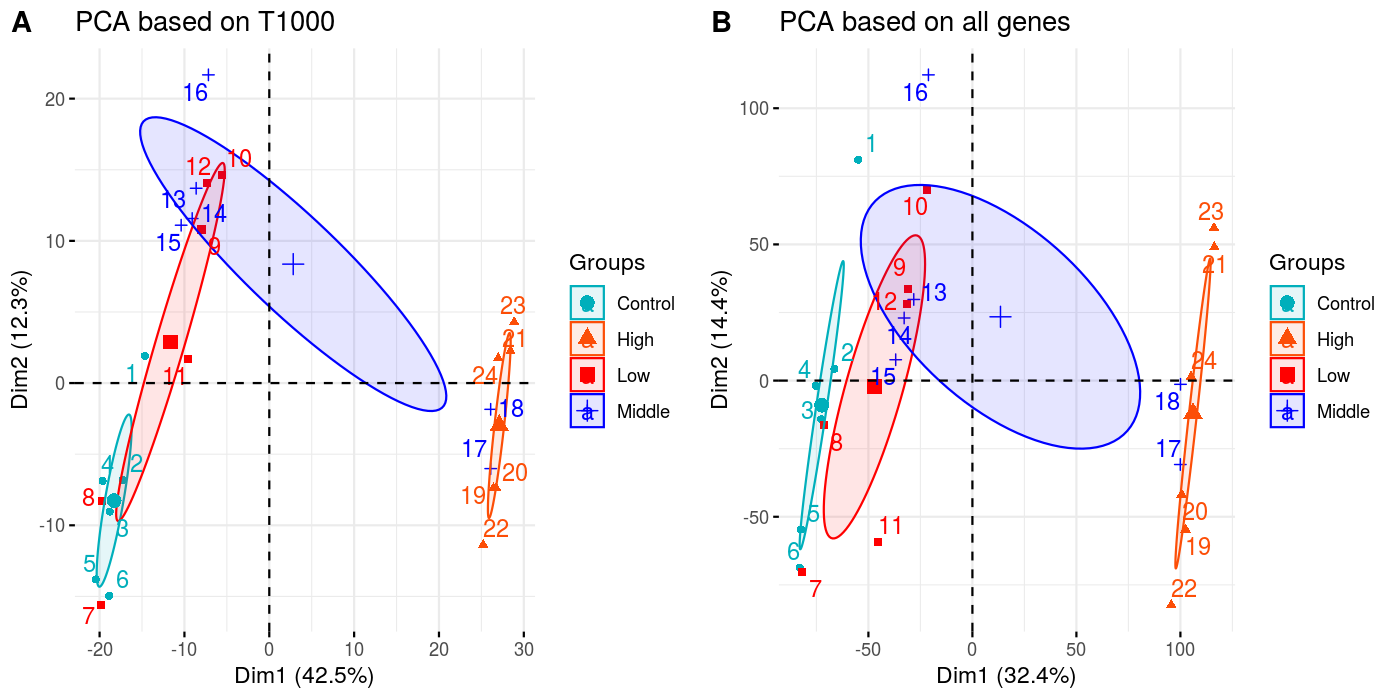

Supplement: Figure S5 [file peerj-07-7975-s005.zip › Supplementary_Figures_S5/methyltestosterone.Human.in_vitro.Liver.tiff]

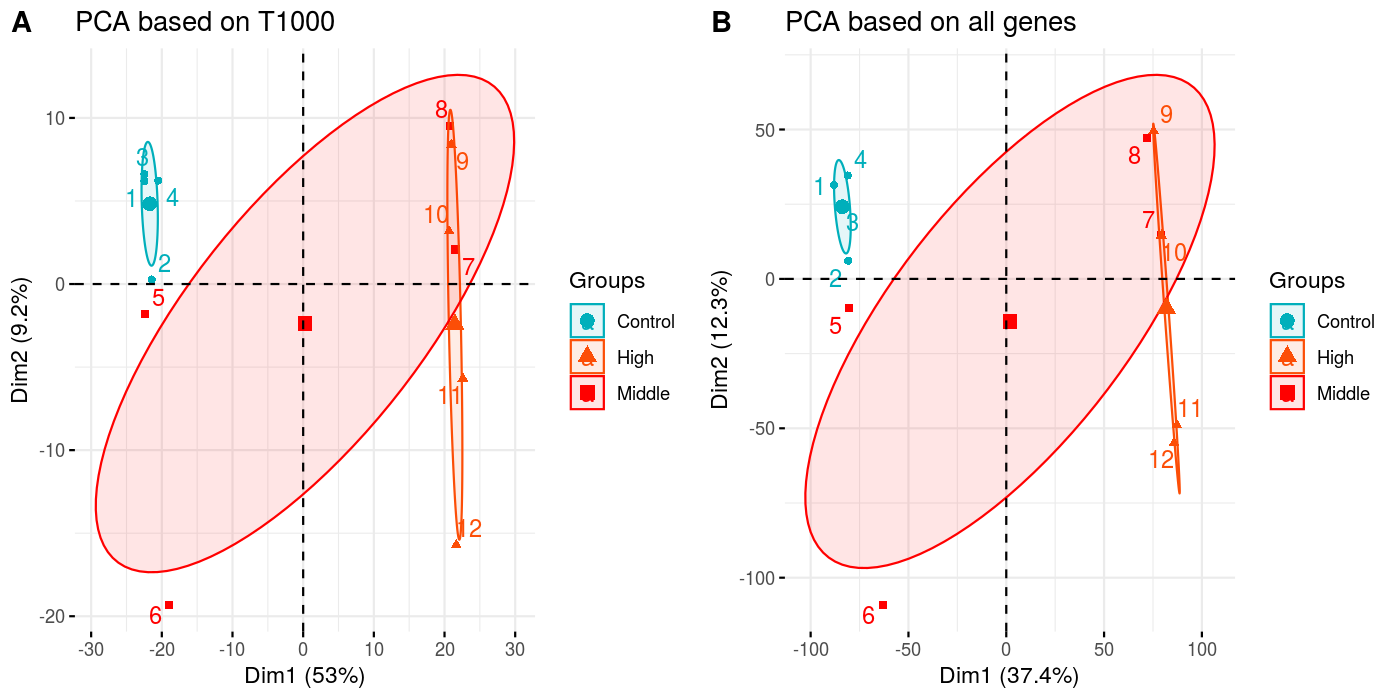

Supplement: Figure S5 [file peerj-07-7975-s005.zip › Supplementary_Figures_S5/ciprofloxacin.Human.in_vitro.Liver.tiff]

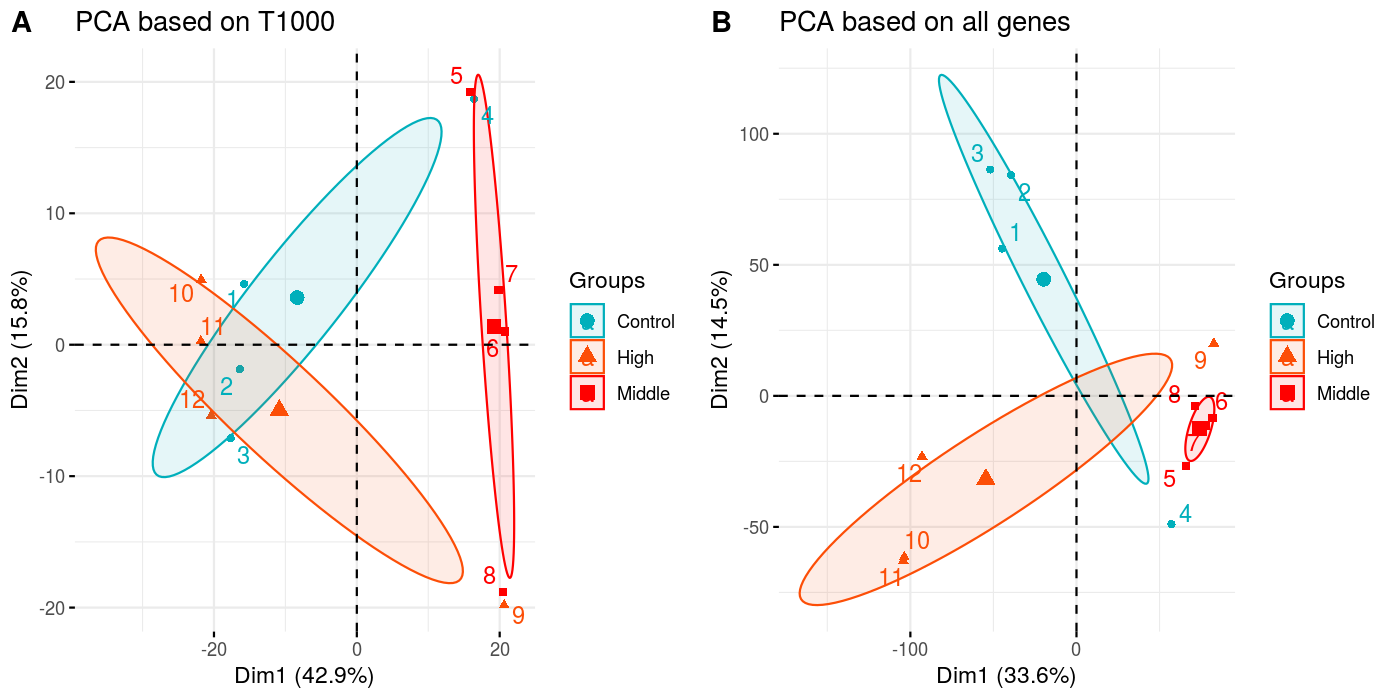

Supplement: Figure S5 [file peerj-07-7975-s005.zip › Supplementary_Figures_S5/tolbutamide.Human.in_vitro.Liver.tiff]

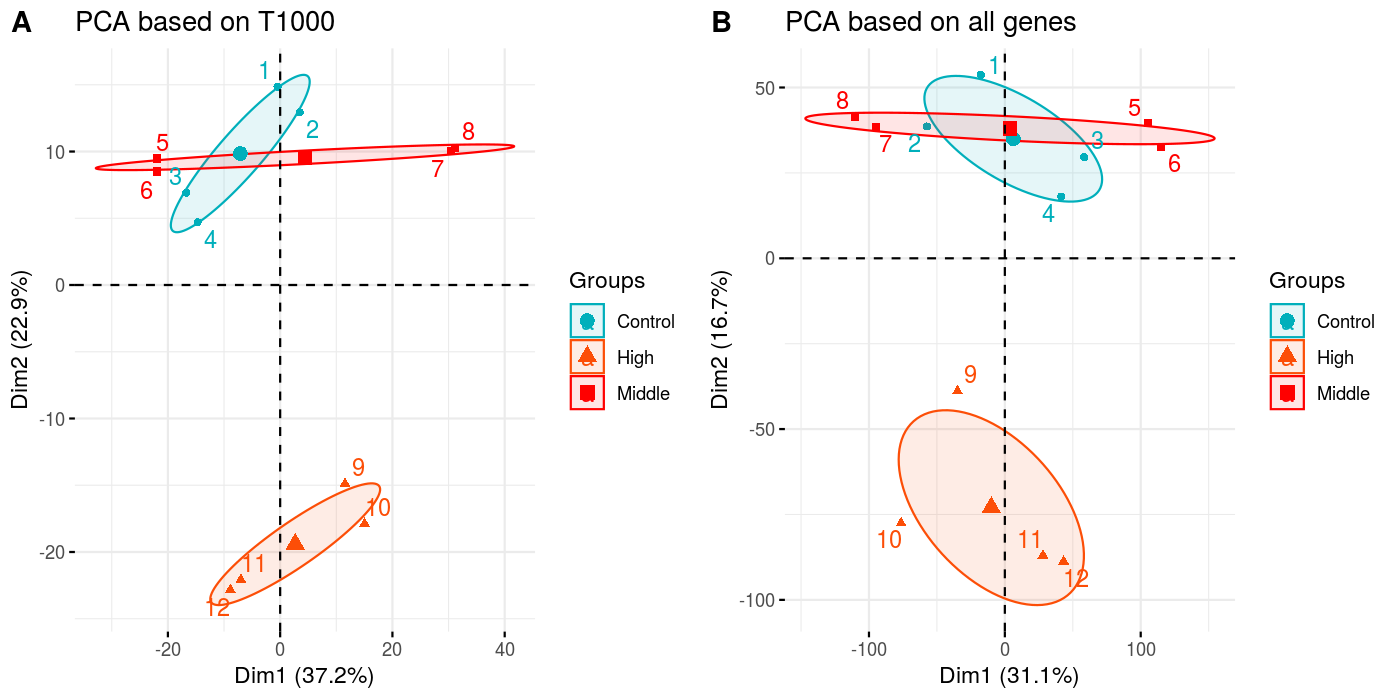

Supplement: Figure S5 [file peerj-07-7975-s005.zip › Supplementary_Figures_S5/furosemide.Human.in_vitro.Liver.tiff]

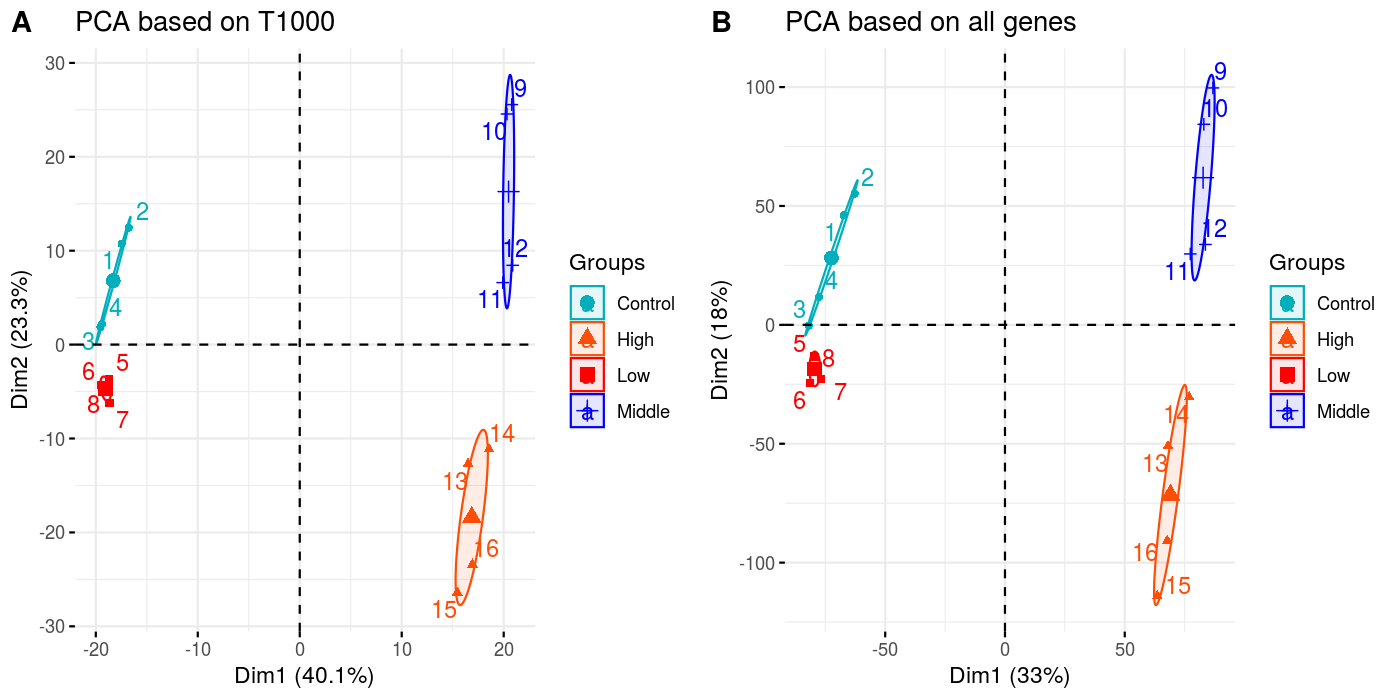

Supplement: Figure S5 [file peerj-07-7975-s005.zip › Supplementary_Figures_S5/TNFa.Human.in_vitro.Liver.tiff]

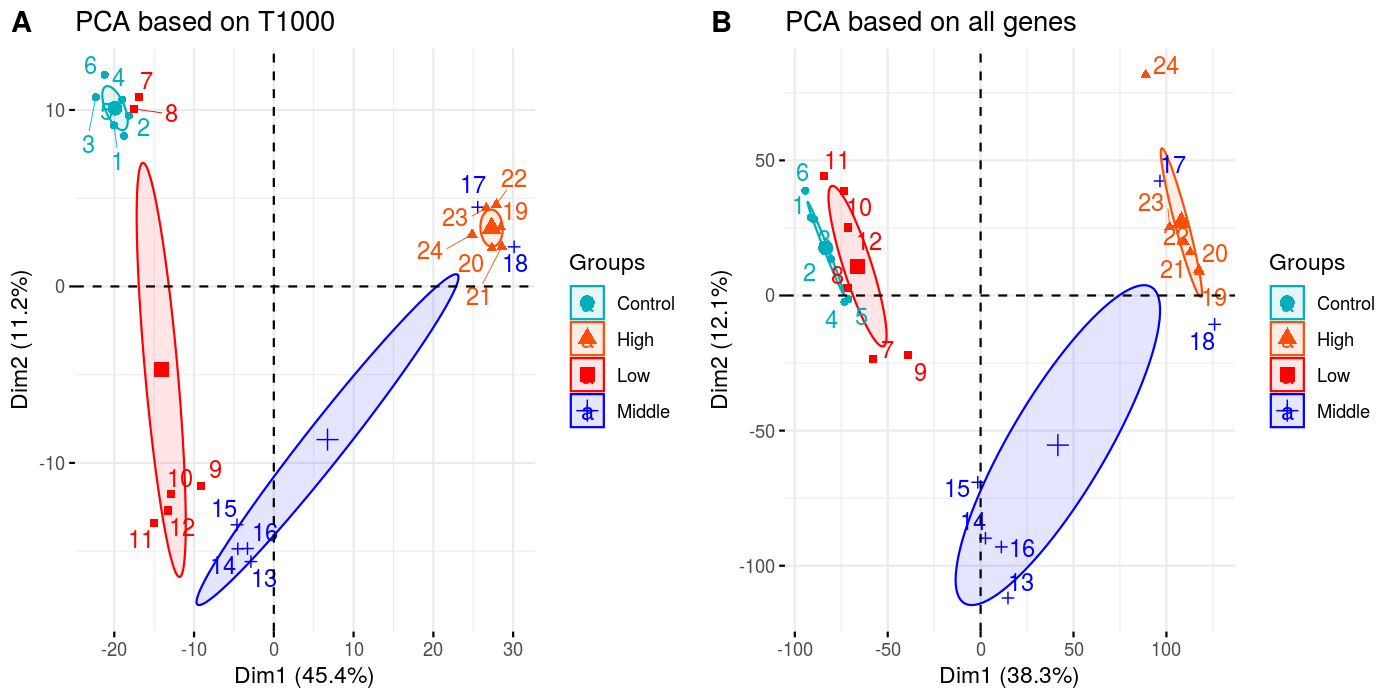

Supplement: Figure S5 [file peerj-07-7975-s005.zip › Supplementary_Figures_S5/gemfibrozil.Human.in_vitro.Liver.tiff]

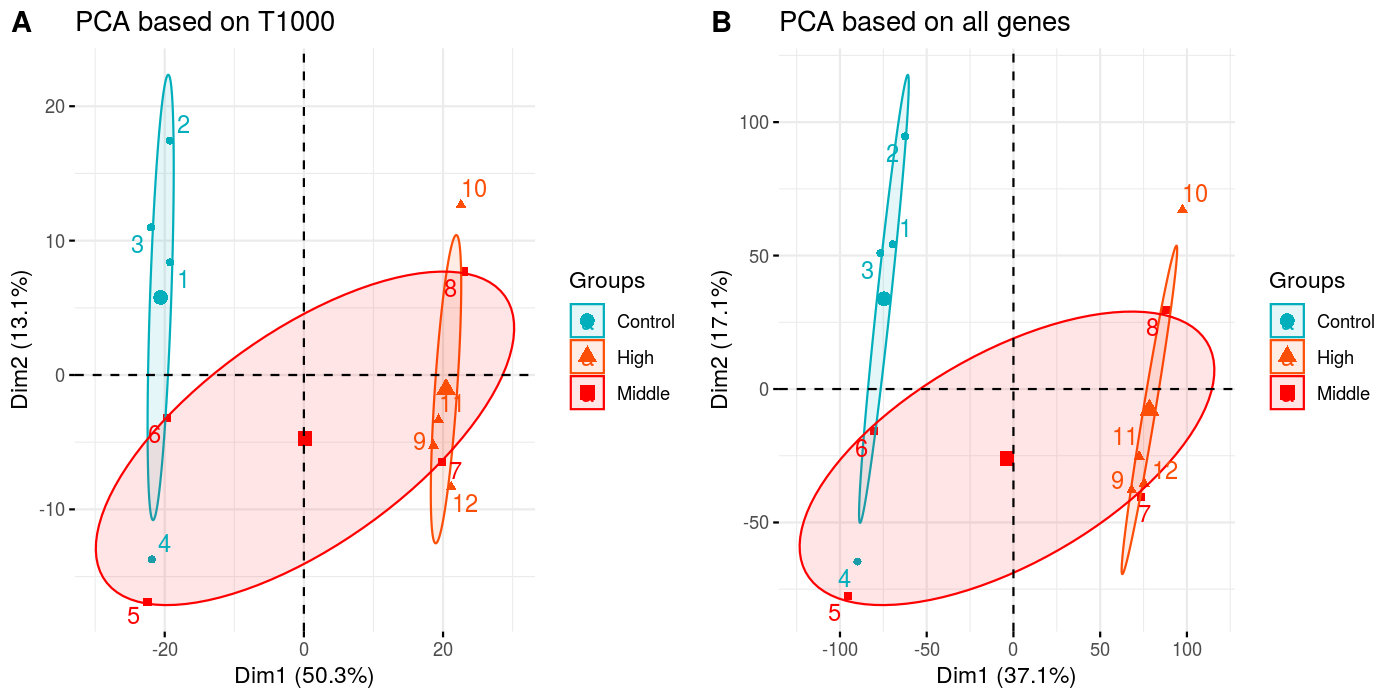

Supplement: Figure S5 [file peerj-07-7975-s005.zip › Supplementary_Figures_S5/lornoxicam.Human.in_vitro.Liver.tiff]

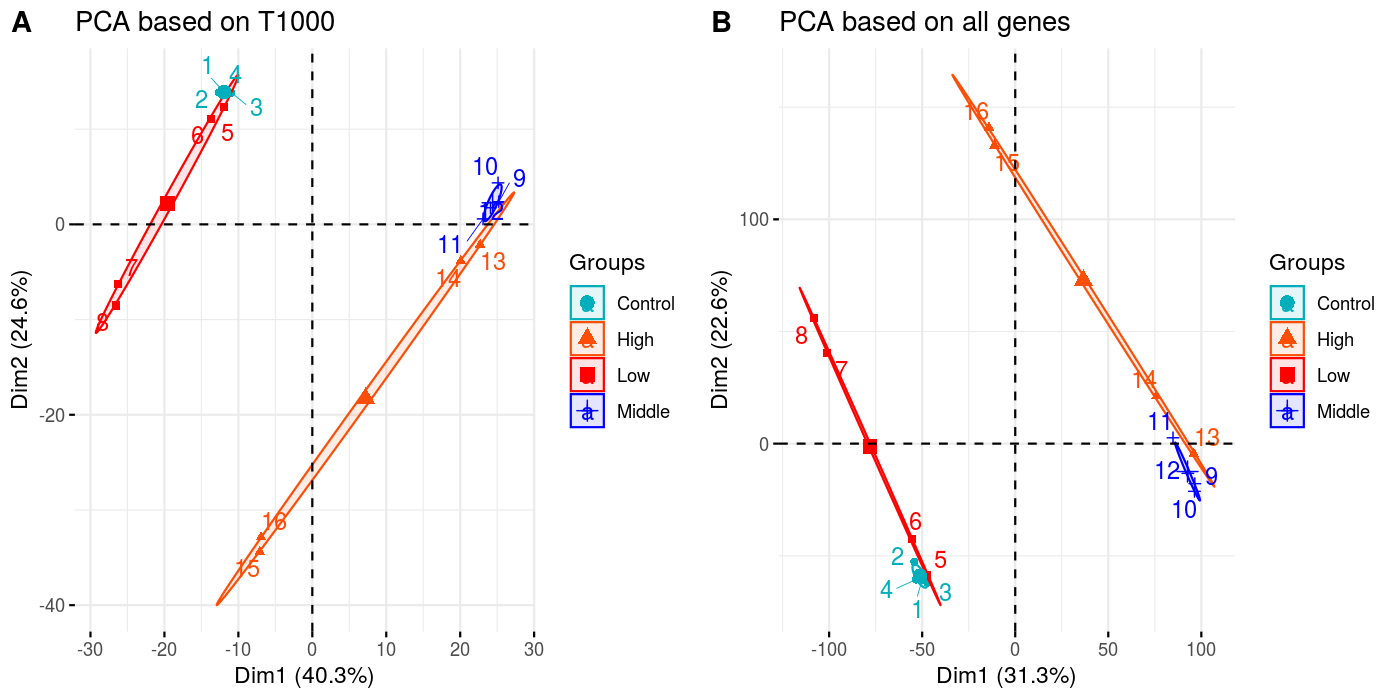

Supplement: Figure S5 [file peerj-07-7975-s005.zip › Supplementary_Figures_S5/2,4-dinitrophenol.Human.in_vitro.Liver.tiff]

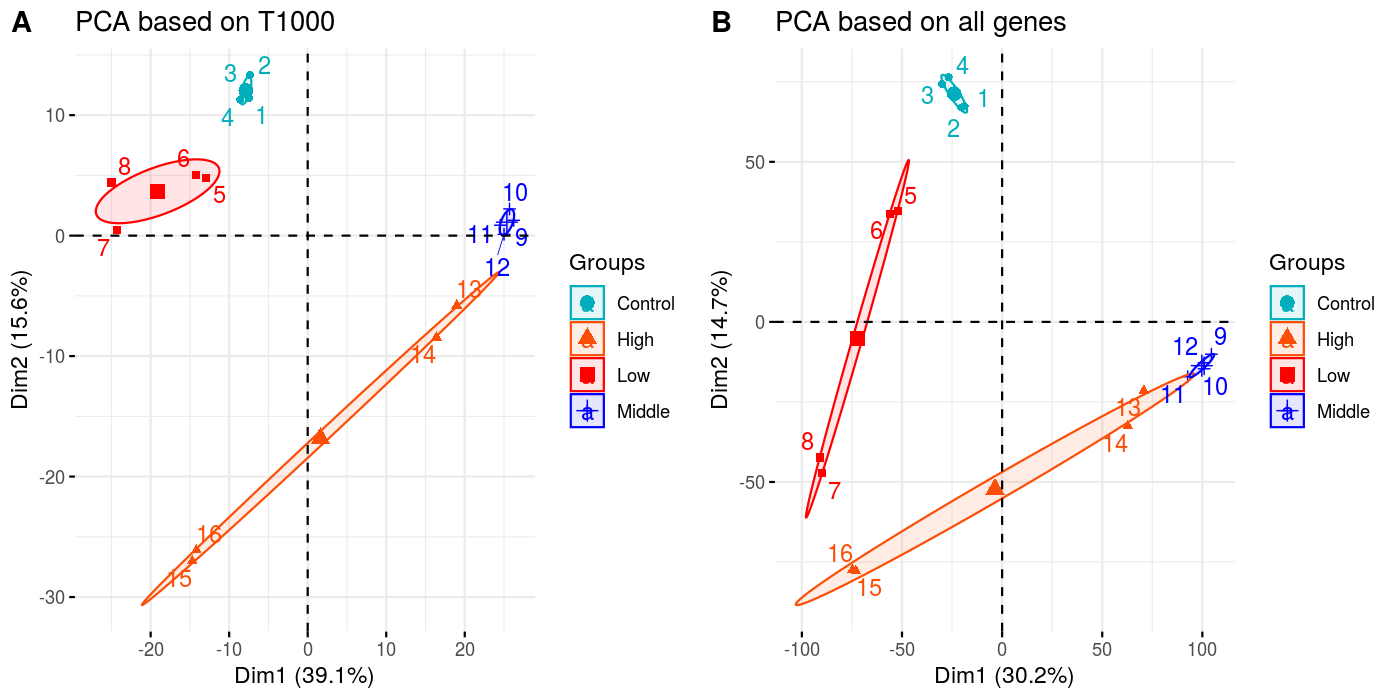

Supplement: Figure S5 [file peerj-07-7975-s005.zip › Supplementary_Figures_S5/diethyl_maleate.Human.in_vitro.Liver.tiff]

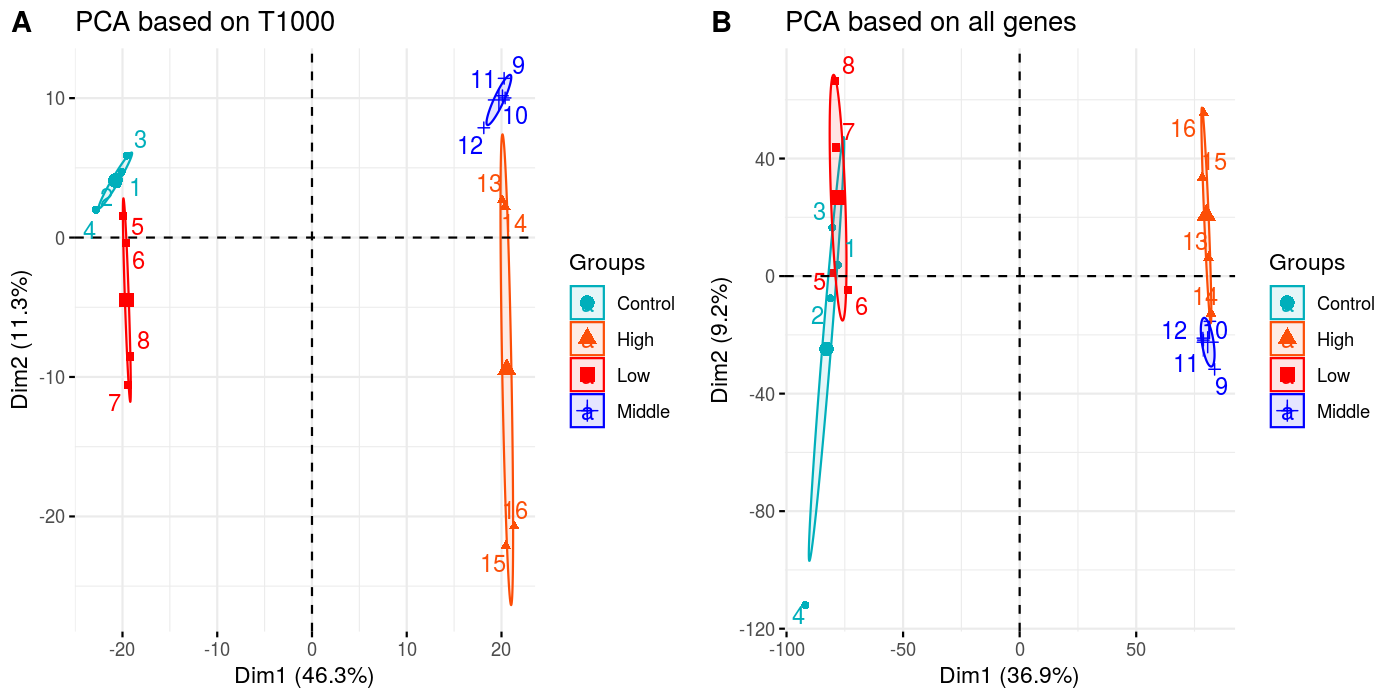

Supplement: Figure S5 [file peerj-07-7975-s005.zip › Supplementary_Figures_S5/bendazac.Human.in_vitro.Liver.tiff]

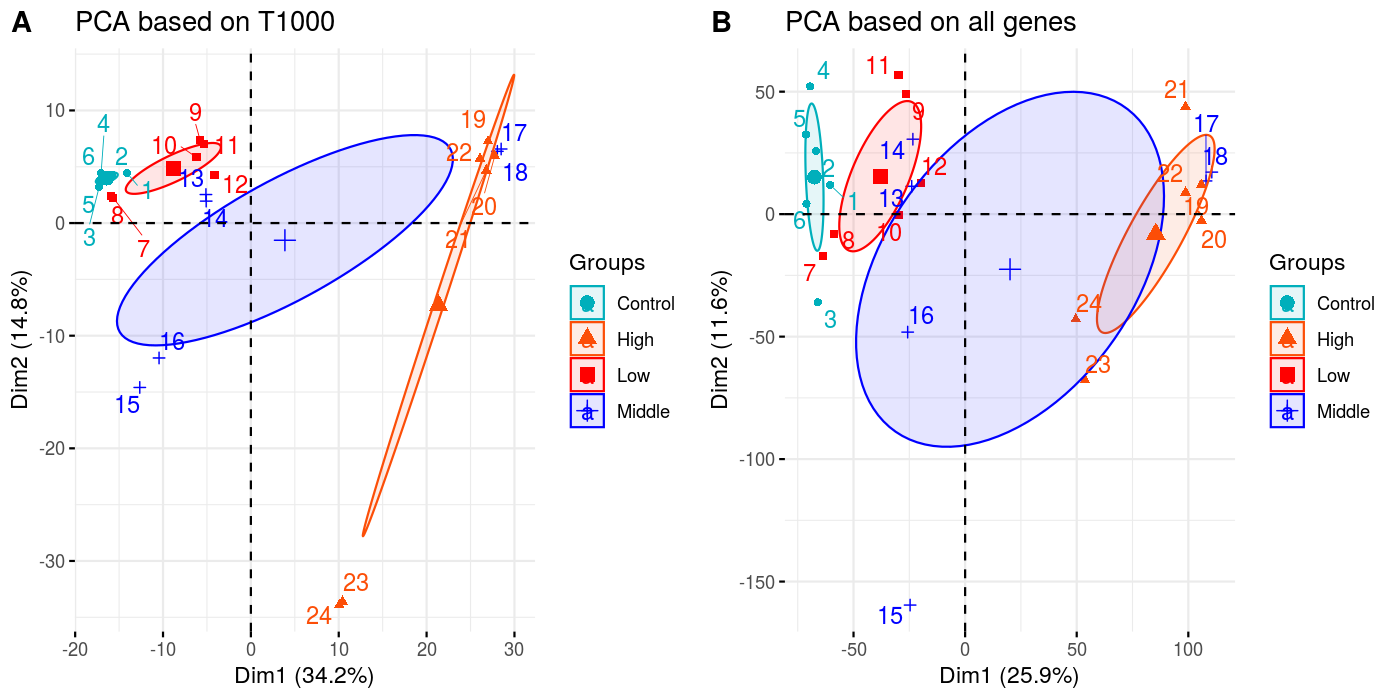

Supplement: Figure S5 [file peerj-07-7975-s005.zip › Supplementary_Figures_S5/flutamide.Human.in_vitro.Liver.tiff]

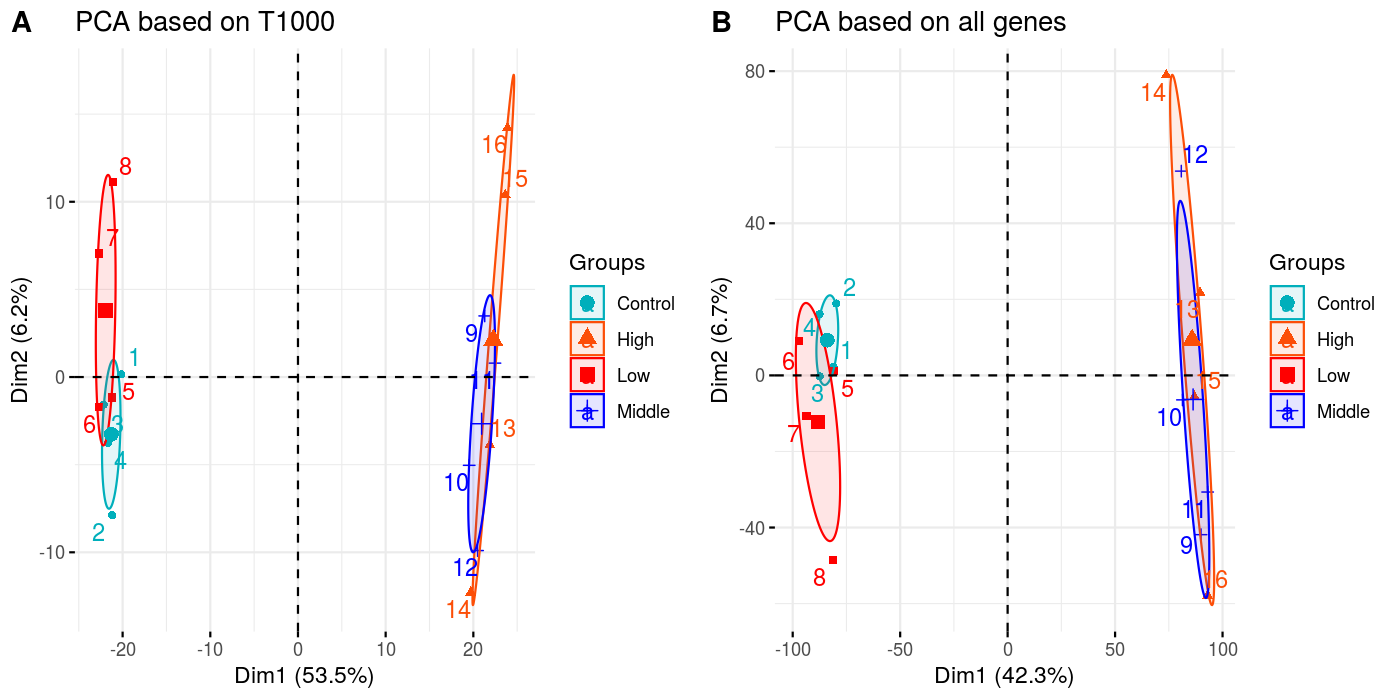

Supplement: Figure S5 [file peerj-07-7975-s005.zip › Supplementary_Figures_S5/buthionine_sulfoximine.Human.in_vitro.Liver.tiff]
