# Supplementary figures and images for: T1000: a reduced gene set prioritized for toxicogenomic studies (part 2 of 2)
Source: PeerJ. 2019 Oct 29;7:e7975. doi: 10.7717/peerj.7975 (PMC6824333; doi:10.7717/peerj.7975)

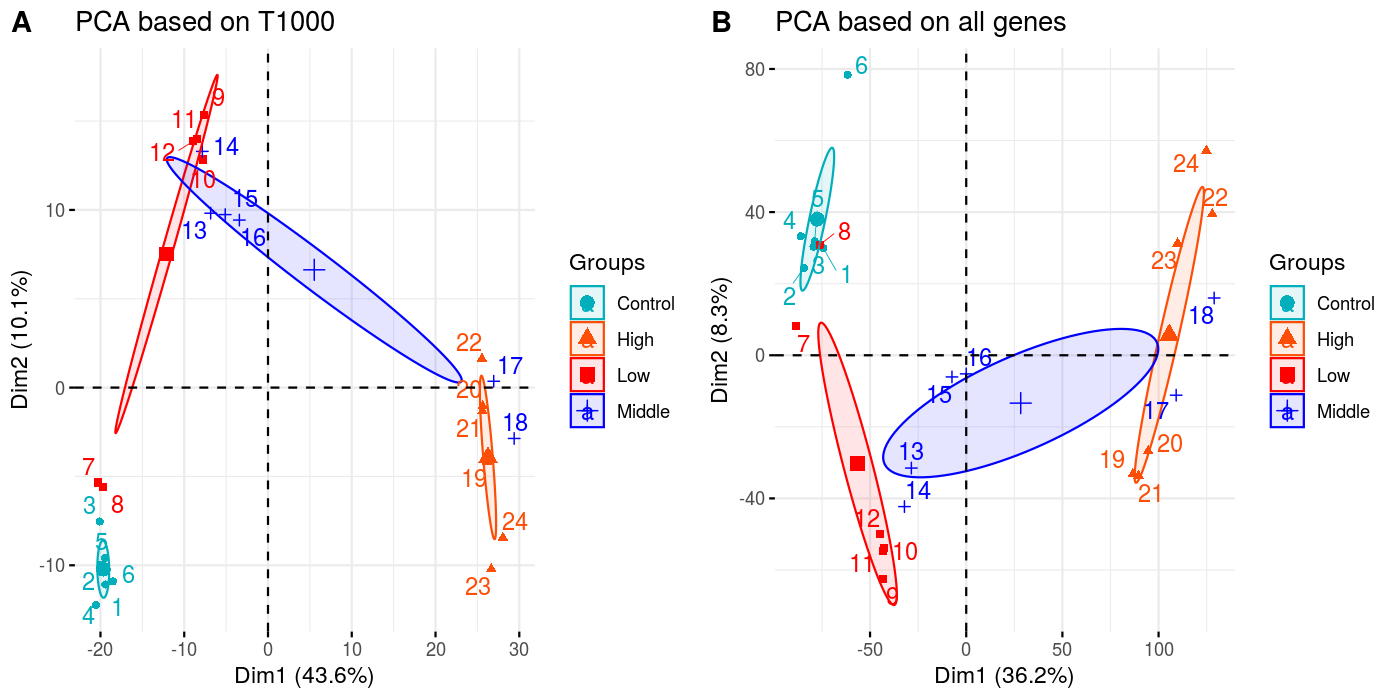

Supplement: Figure S5 [file peerj-07-7975-s005.zip › Supplementary_Figures_S5/indomethacin.Human.in_vitro.Liver.tiff]

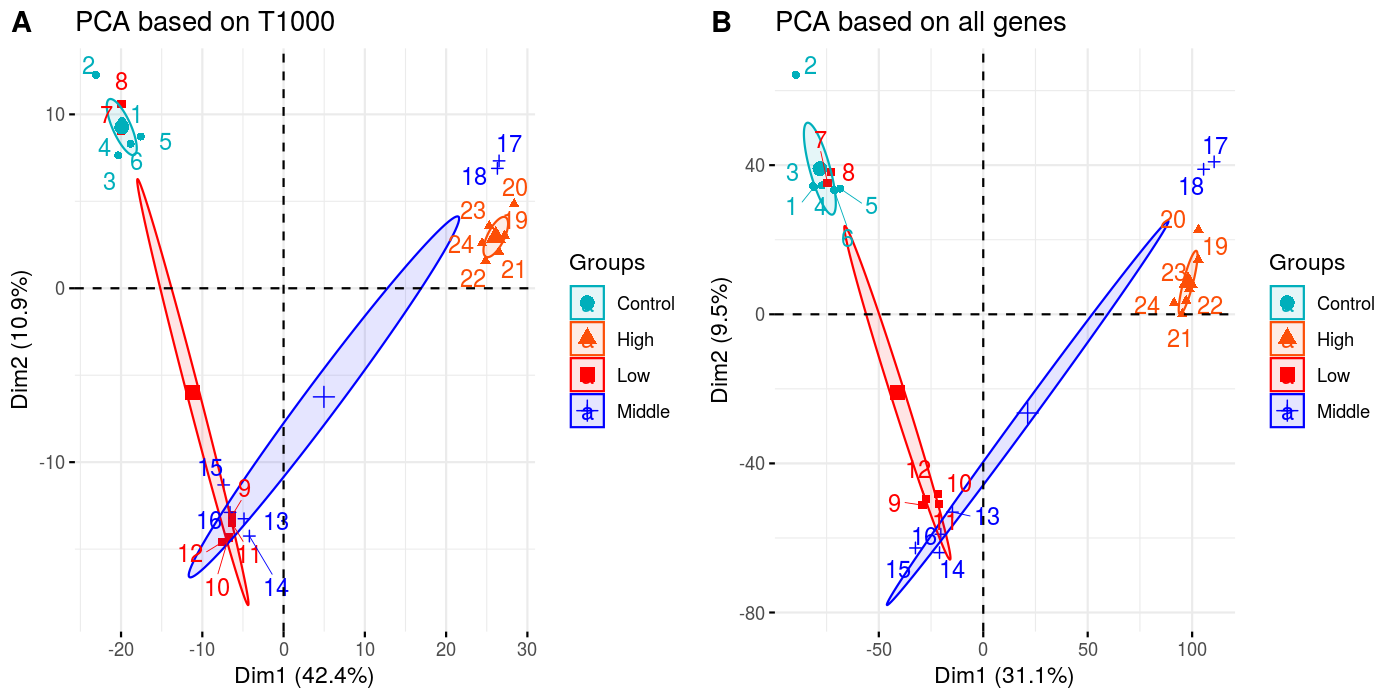

Supplement: Figure S5 [file peerj-07-7975-s005.zip › Supplementary_Figures_S5/allopurinol.Human.in_vitro.Liver.tiff]

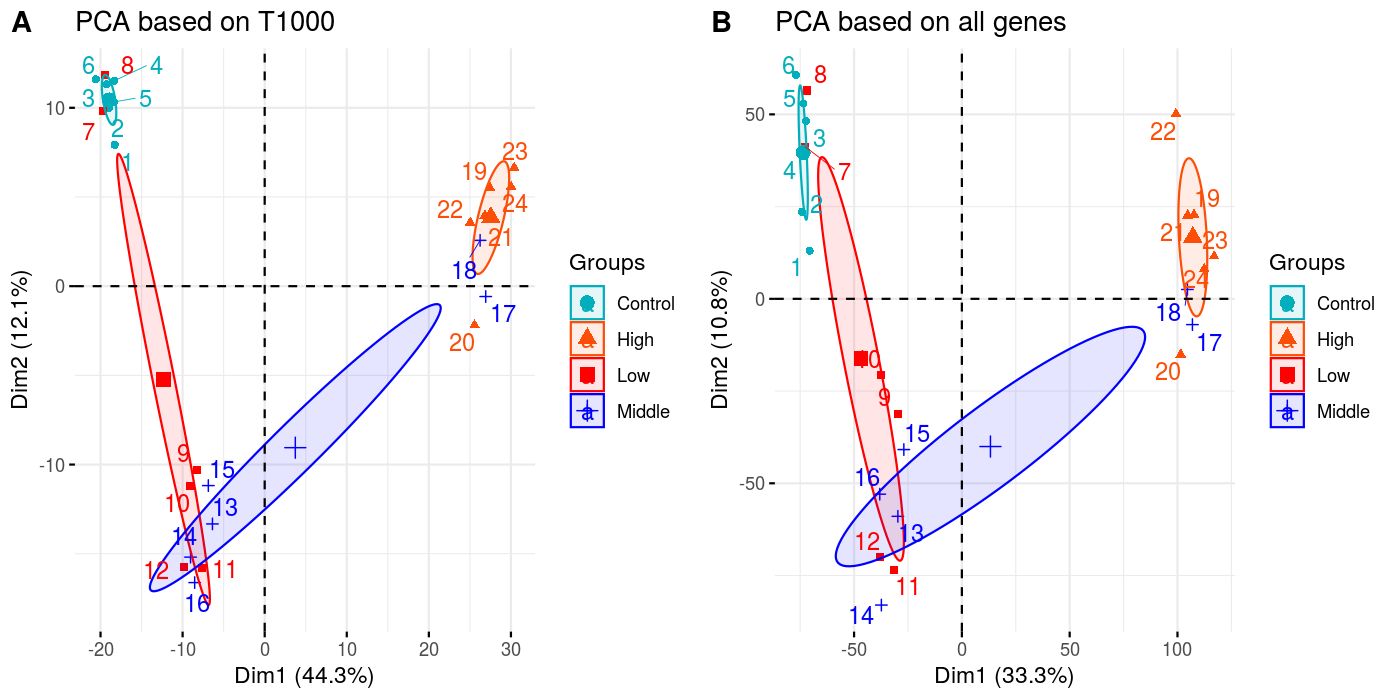

Supplement: Figure S5 [file peerj-07-7975-s005.zip › Supplementary_Figures_S5/hexachlorobenzene.Human.in_vitro.Liver.tiff]

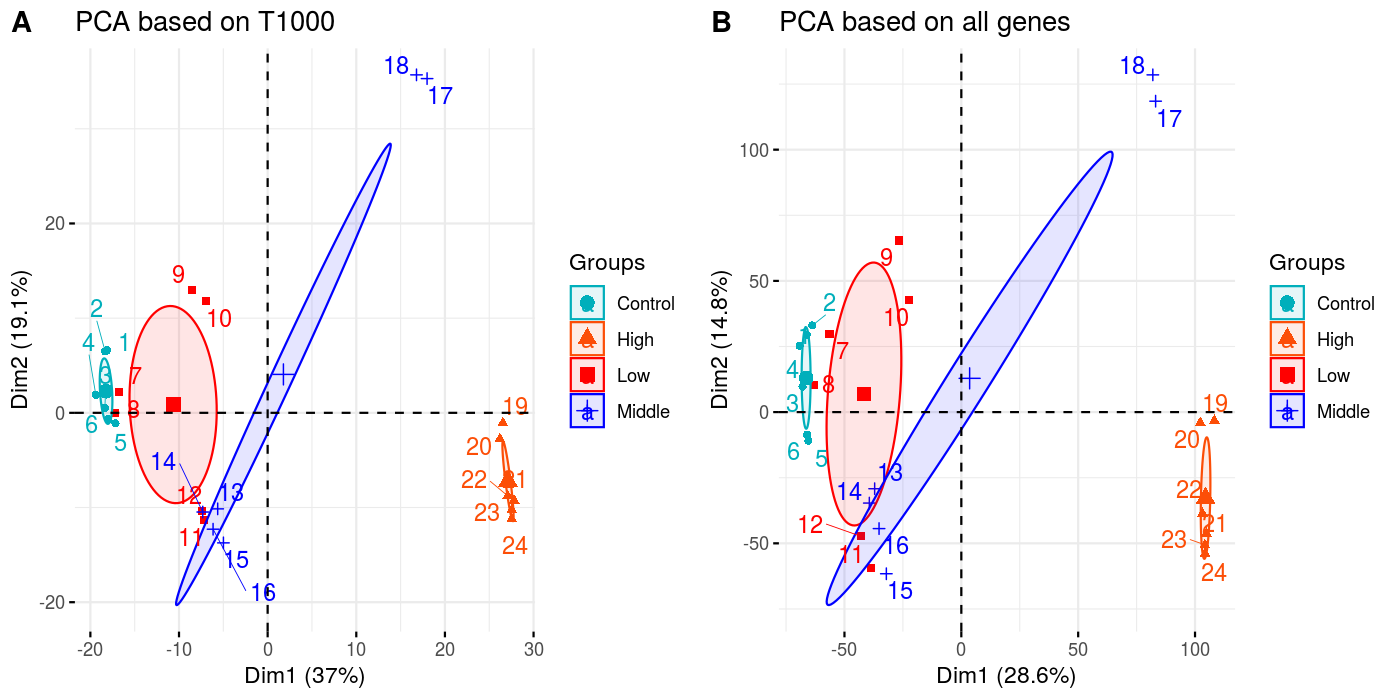

Supplement: Figure S5 [file peerj-07-7975-s005.zip › Supplementary_Figures_S5/interleukin_1_beta,_human.Human.in_vitro.Liver.tiff]

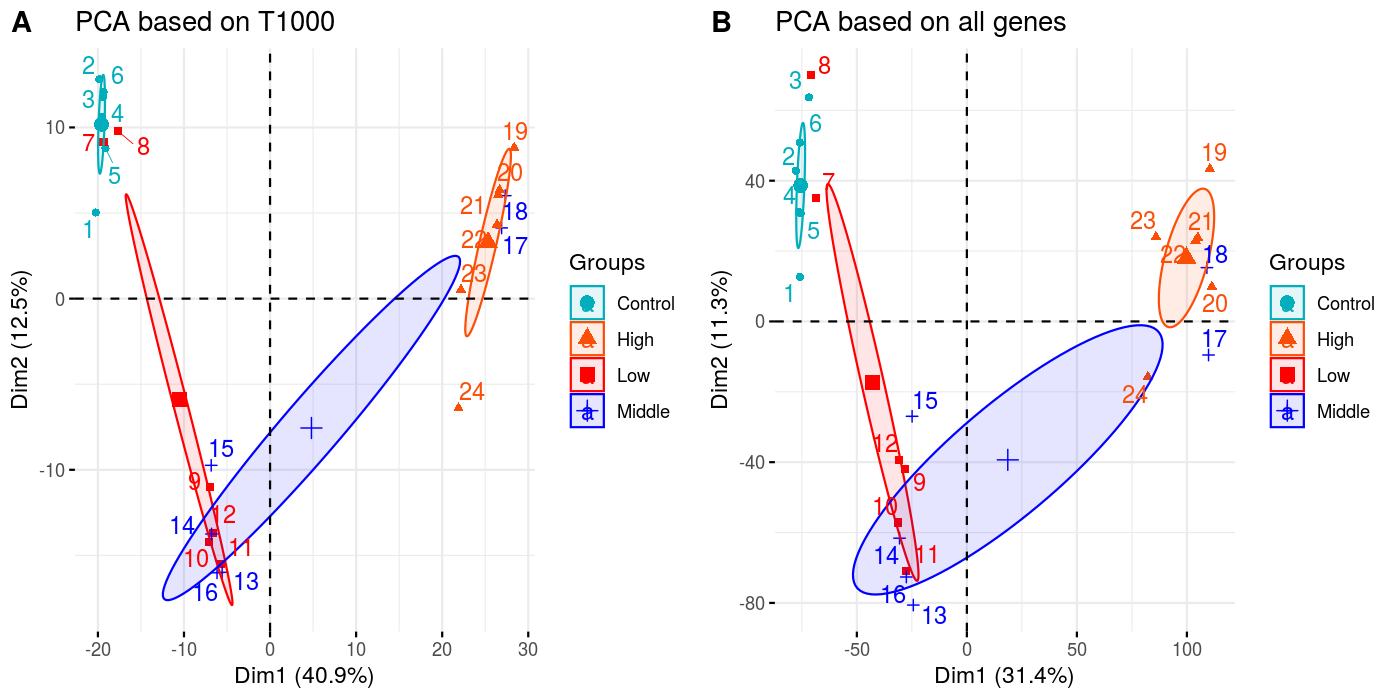

Supplement: Figure S5 [file peerj-07-7975-s005.zip › Supplementary_Figures_S5/carbamazepine.Human.in_vitro.Liver.tiff]

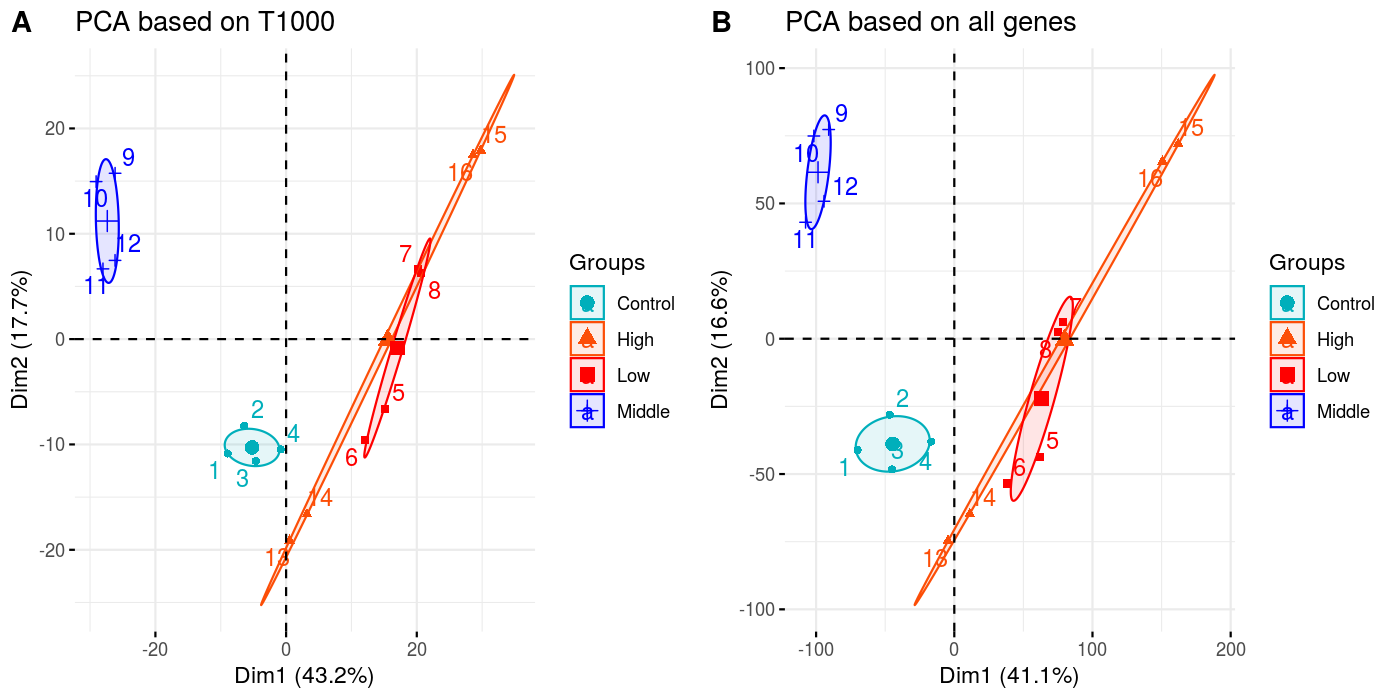

Supplement: Figure S5 [file peerj-07-7975-s005.zip › Supplementary_Figures_S5/N-methyl-N-nitrosourea.Human.in_vitro.Liver.tiff]

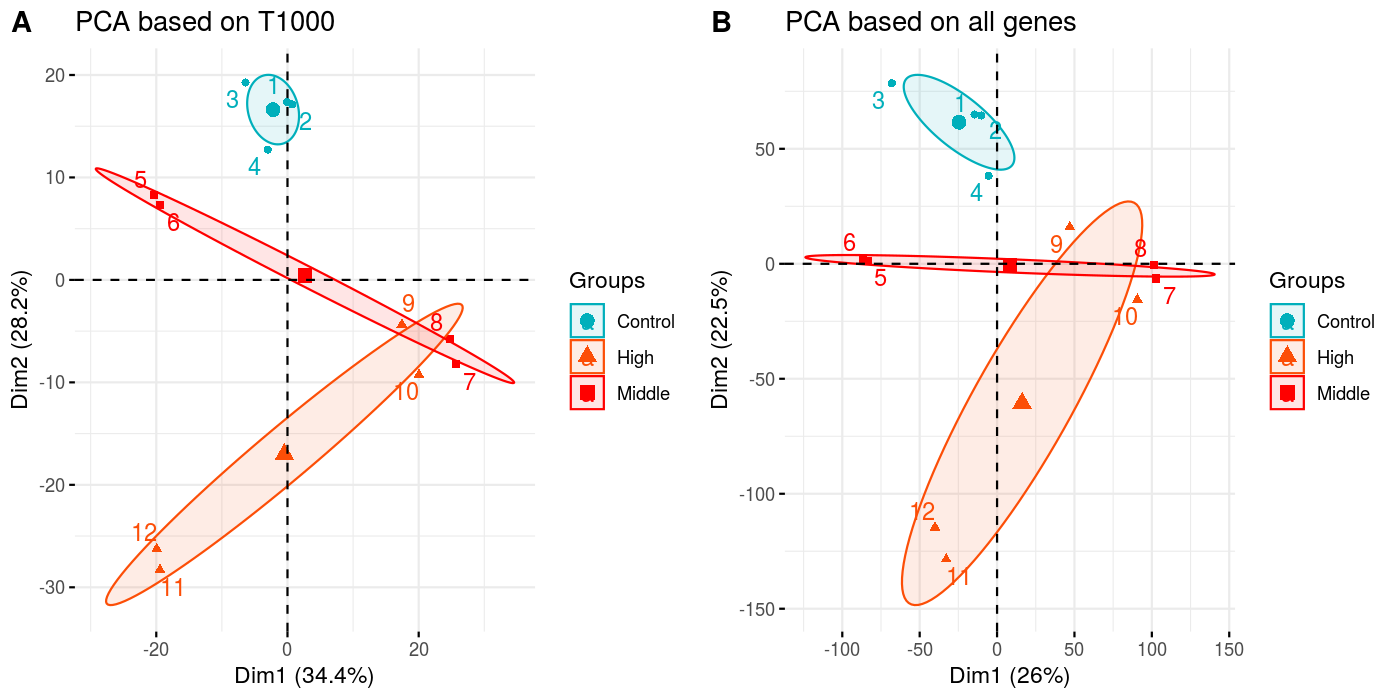

Supplement: Figure S5 [file peerj-07-7975-s005.zip › Supplementary_Figures_S5/danazol.Human.in_vitro.Liver.tiff]

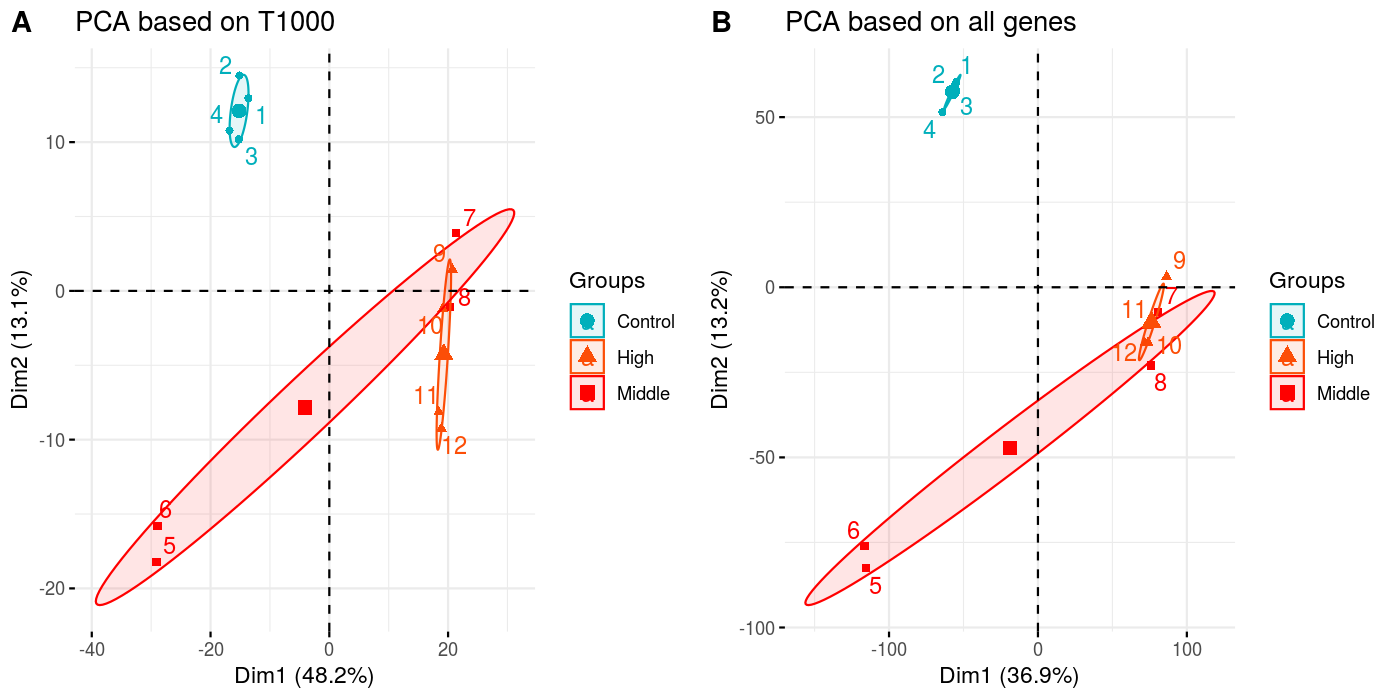

Supplement: Figure S5 [file peerj-07-7975-s005.zip › Supplementary_Figures_S5/disulfiram.Human.in_vitro.Liver.tiff]

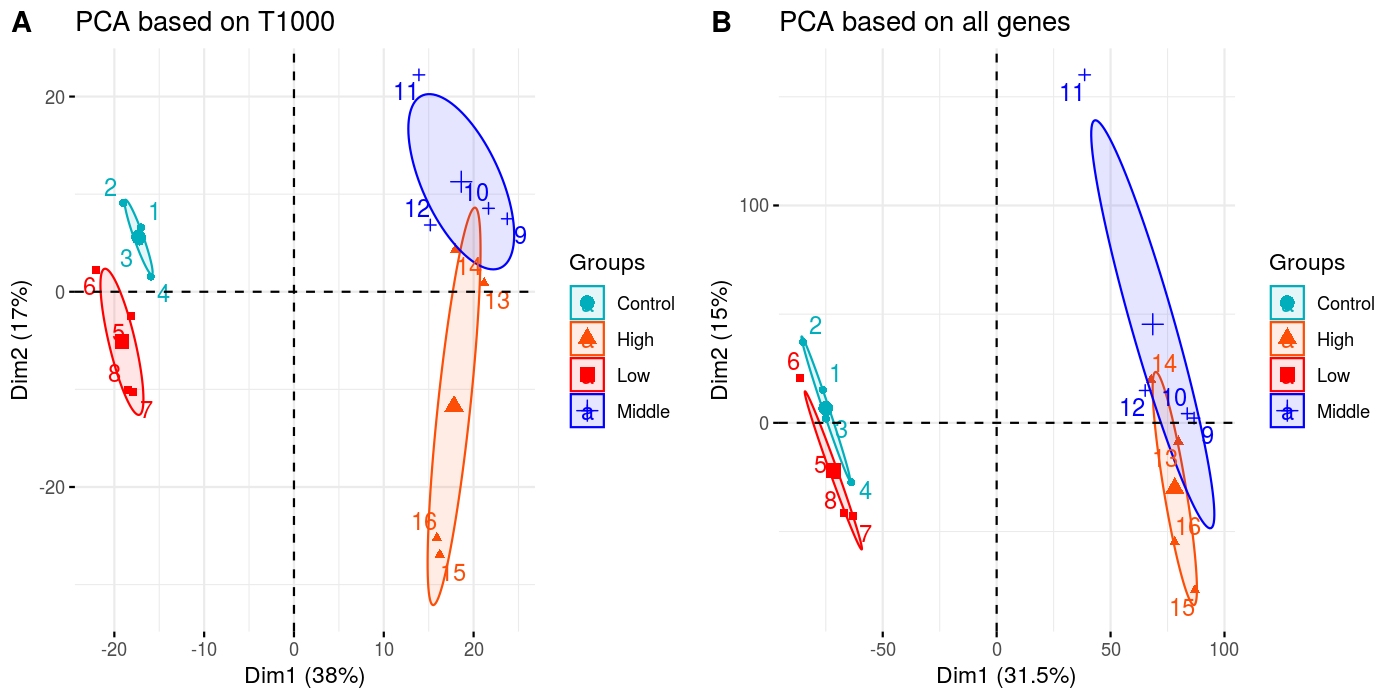

Supplement: Figure S5 [file peerj-07-7975-s005.zip › Supplementary_Figures_S5/cyclosporine_A.Human.in_vitro.Liver.tiff]

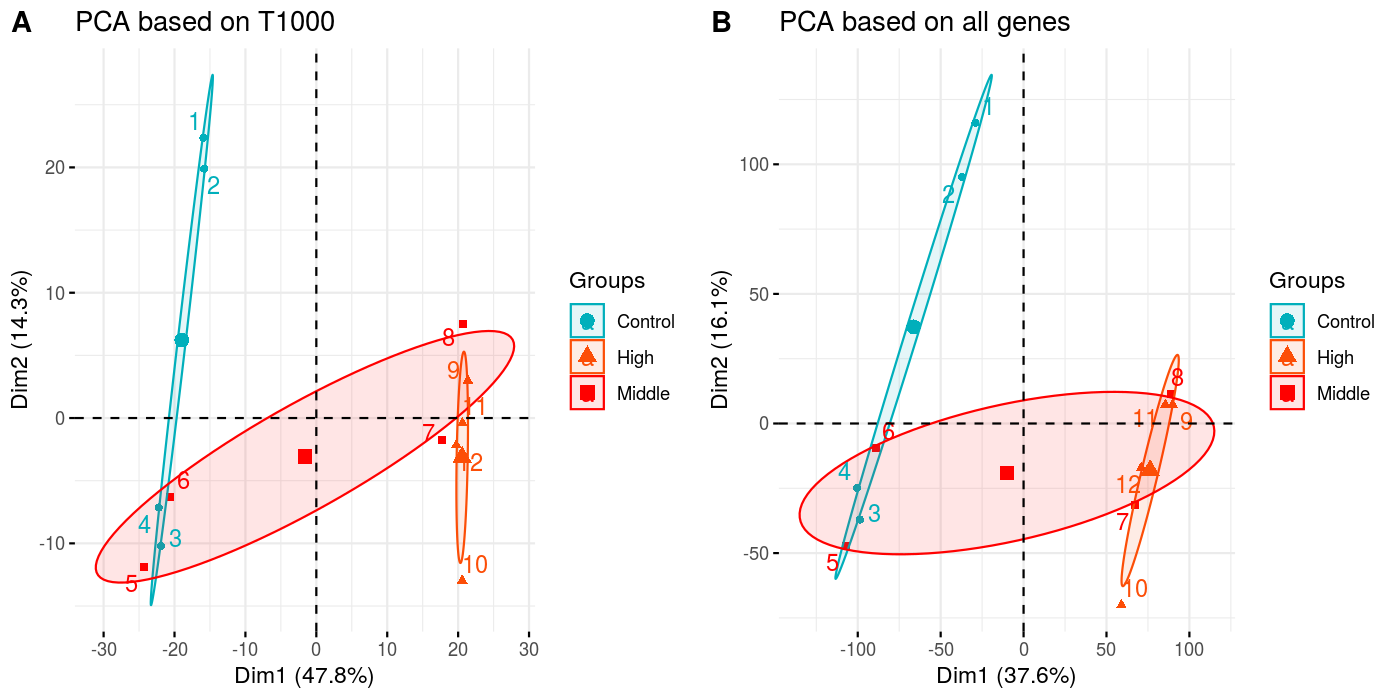

Supplement: Figure S5 [file peerj-07-7975-s005.zip › Supplementary_Figures_S5/vitamin_A.Human.in_vitro.Liver.tiff]

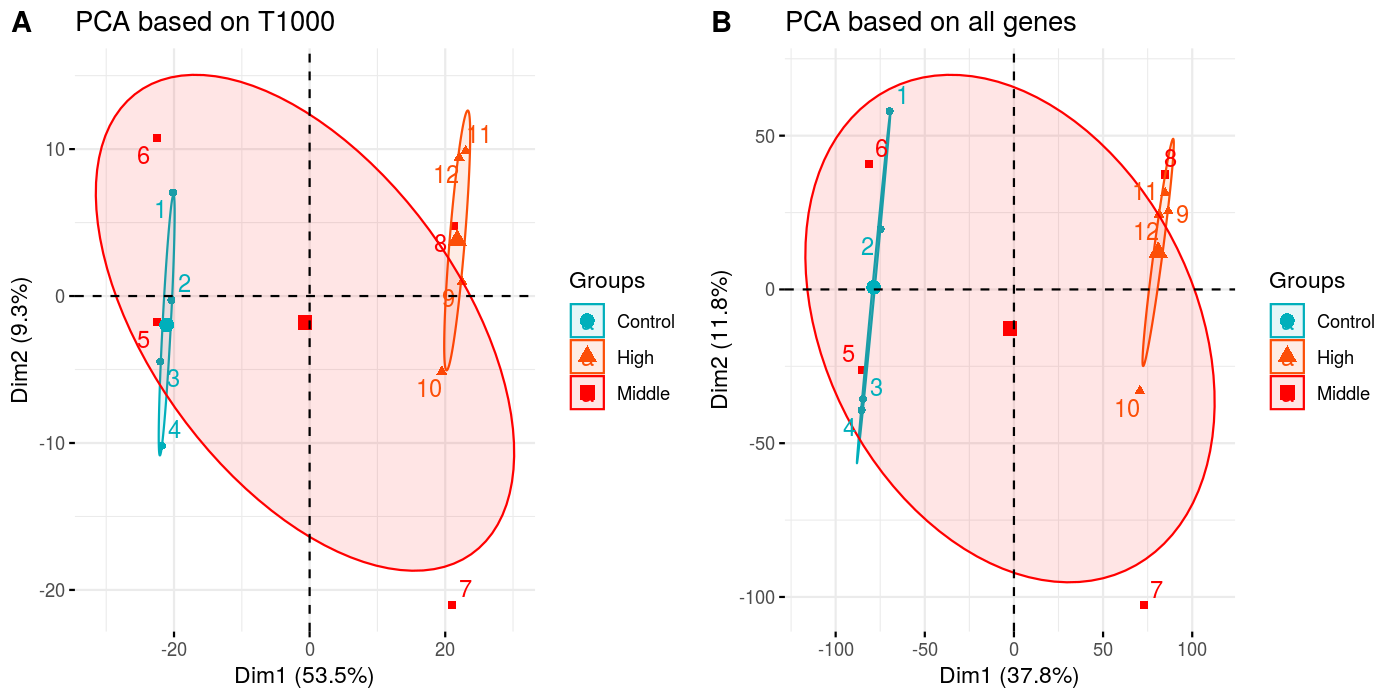

Supplement: Figure S5 [file peerj-07-7975-s005.zip › Supplementary_Figures_S5/imipramine.Human.in_vitro.Liver.tiff]

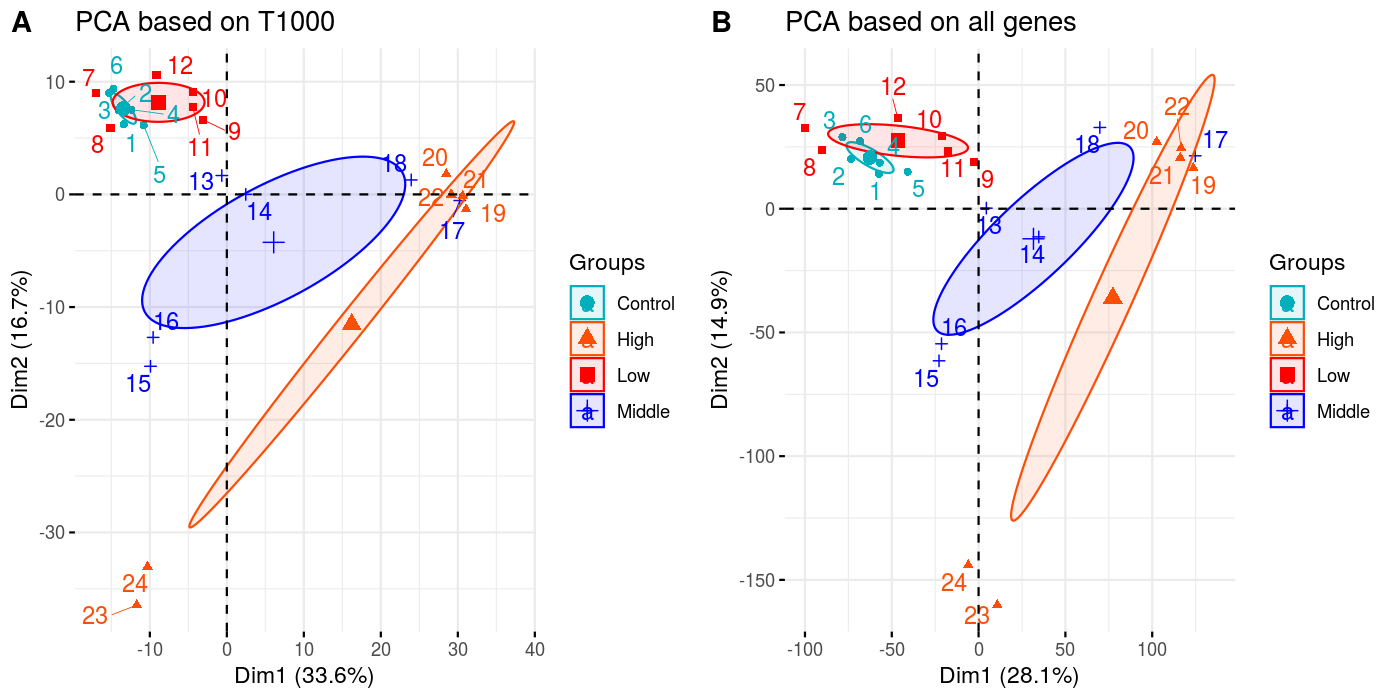

Supplement: Figure S5 [file peerj-07-7975-s005.zip › Supplementary_Figures_S5/allyl_alcohol.Human.in_vitro.Liver.tiff]

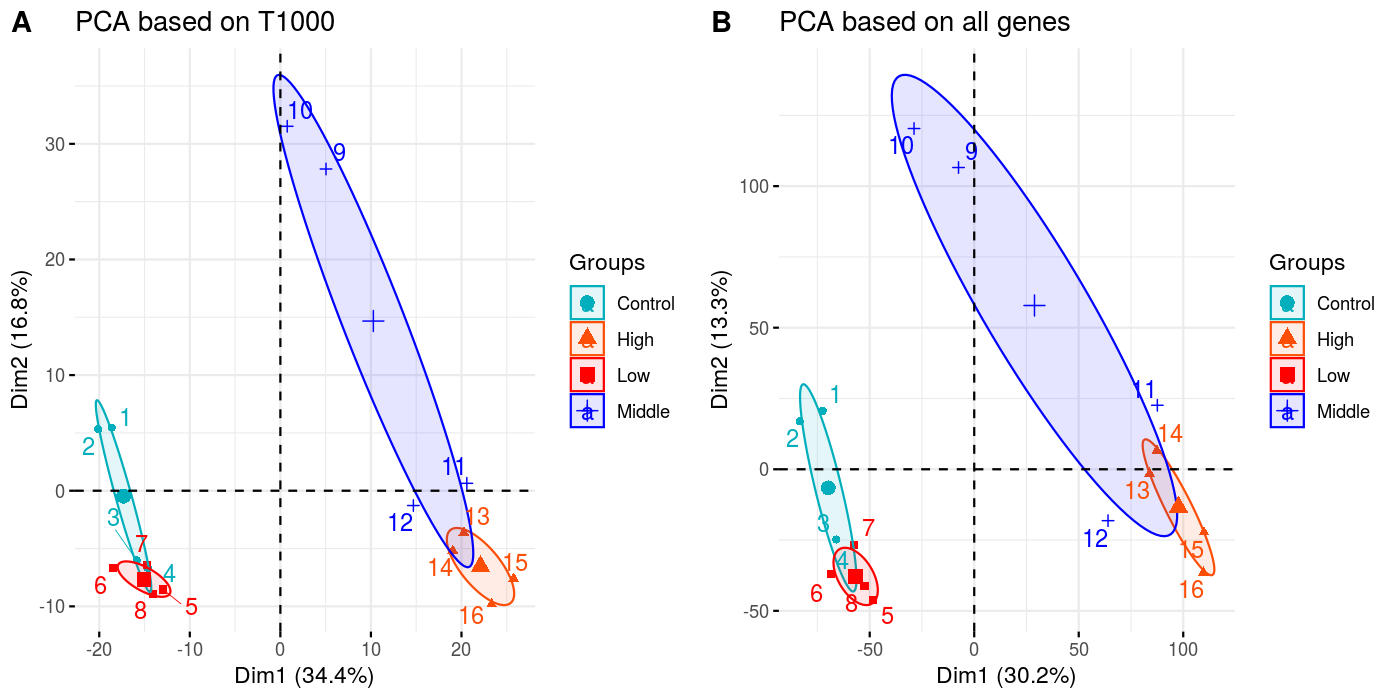

Supplement: Figure S5 [file peerj-07-7975-s005.zip › Supplementary_Figures_S5/interferon_alpha,_human.Human.in_vitro.Liver.tiff]

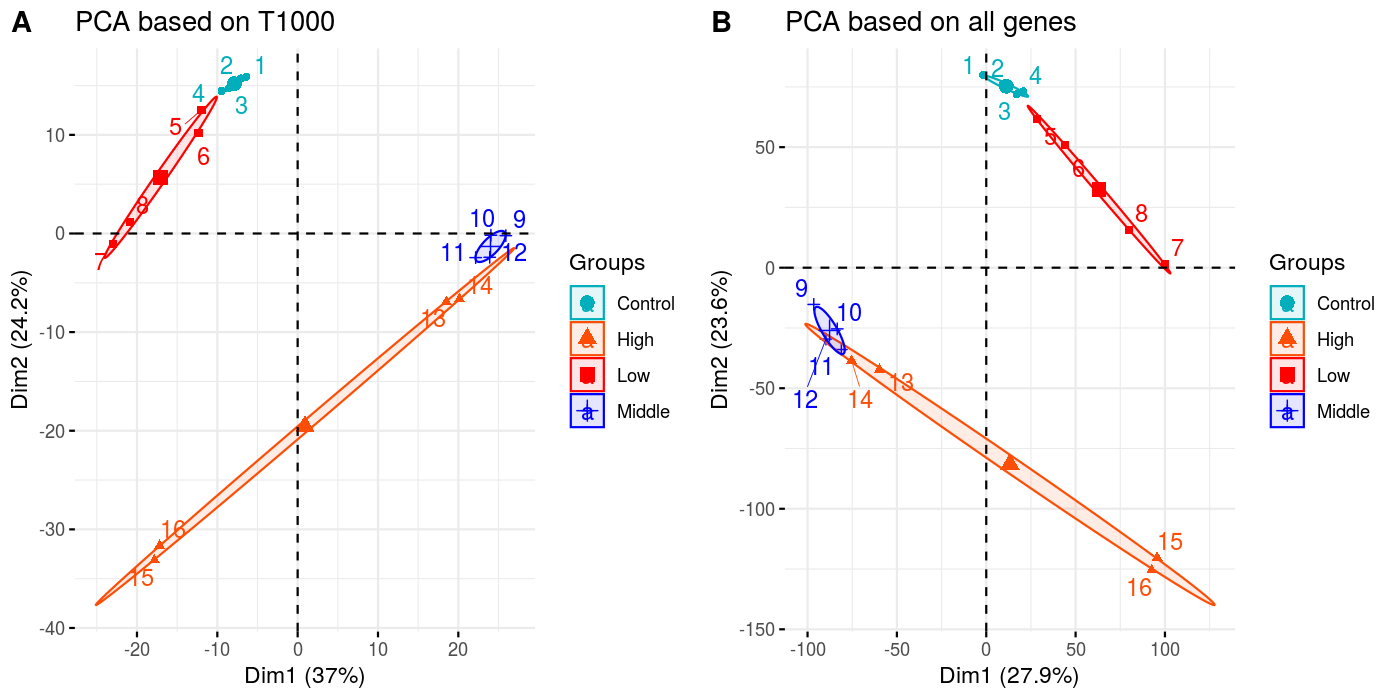

Supplement: Figure S5 [file peerj-07-7975-s005.zip › Supplementary_Figures_S5/methylene_dianiline.Human.in_vitro.Liver.tiff]

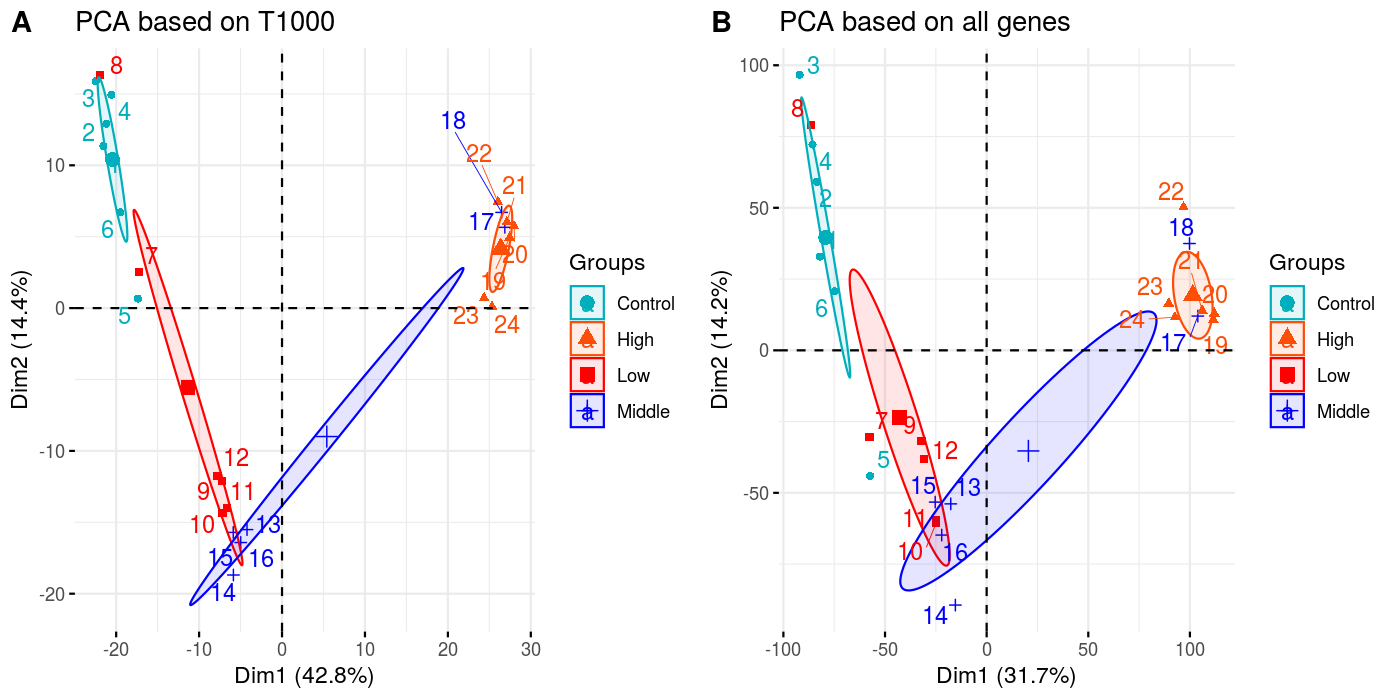

Supplement: Figure S5 [file peerj-07-7975-s005.zip › Supplementary_Figures_S5/phenytoin.Human.in_vitro.Liver.tiff]

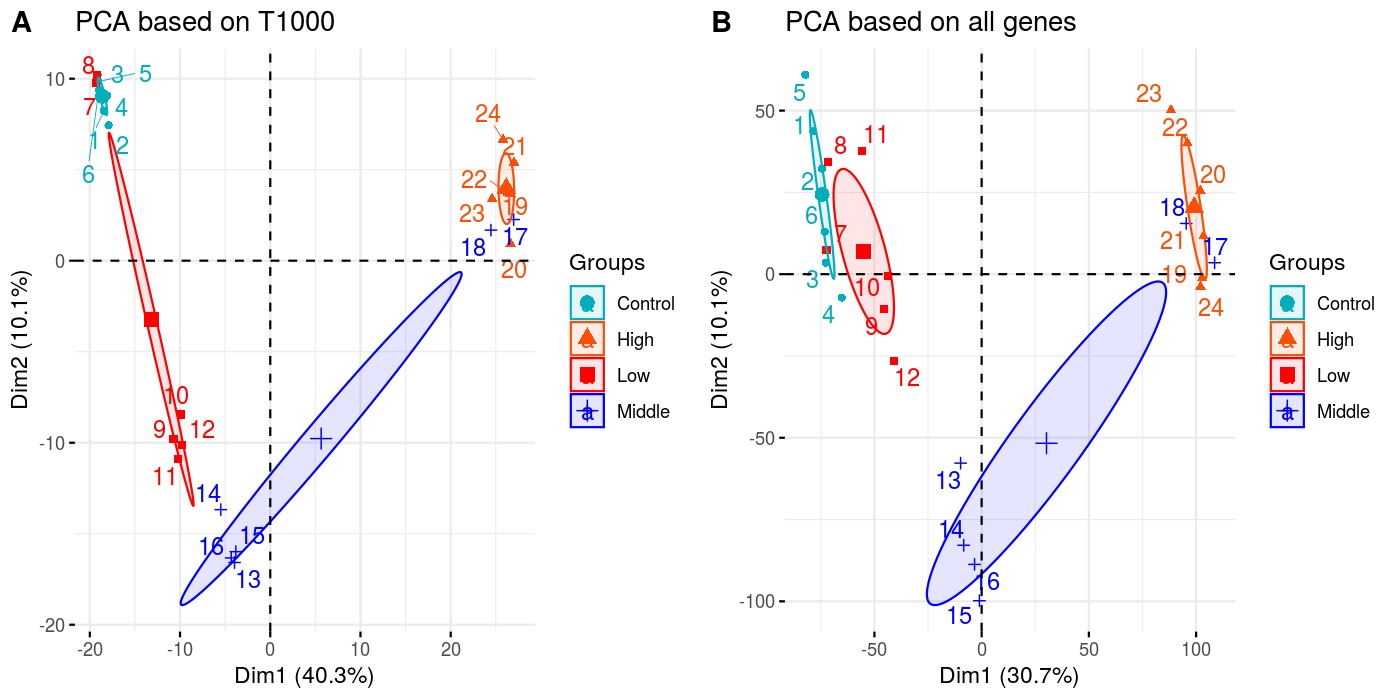

Supplement: Figure S5 [file peerj-07-7975-s005.zip › Supplementary_Figures_S5/sulfasalazine.Human.in_vitro.Liver.tiff]

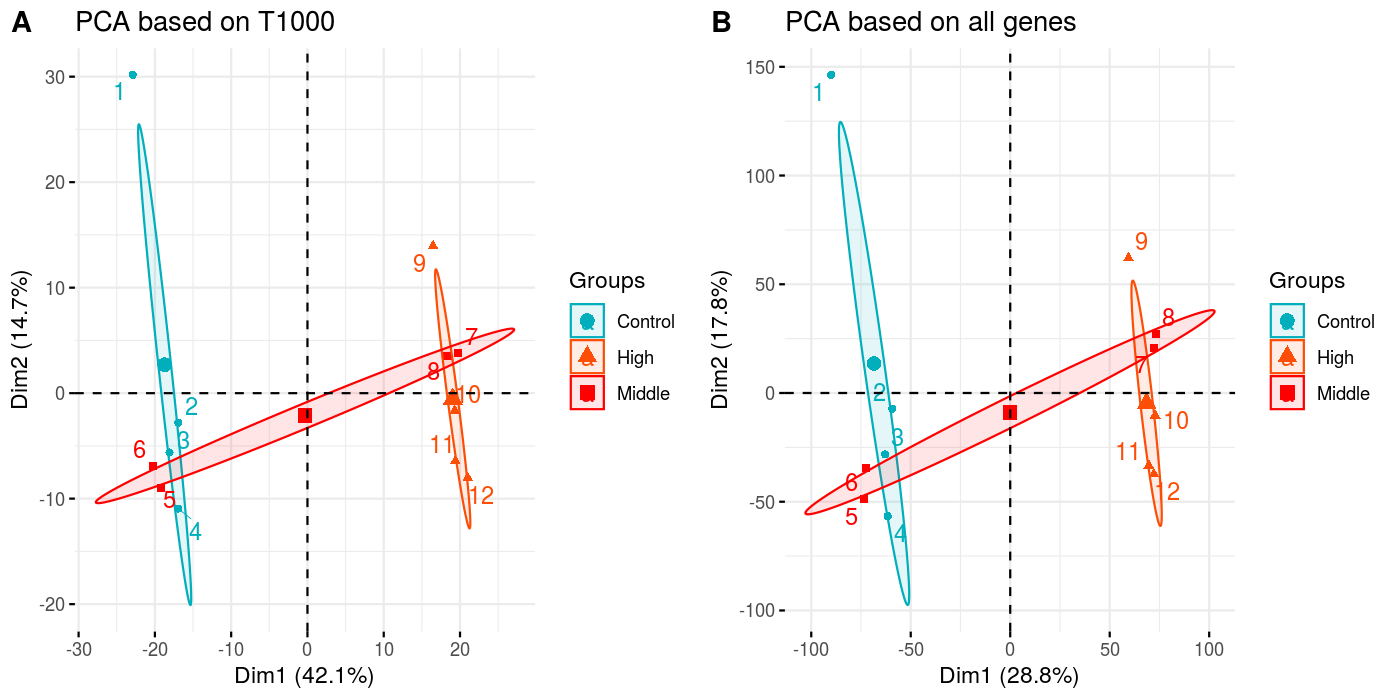

Supplement: Figure S5 [file peerj-07-7975-s005.zip › Supplementary_Figures_S5/acetamidofluorene.Human.in_vitro.Liver.tiff]

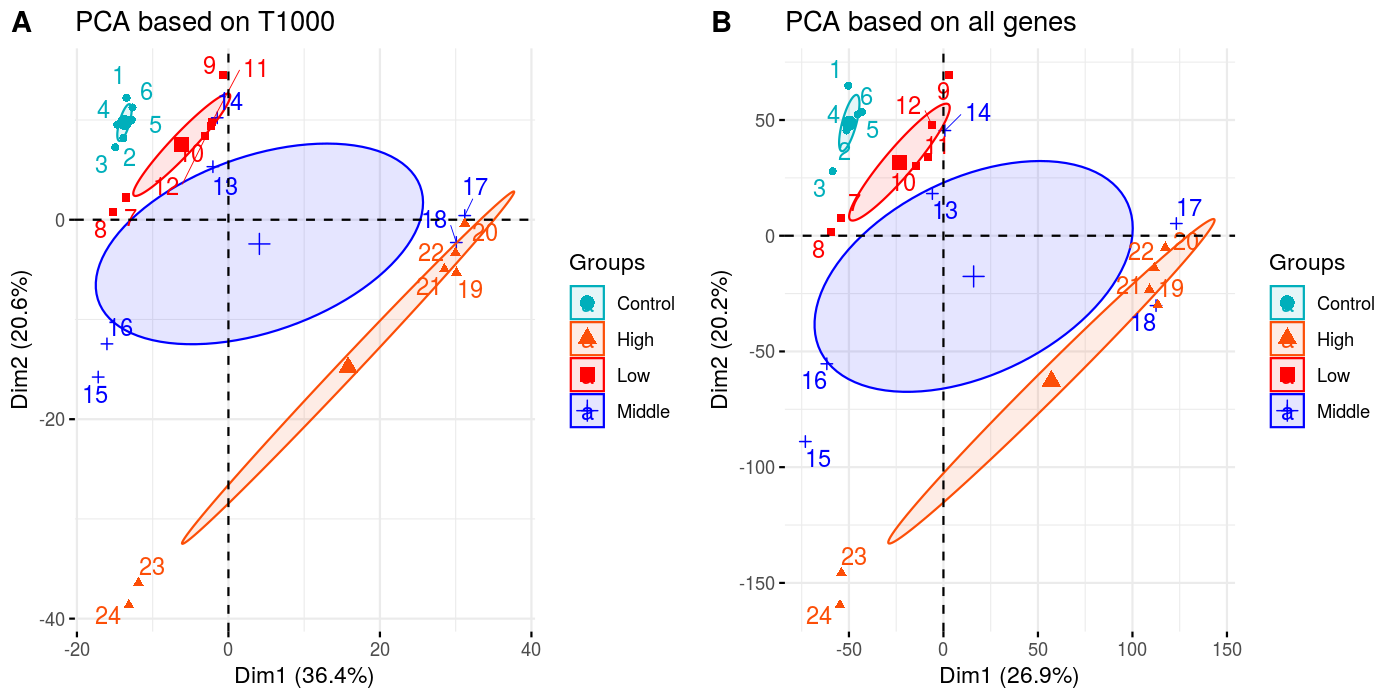

Supplement: Figure S5 [file peerj-07-7975-s005.zip › Supplementary_Figures_S5/benzbromarone.Human.in_vitro.Liver.tiff]

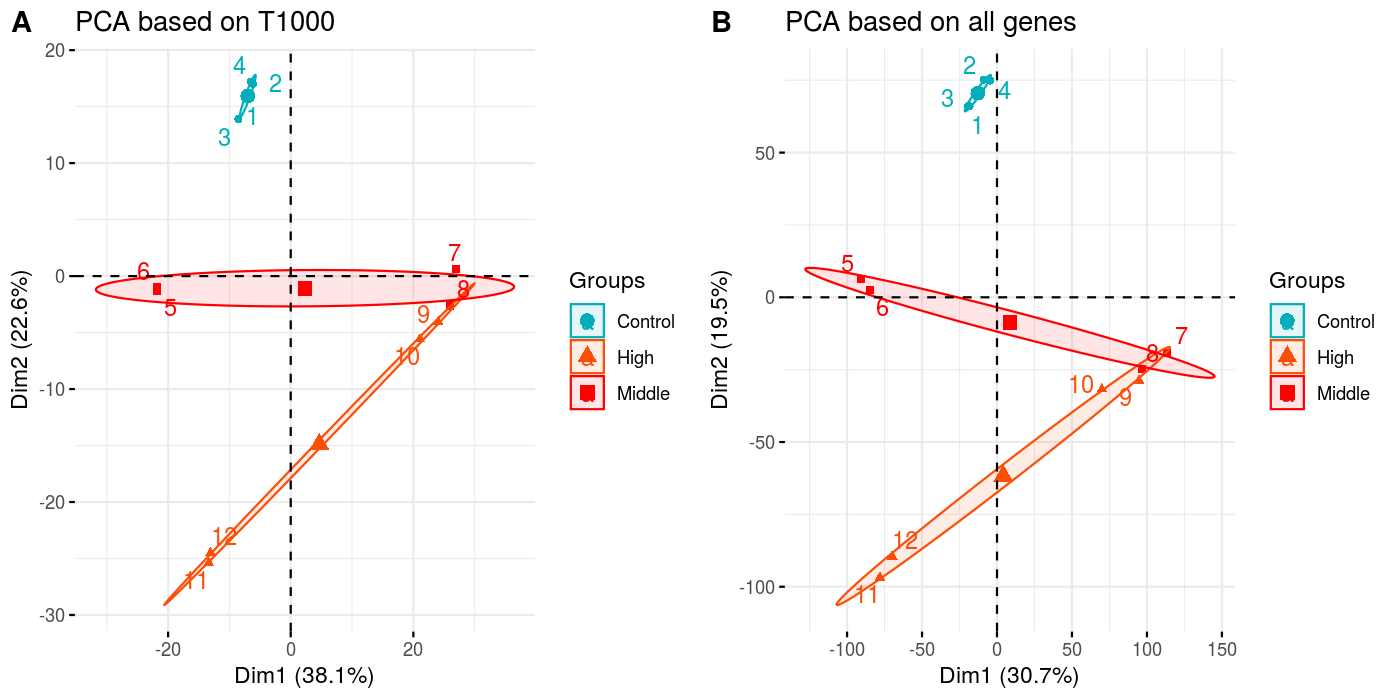

Supplement: Figure S5 [file peerj-07-7975-s005.zip › Supplementary_Figures_S5/nimesulide.Human.in_vitro.Liver.tiff]

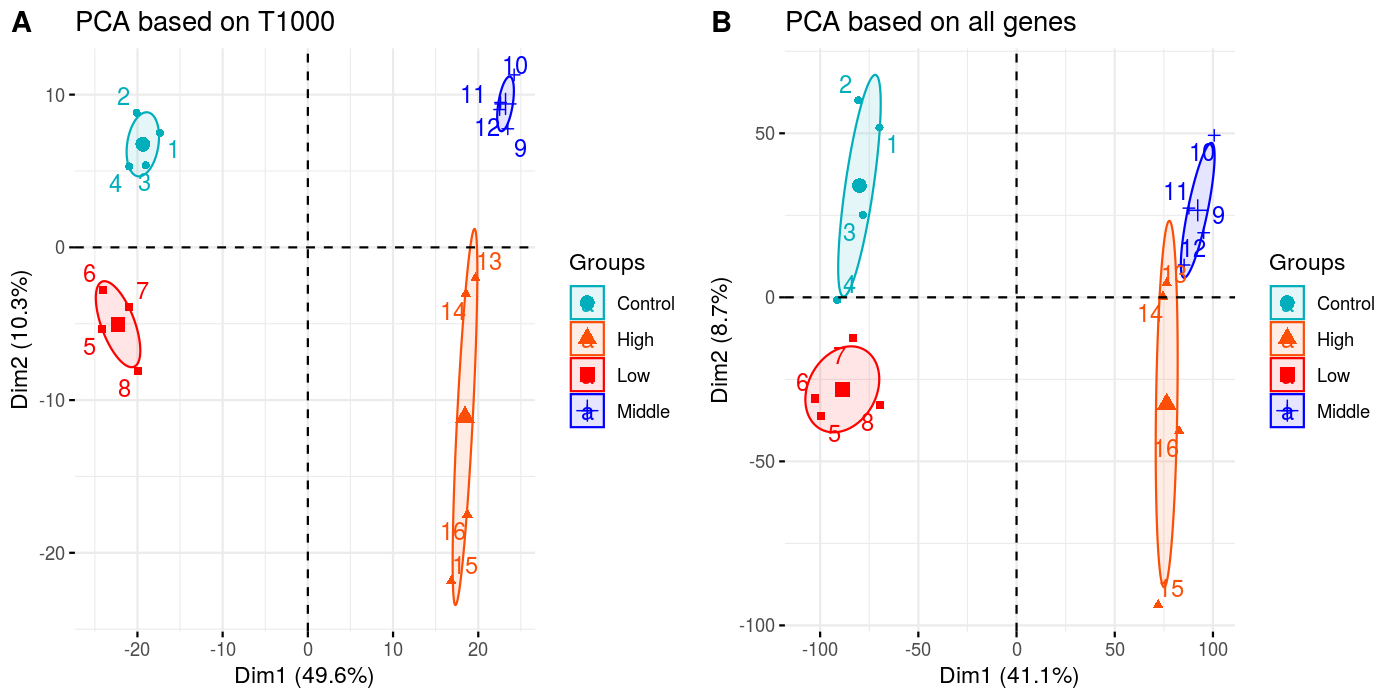

Supplement: Figure S5 [file peerj-07-7975-s005.zip › Supplementary_Figures_S5/2-nitrofluorene.Human.in_vitro.Liver.tiff]

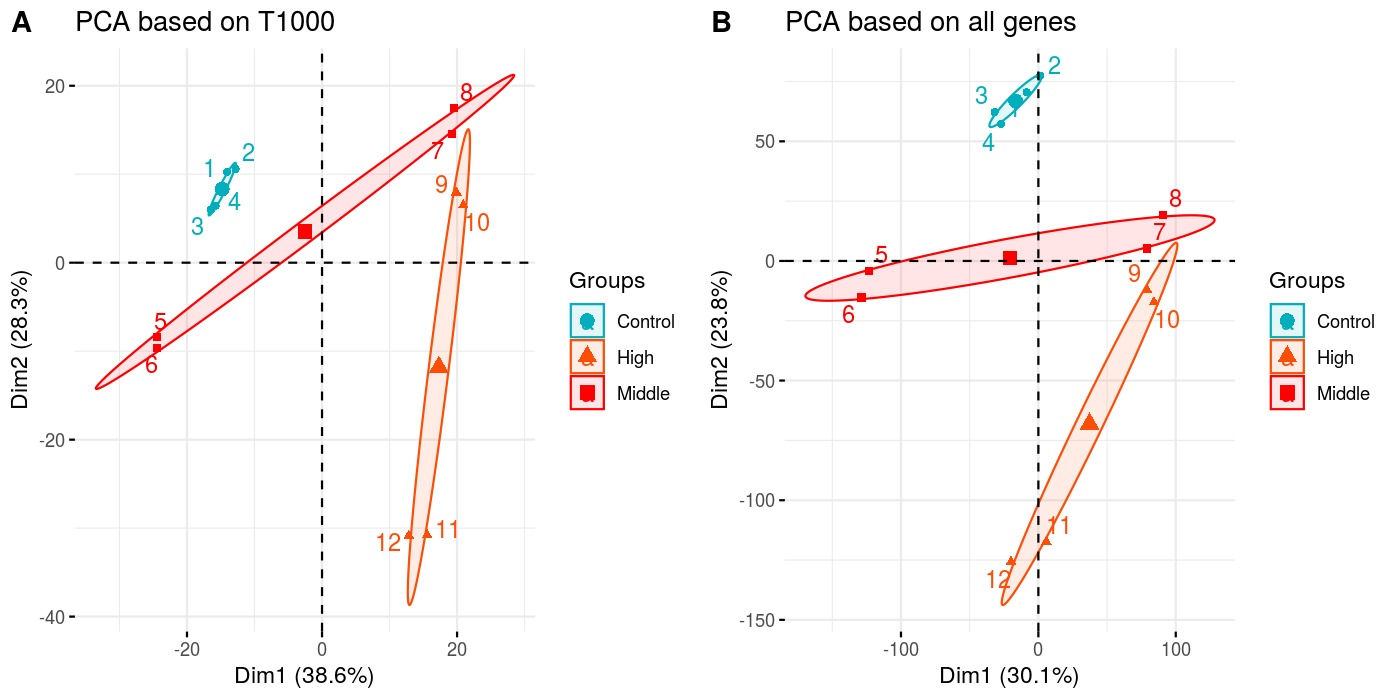

Supplement: Figure S5 [file peerj-07-7975-s005.zip › Supplementary_Figures_S5/ranitidine.Human.in_vitro.Liver.tiff]

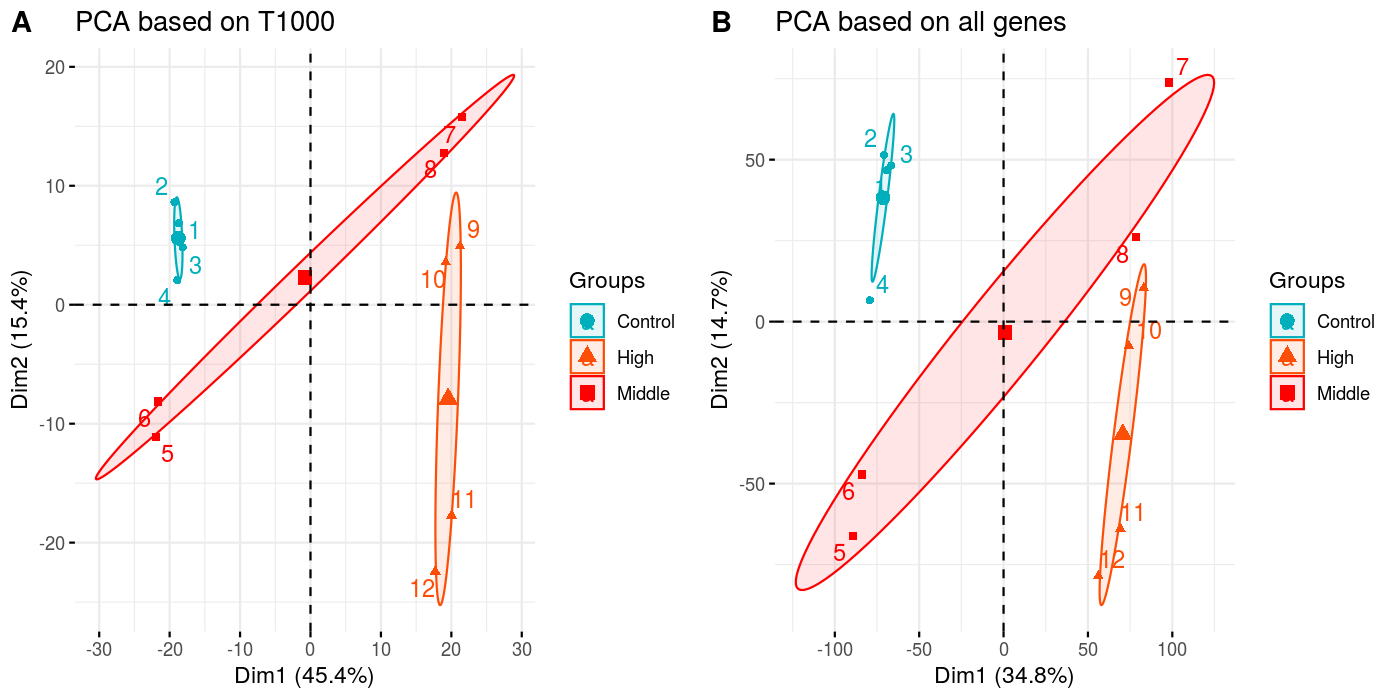

Supplement: Figure S5 [file peerj-07-7975-s005.zip › Supplementary_Figures_S5/benziodarone.Human.in_vitro.Liver.tiff]

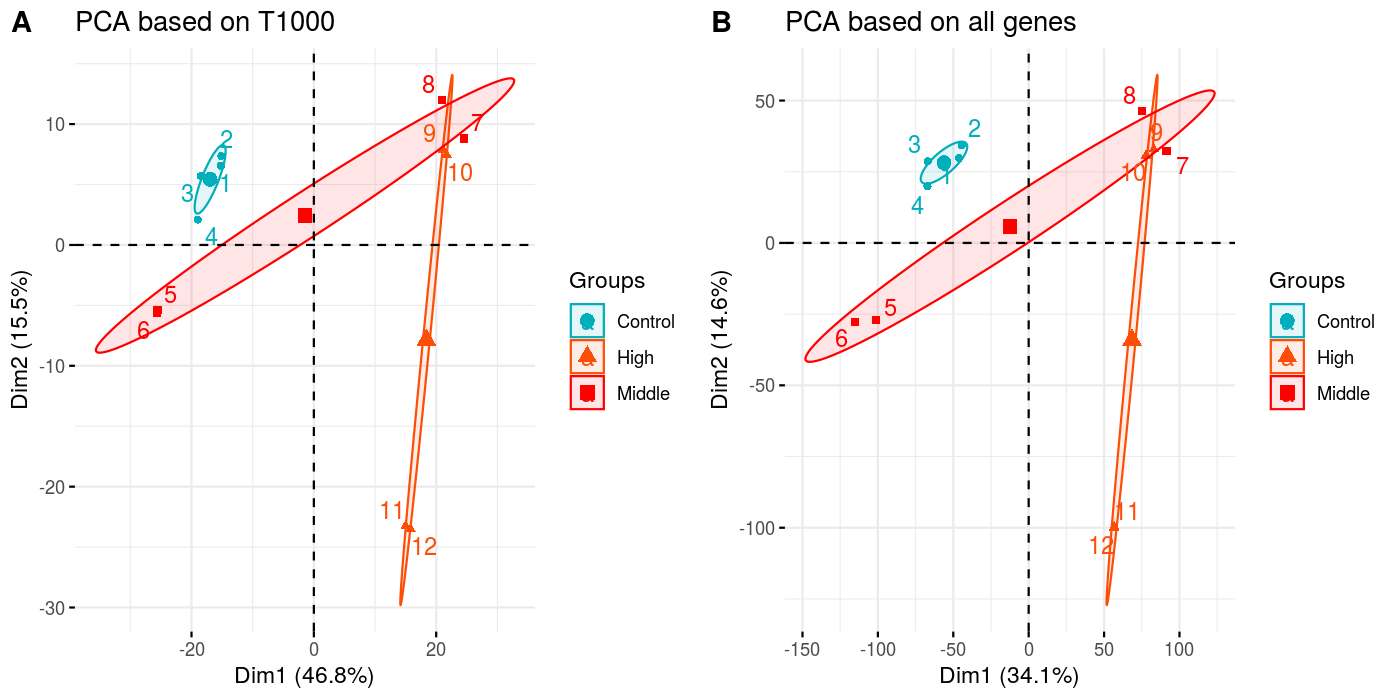

Supplement: Figure S5 [file peerj-07-7975-s005.zip › Supplementary_Figures_S5/enalapril.Human.in_vitro.Liver.tiff]

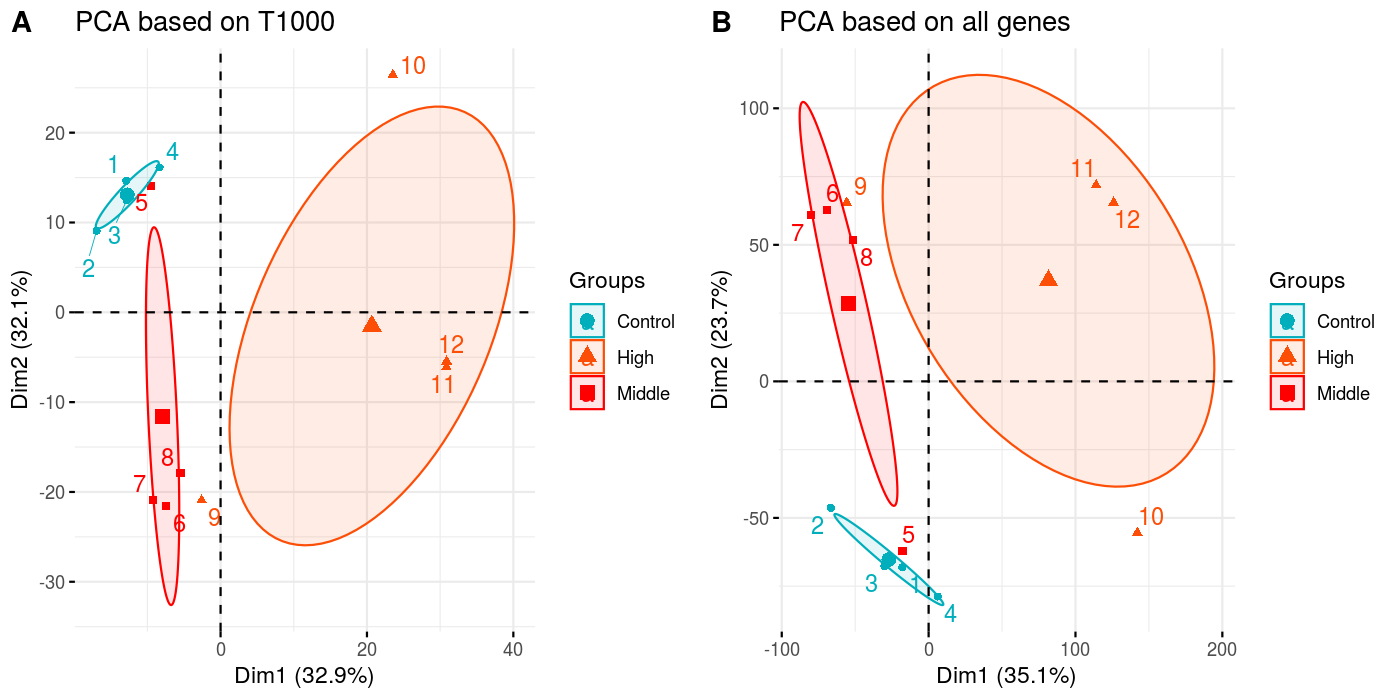

Supplement: Figure S5 [file peerj-07-7975-s005.zip › Supplementary_Figures_S5/phenylanthranilic_acid.Human.in_vitro.Liver.tiff]

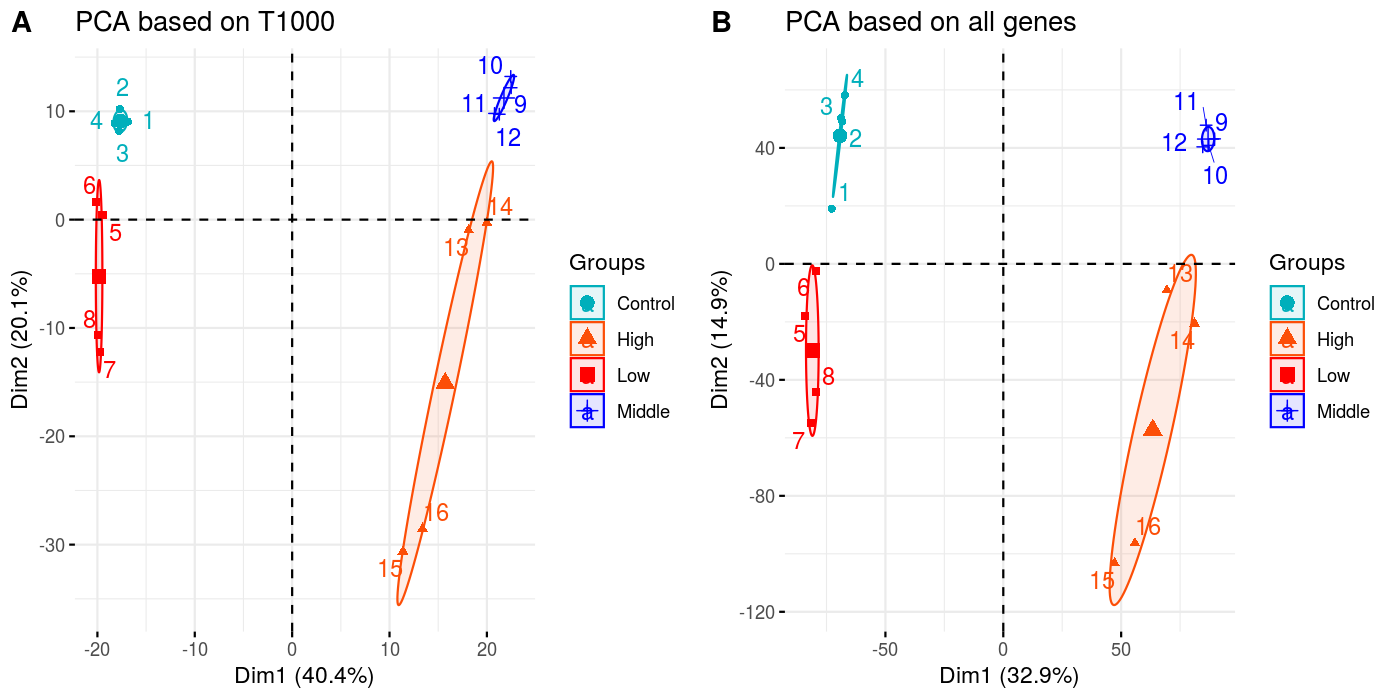

Supplement: Figure S5 [file peerj-07-7975-s005.zip › Supplementary_Figures_S5/fluoxetine_hydrochloride.Human.in_vitro.Liver.tiff]

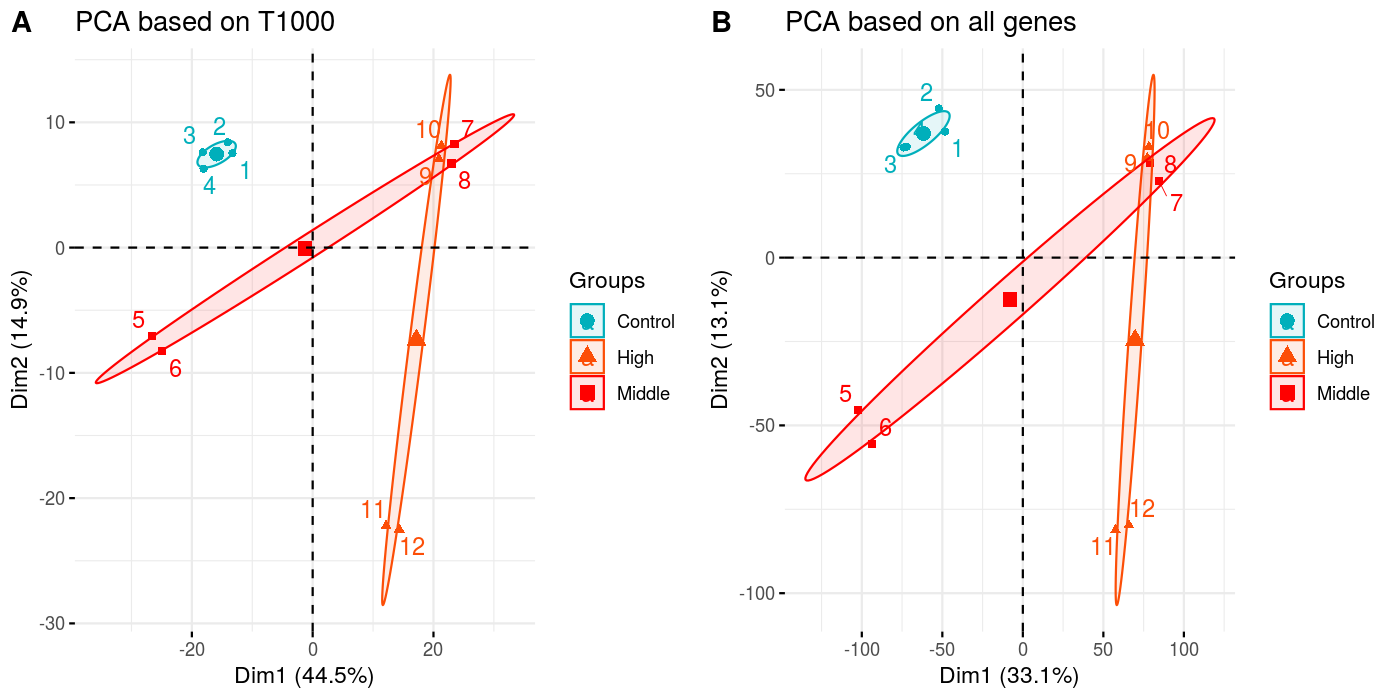

Supplement: Figure S5 [file peerj-07-7975-s005.zip › Supplementary_Figures_S5/nifedipine.Human.in_vitro.Liver.tiff]

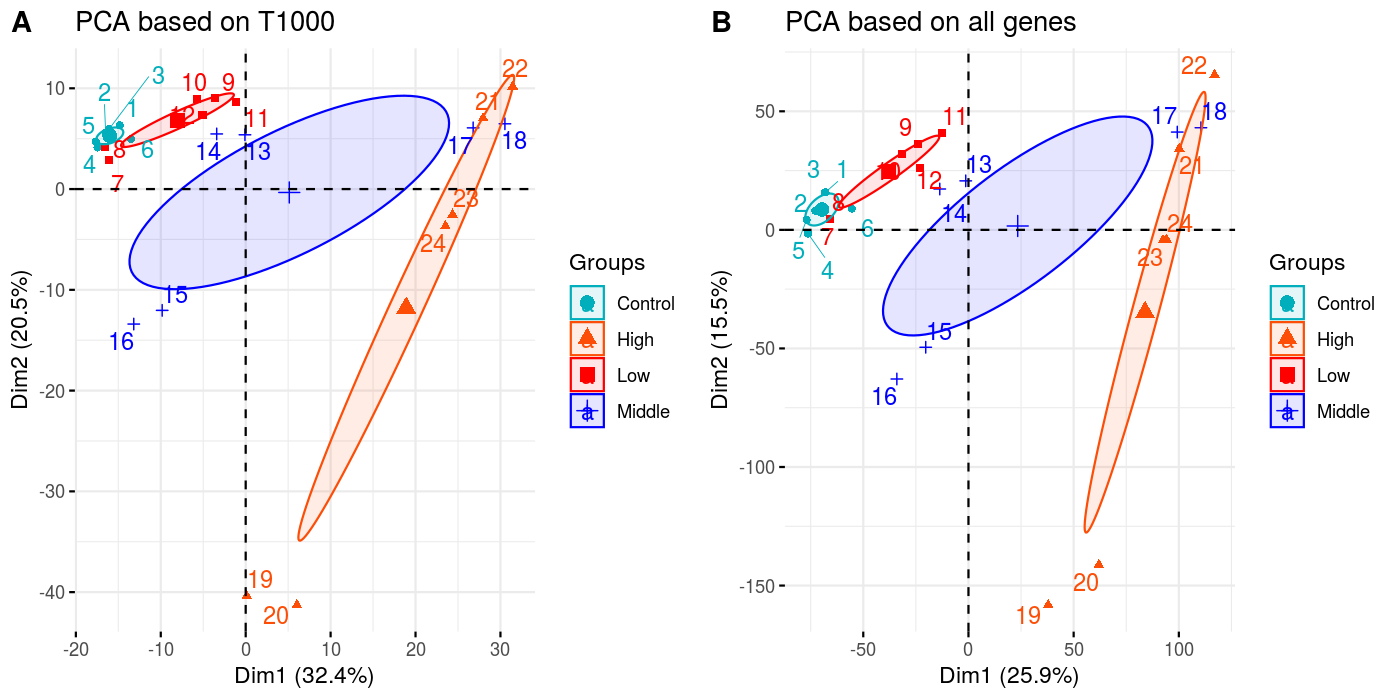

Supplement: Figure S5 [file peerj-07-7975-s005.zip › Supplementary_Figures_S5/nitrofurantoin.Human.in_vitro.Liver.tiff]

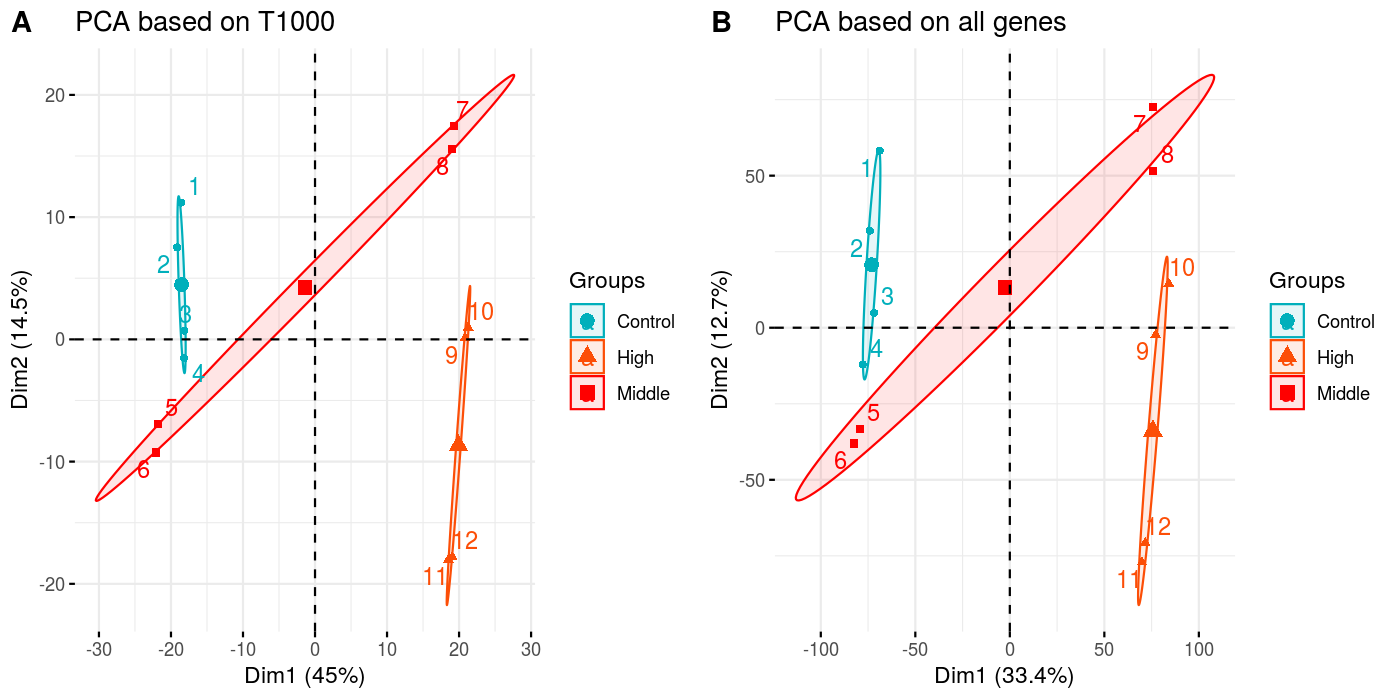

Supplement: Figure S5 [file peerj-07-7975-s005.zip › Supplementary_Figures_S5/ethionamide.Human.in_vitro.Liver.tiff]

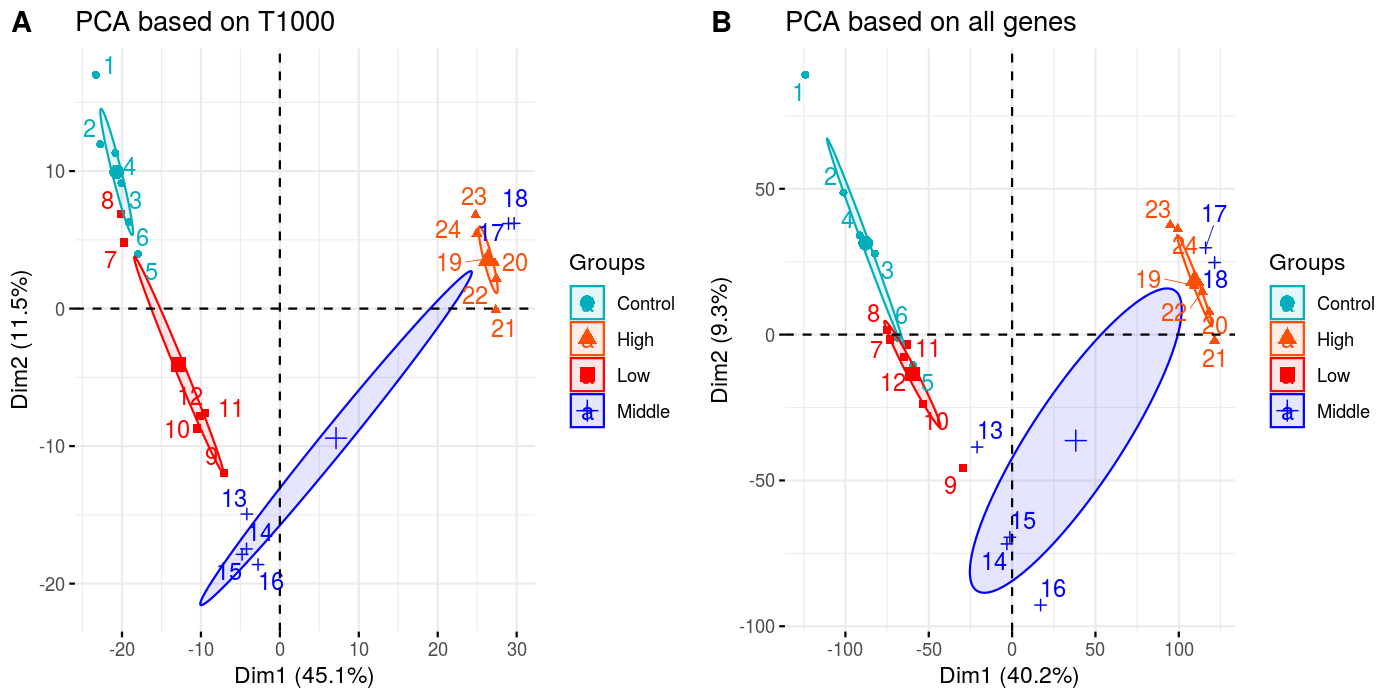

Supplement: Figure S5 [file peerj-07-7975-s005.zip › Supplementary_Figures_S5/rifampicin.Human.in_vitro.Liver.tiff]

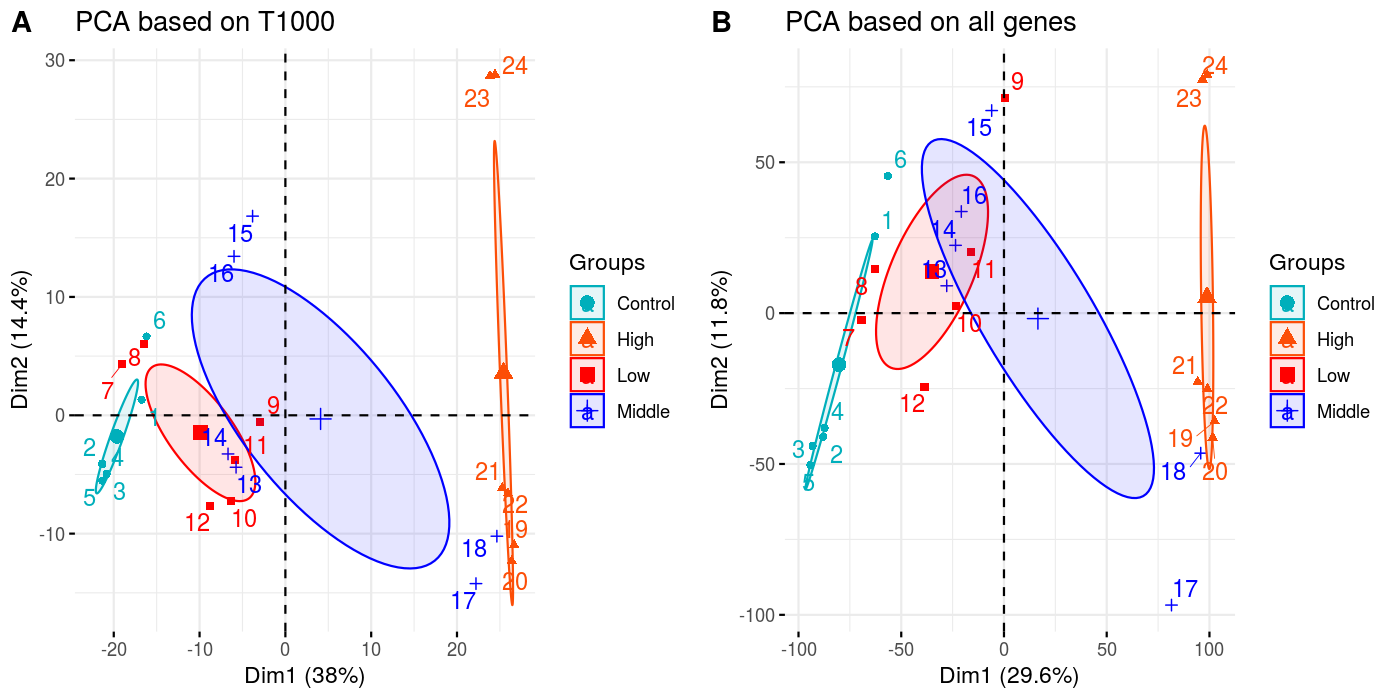

Supplement: Figure S5 [file peerj-07-7975-s005.zip › Supplementary_Figures_S5/adapin.Human.in_vitro.Liver.tiff]

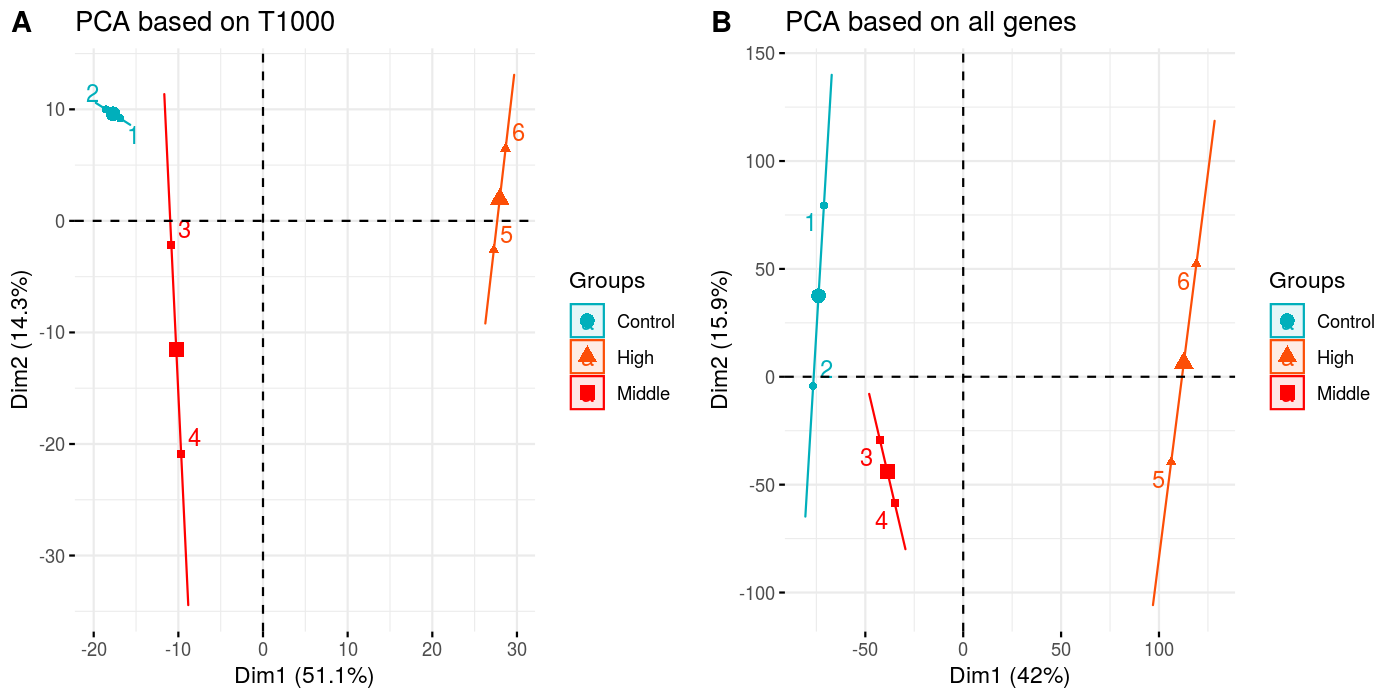

Supplement: Figure S5 [file peerj-07-7975-s005.zip › Supplementary_Figures_S5/buspirone.Human.in_vitro.Liver.tiff]

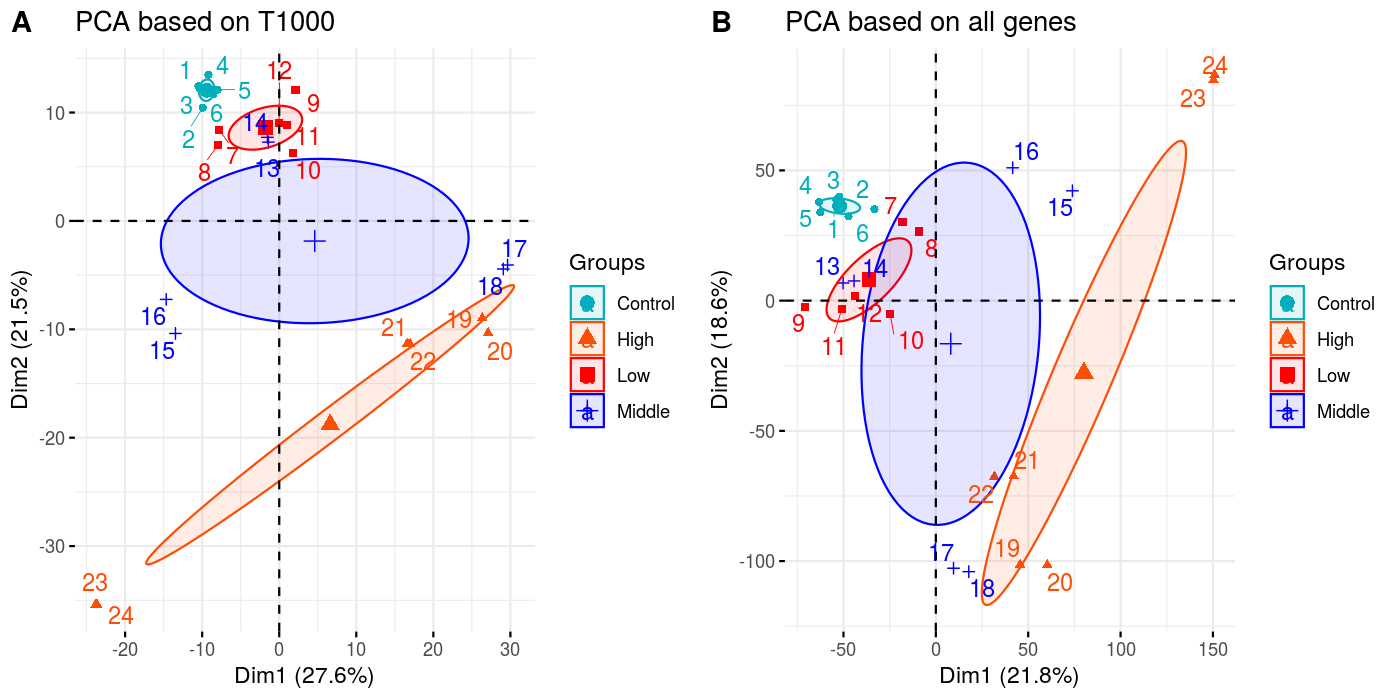

Supplement: Figure S5 [file peerj-07-7975-s005.zip › Supplementary_Figures_S5/omeprazole.Human.in_vitro.Liver.tiff]

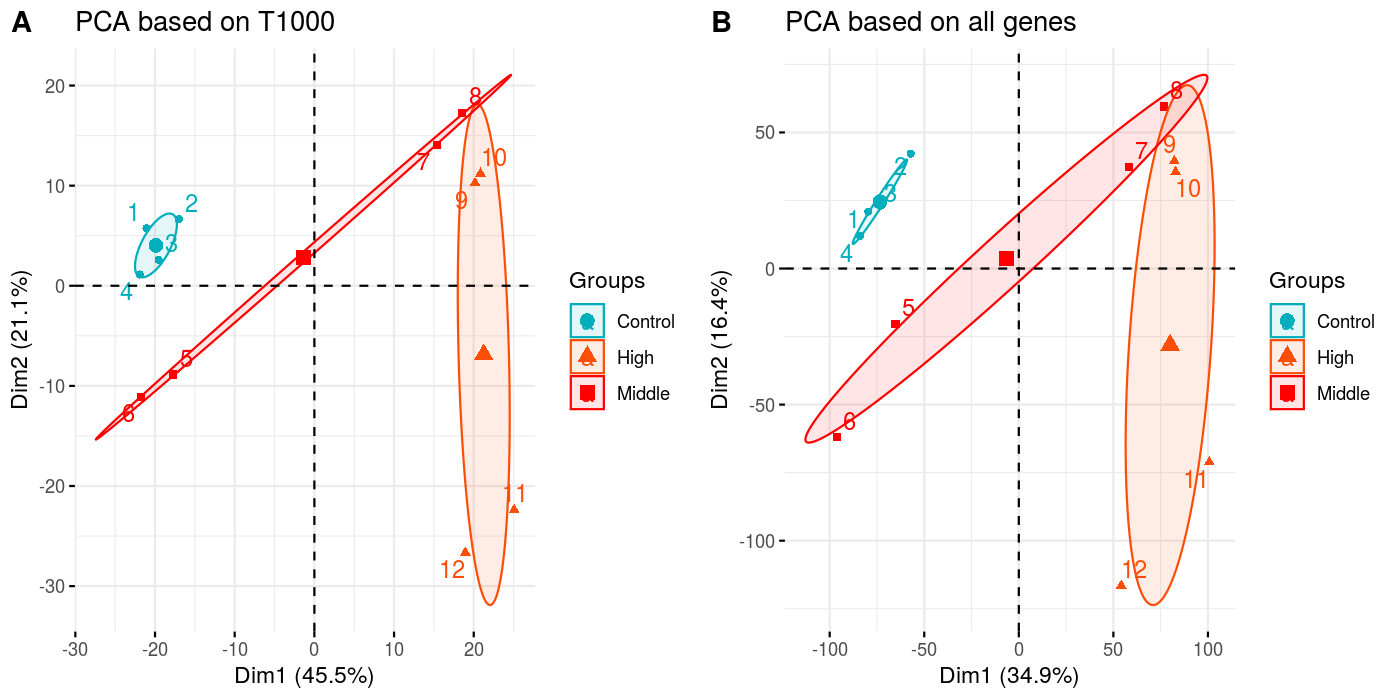

Supplement: Figure S5 [file peerj-07-7975-s005.zip › Supplementary_Figures_S5/hydroxyzine.Human.in_vitro.Liver.tiff]

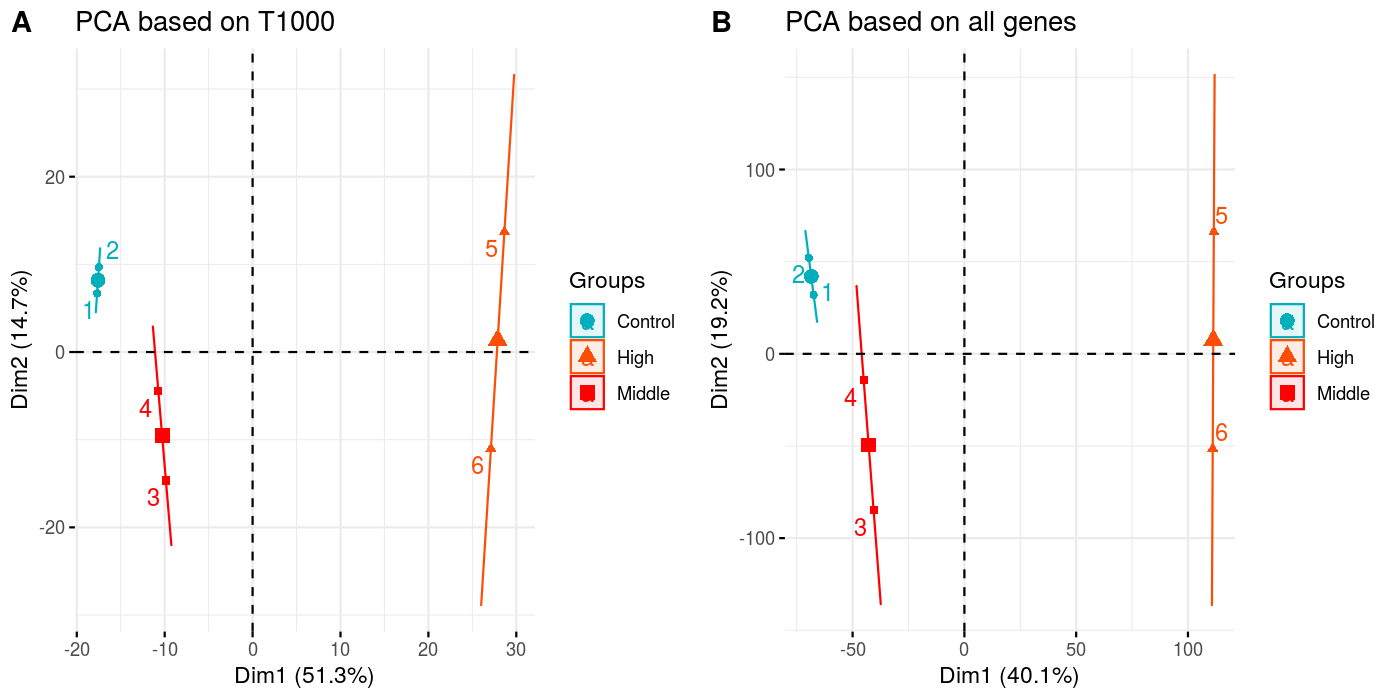

Supplement: Figure S5 [file peerj-07-7975-s005.zip › Supplementary_Figures_S5/clozapine.Human.in_vitro.Liver.tiff]

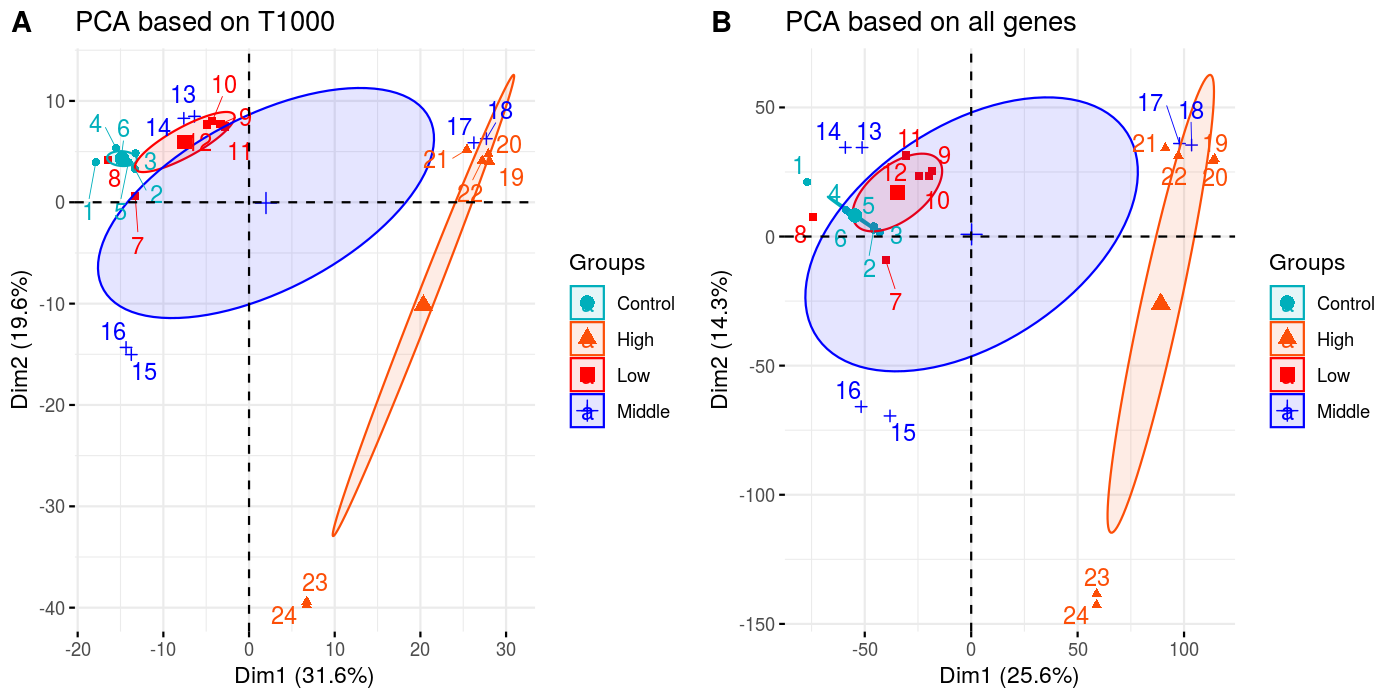

Supplement: Figure S5 [file peerj-07-7975-s005.zip › Supplementary_Figures_S5/diclofenac.Human.in_vitro.Liver.tiff]

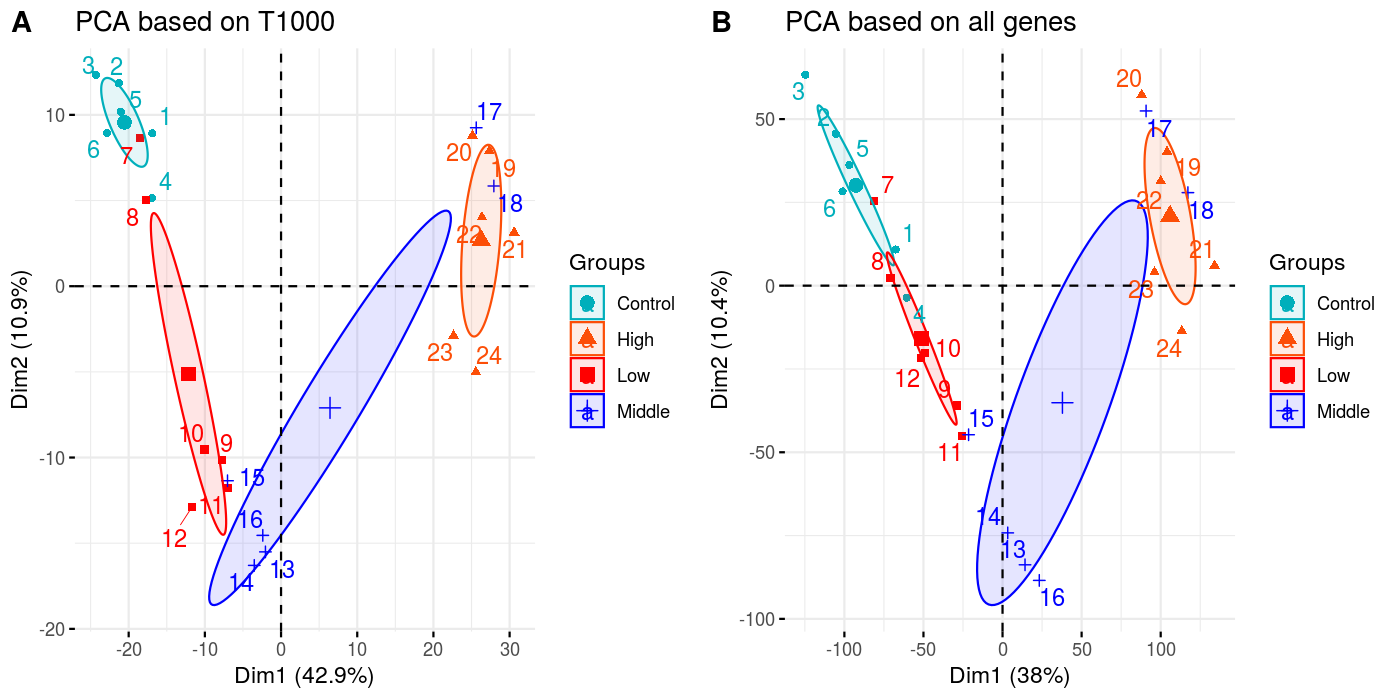

Supplement: Figure S5 [file peerj-07-7975-s005.zip › Supplementary_Figures_S5/coumarin.Human.in_vitro.Liver.tiff]

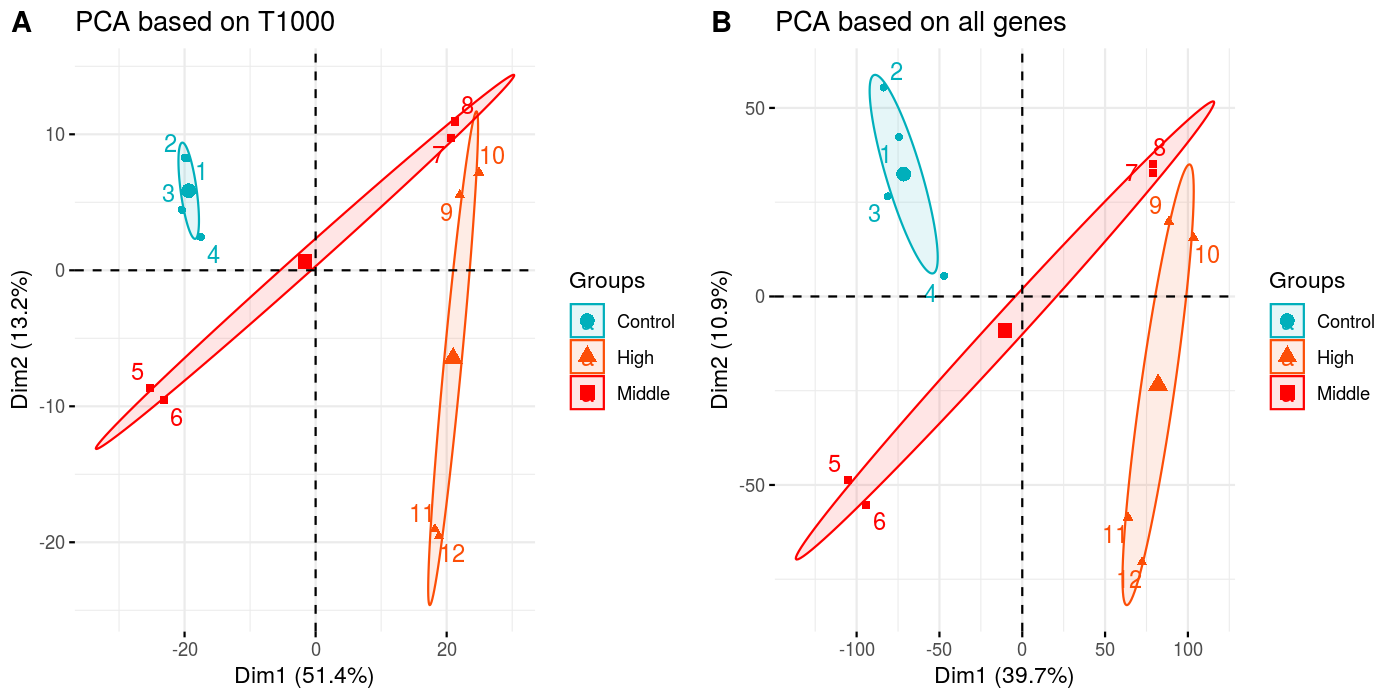

Supplement: Figure S5 [file peerj-07-7975-s005.zip › Supplementary_Figures_S5/moxisylyte.Human.in_vitro.Liver.tiff]

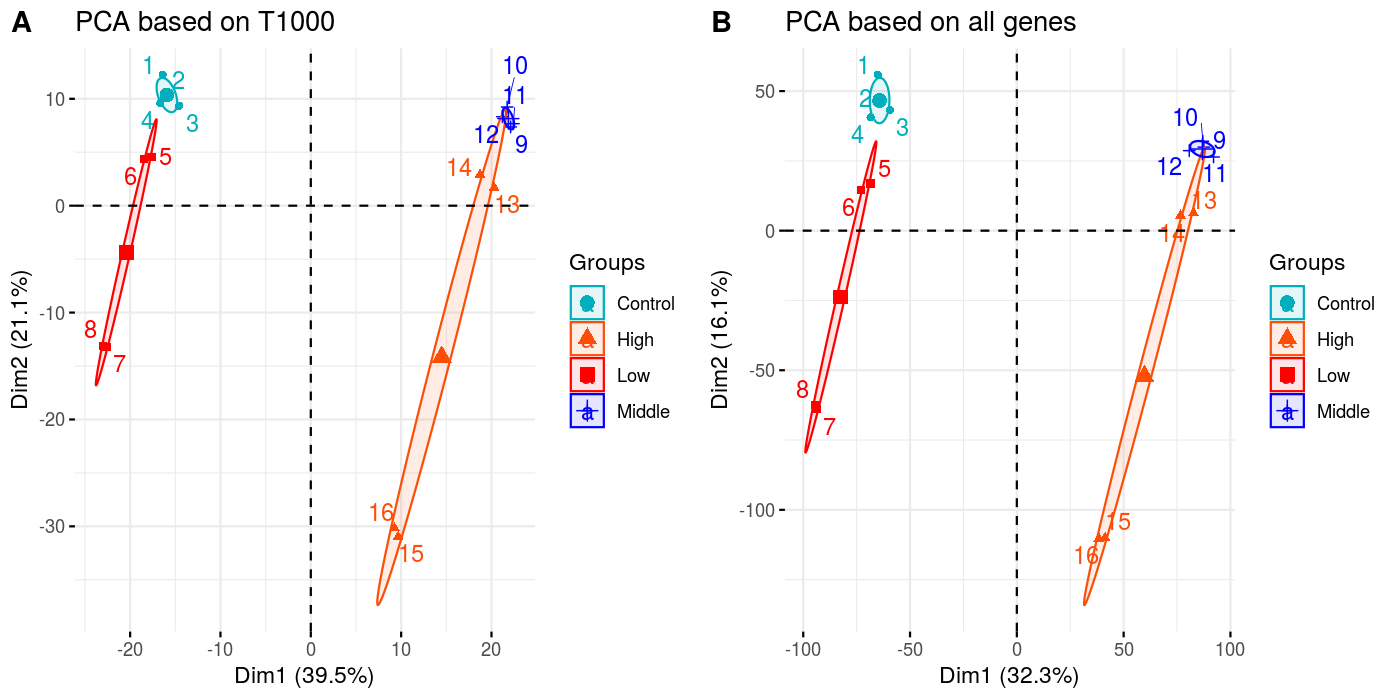

Supplement: Figure S5 [file peerj-07-7975-s005.zip › Supplementary_Figures_S5/propranolol.Human.in_vitro.Liver.tiff]

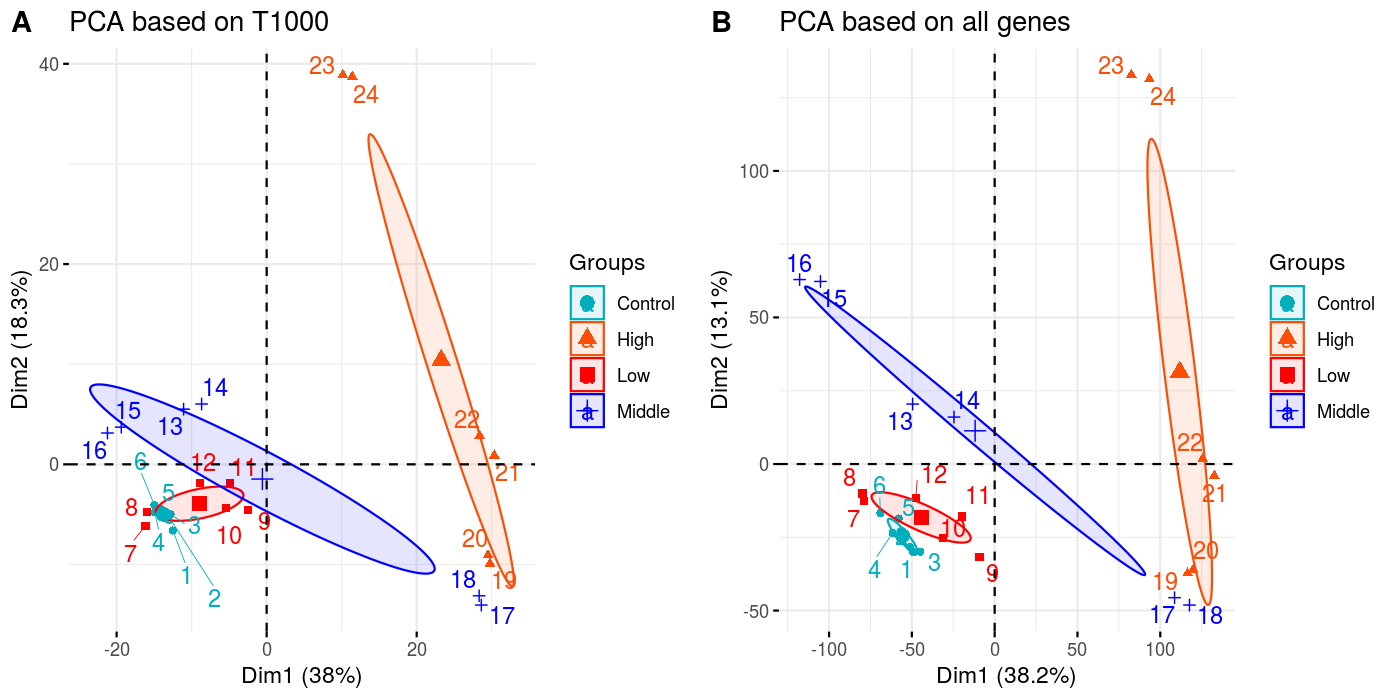

Supplement: Figure S5 [file peerj-07-7975-s005.zip › Supplementary_Figures_S5/acetaminophen.Human.in_vitro.Liver.tiff]

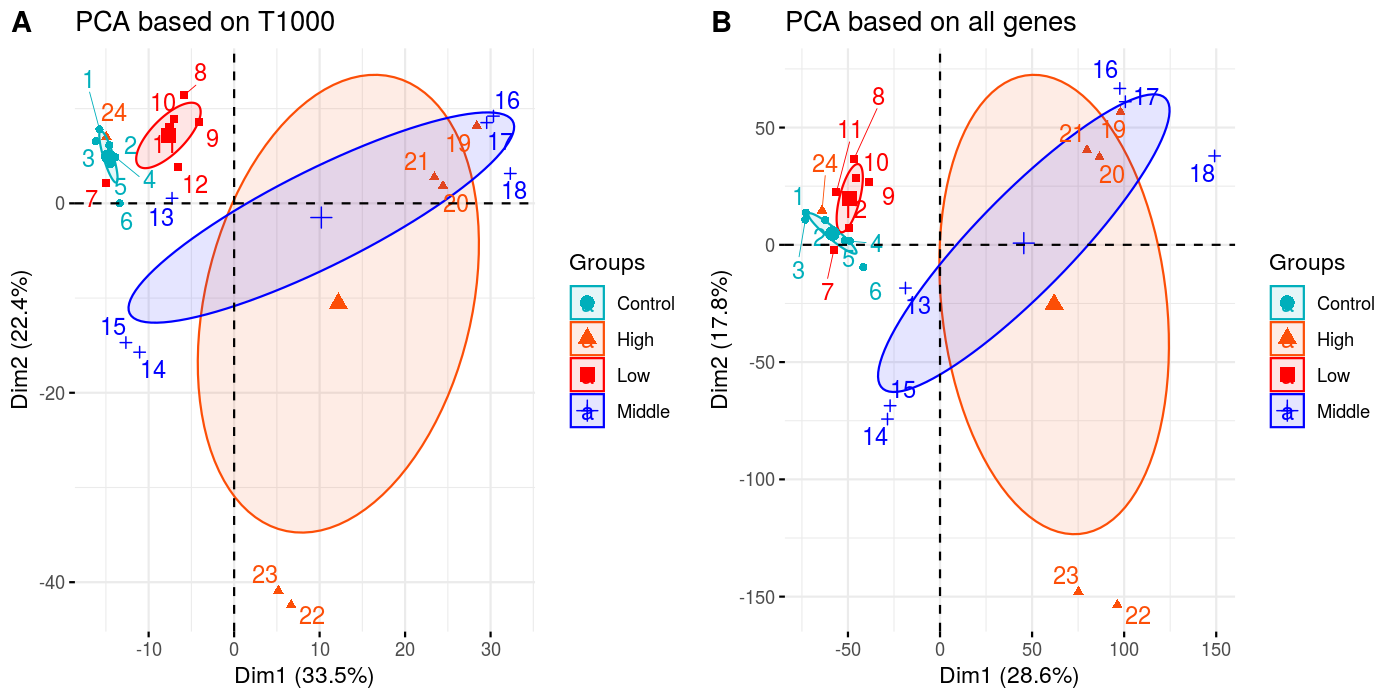

Supplement: Figure S5 [file peerj-07-7975-s005.zip › Supplementary_Figures_S5/methapyrilene.Human.in_vitro.Liver.tiff]

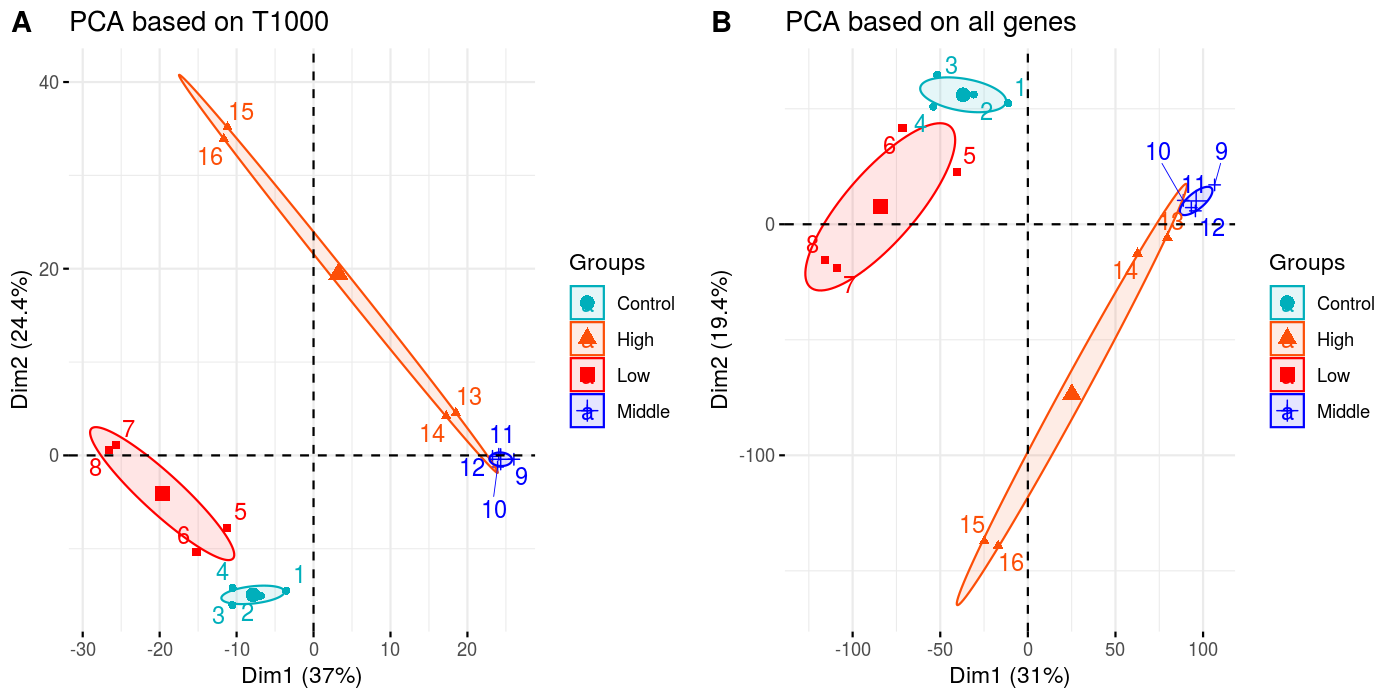

Supplement: Figure S5 [file peerj-07-7975-s005.zip › Supplementary_Figures_S5/butylated_hydroxyanisole.Human.in_vitro.Liver.tiff]

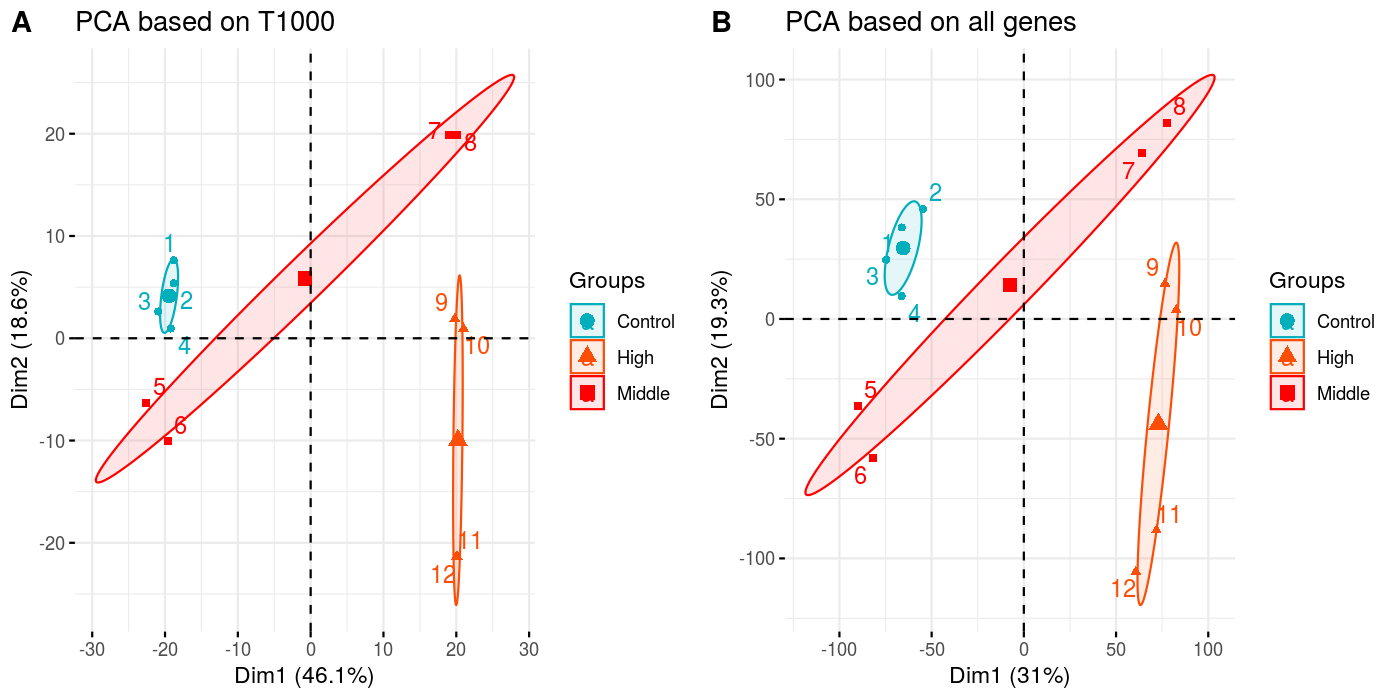

Supplement: Figure S5 [file peerj-07-7975-s005.zip › Supplementary_Figures_S5/tannic_acid.Human.in_vitro.Liver.tiff]

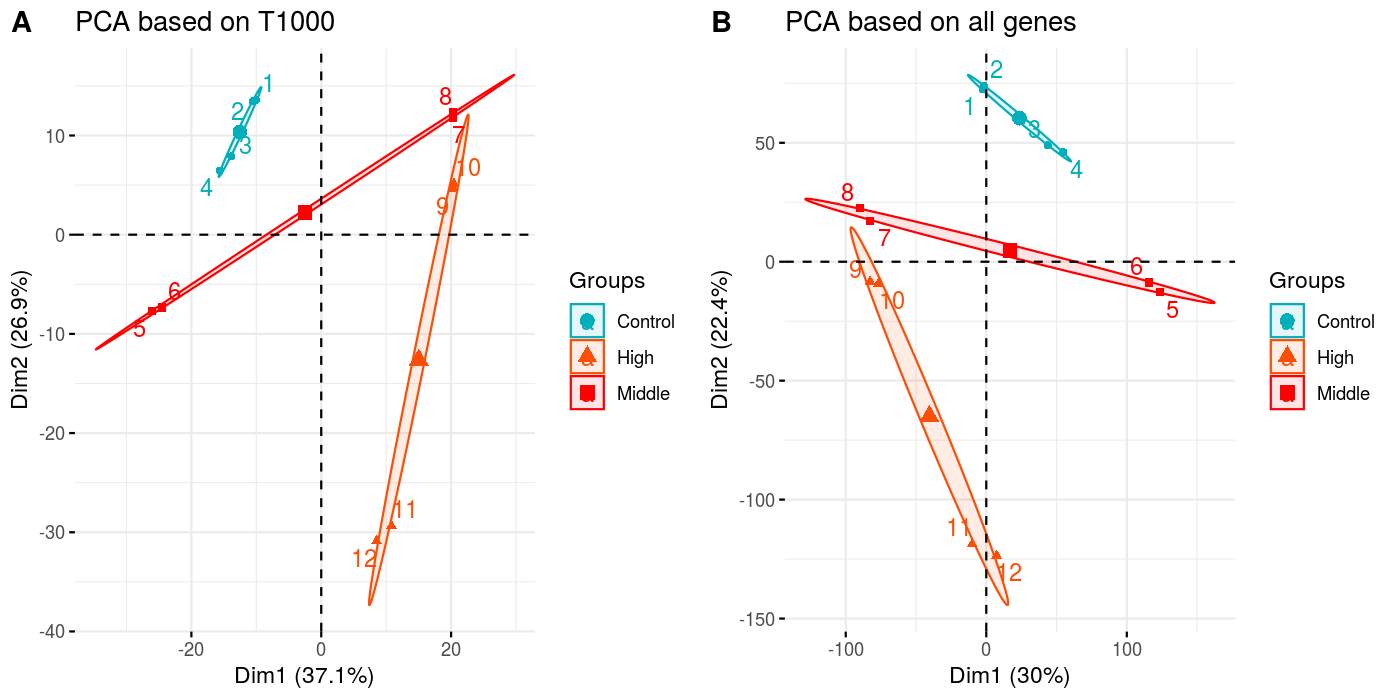

Supplement: Figure S5 [file peerj-07-7975-s005.zip › Supplementary_Figures_S5/sulpiride.Human.in_vitro.Liver.tiff]

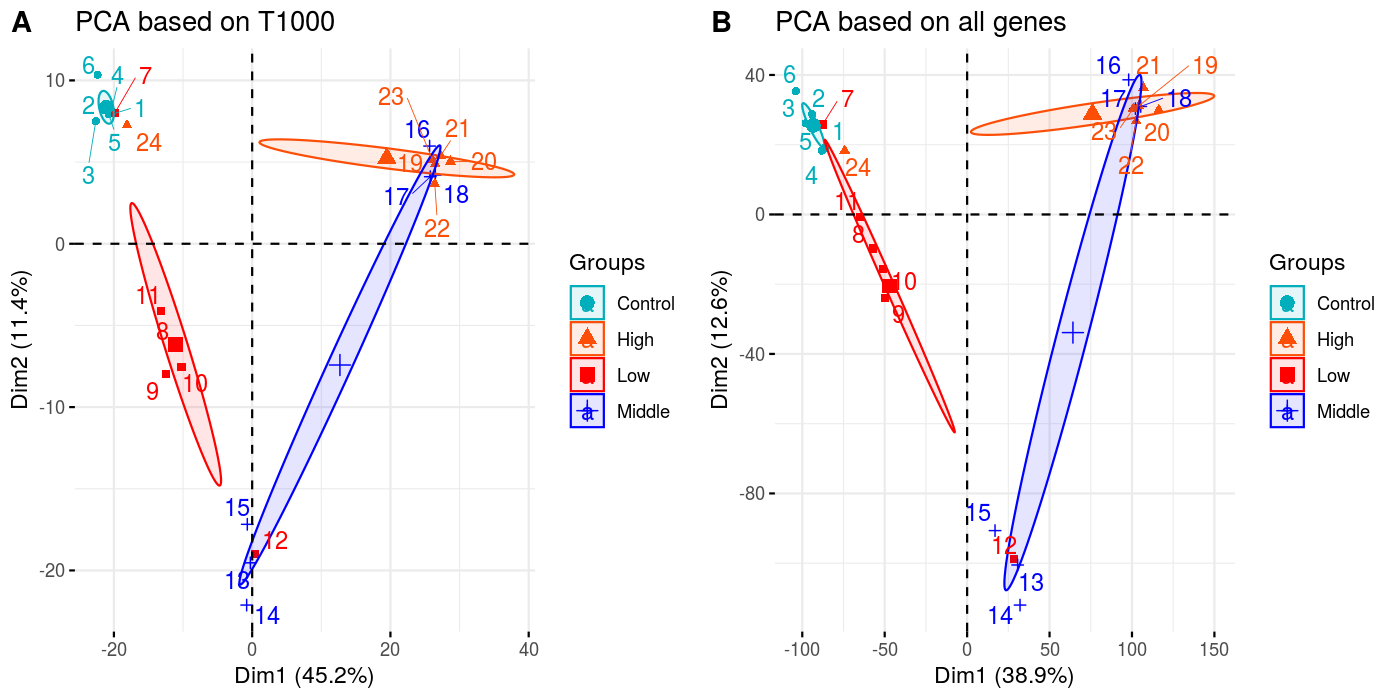

Supplement: Figure S5 [file peerj-07-7975-s005.zip › Supplementary_Figures_S5/WY-14643.Human.in_vitro.Liver.tiff]

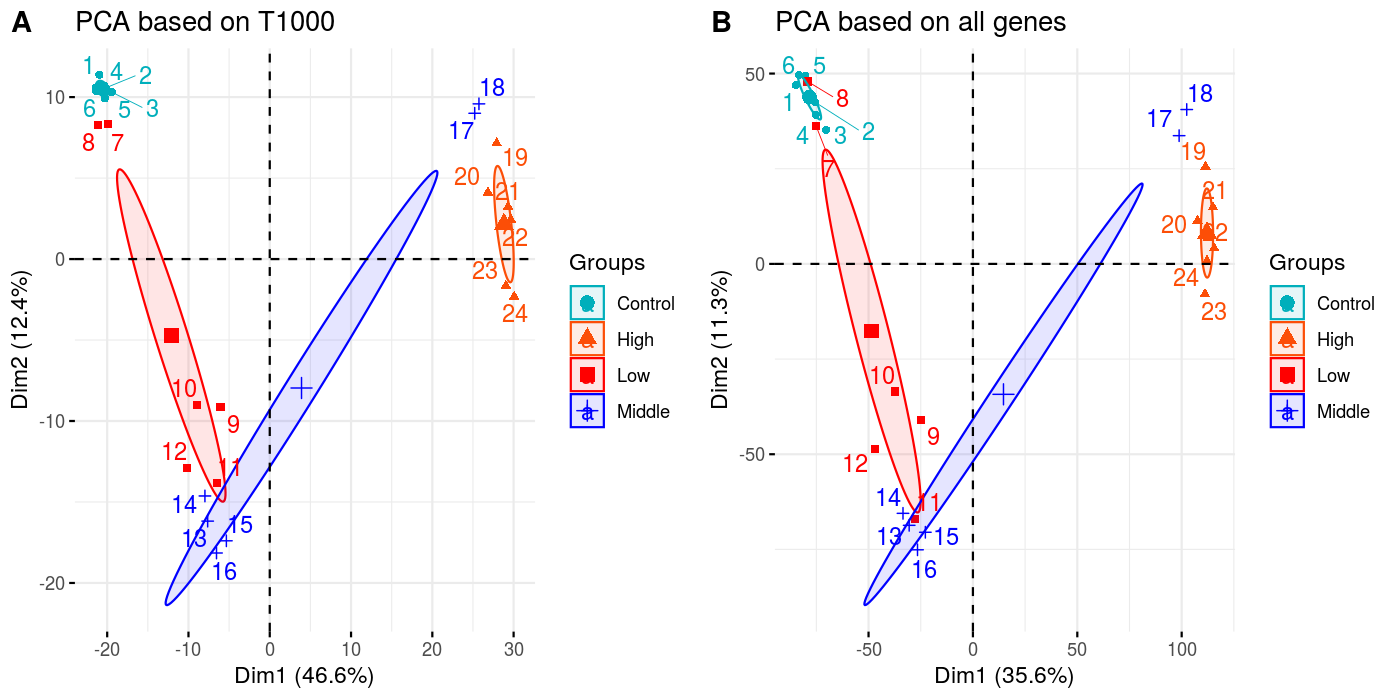

Supplement: Figure S5 [file peerj-07-7975-s005.zip › Supplementary_Figures_S5/hepatocyte_growth_factor,_human.Human.in_vitro.Liver.tiff]

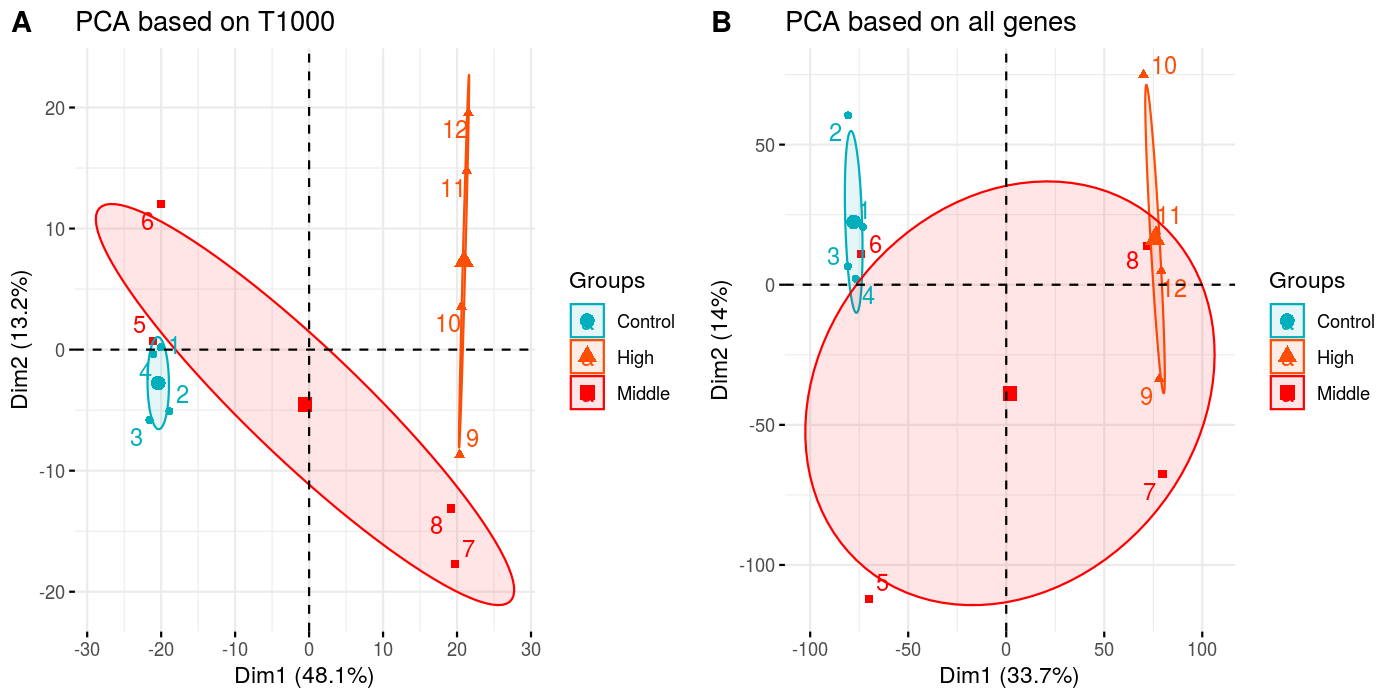

Supplement: Figure S5 [file peerj-07-7975-s005.zip › Supplementary_Figures_S5/amitriptyline.Human.in_vitro.Liver.tiff]

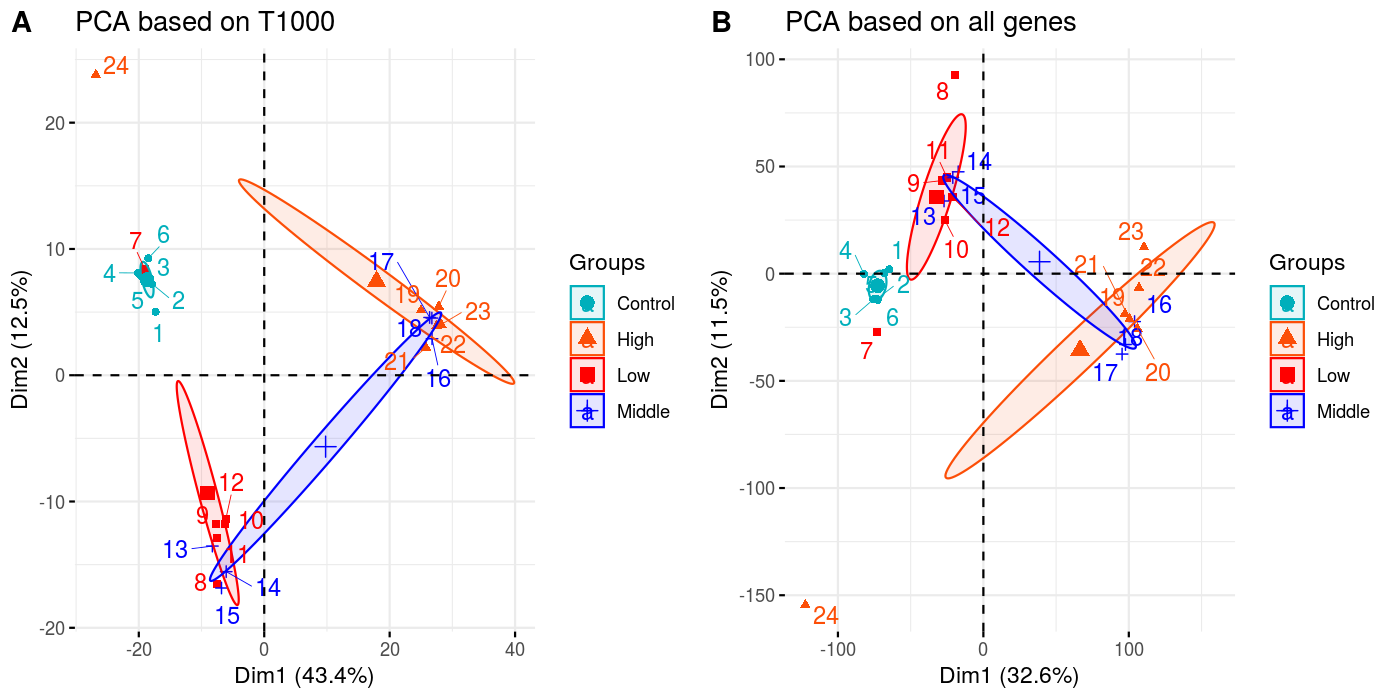

Supplement: Figure S5 [file peerj-07-7975-s005.zip › Supplementary_Figures_S5/cimetidine.Human.in_vitro.Liver.tiff]

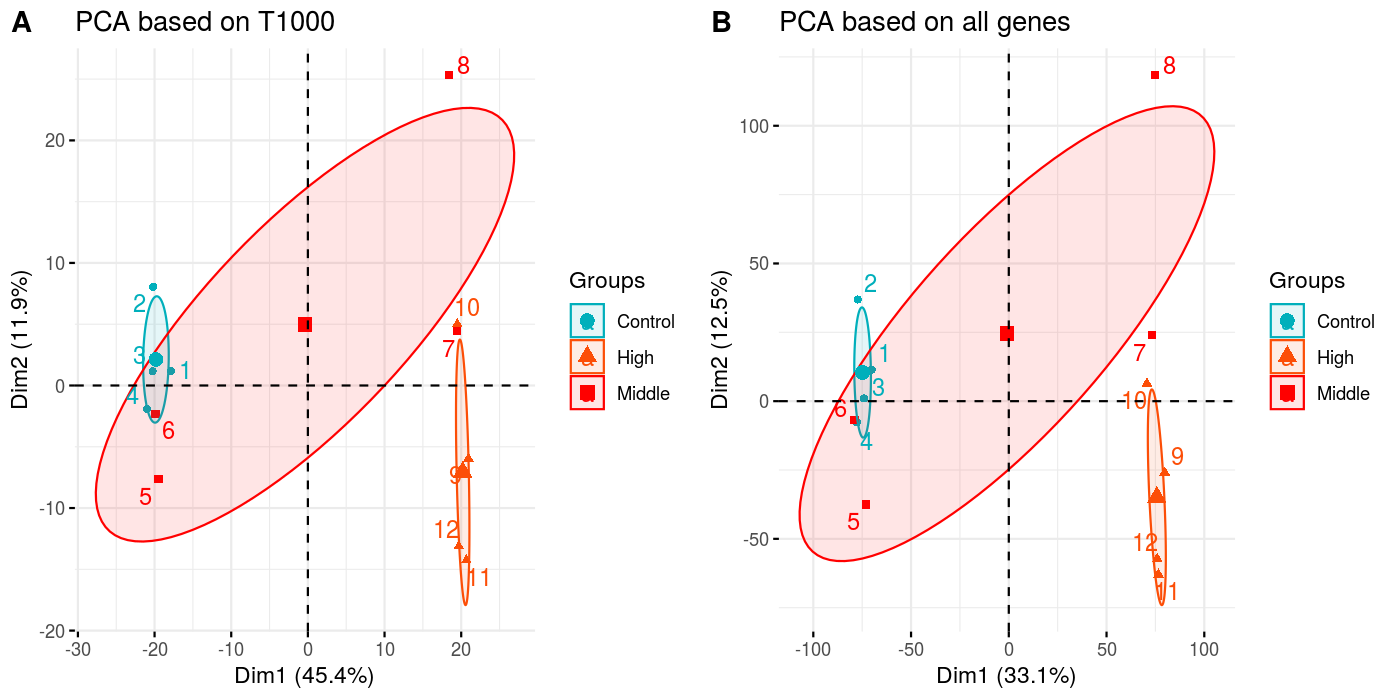

Supplement: Figure S5 [file peerj-07-7975-s005.zip › Supplementary_Figures_S5/ticlopidine.Human.in_vitro.Liver.tiff]

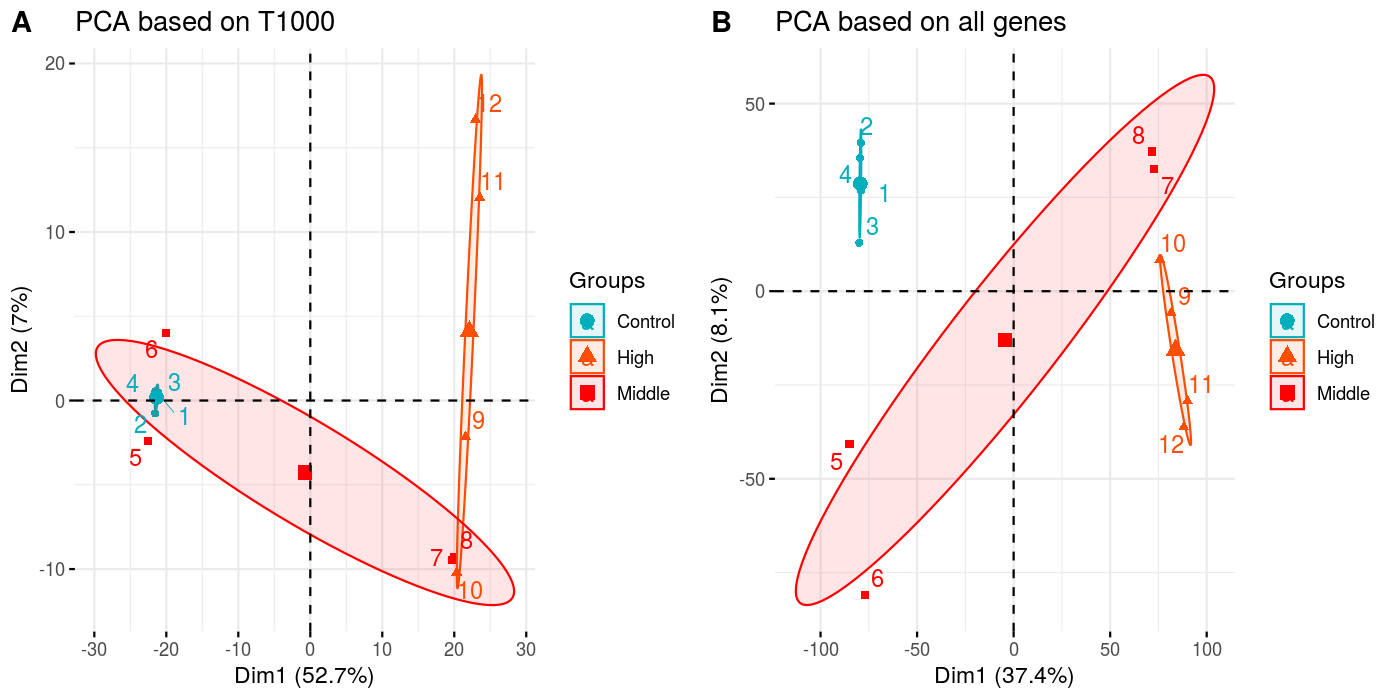

Supplement: Figure S5 [file peerj-07-7975-s005.zip › Supplementary_Figures_S5/tiopronin.Human.in_vitro.Liver.tiff]

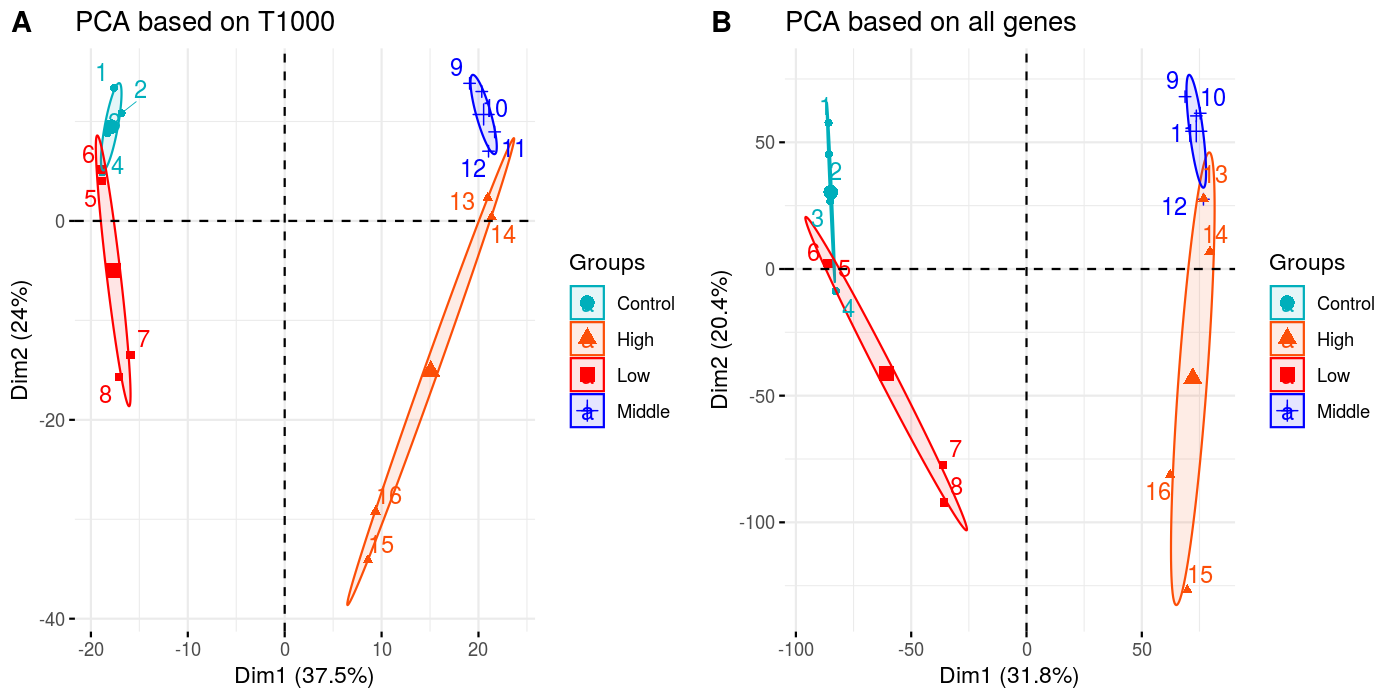

Supplement: Figure S5 [file peerj-07-7975-s005.zip › Supplementary_Figures_S5/rosiglitazone_maleate.Human.in_vitro.Liver.tiff]

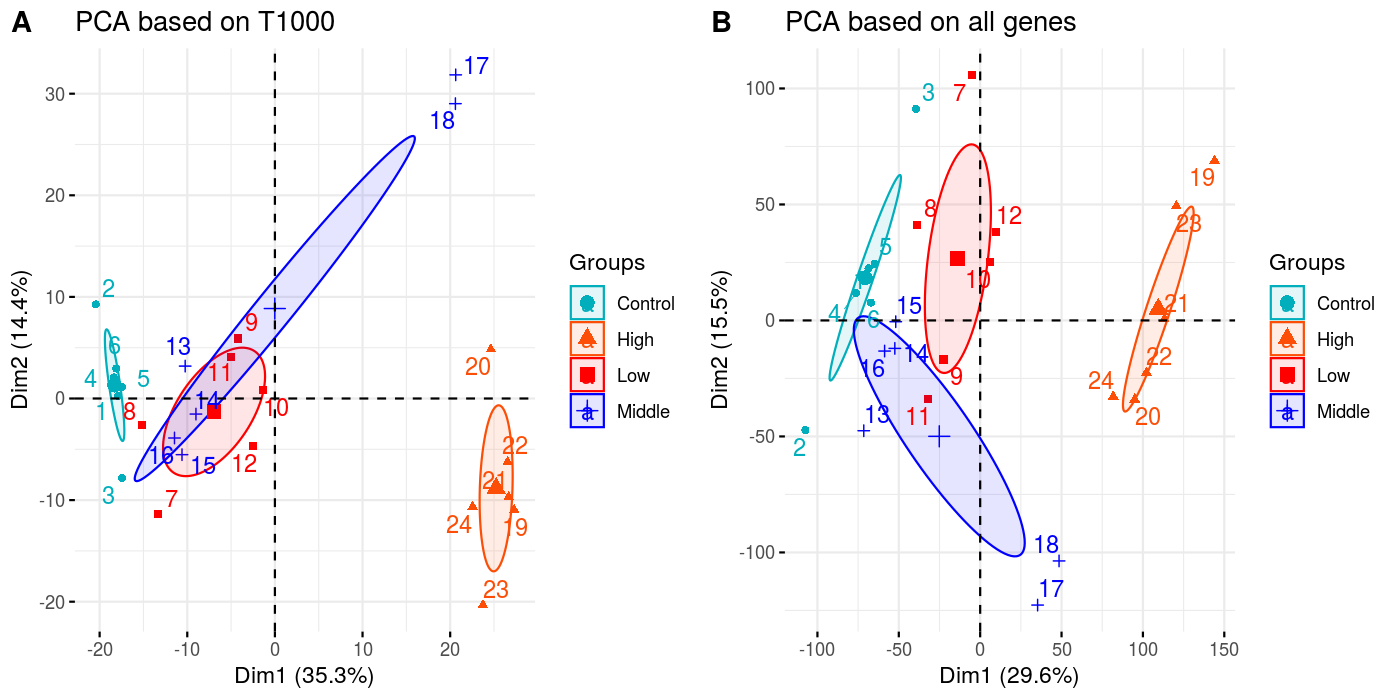

Supplement: Figure S5 [file peerj-07-7975-s005.zip › Supplementary_Figures_S5/azathioprine.Human.in_vitro.Liver.tiff]

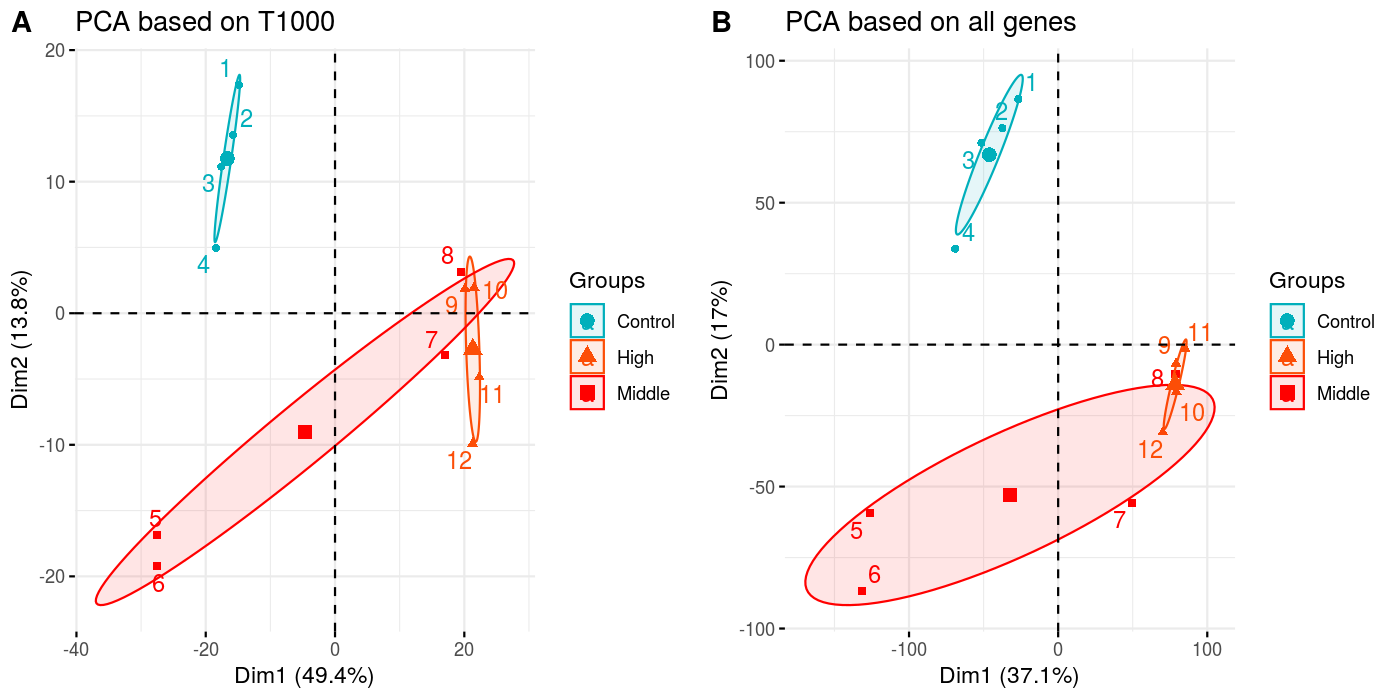

Supplement: Figure S5 [file peerj-07-7975-s005.zip › Supplementary_Figures_S5/ibuprofen.Human.in_vitro.Liver.tiff]

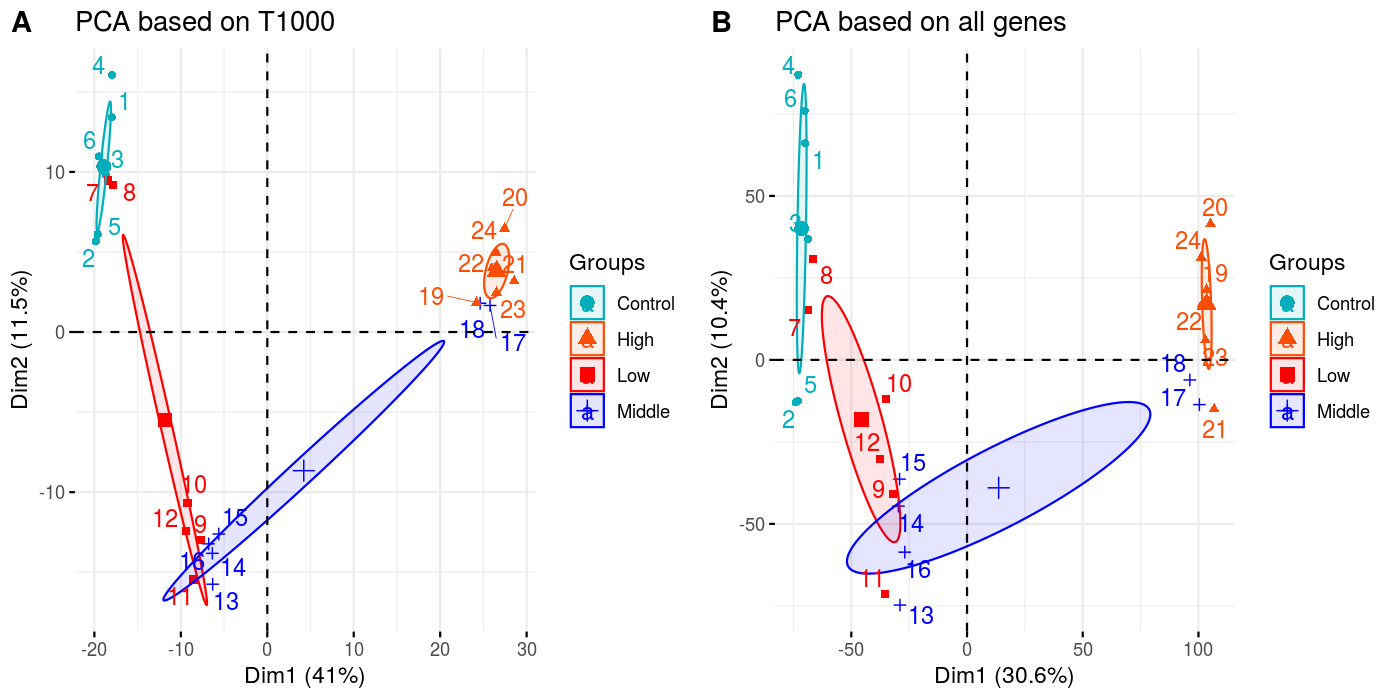

Supplement: Figure S5 [file peerj-07-7975-s005.zip › Supplementary_Figures_S5/bromobenzene.Human.in_vitro.Liver.tiff]

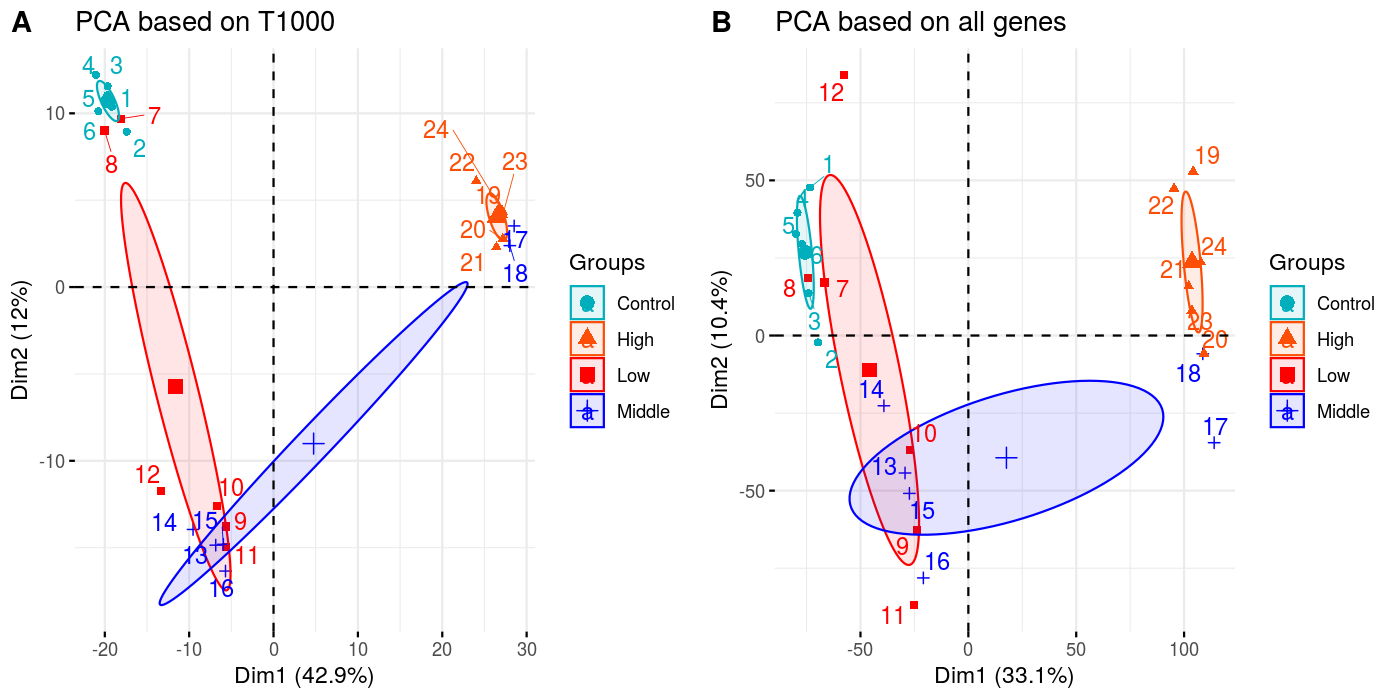

Supplement: Figure S5 [file peerj-07-7975-s005.zip › Supplementary_Figures_S5/glibenclamide.Human.in_vitro.Liver.tiff]

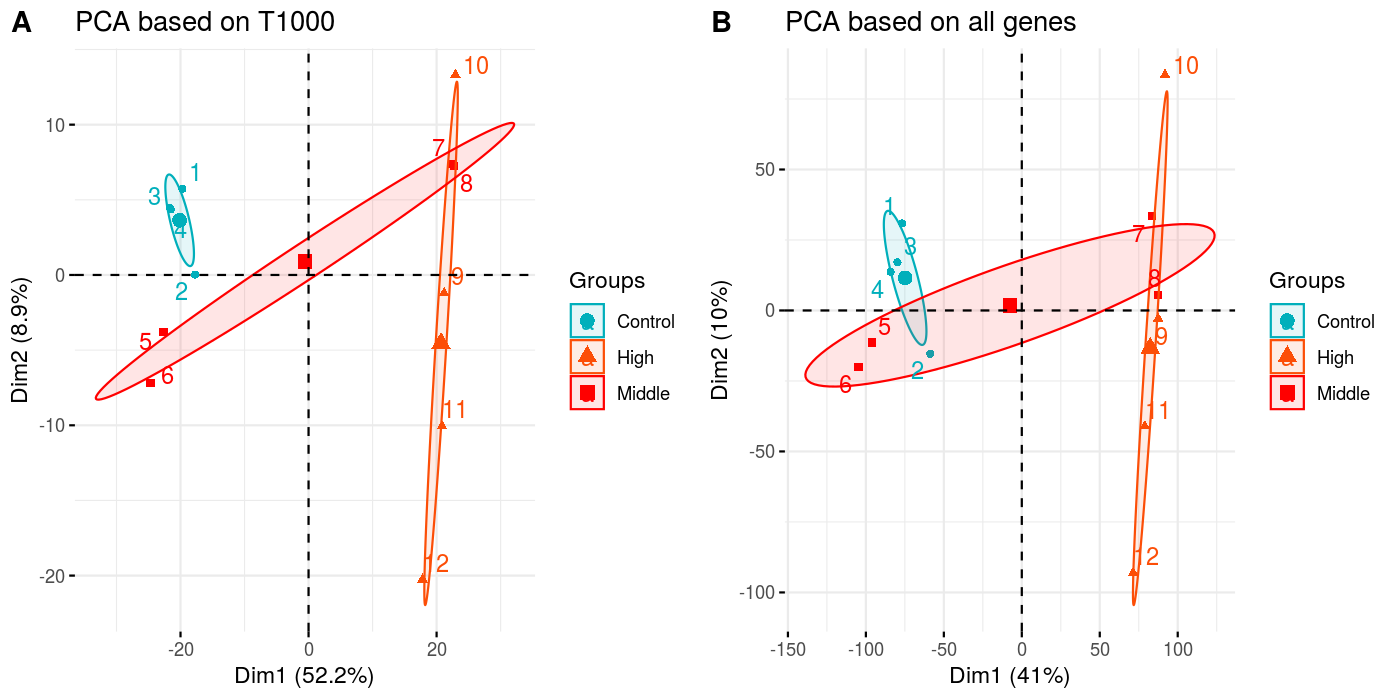

Supplement: Figure S5 [file peerj-07-7975-s005.zip › Supplementary_Figures_S5/tamoxifen.Human.in_vitro.Liver.tiff]

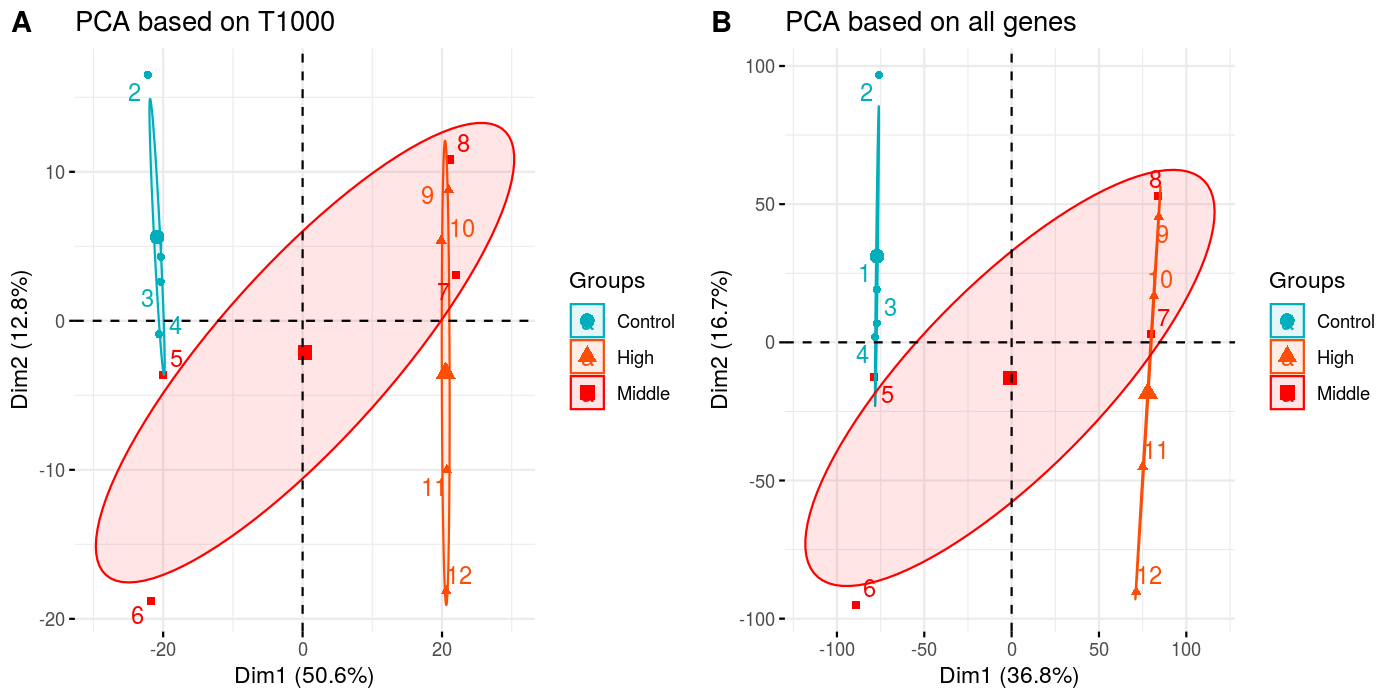

Supplement: Figure S5 [file peerj-07-7975-s005.zip › Supplementary_Figures_S5/meloxicam.Human.in_vitro.Liver.tiff]

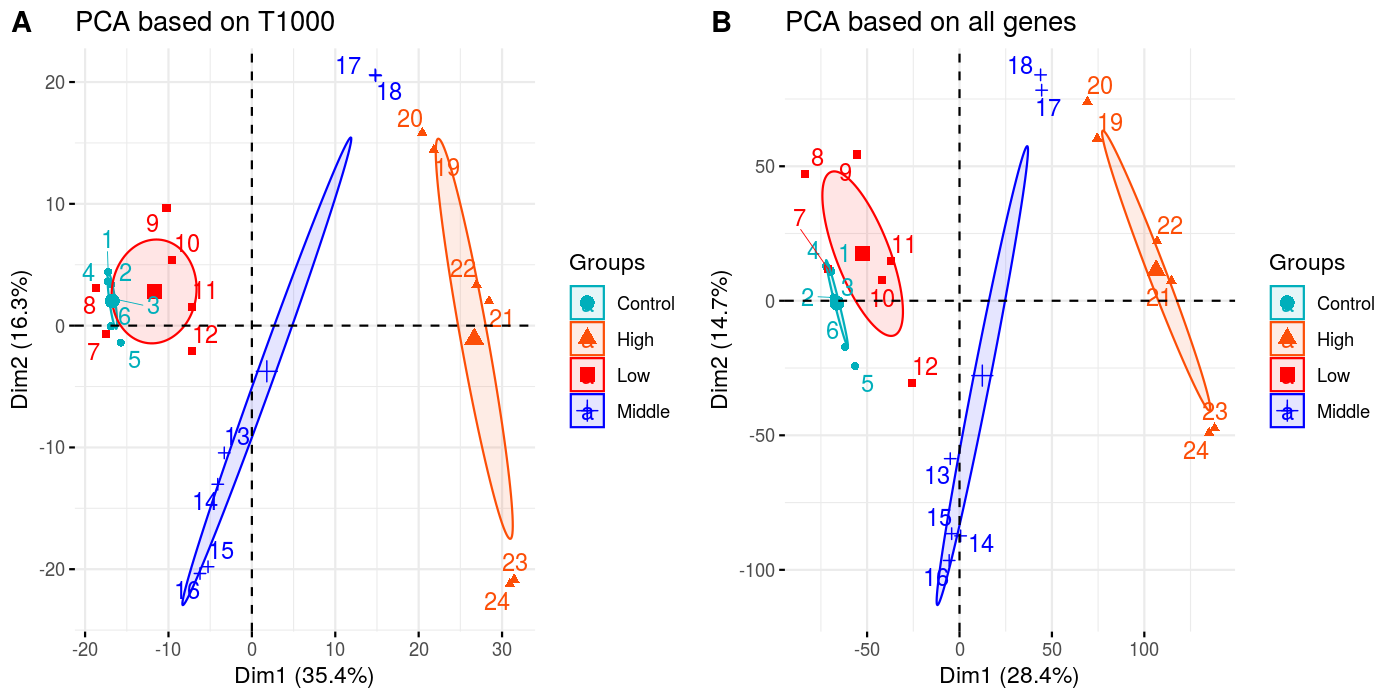

Supplement: Figure S5 [file peerj-07-7975-s005.zip › Supplementary_Figures_S5/valproic_acid.Human.in_vitro.Liver.tiff]

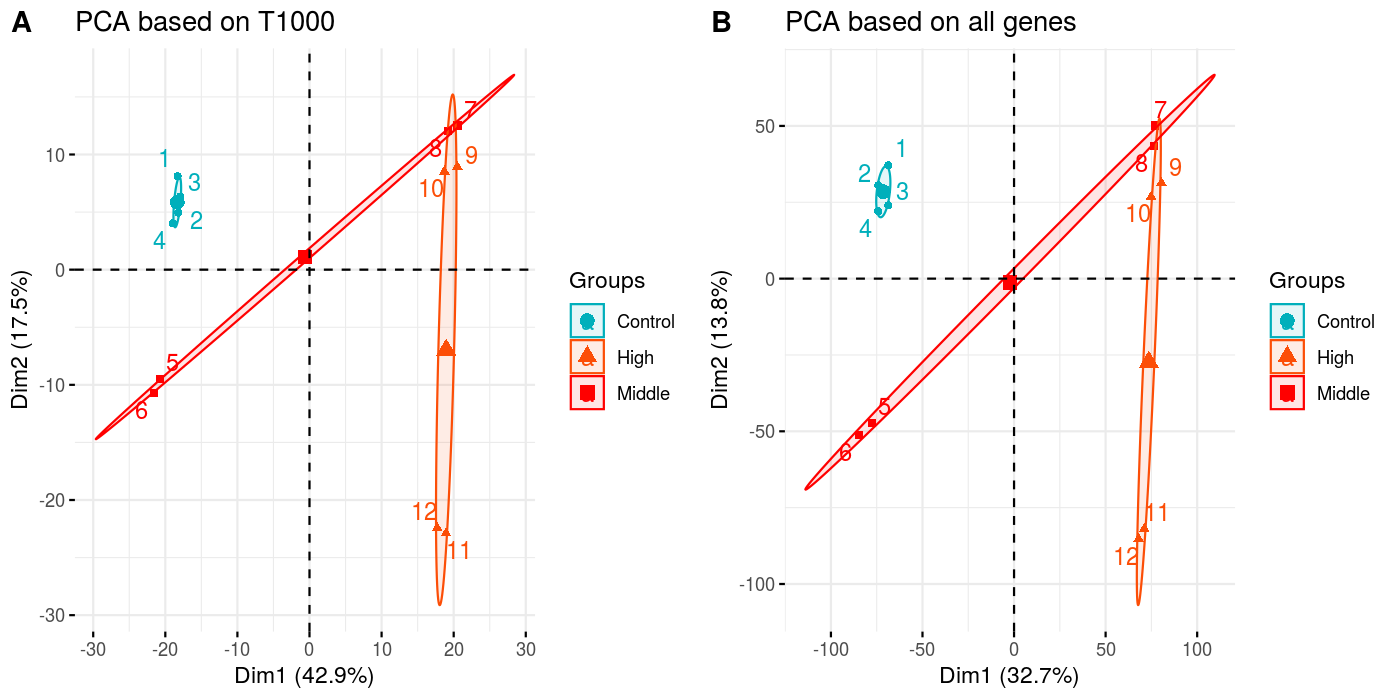

Supplement: Figure S5 [file peerj-07-7975-s005.zip › Supplementary_Figures_S5/mexiletine.Human.in_vitro.Liver.tiff]

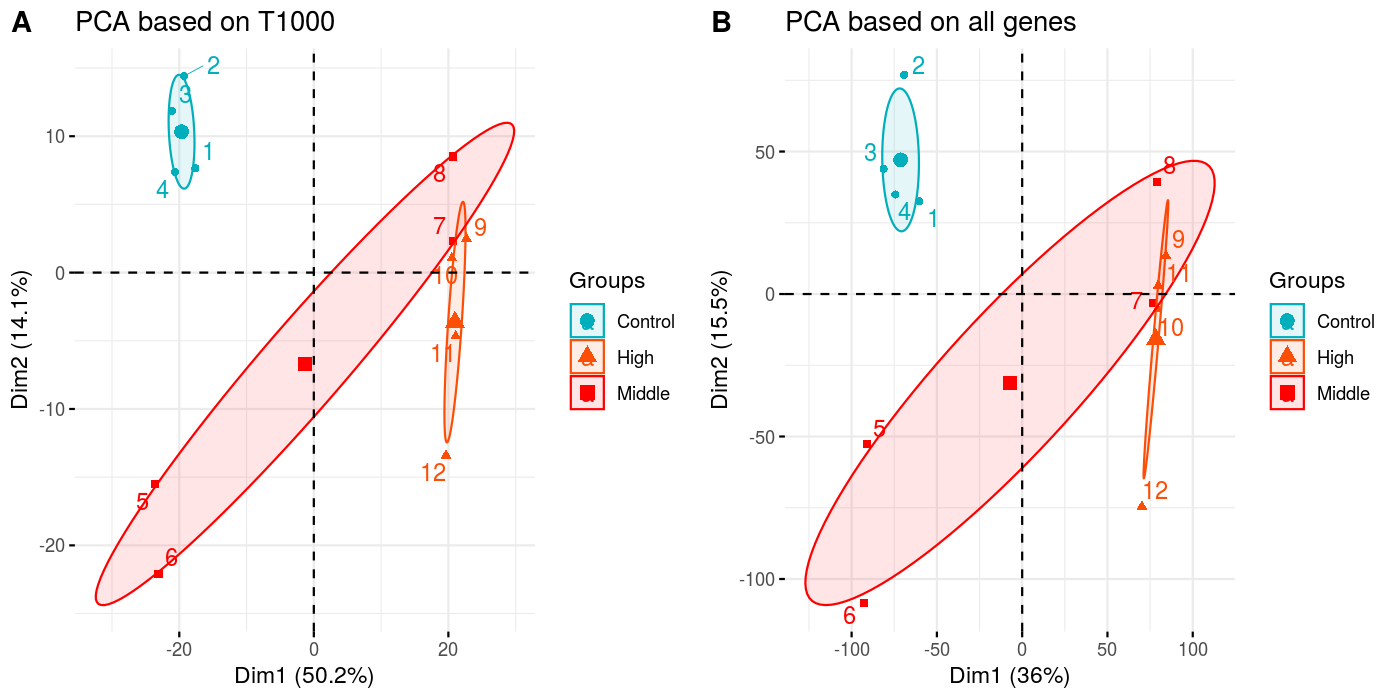

Supplement: Figure S5 [file peerj-07-7975-s005.zip › Supplementary_Figures_S5/mefenamic_acid.Human.in_vitro.Liver.tiff]

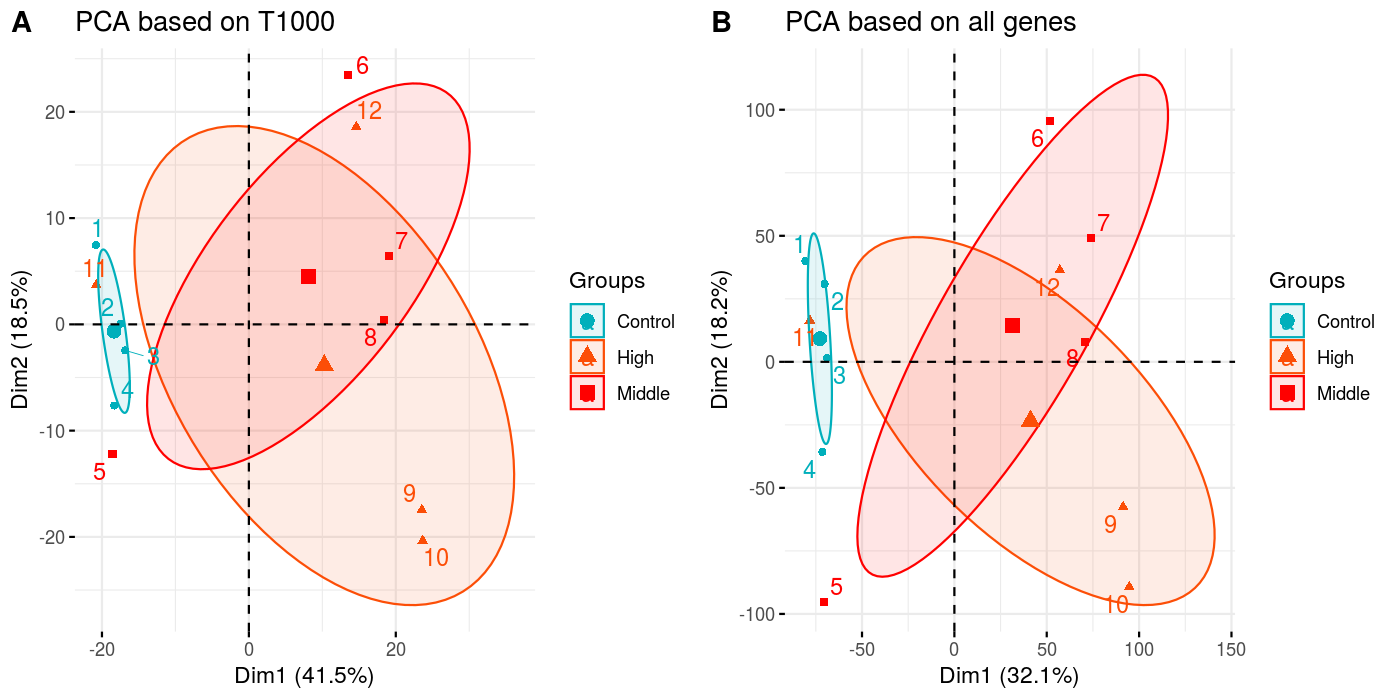

Supplement: Figure S5 [file peerj-07-7975-s005.zip › Supplementary_Figures_S5/simvastatin.Human.in_vitro.Liver.tiff]

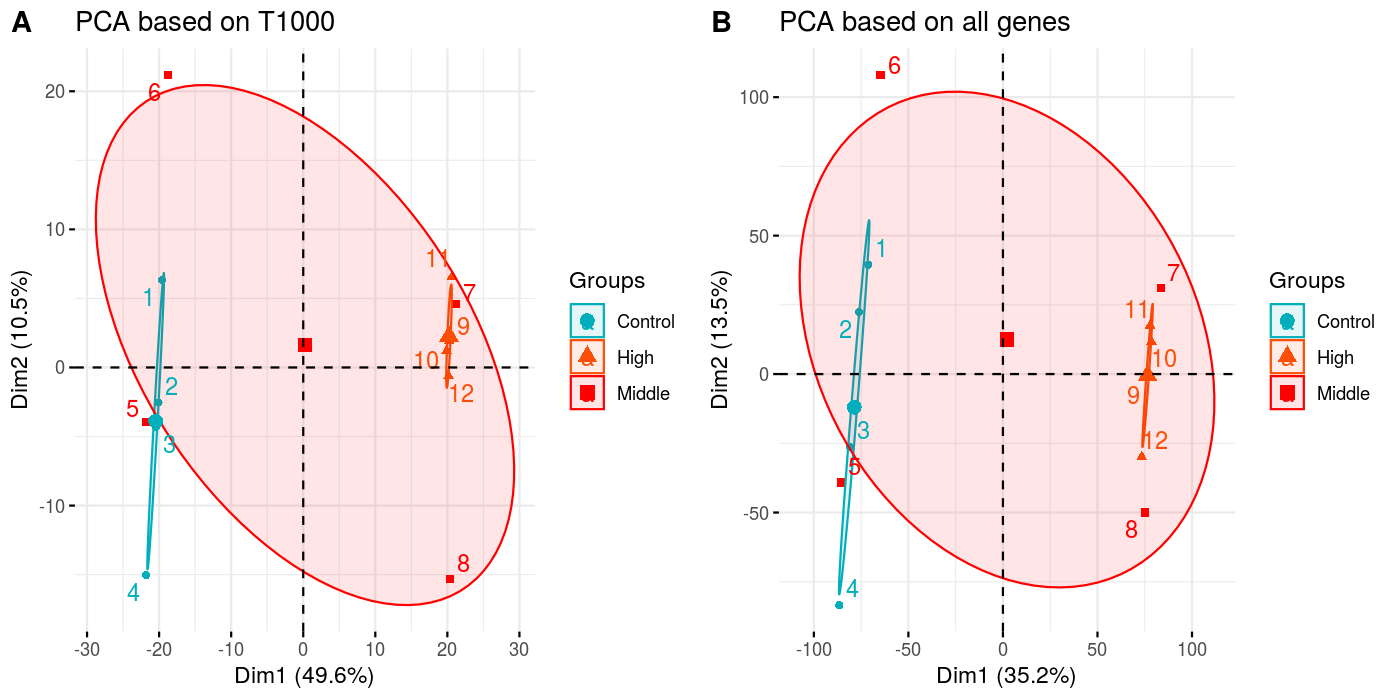

Supplement: Figure S5 [file peerj-07-7975-s005.zip › Supplementary_Figures_S5/methyldopa.Human.in_vitro.Liver.tiff]

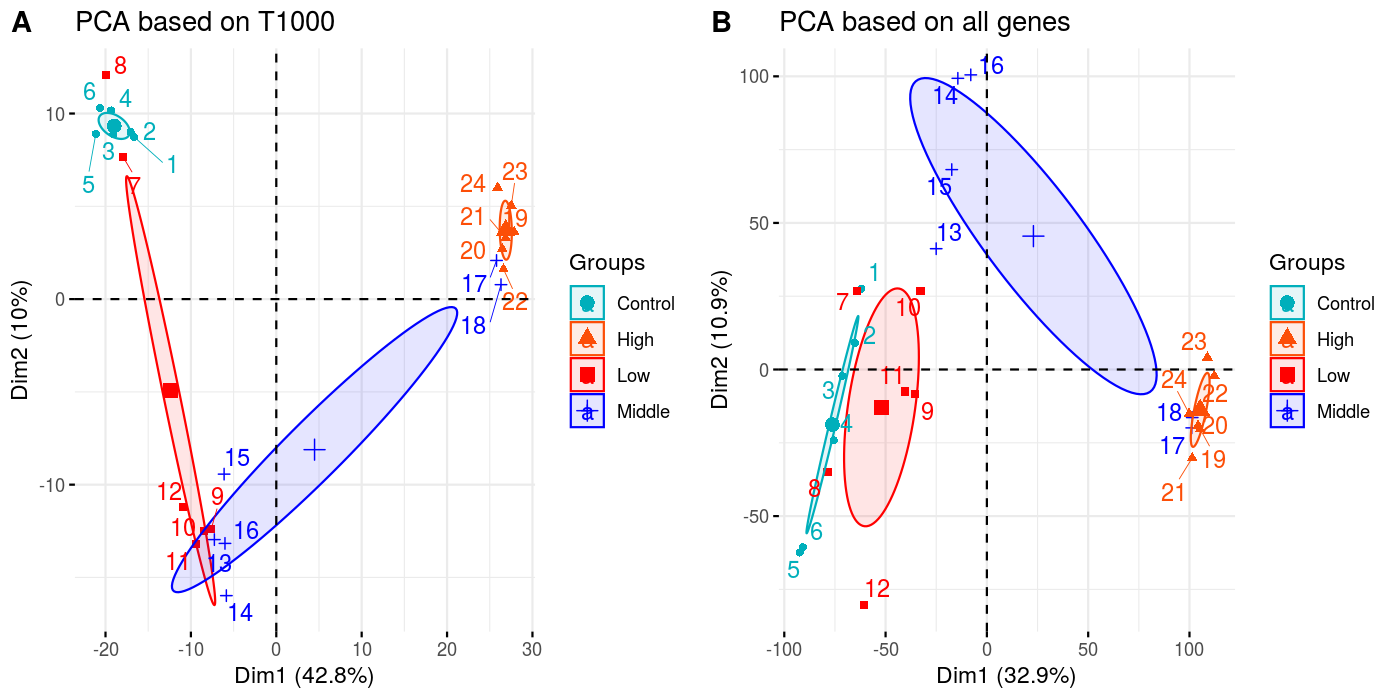

Supplement: Figure S5 [file peerj-07-7975-s005.zip › Supplementary_Figures_S5/haloperidol.Human.in_vitro.Liver.tiff]
