# Supplementary material for: T1000: a reduced gene set prioritized for toxicogenomic studies
Source: PeerJ. 2019 Oct 29;7:e7975. doi: 10.7717/peerj.7975 (PMC6824333; doi:10.7717/peerj.7975)
Supplement: Supplemental Information 1 [file peerj-07-7975-s006.docx]

# T1000: A reduced gene set prioritized for toxicogenomic studies

# ^1^Othman Soufan, ^2^Jessica Ewald, ^1^Charles Viau, ^3^Doug Crump, ^4^Markus Hecker, ^2,*^Niladri Basu and ^1,5,*^Jianguo Xia

^1^Institute of Parasitology, McGill University, Montreal, Quebec, Canada; ^2^Faculty of Agricultural and Environmental Sciences, McGill University, Montreal, Quebec, Canada; ^3^Ecotoxicology and Wildlife Health Division, Environment and Climate Change Canada, National Wildlife Research Centre, Carleton University, Ottawa, Canada; ^4^School of the Environment & Sustainability and Toxicology Centre, University of Saskatchewan, Saskatoon, Canada; ^5^Department of Animal Science, McGill University, Montreal, Quebec, Canada.

Supplemental Information S1

For building a binary classification model, the target variable of decision should be scaled to binary values. In the case of *in vitro* experiments, LDH% scores are provided in a continuous scale. When examining the values of LDH% across different doses as illustrated in Supplementary Information Figure 1 and Supplementary Information Figure 2, we notice that LDH% dramatically increases and decreases when dose becomes high, in general. Since LDH% reflects the relative survival rate, one expects drops in LDH% with high dose. The increase in LDH% with high doses could be the result of uncommon conditions or experimental error. So, we decided to consider all of these cases as part of the selection of profiles that represent dysregulated values of LDH%. As a result, we applied a threshold to convert LDH% from continuous scale to binary one (i.e. dysregulated vs. non-dysregulated). In machine learning, regression models can handle continuous values. Given that we wanted i*n vitro* models that can integrate with *in vivo* experiments which are discrete in nature, we gave preference to the binarization of LDH%. LDH% below 95% and higher than 105% were considered as the dysregulated cases.

Regardless of the specific choice of the binarization thresholds, it should be noted that LDH response were not used for performance evaluation in any stage. The LDH variable server only the selection process and was not used in evaluation to avoid potential bias after the binarization applied.


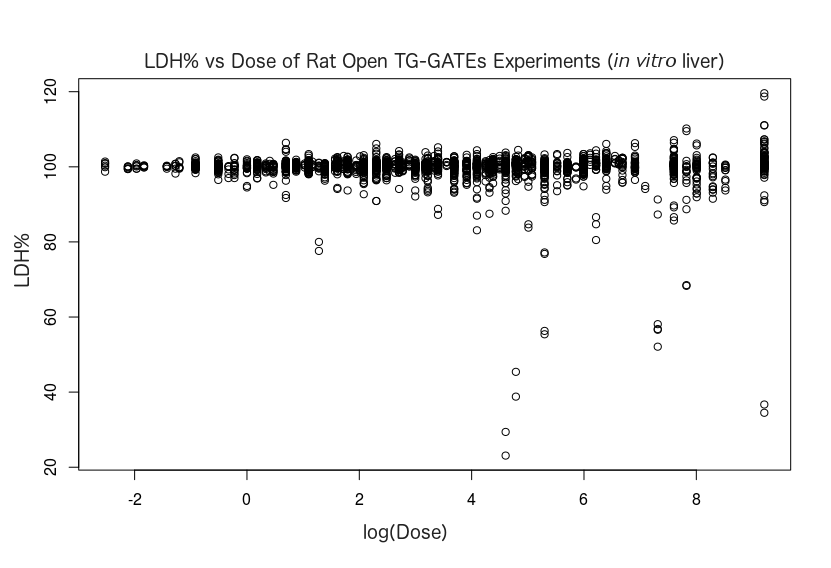


Supplementary Information Figure 1: LDH vs Dose in Human


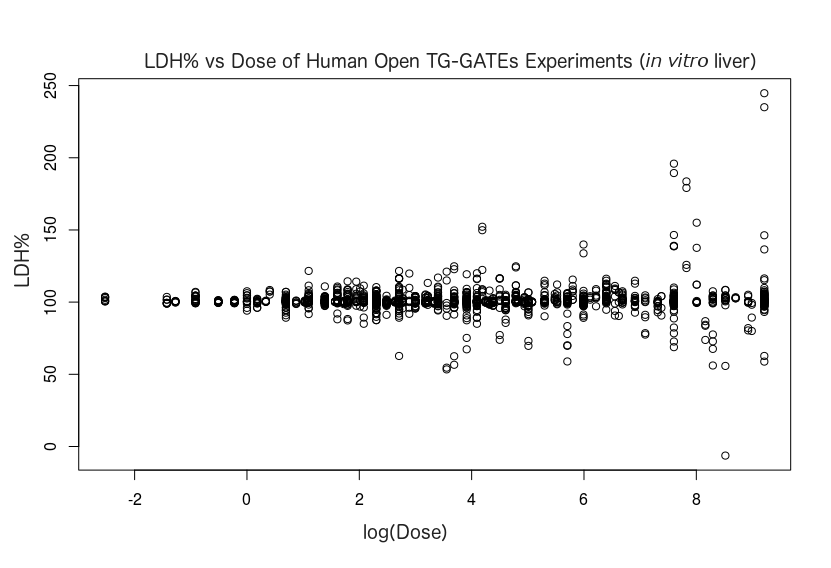


Supplementary Information Figure 2: LDH vs Dose in Rat
