# Supplementary material for: T1000: a reduced gene set prioritized for toxicogenomic studies
Source: PeerJ. 2019 Oct 29;7:e7975. doi: 10.7717/peerj.7975 (PMC6824333; doi:10.7717/peerj.7975)
Supplement: Supplemental Information 2 [file peerj-07-7975-s007.docx]

# T1000: A reduced gene set prioritized for toxicogenomic studies

# ^1^Othman Soufan, ^2^Jessica Ewald, ^1^Charles Viau, ^3^Doug Crump, ^4^Markus Hecker, ^2,*^Niladri Basu and ^1,5,*^Jianguo Xia

^1^Institute of Parasitology, McGill University, Montreal, Quebec, Canada; ^2^Faculty of Agricultural and Environmental Sciences, McGill University, Montreal, Quebec, Canada; ^3^Ecotoxicology and Wildlife Health Division, Environment and Climate Change Canada, National Wildlife Research Centre, Carleton University, Ottawa, Canada; ^4^School of the Environment & Sustainability and Toxicology Centre, University of Saskatchewan, Saskatoon, Canada; ^5^Department of Animal Science, McGill University, Montreal, Quebec, Canada.

Supplemental Information S2

In the following figure, the plot of the different iterations for computing prior scores is illustrated. At each step, most distant (i.e. most contributing) genes are selected, removed from the dataset and then the process of applying dimensionality reduction using PCA followed by K-means clustering is repeated. For Part A, a 2-Dimensional visualization using PCA is provided for the information prepared for the genes. Each single point represents the prior information encoded and gathered from CTD, KEGG and Hallmark for a corresponding single gene. The green, light blue and light red colors reflects the three clusters after applying K-means. The black squares are the centroids (or Cen.) for these clusters. The blue crosses are the most distant points (or Out. for outliers). After the Out. points are removed and the process is repeated for a second time, we get to Itr2 as illustrated in Part B. In Part C, we show the statistics for 100 iterations to see that the proposed features well characterize Out. from Cent. genes and that Out. genes have more prominent effects indicated by the light blue curves. In part A, we have highlighted the gene degree, Hallmark and KEGG coverage for the distant (or outlier) points and the centroids. The distant points have clearly larger values. A higher degree would indicate a gene influencing many other genes. A higher Hallmark and KEGG percentage would refer to a gene that is reported to be part of more gene sets or pathways, respectively.


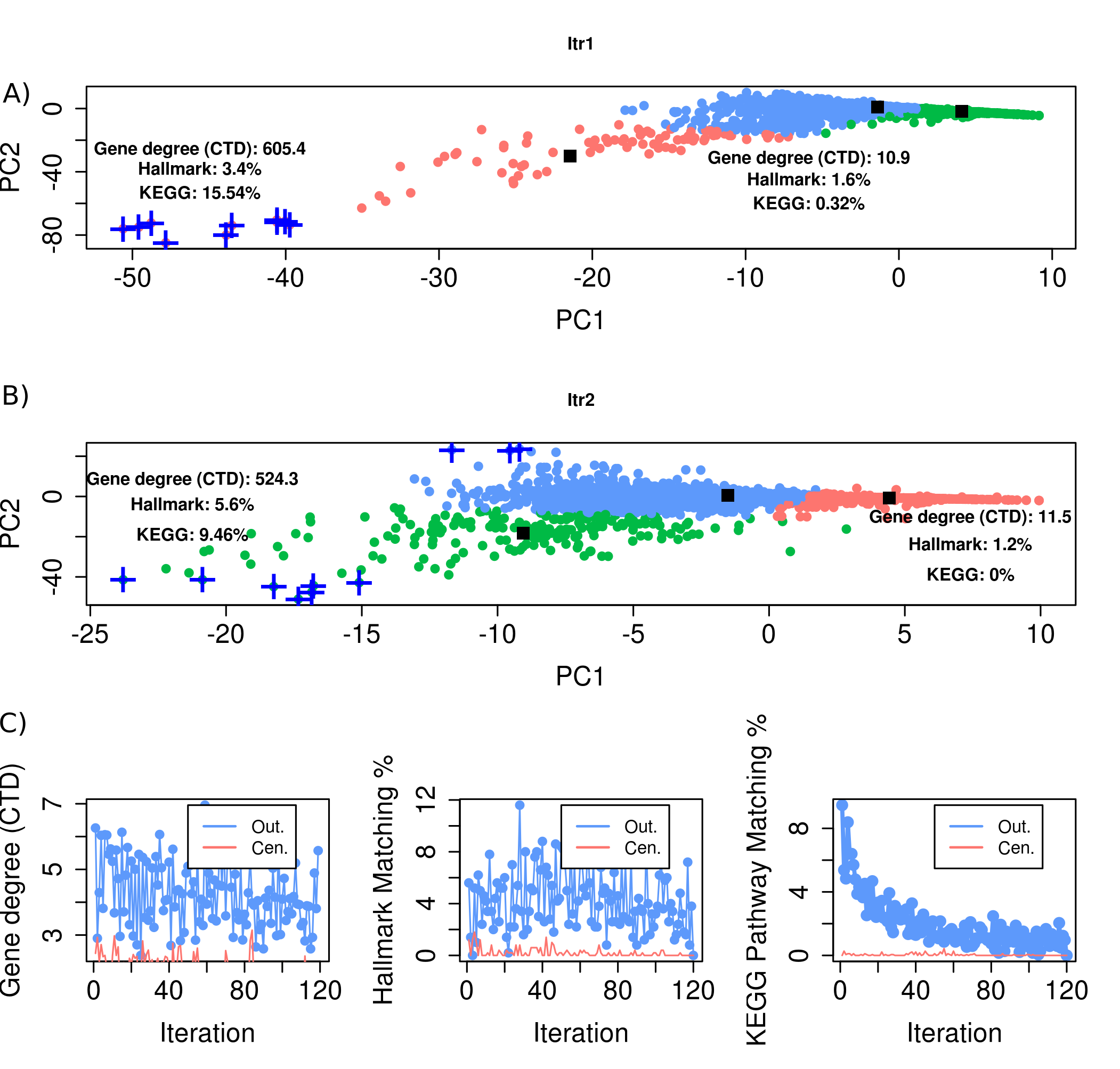


Supplemental Information S2 Figure 1: Plot of the different iterations for computing prior scores.

Thus, we used the Euclidean distance of genes from the cluster centroids to rank genes based on the prior knowledge space. Another possibility is to normalize distance by the cluster size. The ranked list was used to generate prior scores such that the first ranked gene would have a prior score of 100% and the last ranked gene would have a prior score close to 0%. The computational steps for computing the prior score are shown in **Supplemental Information 1**. Although the focus was on prioritizing 1000 genes, at this stage of building the prior knowledge, it was necessary to collect information for all potentially relevant genes. Thus, this was done for 22,336 genes.
