# Supplementary material for: T1000: a reduced gene set prioritized for toxicogenomic studies
Source: PeerJ. 2019 Oct 29;7:e7975. doi: 10.7717/peerj.7975 (PMC6824333; doi:10.7717/peerj.7975)
Supplement: Supplemental Information 5 [file peerj-07-7975-s010.docx]

# T1000: A reduced gene set prioritized for toxicogenomic studies

# ^1^Othman Soufan, ^2^Jessica Ewald, ^1^Charles Viau, ^3^Doug Crump, ^4^Markus Hecker, ^2,*^Niladri Basu and ^1,5,*^Jianguo Xia

^1^Institute of Parasitology, McGill University, Montreal, Quebec, Canada; ^2^Faculty of Agricultural and Environmental Sciences, McGill University, Montreal, Quebec, Canada; ^3^Ecotoxicology and Wildlife Health Division, Environment and Climate Change Canada, National Wildlife Research Centre, Carleton University, Ottawa, Canada; ^4^School of the Environment & Sustainability and Toxicology Centre, University of Saskatchewan, Saskatoon, Canada; ^5^Department of Animal Science, McGill University, Montreal, Quebec, Canada.

Supplemental Information S5

Connectivity map is a widely used resource to query and compare signatures of a gene, a drug candidate, or a disease state to the database to discover and reveal potential connections among drugs, genes, and diseases (Lamb et al. 2006). T1000 as a toxicogenomics developed signature can be used to indicate drugs with potential toxic effects through mapping to reference profiles of diseases in connectivity map. This gives an example of clinical applications of T1000. DrugVsDisease R package (Pacini 2013) was used to match expression profiles and find significant list of drugs such that data is based on the connectivity map drug screening. We report the results in the following table.

| Drug | ES Distance | Indication | Globally Harmonized System (GHS) Classification | Toxicity Notes | Reference |
| --- | --- | --- | --- | --- | --- |
| Dextromethorphan | 0.846 | treatment of dry cough. | Acute Toxic (Danger) |  | DB00514 |
| Iloprost | 0.857 | treatment of pulmonary arterial hypertension. | Acute Toxic; health hazard (Danger) | Overdoses can lead to hypotension, headache, flushing, nausea, vomiting, and diarrhea. | CID 5311181; DB01088 |
| Zoxazolamine | 0.858 | Muscle relaxant | Irritant | [withdrawn](https://en.wikipedia.org/wiki/List_of_withdrawn_drugs) due to hepatotoxicit | CID 6103 |
| Meclocycline | 0.859 | Currently under investigation for the topical treatment of ulcerative oral mucositis | NA | NA | CID 54676539; DB13092 |
| Phenformin | 0.861 | A biguanide hypoglycemic agent with actions and uses similar to those of metformin. | NA | withdrawn or removed from the US and other market sfor reasons of safety or effectiveness. | CID 8249; |
| Enilconazole | 0.863 | veterinary medicine Antifungal | Corrosive; acute toxic; irritant; health hazard; environmental hazard | NA | CID 37175 |
| Isoetarine | 0.865 | For the treatment of asthma, wheezing, and chronic asthmatic bronchitis. | NA | Signs of overdose include tachycardia, palpitations, nausea, headache, and [epinephrine](https://pubchem.ncbi.nlm.nih.gov/compound/epinephrine)-like side effects. | CID 3762 |
| Laudanosine | 0.866 | NA | Acutate toxic | NA | CID 15548 |
| Cefoperazone | 0.867 | treatment of various bacterial infections caused by susceptible organisms in the body, including respiratory tract infections, peritonitis, skin infections, endometritis, and bacterial septicemia. | Irritant; Health Hazard | discontinued in the U.S. | CID 44187; DB01329 |

In the table, ES distance refers to the distance between the input profile based and corresponding reference profile using T1000 list of genes. The profile classification computed using DrugVsDisease over the internal database for Asthma expression profiles, returned the listed significant influencing drugs. As summarized in the table, out of the ten reported significant drugs, we see that 3 drugs (Zoxazolamine, Phenformin, Cefoperazone) have been already withdrawn from specific markets due to toxicity reports. Phenformin was withdrawn since it was reported to be hepatototoxic.

References

Lamb J, Crawford ED, Peck D, Modell JW, Blat IC, Wrobel MJ, Lerner J, Brunet JP, Subramanian A, Ross KN, Reich M, Hieronymus H, Wei G, Armstrong SA, Haggarty SJ, Clemons PA, Wei R, Carr SA, Lander ES, and Golub TR. 2006. The Connectivity Map: using gene-expression signatures to connect small molecules, genes, and disease. *Science* 313:1929-1935. 10.1126/science.1132939

Pacini C. 2013. DrugVsDisease: Comparison of disease and drug profiles using Gene set Enrichment Analysis. *Bioinformatics, R package version* 2.
