# Supplementary material for: T1000: a reduced gene set prioritized for toxicogenomic studies
Source: PeerJ. 2019 Oct 29;7:e7975. doi: 10.7717/peerj.7975 (PMC6824333; doi:10.7717/peerj.7975)
Supplement: Supplemental Information 7 [file peerj-07-7975-s012.docx]

# T1000: A reduced gene set prioritized for toxicogenomic studies

# ^1^Othman Soufan, ^2^Jessica Ewald, ^1^Charles Viau, ^3^Doug Crump, ^4^Markus Hecker, ^2,*^Niladri Basu and ^1,5,*^Jianguo Xia

^1^Institute of Parasitology, McGill University, Montreal, Quebec, Canada; ^2^Faculty of Agricultural and Environmental Sciences, McGill University, Montreal, Quebec, Canada; ^3^Ecotoxicology and Wildlife Health Division, Environment and Climate Change Canada, National Wildlife Research Centre, Carleton University, Ottawa, Canada; ^4^School of the Environment & Sustainability and Toxicology Centre, University of Saskatchewan, Saskatoon, Canada; ^5^Department of Animal Science, McGill University, Montreal, Quebec, Canada.

Supplemental Information S7

Each dataset of Open TG-GATES is presented, statistically, as a matrix with rows of experiments and columns of genes. For example, the human *in vitro* dataset of Open TG-GATES is composed of a matrix of 2,606 experiments x 20,502 genes. Here, we are interested to see if the expression matrix based on the different gene sets would be representative of the original space covered by all 20,502 genes. A common measure for such comparison can be performed using Pearson’s correlation coefficient of each pair of samples such that a sample from original matrix composed of 20,502 genes is compared with the one composed of 1,000 genes only (T1000 case). However, since the correlation coefficient cannot handle two samples where each is having a different number of genes, we need to unify this by projecting into a 3-dimenstional PCA so that the two samples would have the same number of variables (i.e., 3 in the case of 3-dimenstional PCA) (Liu *et al.*, 2015). The comparison here is based on the data matrices when a group of genes are extracted using T1000, L1000, S1500 and Limma versus the original gene expression matrix. If the sample projected using T1000 genes, for example, is highly correlated with the one projected using all genes, then we can conclude that the genes of T1000 are highly representative. We refer to this type of comparison as analysis of coverage of full gene expression space using a group of genes (Subramanian *et al.*, 2017).

In order to address the case where different gene sets are composed of different number of genes, we divide by a normalized factor to penalize larger number of genes. This is based on the assumption, without loss of generality, that when more genes are selected, a higher correlation score is achieved (Liu, et al., 2015). The normalized factor is based on number of genes divided by total number of genes in datasets (i.e., 1000/20,502 = 0.049 for T1000 and Limma, 978/20,502 = 0.048 for L1000 and 2861/20,502 = 0.14 for S1500). The average correlation scores are 92.4%, 90.1%, 94.5% and 87.9% for T1000, L1000, S1500 and Limma, respectively. This analysis was applied over the first three datasets of Open TG-GATES (see Table 1) and the correlations score are summarized using average. Regarding the strength of coverage of the different sets of genes when compared to the complete expression space, T1000 and L1000 achieved the highest ratios with T1000 being slightly higher as illustrated in **Figure 1**.


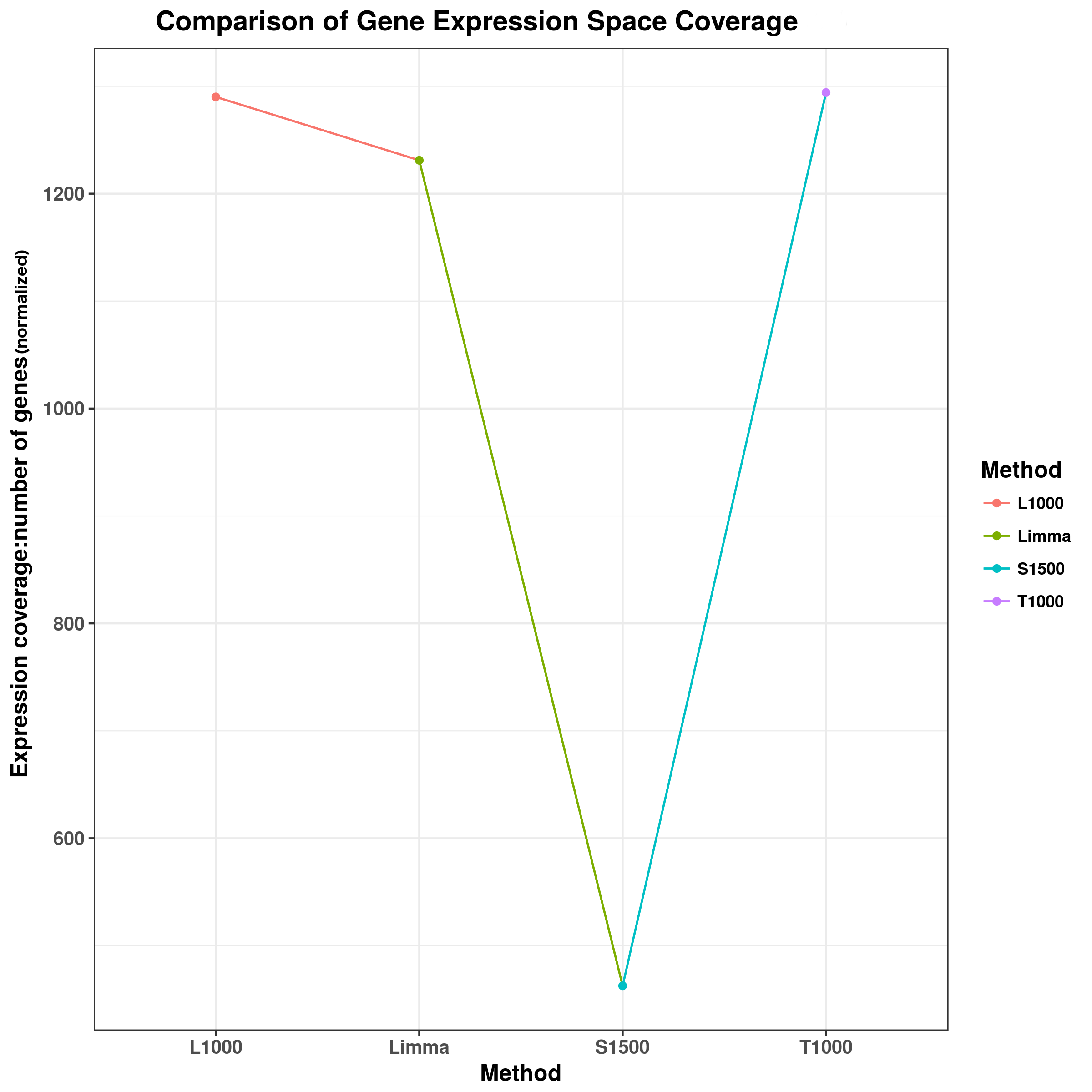


Figure 1: Comparison of gene expression space coverage. The pearson’s correlation between corresponding spaces in reduced (i.e., when using the gene set) and full gene expression space. For estimation of correlation, dimensionality reduction is applied over the expression matrices.

**Gene set coverage**

We performed two different analyses to understand the gene set coverage of T1000. First, to see if the expression patterns of T1000 genes were representative of the original space covered by all 20,502 genes (from Open TG-GATEs), we compared PCA plots for each chemical as per the example in **Figure 2**, which shows that the expression patterns were quite similar. This analysis was performed for all 158 chemicals from Open TG-GATEs with all plots provided in **Supplementary Figures 5**.


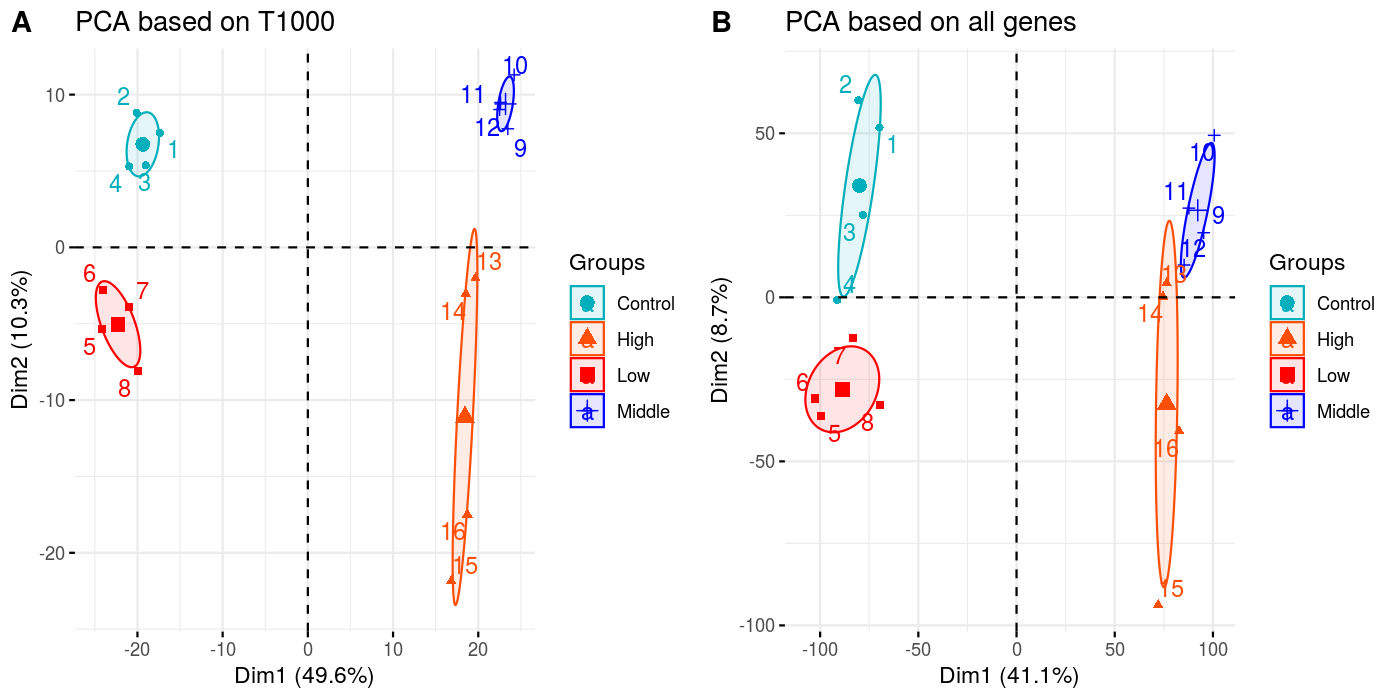


Figure 2: PCA plot for gene expression data derived from a 2-nitrofluorene Human in vitro experiment (Open TG-GATEs). Panel A shows the plot using T1000 genes only while panel B includes all 20,502 genes.

Next, we determined the biological space captured by T1000 by comparing it to reference libraries. The selected genes were mapped to Gene Ontology of Biological Processes (GO-BP), and then the number of significantly enriched gene sets (p-adjusted < 0.1) in T1000 was compared to those from S1500, L1000, Limma and Random. We also considered the Hallmark set, which addresses increased redundancy across, and heterogeneity within, the different gene sets. When general biological processes were examined (**Figure 3a**), T1000 did not outperform L1000 or S1500 in any iteration. However, T1000 did outperform other sets for most of the iterations for the Hallmark set coverage (**Figure 3b**).


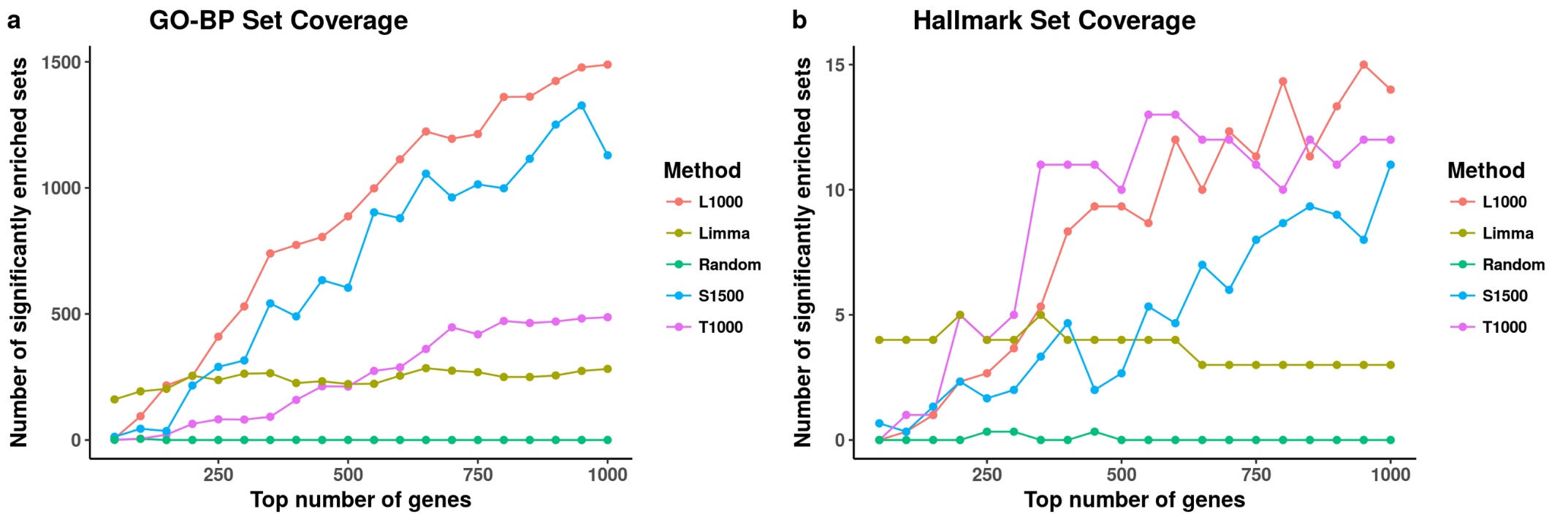


Figure 3: Comparison of number of significantly enriched gene sets (adjusted p-value < 0.1) within each of the selected gene sets. For gene sets in which ranking was not provided (e.g. L1000), the experiment was run several times and the average was reported.

When examining the eigengene (Figure 4) (i.e., first principle component) of both human and rat liver expression profiles for the 258 clusters, more gene profiles were represented by T1000 compared to the S1500 and L1000 gene sets. White gaps as in parts b, c and d of Figure 4 reflects clusters for which no genes were selected by the signature.


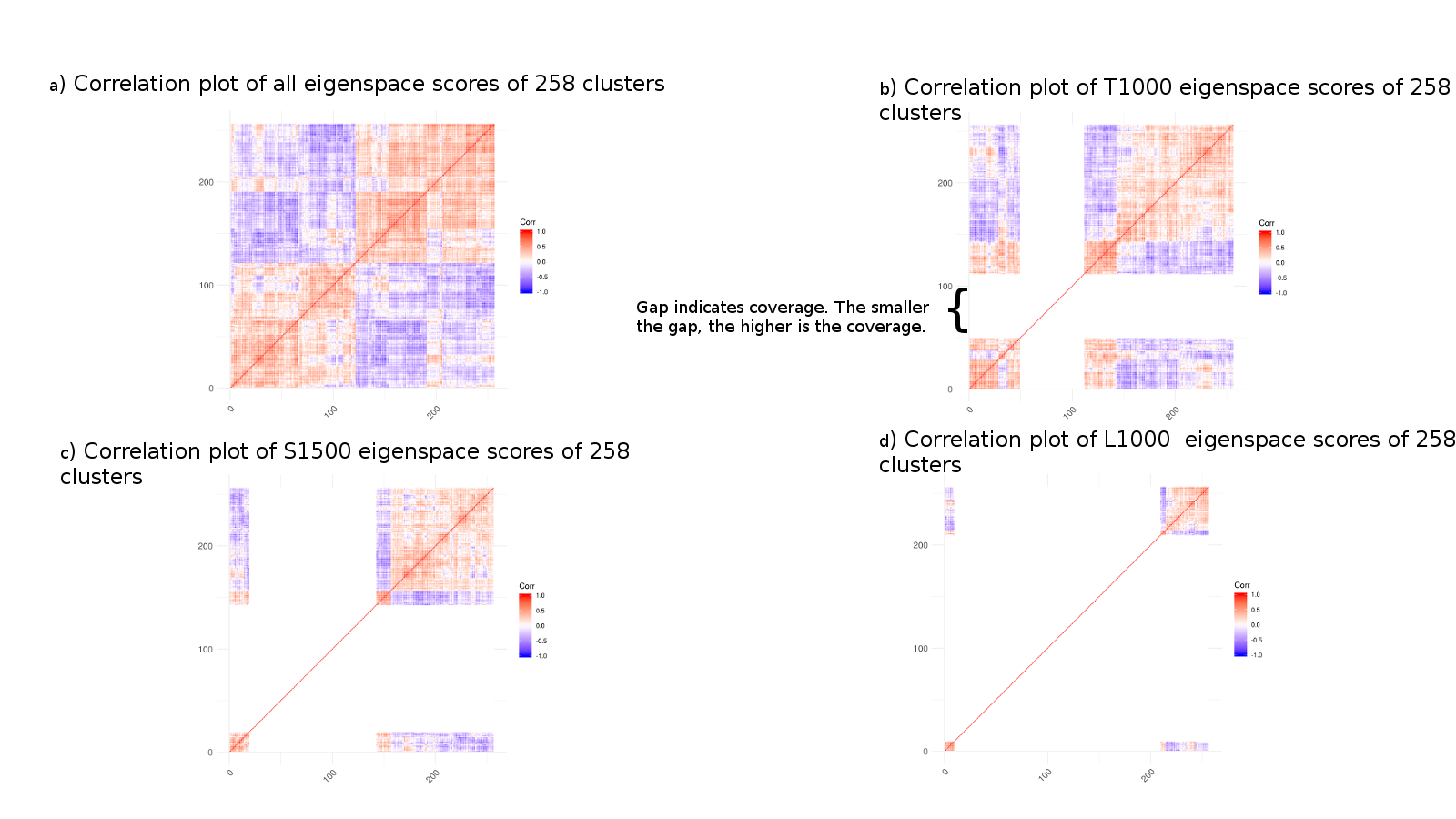


Figure 4: Correlation plots of the generated 256 clusters in across different gene signatures using first principle component of expression profiles (i.e., eigengene).

Liu, C., Su, J., Yang, F., Wei, K., Ma, J., and Zhou, X. (2015). Compound signature detection on LINCS L1000 big data. *Mol Biosyst* **11**(3), 714-22.

Subramanian, A., Narayan, R., Corsello, S. M., Peck, D. D., Natoli, T. E., Lu, X., Gould, J., Davis, J. F., Tubelli, A. A., Asiedu, J. K.*, et al.* (2017). A Next Generation Connectivity Map: L1000 Platform And The First 1,000,000 Profiles. *bioRxiv* doi: 10.1101/136168.
